# Photochemical Cyclization of Tertiary Buta‐2,3‐dienamides to β‐Lactams Upon Triplet Energy Transfer

**DOI:** 10.1002/anie.202525347

**Published:** 2026-02-17

**Authors:** Johannes Hofer, Maria‐Sophie Bertrams, Christoph Kerzig, Thorsten Bach

**Affiliations:** ^1^ Department Chemie and Catalysis Research Center (CRC) School of Natural Sciences Technische Universität München Garching Germany; ^2^ Department of Chemistry Johannes Gutenberg University Mainz Mainz Germany

**Keywords:** cyclization, C−H activation, lactams, photochemistry, time‐resolved spectroscopy

## Abstract

A series of *N*,*N*‐disubstituted buta‐2,3‐dienamides was prepared from 3‐butynoic acid and probed as substrates in a light‐induced photocyclization. It was found that xanthen‐9‐one (10 mol%) promotes the desired reaction to 3‐vinyl‐substituted 2‐azetidinones (β‐lactams) when performed at *λ* = 350 nm in acetonitrile as the solvent. Evidence was collected by transient absorption spectroscopy that the catalyst promotes excitation of the allene amide to its triplet state by Dexter energy transfer. Upon intramolecular hydrogen atom transfer from one of the nitrogen substituents, the ensuing 1,4‐diradical undergoes C─C bond formation to the lactam product. If the substituent at the nitrogen atom is a primary benzyl group, the product displays a stereogenic center in 4‐position and is formed exclusively as the *trans*‐product (eleven examples, 18%–73% yield). If the substituent is secondary, 4,4‐disubstituted products are formed. If the buta‐2,3‐dienamide is substituted at the terminal carbon atom, the substituent at C3 in the 2‐azetidinone is an (*E*)‐configured alkenyl group. Two alternative reaction pathways were observed, i.e. an intramolecular *para* photocycloaddition for *N*‐phenyl substituted substrates and an elimination from the 1,4‐diradical intermediate. The vinyl group at C3 can serve as useful handle for consecutive transformations.

## Graphical abstract

Energy transfer is the key to the successful photocyclization of the title compounds to 3,4‐disubstituted 2‐azetidinones. If the substituent at the nitrogen atom is primary (R^2^ = H), *trans* products are formed exclusively. The triplet state of xanthen‐9‐one (**XT**) was found to be quenched by an allene amide (R, R^2^ = H, R^1^ = Bn, and R^3^ = Ph) with a bimolecular rate constant *k*_q_ ∼ 1 × 10^9^ M^−1^ s^−1^.

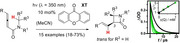

## 1 Introduction

**SCHEME 1:**
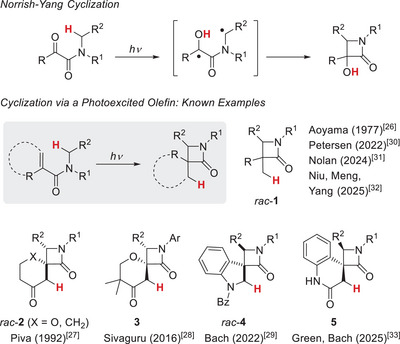
Norrish–Yang cyclization as an entry to β‐lactams (top) and previously studied photochemical cyclization reactions of α,β‐unsaturated or conjugated amides leading to β‐lactams (2‐azetidinones) with undefined (*rac*‐**1**, *rac*‐**2**, and *rac*‐**4**) or defined (**3**, **5**) absolute configuration.

A salient feature of photochemical reactions is the fact that they allow for the synthesis of strained compounds, which are challenging to access by thermal reactions. The [2+2] photocycloaddition to cyclobutanes [1, 2] and the Paternò–Büchi reaction to oxetanes [3, 4, 5] represent key transformations of organic photochemistry with an enormous synthetic scope and utility. Beyond cycloadditions, there are several photochemical cyclization reactions displaying unique reactivity patterns and paving the way to strained compounds. The Norrish–Yang cyclization (also referred to as Yang cyclization) [6, 7, 8, 9, 10, 11, 12] comprises a remote hydrogen abstraction by a photoexcited carbonyl compound and an ensuing cyclization. Although competing pathways exist, depending on the ring size and the nature of the connecting carbon atoms, the reaction can serve to prepare four‐membered hetero‐ and carbocyclic products. Its application to the synthesis of β‐lactams (Scheme 1) has been first reported in 1969 and has attracted substantial synthetic attention [13, 14, 15, 16, 17, 18, 19, 20, 21], including its use in enantioselective solid state photochemistry [22, 23, 24, 25]. Although the nπ* state of a ketone or aldehyde seems to be particularly well suited for hydrogen abstraction *via* its electrophilic oxygen atom, attempts have been made to involve also excited double bonds in a similar transformation leading to β‐lactams. Pioneering studies were reported by the groups of Aoyama [26] and Piva [27] who employed a direct excitation of the respective α,β‐unsaturated carbonyl compound to access racemic products *rac*‐**1** and *rac*‐**2**.

Both a direct excitation and an energy transfer approach were taken by Sivaguru and coworkers in their work on the photochemistry of atropisomeric *N*‐arylated enone carboxamides [28]. Here, the inherent axial chirality of the substrates induces a diastereoselective reaction course that provides enantioenriched products **3** from enantiopure starting material. More recently, several groups have investigated energy transfer catalysis as a tool to employ longer wavelength light for the photocyclization. Our group reported an access to spirocyclic indolines *rac*‐**4** employing thioxanthen‐9‐one as the catalyst [29]. The Petersen [30] and the Nolan group [31] revisited the cyclization of *N*‐benzyl acrylamides to products *rac*‐**1** and identified 2‐chlorothioxanthen‐9‐one and an *N*‐heterocyclic carbene‐ligated gold amido complex as catalysts that promote the photocyclization with visible light. Iridium energy transfer catalysis can be applied to the photocyclization, if the group R is an aryl group that lowers the triplet energy of the acrylamides [32]. A recent study showed that a thio‐xanthone‐containing enzyme is competent to process quinolone‐4‐carboxamides with high enantioselectivity, resulting in almost enantiopure (95%–99% *ee*) spirocyclic products **5** [33].

**SCHEME 2:**
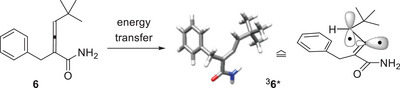
Upon energy transfer from a suitable sensitizer, 2,4‐disubstituted buta‐2,3‐dienamide **6** reaches a triplet state ^3^ 6* in which the central allene carbon atom becomes sp^2^‐hybridized and carries an unpaired electron in an sp^2^‐orbital [34].

When considering other α,β‐unsaturated carboxamides for a hydrogen atom transfer (HAT)/cyclization cascade, we were inspired by a previous study from our group on the deracemization of allene amides, such as compound **6** (Scheme 2) [34]. It had been found that energy transfer to the allene amide leads to population of the lowest lying triplet state ^3^ 6*, which was found by quantum‐chemical calculations to display a bent structure [35]. The electronic situation can be best described by two unpaired electrons, one of which is located in an allylic π‐orbital and the second of which resides in an sp^2^‐orbital at the central allene carbon atom. Since the latter radical has a high driving force to form a stable C─H bond, we envisioned triplet allene [36, 37, 38] amides to be potentially suited for a photocyclization. We now report in detail on the results of our study in which we interrogated the photocyclization of *N,N*‐disubstituted buta‐2,3‐dienamides.

## 2 Results and Discussion

### 2.1 Optimization Studies and Substrate Selection

The desired photocyclization was initially probed with *N*,*N*‐dibenzylbuta‐2,3‐dienamide (**7a**). It turned out that thioxanthen‐9‐one (**TXT**) was not suited to induce the desired cyclization in dichloromethane as the solvent. At *λ* = 420 nm, the starting material was fully recovered (Table 1, entry 1), possibly indicating that the substrate displays a higher triplet energy than the previously studied amides, such as **6**. In the latter case, energy transfer from **TXT** had been successful and had led to the above‐mentioned (de)racemization [34]. The reported triplet energy of **TXT** is *E*_T_ = 274 kJ mol^−1^ (methylcyclohexane‐isopentane, 77 K) [39]. In the same vein, a variety of iridium catalysts with triplet energies between 231 kJ mol^−1^ ≤ *E*_T_ ≤ 251 kJ mol^−1^ failed to facilitate a significant conversion under various conditions (see the Supporting Information for a full set of the optimization studies). Since the suspicion that allene **7a** might display a triplet energy *E*_T_ ≥ 290 kJ mol^−1^ was supported by calculations (*vide infra*), sensitizers with higher lying triplet states were tested.

**TABLE 1:** Optimization of reaction conditions for the photocyclization of tertiary buta‐2,3‐dienamide **7a** to β‐lactam *rac*‐**8a**.

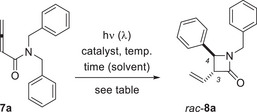			

Entry^a^	cat.^b^	Temp. [°C]	Solvent	*λ* [nm]	Time [h]	Yield^c^ [%]
1	**TXT**	30	CH_2_Cl_2_	420	24	—
2	**XT**	30	CH_2_Cl_2_	366	24	50
3	**XT**	30	MeCN	366	8	63
4	**XT**	30	PhCF_3_	366	16	62
5	**XT**	30	PhH	366	18	55
6	**XT**	−10	MeCN	366	24	64
7	**XT** ^d^, ^f^	30	MeCN	366	17	71
8	**XT** ^e^	30	MeCN	366	19	61
9	**TXT″** ^d^	30	MeCN	366	20	65
10	**XT** ^d^	30	MeCN	350	1.5	73
11	**TXT″** ^d^	30	MeCN	350	3	64
12	**ACP** ^f^	30	MeCN	350	23	41

^a^ Unless otherwise noted, optimization experiments were conducted on a scale of ≥100 µmol (*c* = 20 mM) under the indicated conditions with 20 mol% catalyst. Irradiation was performed with fluorescent lamps with an emission maximum at the given wavelength λ.

^b^ **TXT** = thioxanthen‐9‐one; **XT** = xanthen‐9‐one, **TXT″** = 3,3’‐dimethoxythioxanthen‐9‐one, and **ACP** = acetophenone.

^c^ Yield of isolated product after chromatography.

^d^ 10 mol% catalyst.

^e^ 5 mol% catalyst.

^f^ *c* = 10 mM.

Based on our previous experience with triplet energy transfer catalysis [40, 41], xanthen‐9‐one (**XT**, xanthone) seemed to be an ideal choice. Its triplet state energy is reported as *E*_T_ = 310 kJ mol^−1^ (3‐methylpentane, 77 K) [39], and the triplet state has a relative long lifetime *τ* in the absence of oxygen (*τ* > 4 µs in MeCN, *vide infra*) [42, 43]. When applying 20 mol% **XT** and an excitation wavelength of *λ* = 366 nm, we indeed observed conversion to the desired β‐lactam *rac*‐**8a**, irrespective of the choice of solvent (entries 2–5). Since the reaction proceeded fastest in acetonitrile, further optimization reactions were performed in the latter solvent. Lowering the reaction temperature (entry 6) led to a notable decrease in reaction rate without improving the yield. A decrease in catalyst loading to 10 mol% had a similar effect on the reaction rate, but the yield of isolated product was higher (entry 7). A further reduction of the catalyst to 5 mol% loading turned out to be detrimental to the yield (entry 8). Given the propensity of **XT** to abstract hydrogen atoms in its nπ* triplet [44], thioxanthone **TXT’** (3,3’‐dimethoxythioxanthen‐9‐one) was employed as an alternative catalyst with a relatively high triplet state energy (*E*_T_ = 298 kJ mol^−1^, methylcyclohexane, 77 K) [45]. The outcome (entry 9) was similar to the result achieved with **XT** at the same catalyst loading (entry 7) but not better. The UV–vis absorption properties of **XT** (*ε* = 6330 M^−1^ cm^−1^ at *λ*_max_ = 338 nm) in acetonitrile suggested to attempt an irradiation at shorter wavelength, i.e. closer to its local absorption maximum. In fact, at *λ* = 350 nm, the reaction was considerably faster, and full conversion was recorded after an irradiation time of only 90 min. The yield was 73% under these conditions (entry 10). Neither **TXT″** (entry 11) nor acetophenone (**ACP**) with a triplet energy of *E*_T_ = 308 kJ mol^−1^ (methylcyclohexane‐isopentane, 77 K) [39] (entry 12) performed better than **XT**, which is why we settled with the conditions of entry 10 as being optimal for the desired transformation. The reaction was susceptible to scale‐up. If run on gram scale (4.0 mmol), the yield was 68%.

In all reactions, the isolated product *rac*‐**8a** turned out to be a single diastereoisomer, and its relative configuration was assigned based on comparison with known NMR data [46, 47, 48, 49]. The low coupling constant (^3^ *J*_HH_ = 2.0–2.5 Hz) of the hydrogen atoms at C3 and C4 are indicative for the *trans*‐configuration while the *cis*‐isomer displays a larger coupling constant (^3^ *J*_HH_ ≥ 5.0 Hz). The assignment agrees with nuclear Overhauser effect spectroscopy (NOESY) experiments performed with product *rac*‐**8a** and with additional products obtained during the study (see the Supporting Information for further details).

### 2.2 Reaction Scope and Limitations

**FIGURE 1:**
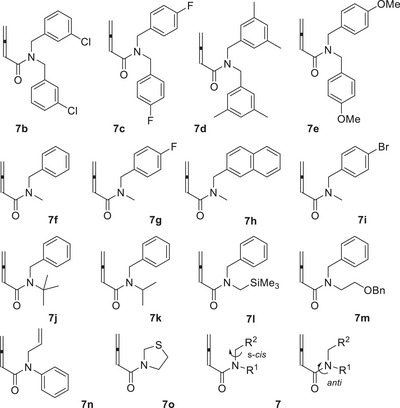
Structures of synthesized buta‐2,3‐dienamides **7** without additional substituents at the allene core and conformation required for a successful HAT.

With optimized conditions established for the conversion **7a** → *rac*‐**8a**, we considered the synthesis of diversely substituted buta‐2,3‐dienamides **7** for an exploration of the reaction scope. Since we felt a vinyl substituent at position C3 of the product lactams most useful, the allene was left unsubstituted. The synthesis of the compounds was achieved in two steps from 3‐butynoic acid (see the Supporting Information for details). The first set of compounds (**7b**‐**7e**) was prepared to study the electronic influence of substituents in an *N*,*N*‐dibenzyl‐substituted amide. With substrates **7f**‐**7i**, it was probed whether it is sufficient to have a single benzyl group at the nitrogen atom and whether the cyclization also occurs via HAT at the methyl group. Allenes **7j**‐**7m** served to check the regioselectivity of the photocyclization for other *N*‐alkyl groups beyond methyl. It was to be seen whether the reaction occurred exclusively at the benzyl group. Finally, substrates **7n**‐**7o** without a benzyl group were evaluated. Since most allenes **7b**‐**7o** bear a functional group, the compatibility with specific functional groups was interrogated in their putative photocyclization. A successful HAT requires an s‐*cis*‐conformation of R^2^ (relative to R^1^) and the shown *anti*‐amide rotamer of **7** (Figure 1).

**SCHEME 3:**
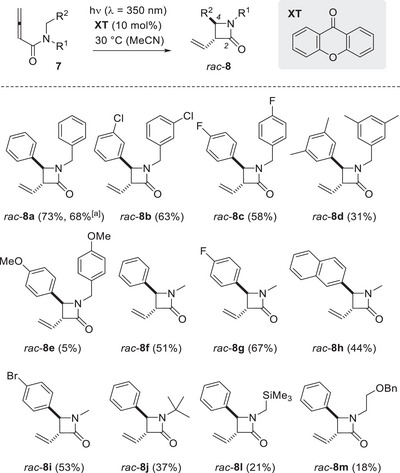
Diastereoselective formation of *trans*‐3,4‐disubstituted β‐lactams (2‐azetidinones) *rac*‐**8** by photocatalytic cyclization of buta‐2,3‐dienamides **7**. The reaction was performed with fluorescent lamps displaying an emission maximum at *λ* = 350 nm at a substrate concentration of *c* = 20 mm (see the Supporting Information for details) on a scale of ≥100 µmol. Irradiation was continued until no starting material was detected by TLC (*t* = 1.5 to 30 h). [a] The reaction was performed with 4.0 mmol substrate **7a**.

The photocyclization of buta‐2,3‐dienamides **7** was performed consistently with a catalyst loading of 10 mol% **XT** and at *λ* = 350 nm in MeCN as the solvent (*c* = 20 mM). Irradiation was continued until no starting material was detected by TLC. Although this procedure avoided separation and re‐isolation of starting material, it implicitly challenged the stability of the products under the reaction conditions. Typically, lower yields were recorded in cases in which irradiation had to be continued for 3 h and beyond. The reactions to products *rac*‐**8b** and *rac*‐**8c** could be terminated already after 3 and 2.5 h, respectively, which correlated with a good yield of 63% and 58% (Scheme 3). Substrate **7d**, however, required 5 h for the reaction to be completed, and the yield of product *rac*‐**8d** was lower (31%). Allene **7e** revealed a first limitation of the method that relates to the incompatibility of electron rich substrates with the **XT** catalyst. We assume the oxidative power of **XT** is too high, and photoredox processes prevail. In fact, based on the known triplet energy of **XT** and its ground state redox potential E_1/2_ (**XT**/**XT^•^** ^−^) = −1.74 V (vs. SCE, MeCN) [50], the calculated redox potential in the excited state is E_1/2_ (XT*/**XT^•^** ^−^) = +1.47 V (vs. SCE). Product *rac*‐**8e** was obtained in a yield of only 5%.

Because of the more stable C─H bond in methyl‐ [e.g. H─CH_2_NH_2_, bond dissociation energy (BDE) = 392 kJ mol^−1^] [51] versus benzyl‐substituted amino compounds (H─CHPhNH_2_, BDE = 368 kJ mol^−1^), only the benzyl group was found to be involved in the cyclization for the *N*‐methylated substrates **7f**‐**7i**. However, since there is only one benzylic nitrogen substituent, only the *anti*‐amide rotamer is competent to do the HAT. With a rotamer ratio of approx. 1/1, the process is statistically disfavored compared to substrates **7a**‐**7e**. Prolonged reaction times of up to 30 h (product *rac*‐**8 h**) were required, which led to a decrease in yield for some substrates (44%–67%). The functional group tolerance toward halogen substituents within substrates **7b**, **7c**, **7g**, **7i** was high. If the substituent R^1^ at the nitrogen atom gets bulkier, the required s‐*cis*‐conformation of the phenyl group (R^2^) is disfavored. In addition, if R^1^ displays abstractable hydrogen atoms, a second reaction pathway is accessible for the other amide rotamer (*vide infra*). For substrate **7j**, the former aspect compromises its reactivity as seen by prolonged irradiation (*t* = 6 h) and a yield of 37% for product *rac*‐**8j**. Allenes **7l** and **7m**, which display either a trimethylsilylmethyl or a benzyloxy(BnO)‐substituted ethyl group at the nitrogen atom (R^1^), reacted with low chemoselectivity, and decomposition was observed. Although oxidation might be an issue in the formation of product *rac*‐**8m**, the lack of selectivity for **7l** is likely due to the trimethylsilylmethyl group favoring the substrate to be in an s‐*trans*‐conformation and rendering a HAT from the benzyl group disfavored.

**SCHEME 4:**
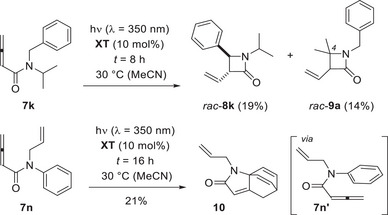
Photochemical reaction of buta‐2,3‐dienamides **7k** and **7n**: Formation of *trans*‐3,4‐disubstitued β‐lactam *rac*‐**8k** and of 3,4,4‐trisubstituted product *rac*‐**9a** (top). Intramolecular formal [4+2] cycloaddition reaction to tricyclic product **10** via amide conformer **7n’**.

Although primary and tertiary alkyl group R^1^ do not invite a hydrogen abstraction as prelude to the photocyclization, it was found for the isopropyl group that its secondary carbon atom can be involved in the reaction (Scheme 4). Upon irradiation of substrate **7k**, the expected product *rac*‐**8k** was isolated after a relatively long irradiation time (*t* = 6 h) in only 19% yield. However, a second product was obtained which was identified as the 4,4‐disubstituted 2‐azetidinone *rac*‐**9a**. Here, the isopropyl group appears to serve as hydrogen atom donor to the photoexcited allene (H─CMe_2_NH_2_, BDE = 372 kJ mol^−1^) [51]. The finding encouraged us to study buta‐2,3‐dienamides with secondary alkyl groups at the nitrogen atom (*vide infra*).

Replacing the benzyl group by an allyl group as potential site for a cyclization turned out to be difficult. The respective *N*,*N*‐diallyl‐ and *N*‐allyl‐*N‐*methylbuta‐2,3‐dienamides could not be prepared by our standard procedure. The *N*‐allyl‐*N*‐phenyl‐substituted compound **7n** was successfully synthesized [52, 53] but resisted the desired photocyclization by taking a different reaction pathway upon sensitized irradiation. The known [52] *para* cycloaddition product **10** was generated instead. It is noteworthy that we could rule out that the reaction occurs thermally at 30°C, which indicates the involvement of a photoexcited allene in the addition, presumably via conformer **7n’**.

The strength of the C─H bond is clearly a decisive parameter for the allene photocyclization to occur. Only benzylic and secondary C─H bonds can be involved in the reaction. The silyl group in substrate **7l** is insufficient to lower the BDE of the C─H bond at the adjacent methylene group, and no regioisomer of *rac*‐**8l** was observed. The complete decomposition observed for 1,3‐thiazoli‐dine **7o** is associated with its oxidation‐sensitive sulfur atom.

The scope studies, thus, revealed that the method offers a general route to access *trans*‐substituted 3‐vinyl‐substituted β‐lactams *rac*‐**8** with R^2^ being an aryl and R^1^ a methyl, a primary or a tertiary alkyl group. The irradiation time is the key factor that governs the yield. Although the time was optimized for substrate **7a**, the reaction requires for best results to monitor the conversion not by irregular TLC, but by continuous GLC or HPLC controls. If clean product *rac*‐**8a** was submitted to the irradiation conditions for 24 h, only 68% of the material was recovered, underpinning the sensitivity of the β‐lactam products. In terms of functional group incompatibility, substrates with electron rich substituents were found to be incompatible with the **XT** catalyst and gave poor results.

**SCHEME 5:**
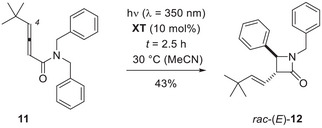
Upon irradiation in the presence of **XT**, 4‐substituted buta‐2,3‐dienamide **11** delivered product rac‐(*E*)‐**12** which was isolated in 43% yield. The starting compound **11** contained 12% of the respective 3‐butynoic amide as an impurity. Although *rac*‐(*Z*)‐**12** was detected in the crude product (*E*/*Z* = 78/22), it could not be obtained in pure form.

For the synthesis of 4‐substituted allene amide **11**, we attempted an amide bond formation at the known allenoic acid [54, 55] (5,5‐dimethylhexa‐2,3‐dienoic acid) via its acid chloride. Although the reaction worked, partial isomerization to the respective 2‐alkynamide was observed, which was not separable from the desired substrate. Since the amount of by‐product was small (9/1 in favor of **11**) and since we expected the alkyne to be unreactive in the photocyclization, the reaction was performed under standard conditions delivering β‐lactam *rac*‐**12** mainly as the (*E*)‐isomer (Scheme 5).

The diastereoselectivity (d.r. = diastereomeric ratio) was moderate (d.r. = *E*/*Z* = 78/22), and the isolation of the minor isomer in pure form was not possible. The major isomer rac‐(*E*)‐**12** was isolated in 43% yield. Although the reaction of **11** had delivered a single isolated product, it was clear that an additional substitution in 2‐position of the substrate would complicate the product analysis further. The diastereoselectivity in preliminary work with a 2‐substituted buta‐2,3‐dienamide was only moderate which meant that a mixture of four diastereomeric products was to be expected if a 2,4‐disubstituted buta‐2,3‐dienamide was used. In addition, we encountered difficulties when we attempted to prepare the tertiary amides in full analogy to the primary amides [34], such as **6**.

**SCHEME 6:**
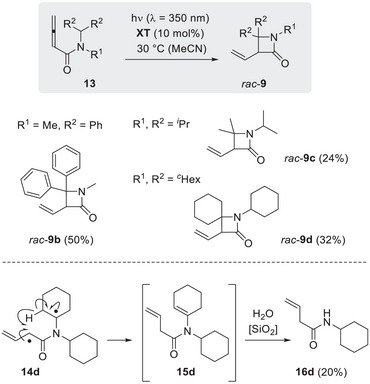
Buta‐2,3‐dienamides **13** with a secondary alkyl group at the nitrogen atom gave access to 3,4,4‐trisubstituted β‐lactams *rac*‐**9**. In the reaction of *N*,*N*‐dicyclohexylamide **13d**, the formation of a side product **16d** was observed, which likely results from hydrolysis of enamide **15d**.

The focus was, thus, shifted to *N*,*N*‐disubstituted buta‐2,3‐dienamides **13** with a secondary alkyl group at the nitrogen atom. The synthesis of the starting materials was performed in full analogy to the synthesis of amides **7**. The yield for the respective amides starting from 3‐butynoic acid was 24% for amides **13b** and **13d**, and 43% for amide **13c**. Irradiation of amide **13b** led to the expected product *rac*‐**9b** in 50% yield (Scheme 6). The moderate yield is likely due to the long irradiation time (*t* = 24 h) required to achieve complete conversion. The same applied also to the other two products which were isolated in 24% (*rac*‐**9c**, *t* = 18 h) and 32% yield (*rac*‐**9d**, *t* = 21 h). In the latter two cases, side product formation indicated a competitive reaction pathway. Secondary amide **16d** could be isolated in 20% from the reaction mixture produced by irradiation of substrate **13d**. The crude NMR spectrum suggested the enamide **15d** to be a precursor to the secondary amide, which originated from the former intermediate upon hydrolysis on silica gel. The formation of **15d** can be explained by the intermediacy of 1,4‐diradical **14d** which undergoes intramolecular hydrogen abstraction instead of cyclization. Although no clean product could be isolated from the reaction of substrate **13c**, it is reasonable to also assume the hydrogen abstraction as a competing process.

### 2.3 Mechanistic Experiments

**SCHEME 7:**
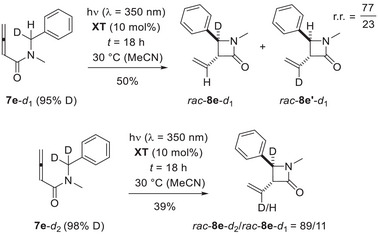
Photocyclization reactions performed with deuterated substrates **7e**‐*d*_2_ and **7e**‐*d*_1_: The monodeuterated substrate **7**‐*d*_1_, in which either hydrogen or deuterium atom transfer to the olefin is possible, shows a clear preference for hydrogen atom transfer (r.r. = regioisomeric ratio = *rac*‐**8e**‐*d*_1_/*rac*‐**8e’**‐*d*_1_; top). The reaction of **7e**‐*d*_2_ leads predominantly to the twofold‐deuterated product *rac*‐**8e**‐*d*_2_ although ^1^H NMR integration suggests the deuterium transfer to the olefinic carbon atom to be not perfect (bottom).

Our preliminary mechanistic hypothesis rested on a putative triplet energy transfer from photoexited triplet xanthone (^3^ XT*) to the allene followed by intramolecular hydrogen abstraction and cyclization. When studying the photocyclization of mono‐deuterated compound **7e**‐*d*_1_ (95% D), we isolated products *rac*‐**8e**‐*d*_1_ and *rac*‐**8e’**‐*d*_1_ as an inseparable mixture with traces of non‐deuterated product *rac*‐**8e** stemming from non‐deuterated starting material **7e** (Scheme 7).

The ratio of the two regioisomeric products reflects the propensity with which hydrogen abstraction occurs at the benzylic position. The primary kinetic isotope effect calculated from this value is 3.4 and relates to an intramolecular competition experiment [56]. Surprisingly, the photocyclization of the doubly deuterated substrate **7e**‐*d*_2_ delivered a product mixture in which the deuterium incorporation at the vinylic carbon atom was not complete. In previous experiments [29], we had seen a quantitative deuterium transfer under similar conditions. The result shed some doubt on the initial hypothesis of **XT** acting as an energy transfer catalysis and let us consider an initial HAT from the benzylic position to the photoexcited ^3^ XT* followed by a radical cyclization and a return HAT. Although any deuterium incorporation from the solvent was ruled out (see the Supporting Information for details), we felt it required to obtain additional evidence for an energy transfer pathway.

**FIGURE 2:**
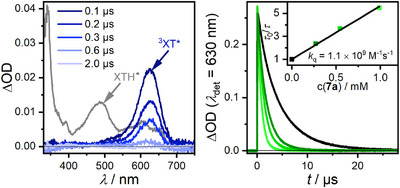
Left: TA spectra at different delay times after the excitation (355 nm, 10.5 mJ, and ∼5 ns pulse) of xanthone (**XT**, 90 µM) with allene amide **7a** as the quencher (9.7 mM) dissolved in argon‐saturated MeCN. The gray spectrum (**XT**, 4 mM; in the presence of 10 vol% 2‐propanol) is the reference spectrum of the protonated ketyl radical (**XTH^•^**), which would be the product of a HAT as primary quenching pathway and whose presence can be excluded. Right: Decay traces (detection at 630 nm) of ^3^XT* at different concentrations of the allene amide (excitation with 355 nm pulsed laser, 15 mJ). The stock solution contained xanthone (**XT**, 0.5 mM) and increasing concentrations of allene amide were added. The inset shows the resulting Stern–Volmer plot with the quencher concentrations being color‐coded.

Since the triplet state of **XT** is not phosphorescent in solution at ambient temperature, luminescence quenching studies do not reveal any information on triplet energy transfer. However, the transient signal of the **XT** triplet state can be nicely observed by transient absorption (TA) spectroscopy [44, 57]. We selectively excited **XT** with 355 nm laser pulses in argon‐saturated MeCN and observed its characteristic triplet TA spectrum [44, 57] with a maximum at 630 nm that decays with an intensity‐ and concentration‐dependent lifetime in the lower microsecond range (4–7 µs throughout our studies, see the Supporting Information). In the presence of high concentrations of **7a** ensuring almost quantitative (>90 %) ^3^ XT* quenching, the spectral features of ^3^ XT* returned fully back to the baseline and no long‐lived species was observed (left part of Figure 2).

The weakly pronounced ground‐state bleach peaking at ∼345 nm and the additional UV absorption band in the TA spectrum of ^3^ XT* (see Supporting Information for details) could not be used for the quenching analysis owing to pronounced filter effects resulting from **7a**. The absence of post‐quenching TA signals in the visible region excludes reaction mechanisms involving long‐lived radical species derived from **XT**. Specifically, the protonated xanthone ketyl radical (**XTH^•^**), which would be the primary product of ^3^ XT* quenching through a HAT from the benzylic position of **7a**, has distinct spectroscopic features (see the Supporting Information) [43, 58]. To obtain a reliable reference spectrum of **XTH^•^** under our conditions, we generated this species by using the potent hydrogen atom donor 2‐propanol as co‐solvent [44]. The absorption bands peaking at 640, 490, and 345 nm are clearly absent in the postquenching spectrum in Figure 2. Furthermore, the ketyl radical **XT^•^** ^−^, either generated through a photoinduced electron transfer (PET) or a HAT followed by deprotonation [59], would not escape detection because of its intense signatures in the whole detection range of our experiments [57]. In contrast to these HAT or PET quenching processes, our observations with baseline‐like postquenching spectra are in perfect agreement with a triplet energy transfer as main quenching pathway. All triplet or diradical species that could be formed with the quencher **7a** are expected to be essentially transparent in the visible region and short‐lived, such that the generation of these species is most likely slower than their decay kinetics, preventing the built‐up of detectable concentrations [60, 61].

**FIGURE 3:**
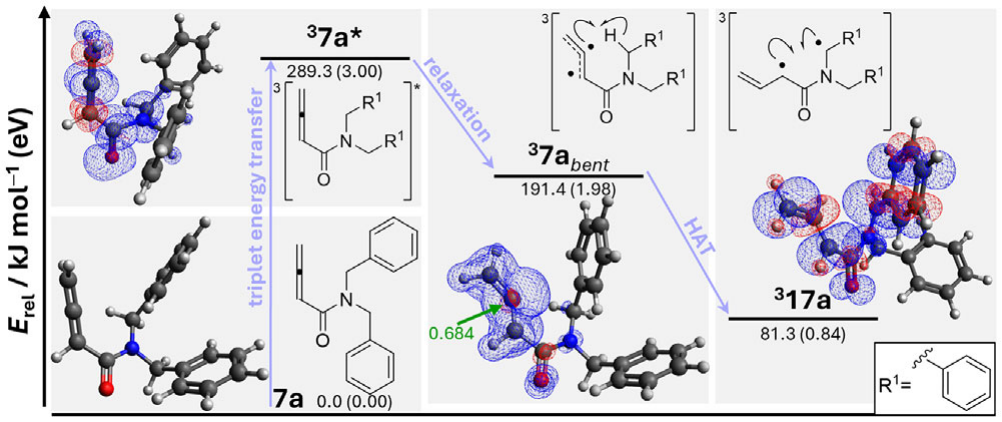
Mechanistic scheme for the described photochemical cyclization reactions with substrate **7a** as representative example and DFT‐calculated (level of theory: B3LYP, 6‐31 g) energy‐optimized key intermediates: Triplet energy transfer generates the pseudo‐vertical ^3^ 7a*, which relaxes to ^3^ **7a** *_bent_*. The latter undergoes an intramolecular HAT yielding diradical ^3^ **17a**, which then undergoes cyclization to β‐lactam *rac*‐**8a** after intersystem crossing. The energies were calculated relative to the energy of the optimized ground state of **7a**. The corresponding spin density plots (blue color denotes positive spin densities) are shown using an iso value of 0.002.

Owing to the above‐mentioned intensity‐ and concentration‐dependent ^3^ XT* lifetimes and some photodegradation issues during initial laser experiments (**XT** decomposition and **7a** depletion due to the investigated cyclization reaction), we carefully optimized the conditions for the quenching studies (see Supporting Information for details). As we found, a higher **XT** concentration (0.5 mM ensuring that about 20 % of the 355 nm laser light was absorbed) in conjunction with 15 mJ pulse energy (capable of exciting ∼10 % of all **XT** molecules in the laser beam) gave kinetic traces with sufficient signal‐to‐noise‐ratio after 10 laser pulses only. Importantly, both **XT** and the quencher **7a** are completely stable under these conditions, as is evident from comparative UV–vis absorption measurements before and after the TA experiments. A Stern–Volmer analysis of the ^3^ XT* quenching by **7a** under optimized conditions gave a quenching rate constant on the order of *k_q_* ∼ 1 × 10^9^ M^−1^ s^−1^ (right part of Figure 2). The UV–vis absorption band of **XT** is unaffected by the addition of **7a**, and the initial ^3^ XT* amplitude does not change during the quenching experiments. Static quenching can, thus, be excluded. The value obtained for dynamic quenching is more than one order of magnitude slower compared to the diffusion limit in MeCN (1.9 × 10^10^ M^−1^ s^−1^) [62], which implies that the triplet energy transfer has a low driving force or it is even slightly endergonic [63]. However, owing to the long unquenched lifetime of ^3^ XT* the overall quenching efficiency is expected to be close to unity under our standard photocatalysis conditions (20 mM of **7a**) and would be still on the order of 80% at 95% substrate conversion (corresponding to 1 mM, i.e. the highest concentration of **7a** used in Figure 2). Qualitatively, these estimates demonstrate that the efficiency of the initial quenching step in our mechanism is very high, taking potential errors of a four‐point Stern–Volmer analysis into account. The experimental triplet energy of **7a** could not be determined through 77 K phosphorescence measurements in a direct manner, which prompted us to perform DFT calculations. For molecules with a similar structure in the ground state and the triplet state, the calculated adiabatic triplet energy, *i.e*. the triplet energy obtained through the comparison of the DFT‐calculated energy of both optimized species, is known to reproduce experimental triplet energies extremely well [60, 64, 65, 66]. However, the situation is more complicated for the allene amides employed in the present study. Guided by sophisticated calculations on the sensitization of allene amide **6**, whose minimum triplet structure is bent and, thus, involves pronounced structural changes upon energy transfer (cf. Scheme 2) [34], we also found a bent minimum structure for the **7a** triplet (^3^ **7a** *_bent_* in Figure 3) at 191 kJ mol^−1^ (1.98 eV) above ground‐state **7a**. This behavior is somewhat reminiscent of sensitized olefin isomerization processes, in which a perpendicular minimum structure is obtained starting from either the (*E*)‐ or the (*Z*)‐isomer. Here, it was found that the experimental triplet energies are halfway between the computed vertical and adiabatic triplet energies [67]. The vertical triplet energy of **7a** using the unmodified ground‐state minimum structure is calculated to be 388 kJ mol^−1^ (4.03 eV). That energy would be too high for allowing any energy transfer quenching with ^3^ XT* (*E*_T_ = 310 kJ mol^−1^). Interestingly, we found a stable minimum structure at the triplet surface with a very similar geometry of the allene system compared to that of the ground state (^3^ 7a* in Figure 3). The triplet energy of this pseudo‐vertical species ^3^ 7a* is 289 kJ mol^−1^ (3.00 eV). Although this triplet energy might still underestimate the actual value being relevant for the quenching kinetics, it would provide a good explanation for our spectroscopic results obtained with **TXT** (*E*_T_ = 274 kJ mol^−1^) as the sensitizer. We indeed observed some ^3^ TXT* quenching by **7a** (see Supporting Information for details), but the quenching rate constant is estimated to be as slow as *k_q_* = 3 × 10^7^ M^−1^ s^−1^, *i.e*. about 40 times slower than observed for ^3^ XT*. The latter rate constant is clearly indicative for an uphill energy transfer and it could lead to fast back energy transfer from ^3^ 7a* to the sensitizer in its ground state [68].

The given rationale appears valid in the framework of the Sandros equation [63, 69], because in the absence of additional steric hinderance the rate of a bimolecular Dexter energy transfer typically follows the triplet energy levels. We stress that the calculated adiabatic triplet energy (i.e. that of ^3^ **7a** *_bent_*) is likely by at least 0.5 eV too low and cannot be correlated with experimental results, specifically with the observed rate constant difference between ^3^ **XT** * and ^3^ TXT* quenching and the fact that all visible light‐absorbing sensitizers with triplet energies well below that of **XT** and **TXT** (*E*_T_ < 250 kJ mol^−1^, e.g. Ir(ppy)_3_ and 4CzIPN, see the Supporting Information for details) do not catalyze the reaction.

Given the result of the TA study, we revisited the **TXT**‐catalyzed reaction in MeCN instead of CH_2_Cl_2_ as the solvent (cf. Table 1, entry 1). Under otherwise identical conditions, a low conversion was observed, and the product was isolated in 27% yield. We believe that these experimental results and calculations on a triplet species that can undergo pronounced structural changes upon sensitization will contribute to a better kinetic and mechanistic understanding of “unusual” triplet energy transfer processes, which are currently investigated in detail both experimentally and theoretically [68, 70, 71, 72, 73, 74, 75].

**FIGURE 4:**
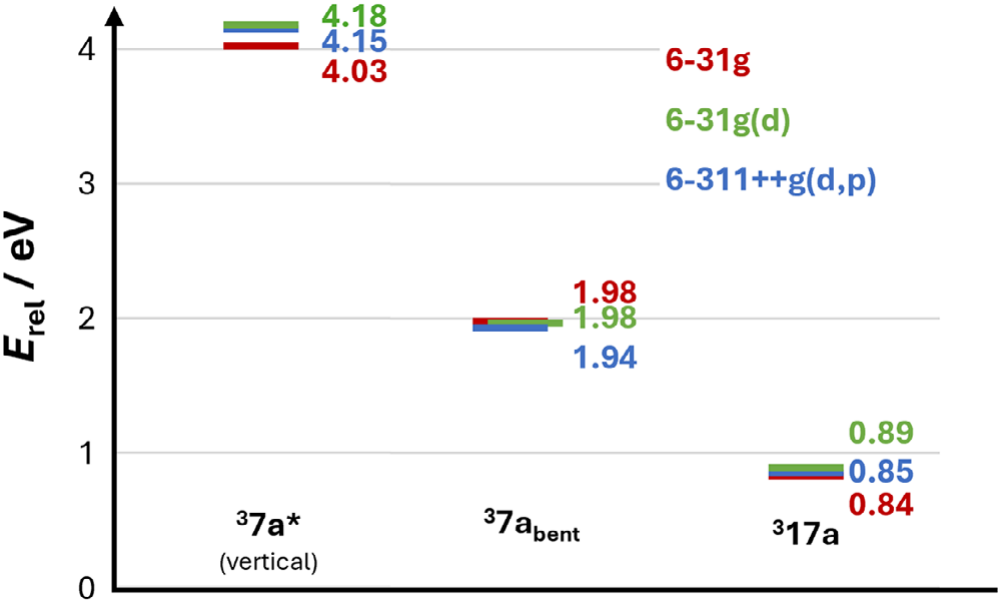
Comparison of calculated energies of triplet species using B3LYP as functional and different basis sets (color coded). The energy difference is given relative to the optimized ground state structure (**7a**) using the respective basis set for geometry optimization.

Additional DFT calculations proved useful for substantiating our assumptions regarding the fate of ^3^ 7a* in the cyclization reaction. The vertical triplet energies, and the structures as well as energies of ^3^ **7a** *_bent_* and ^3^ **17a** were calculated using extended basis sets with additional diffuse and polarization functions. The obtained structures, spin density distributions, and triplet energies (see Figure 4 and Supporting Information for details) are essentially identical regardless of the basis set, which implies that already the 6‐31 g basis set yields meaningful results. A closer look at the electronic structure of ^3^ **7a** *_bent_* revealed that the two unpaired electrons are mainly distributed over the allylic π‐system (sum of spin densities at the three carbon atoms, 1.79; see Supporting Information for details). The central allene carbon atom has the most pronounced radical character as indicated by the highest spin density (0.684), and the unpaired electron is localized in an sp^2^‐hybrid orbital at this atom, which was sp‐hybridized in the initial **7a** ground state. The spin densities at the two adjacent carbon atoms belonging to the allylic π‐system are localized in orbitals that are orthogonal to the orbital with the unpaired electron at the central carbon atom (for a tabular survey, see the Supporting Information). This electronic situation and the calculated bent structure are in line with previous investigations on triplet states of other allenes [34, 35].

The lack of stabilization of the unpaired electron at the central allene carbon atom and the close proximity to the benzylic hydrogen atom in the calculated structure for ^3^ **7a** *_bent_* (Figure 3) can be regarded as ideal properties for an intramolecular HAT. Thermodynamically, triplet diradical ^3^ **17a** as the HAT product is indeed lower in energy than ^3^ **7a** *_bent_* by more than 100 kJ mol^−1^. The DFT calculated structure of ^3^ **17a** displays the highest spin densities in the α‐positions next to the carbonyl carbon atom (0.65) and the nitrogen atom (0.71). The chemical structure in the upper right corner of Figure 3 with the 1,4‐diradical character is thus a meaningful representation of the calculated species ^3^ **17a**. Starting from ^3^ **17a** it seems plausible to assume that after intersystem crossing radical recombination yields the cyclization product *rac*‐**8a**. Like seen for most 1,4‐diradicals in triplet photocycloaddition reactions [2, 76], the bulky substituents (here vinyl and R^1^) have sufficient time to end up in a favorable *trans*‐configuration upon C─C bond formation.

### 2.4 Consecutive Reactions of Representative Product Rac‐**8a**

**SCHEME 8:**
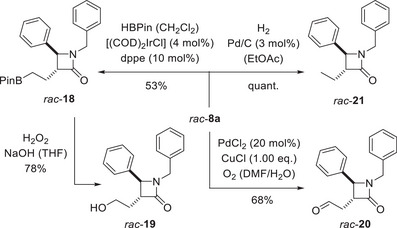
Consecutive reactions of compound *rac*‐**8a** underpinning the stability of the β‐lactam ring under oxidative and reductive conditions. Abbreviations: COD = 1,5‐cyclooctadiene; dppe = 1,2‐bis(diphenylphosphino)ethane.

Given that there is an extensive set of methods available for the functionalization of terminal C═C double bonds and that the reagent tolerance of β‐lactams is well explored [77, 78], we only performed a few preliminary experiments on the further functionalization of representative product *rac*‐**8a**. The focus was on transformations which would allow for a functionalization at the terminal olefin carbon atom. Boronate *rac*‐**18** was accessed by iridium‐catalyzed hydroboration [79] with pinacolborane (HBPin) and could be further transformed oxidatively into alcohol *rac*‐**19** (Scheme 8). An alternative oxidative functionalization of the terminal carbon atom was possible by a Pd‐catalyzed Wacker‐type oxidation [80] employing stoichiometric quantities of oxygen and CuCl. The desired terminal aldehyde *rac*‐**20** was obtained in good yield with perfect regioselectivity. The hydrogenation to the saturated ethyl‐substituted β‐lactam *rac*‐**21** proceeded quantitatively by applying hydrogen under atmospheric pressure and palladium on carbon as the catalyst [81].

## 3 Conclusion

In summary, we have shown that allene amides are suitable substrates for a cascade of intramolecular hydrogen abstraction and subsequent cyclization. The reaction proceeds from the triplet state of the allene which needs to be populated by energy transfer. Xanthen‐9‐one was identified as a suitable sensitizer that is capable to provide the high energy input required for sensitization. Experimental evidence and DFT calculations suggest the allene triplet state to be almost 300 kJ mol^−1^ above ground state, which cannot be accessed by applying more commonly used transition metal complexes as catalysts. Quenching experiments have provided a clear picture on the course of the energy transfer process, and it was found that the representative buta‐2,3‐dienamide **7a** quenches the xanthone triplet with a bimolecular rate constant *k_q_* = 1 × 10^9^ M^−1^ s^−1^. Given the massive energy deposited in the allene amide triplet, it is the more notable that its consecutive chemistry is relatively clean. Structurally, the triplet adopts a bent conformation from which the intramolecular hydrogen atom transfer step successfully competes with a decay to the ground state. The hydrogen atom of the amine substituents ends up at the internal carbon atom of the allene, and the ensuing 1,4‐diracidal undergoes cyclization to the β‐lactam ring. The reaction proceeds most smoothly if the C─H bond from which the hydrogen is abstracted is activated by an adjacent substituent. Benzylic and secondary C─H bonds have been identified as particularly efficient, and the respective substrates have provided the best yields. Intrinsically, the nature of the photoexcited xanthone with its high oxidative power and its aggressive carbonyl triplet state limits the functional group tolerance of the transformation. Still, the scope of the photocyclization is wide and relatively diverse, and the reaction paves a synthetically simple, concise route to functionalized β‐lactams. The synthesis of the starting materials requires only two steps from commercially available 3‐butynoic acid, and the products invited further reactions at the vinyl group, which can act as a useful synthetic handle for functionalization.

## Conflicts of Interest

The authors declare no conflicts of interest.

## Supporting information




**Supporting File 1**: anie71491‐sup‐0001‐SuppMat.pdf.

## Data Availability

The data sets that support the findings of this study are available in the main article and/or the Supporting Information. The laser spectroscopic data sets shown in the main part and DFT output files are accessible via the JGU library, they can be found under https://doi.org/10.25358/openscience-14316.
