# Supplementary material for: Photochemical Cyclization of Tertiary Buta‐2,3‐dienamides to β‐Lactams Upon Triplet Energy Transfer
Source: Angew Chem Int Ed Engl. 2026 Feb 17;65(13):e25347. doi: 10.1002/anie.202525347 (PMC13007592; doi:10.1002/anie.202525347)
Supplement: Supplementary file 1 — Supporting File 1: anie71491‐sup‐0001‐SuppMat.pdf. [file ANIE-65-e25347-s001.pdf]

Supporting Information for:

Photochemical Cyclization of Tertiary Buta-2,3-dienamides to β-Lactams upon Triplet Energy Transfer

Johannes Hofer,[a] Maria-Sophie Bertrams,[b] Christoph Kerzig[b]* and Thorsten Bach[a]*

[a] Department Chemie and Catalysis Research Center (CRC), School of Natural Sciences, Technische Universität München, D-85747 Garching, Germany

[b] Department of Chemistry, Johannes Gutenberg University Mainz, D-55128 Mainz, Germany

1. General Work Methods 3

2. Analytical Methods 5

3. Emission and Characteristics of the Light Sources (350 nm, 366 nm, 420 nm and 455 nm) 9

5. Reaction Condition Screening 14

6. UV/Vis-Spectra 16

7. Experimental Procedures and Characterization 17

7.1 Synthesis of the Photosubstrates 18

7.2 Intramolecular Cyclization of Tertiary Buta-2,3-dienamides to β-Lactams 45

7.3 Reactions of β-Lactam 8a 75

8. Deuteration Experiments 80

8.1 Deuteration Experiment with 7f-*d*$_2$ 80

8.2 Deuteration Experiment with 7f-*d*$_1$ 81

8.3 Photoreaction of 7a to 8a in Deuterated Solvent 82

9. Crude NMR of the Photoreaction 83

10. Stability of Photoproduct 8a under Irradiation Conditions 84

11. Transient Absorption Experiments 85

11.1 Triplet Excited State of Xanthone: 85

11.2 HAT Reactivity of Xanthone: ... 86
11.3 Additional Time-Resolved Experiments with Thioxanthone: ... 87
12. UV/Vis Control Experiments and Optimization of TA Conditions ... 87
12.1 Absorbance of XT at 355 nm ... 87
12.2 Different Excitation Intensities ... 89
13. DFT Calculations ... 94
13.1 General Information ... 94
13.2 Mulliken Spin Density Analysis ... 95
14. NMR Spectra of New Compounds ... 101
15. References ... 146

1. General Work Methods

All air- or moisture sensitive reactions were performed under argon atmosphere (argon 4.8, *Westfalen AG*). Reaction vessels were dried with a heat air blower under reduced pressure with about 650 °C. Moisture sensitive reactions were performed with dry solvents and standard *Schlenk* techniques were applied.

Solvents and reagents were used without additional purification, if not otherwise mentioned. The dry solvents diethylether (Et_2O), dichloromethane (CH_2Cl_2) and tetrahydrofuran (THF) were placed into the reaction vessel from a solvent purification system SPS-800 (*MBraun*) under argon-atmosphere. Following columns were used in order to remove water:

Dichloromethane (CH_2Cl_2): 2 × MB-KOL-M Typ 2 (Al_2O_3);

Tetrahydrofuran (THF): 2 × MB-KOL-A (Molecular sieve 3 Å);

Diethylether (Et_2O): 1 × MB-KOL-A Typ 2 (Al_2O_3)

1 × MB-KOL-A Typ 2 (Molecular sieve 3 Å);

Photoreactions were performed with anhydrous acetonitrile (MeCN), methanol (MeOH), benzene (PhH), α,α,α-trifluorotoluene ($PhCF_3$), acetone (ac), toluene ($PhCH_3$) and dichloromethane (CH_2Cl_2) as solvents. Dichloromethane (CH_2Cl_2) was taken from a solvent purifications system, degassed by three *Freeze-Pump-Thaw*-cycles and stored under argon and over activated molecular sieve (molecular sieve 4 Å). Prior to the start of a photoreaction, each reaction mixture was degassed by being sparged with argon under ultrasonication for 15 min.

The following dry solvents were obtained by the given companies in the corresponding qualities and used without further purification:

Acetonitrile (MeCN): *Thermo Scientific*, 99.9% extra dry, over molecular sieve;

Benzene (PhH): *Sigma Aldrich*, 99.8% anhydrous, <0.005% H_2O;

Dimethylformamide (DMF): *Thermo Scientific*, 99.8%, extra dry, over molecular sieve;

Toluene (PhMe): *Acros Organics,* 99.8% extra dry, over molecular sieve <0.005% H_2O;

Triethylamine (NEt_3): *Sigma Aldrich*, ≥99.5%;

1,4-Dioxane: *Acros Organics,* 99.5%, extra dry, over molecular sieve, stabilized;

Dimethylsulfoxide (DMSO): *Acros Organics,* ≥99.7%, extra dry, over molecular sieve, <0.005% H_2O;

Methanol (MeOH): *Acros Organics,* 99.8%, extra dry, over molecular sieve, <0.005% H_2O;

Reagents were available commercially (*BLD Pharm, Sigma-Aldrich, Acros Organics, TCI Europe, Alfa-Aesar*) and were used without further purification. Purity of raw products were given in mass percent. Cleaning solutions are saturated aqueous solutions of the salts.

Solvents, which were used for thin layer chromatography, flash chromatography, extraction and recrystallization [dichloromethane (CH_2Cl_2), diethylether (Et_2O), ethylacetate (EtOAc), *n*-hexane (*n*-Hex), methanol (MeOH), pentane (P)] were destilled before usage.

Ice/water (0 °C) or dry ice/acetone (–78 °C) were used as colling baths. Reactions at elevated temperatures were performed in silicone oil baths and the temperature regulated by a contact thermometer.

2. Analytical Methods

Irradiation Experiments: Irradiation Reactors

Photochemical reactions were carried out in flame-dried *Duran* tubes (diameter = 1 cm) in a positive geometry setup (cyclindrical array of 16 lamps, 350 nm: *Hitachi* UV-A, BI-B, λ_{max} = 350 nm; 366 nm: *Philips* BLB, λ_{max} = 365 nm; 420 nm: *Luzchem* LZC-UVA, λ_{max} = 420 nm) with the sample placed in the center of the illumination chamber.[1]

Thin Layer Chromatography (TLC)

TLC was performed on silica coated glass plates (*Merck*, silica 60 F254) with detection by UV-light (λ = 254 nm and λ = 366 nm) and/or by staining with a potassium permanganate solution [$KMnO_4$] (3.00 g potassium permanganate, 20.0 g, potassium carbonate, and 5.00 mL 5% aqueous sodium hydroxide solution in 300 mL deionized water) followed by heat treatment.

Flash Chromatography

Flash chromatography was performed using silica (230–400 mesh, ASTM) with particle size 40–63 µm (SI 60) by *Merck*. The amounts of silica and the column diameter were calculated according to the mass of the crude product and the separation difficulty. The crude product was solubilized in solvent and put directly onto the column, if possible. If the crude product was not soluble in the corresponding solvent, a different solvent was used and celite (*Celite*® 545, particle size 0.02–0.1 mm, pH 10, *Merck*) was added. After removal of the solvent under reduced pressure, the dry load was put directly onto the column.

Nuclear Magnetic Resonance Spectroscopy (NMR)

NMR spectra were recorded at room temperature either on a *Bruker* AVHD-400, AVHD-500 or AV-500 cryo. For NMR spectroscopical measurements the substance was solubilized in a deuterated solvent. The chemical shift was noted in δ [ppm] and referenced to the residual proton signals of the following solvents:

- chloroform-d_1 ($CDCl_3$): δ (^{1}H) = 7.26 ppm, δ (^{13}C) = 77.16 ppm;
- dimethylsufloxide-d_6 (DMSO-d_6): δ (^{1}H) = 2.50 ppm, δ (^{13}C) = 39.52 ppm;

The multiplicity within the ^{1}H-NMR-spectra were noted as follows: br (broad signal), s (singlet), d (doublet), t (triplet), q (quartet), p (pentet = quintet), sep (septet), m (multiplet) and *virt.* (virtual). In the case of coincidental coupling patterns of non-equivalent protons, the

coupling pattern was specified by rules of spectra of first order and chemical shifts of the virtual signal were given. The coupling constants *J* [Hz] were displayed as mean of the experimental found values. The assignment of the signal was done with heteronuclear singular quantum coherence (HSQC), heteronuclear multiple bond coherence (HMBC) and homonuclear correlation spectroscopy (COSY). The numbering of the carbon skeleton for assignment of NMR-signals was done according to international union of pure and applied chemistry (IUPAC)-nomenclature.

Infrared Spectroscopy (IR)

IR-spectra were measured directly in substance with a *JASCO* IR-4100 spectroscope by attenuated total reflection (ATR). The signal intensity or signal form was noted as follows: w (weak), m (medium), s (strong), br (broad), v (variable).

High Resolution Mass Spectroscopy (HRMS)

High Resolution Mass Spectra (HRMS) were measured on a Q Exactive Plus from *Thermo Fisher Scientific* (HRMS-ESI) with an orbitrap mass analyzer

UV/Vis Spectroscopy (UV/Vis)

UV/Vis was performed on a *Perkin Elmer* Lambda 35 UV/Vis spectrometer using a *Hellma* precision cell (quartz SUPRASIL®) with a pathway of 1 mm.

Melting point (M. p.)

The melting point of solids was determined using an apparatus by *Kofler* ("Thermopan", *Reichert*) and are not corrected.

Instrumentation (Transient Absorption Spectroscopy):

UV/vis absorption spectra were recorded on a LAMBDA 365 from Perkin Elmer.

For the laser flash photolysis (LFP) measurements, an LP980-KS apparatus form Edinburgh Instruments was used. The LFP setup was used with a thermoelectrically cooled 4-window cuvette holder (for 1 cm × 1 cm cuvettes) distributed by Edinburgh Instruments and all experiments were carried out at 20°C cuvette temperature. A frequency-tripled Nd:YAG laser from Quantel (Q smart 450) with ~5 ns pulses and a pulse frequency 10 Hz was used for excitation. The output energy was modified by the Q-switch delay and set to 5-30 mJ (exact details are provided in the corresponding figure captions). A beam expander (*Thorlabs*) was

used to ensure more homogenous excitation in the detection volume (beam diameter in front of the cuvette window, 0.8 cm extended from originally 0.4 cm). The Xe probe beam of the LP980 setup was unmodified. Kinetic traces were recorded at a single wavelength with a photomultiplier tube and for measuring transient absorption (TA) or (delayed) emission spectra an iCCD camera from Andor was employed. All TA spectra were time-integrated for 100 ns. The concentrations of the sensitizers were adjusted to ensure optical densities at the excitation wavelength of ≤0.2. Exact concentrations are given in the figure captions.

Taking the Beer-Lambert law for transient absorption spectroscopy (T indicates transient species, corresponding to the xanthone (XT) triplet in our case), the change in optical density ΔOD is given as:

$$\Delta OD\,(t,\lambda) \;=\; \varepsilon_T(\lambda)\,c_T(t)\,d$$

Hence, the maximum achievable transient absorption signal ΔOD directly depends on the pathlength *d*, over which the probe beam (always 1 cm, i.e. the cuvette pathlength) overlaps with the pump laser light being mandatory for producing transient species. The crucial overlap region is only 0.4 cm (corresponding to *d* in the equation above) with the unmodified laser beam, which is visualized in the following graph:

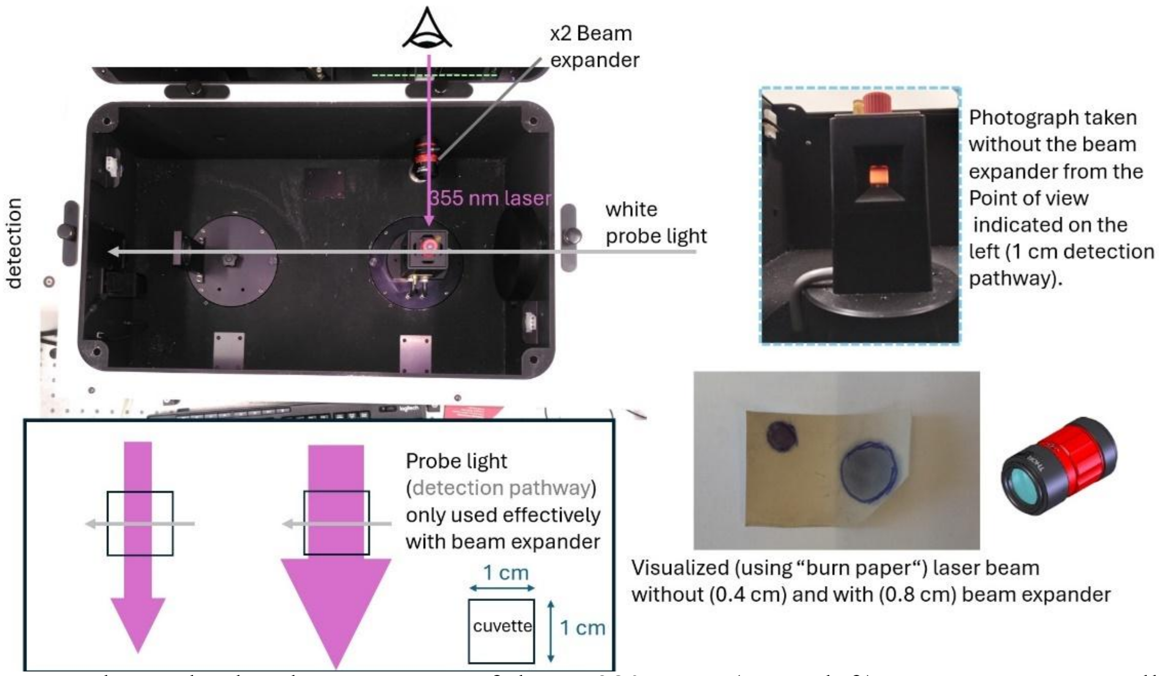

Beam and sample chamber geometry of the LP980 setup (upper left), temperature-controlled cuvette holder for 1×1 cm cuvettes with detection pathlength visualized through the emission of a dye (upper right), effect of the beam expander on the laser diameter (lower right), and improved overlap with the probe light enabled through the beam expander in the laser pump pathway (lower left, same color code as in the photograph directly above).

The incorporation of such a beam expander was initially introduced for a very similar transient absorption setup (LP920),[2] and it (i) improves the overlap between the pump and the probe beam yielding a more intense TA signal, (ii) makes the optical arrangement less prone to errors due to non-ideal beam alignment, and (iii) improves the photostability of a given photosensitizer as the probability of biphotonic side reactions, such as photoionization (which is known for xanthone),[3] is reduced because of the lower actual laser power density.

To determine quenching rate constants, Stern-Volmer analyses of the quenching experiments were performed. Kinetic decay traces were fitted using a mono exponential fitting function. The τ_0/τ values from LFP studies were plotted against the quencher concentration. A linear fit with the intersection fixed at 1 yielded the Stern-Volmer constant (K_{SV}) and division by the known τ_0 lifetime of the respective excited state under given conditions resulted in the bimolecular quenching rate constant.[4]

$$\frac{\tau_0}{\tau} = 1 + K_{SV}[\mathrm{Q}] = 1 + k_q \tau_0 [\mathrm{Q}]$$

Sample Preparation:

For the optical spectroscopy, dry acetonitrile (MeCN, *Thermo scientific*, Extra Dry, 99.9+%) was used. 2-Propanol (i-PrOH, technical grade) was further purified by distillation. Xanthone and **7a** were provided by the Bach group. Thioxanthone was used as obtained from the manufacturer (TXT, *BLDPharm*, 98 %). Before the measurement, all samples were purged with argon (Nippon Gases 5.0) for 10 minutes and sealed under inert gas in 1.0 cm pathlength septum cap cuvettes (quartz glass).

3. Emission and Characteristics of the Light Sources (350 nm, 366 nm, 420 nm and 455 nm)

The characteristics of the specific LEDs and lamps employed for photochemical reactions can be found in the following datasheets:

Datasheet FLT021 LZC-UVA

Basic Information

Type	Fluorescent light tube
Description	Luzchem LZC-UVA
Manufacturer / Supplier	Hitachi / Luzchem
Order number / Date of purch.	LZC-UVA / 09/2015
Internal lot / serial number	2015-09 / FLT021

Specification Manufacturer

Type / size	T5 tube, G5 socket
Mechanical specification	16 mm diameter, 288 mm length
Electrical specification	8 W
Wavelength (range, typ.)	300 - 400 nm, 350m nm, UV-A
Spectral width (FWHM)	~ 40 nm
Datasheet	

Characterization

Description of measurement	Measured with Ocean-optics USB4000 spectrometer using a calibrated setup (cosine corrector/fibre). The cosine corrector was placed at 20 mm distance from a single fluorescent tube at half height.	
Measured dominant wavelength / Int.	350 nm	115 µW/mm²nm
Measured spectral width (FWHM)	40 nm	
Integral Reference intensity / range	5017 µW/cm²	300-425 nm

Spectrum

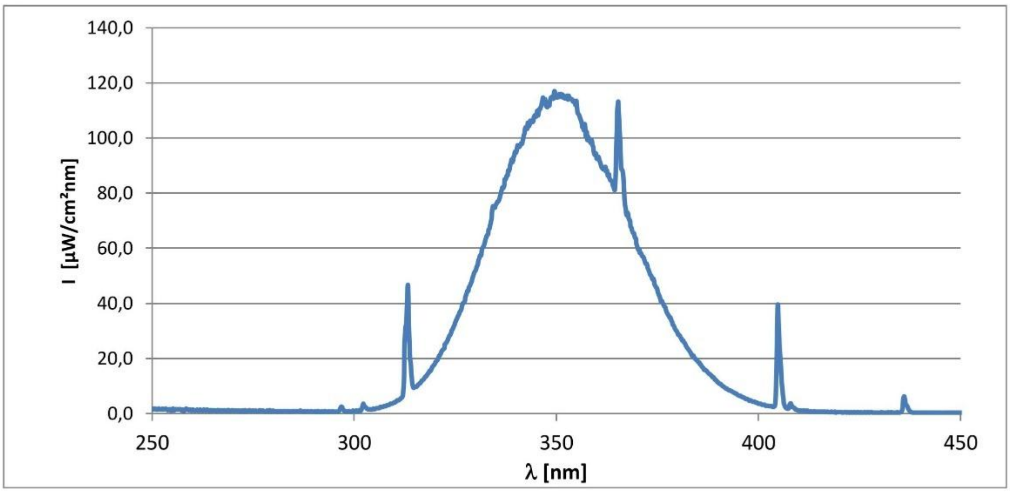

Datasheet FLT031_365 Philipps-BLB-365

Basic Information

Type	Fluorescent light tube
Description	Philipps TL 8W BLB
Manufacturer / Supplier	Philipps / multi-lite
Order number / Date of purch.	n/a / 02/2024
Internal lot / serial number	2024-02 / FLT030

Specification Manufacturer

Type / size	T5 tube, G5 socket
Mechanical specification	16 mm diameter, 288 mm length
Electrical specification	8 W
Wavelength (range, typ.)	350 - 400 nm
Spectral width (FWHM)	~ 16 nm
Datasheet Measurement	13.02.2024

Characterization

Description of measurement	Measured with Ocean-optics USB4000 spectrometer using a calibrated setup (cosine corrector/fibre). The cosine corrector was placed at 20 mm distance from a single fluorescent tube at half height.	
Measured dominant wavelength / Int.	365 nm	183 µW/mm²nm
Measured spectral width (FWHM)	17 nm	
Integral Reference intensity / range	3594 µW/cm²	300-450 nm

Spectrum

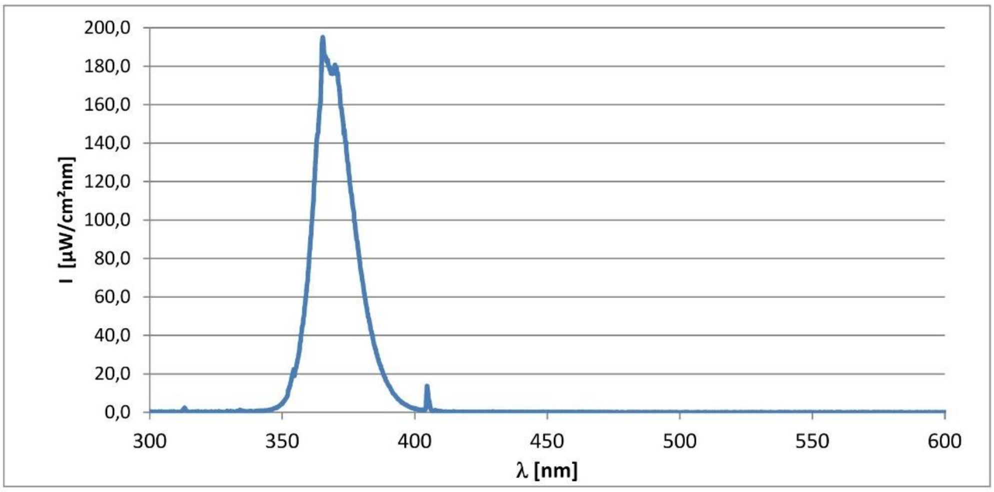

Datasheet FLT022 LZC-420

Basic Information

Type	Fluorescent light tube
Description	Luzchem LZC-420
Manufacturer / Supplier	n/a / Luzchem
Order number / Date of purch.	n/a / 07/2017
Internal lot / serial number	2017-07 / FLT022

Specification Manufacturer

Type / size	T5 tube, G5 socket
Mechanical specification	16 mm diameter, 288 mm length
Electrical specification	8 W
Wavelength (range, typ.)	400 - 440 nm
Spectral width (FWHM)	~ 30 nm
Datasheet	LES-420-016

Characterization

Description of measurement	Measured with Ocean-optics USB4000 spectrometer using a calibrated setup (cosine corrector/fibre). The cosine corrector was placed at 20 mm distance from a single fluorescent tube at half height.	
Measured dominant wavelength / Int.	421 nm	121 µW/mm²nm
Measured spectral width (FWHM)	30 nm	
Integral Reference intensity / range	4142 µW/cm²	350-500 nm

Spectrum

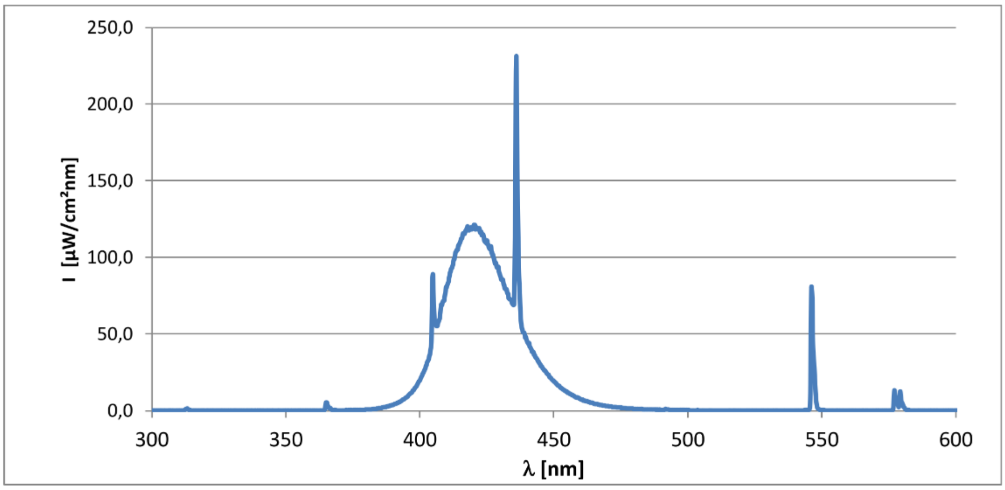

Datasheet pLED002 Av-455-3W

Basic Information

Type	High Power LED
Description	Avonec 455nm - 460nm, 3W
Manufacturer / Supplier	Avonec / Avonec
Order number / Date of purch.	3W455460m / n/a
Internal lot / serial number	n/a / pLED002

Specification Manufacturer

Type / size	Single emitter / 8.05 mm diameter
Mechanical specification	
Electrical specification	max. 750 mA, 3.5-4.5 V
Wavelength (range, typ.)	455-460 nm
Spectral width (FWHM)	n/a
Datasheet	avonec.de

Characterization

Description of measurement	Measured with Ocean-optics USB4000 spectrometer using a calibrated setup (cosine corrector/fibre). The distance between the emitting surface and the surface of the cosine corrector was 20 mm. The LED was operated at 700 mA on a passive heat-sink at approx. 20 °C	
Measured dominant wavelength / Int.	455 nm	2016 µW/mm²nm
Measured spectral width (FWHM)	21 nm	
Integral Reference intensity / range	50925 µW/cm²	400-525 nm

Spectrum

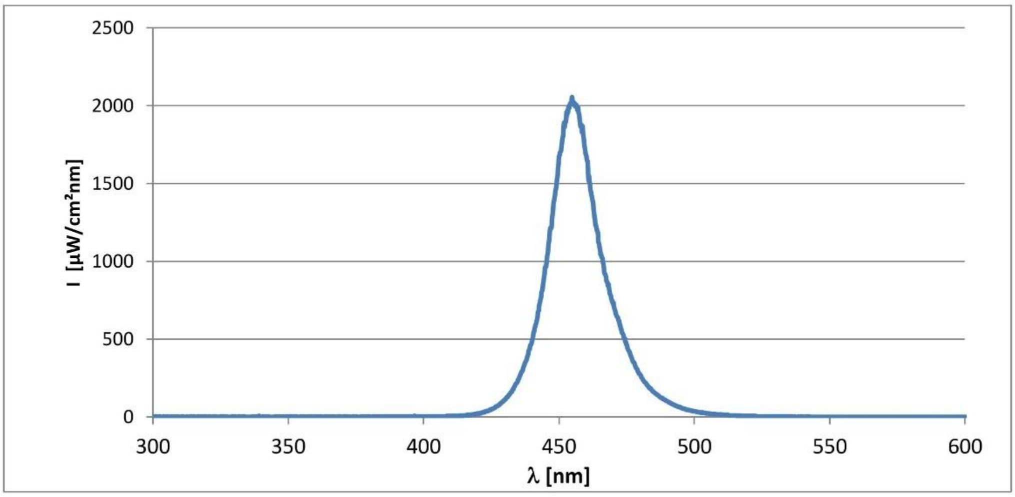

5. Reaction Condition Screening

Table 1: Conditions for the optimization of solvent, catalyst, catalyst-loading, concentration, temperature and wavelength.

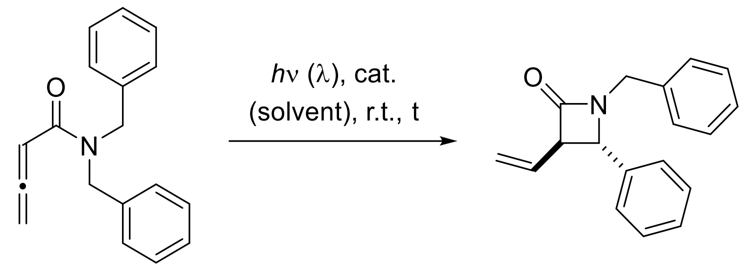

#	Cat.	λ [nm]	Solvent	c [mM]	time [h]	yield [%]	comment
1	TXT (20 mol %)	420	CH_2Cl_2	20	24	-	sm
2	2,2'-Br,Br-TXT (20 mol%)	420	CH_2Cl_2	20	24	-	sm
3	Ir(dFCF3ppy)2(dtbbpy)PF6 (3 mol%)	420	CH_2Cl_2	20	24	19	no full conversion
4	$Ir(ppy)_3$ (3 mol%)	420	CH_2Cl_2	20	3	-	sm
5	TXT (20 mol %)	390 (Kessil, 120 W)	CH_2Cl_2	20	3	-	sm, degradation
6	$Ir(dFCF_3ppy)_2(dtbbpy)PF_6$ (3 mol%)	455	CH_2Cl_2	20	4	-	sm
7	$Ir(dFCF_3ppy)_2(dtbbpy)PF_6$ (3 mol%)	455	MeCN	20	4	-	sm
8	$Ir(dFCF_3ppy)_2(dtbbpy)PF_6$ (3 mol%)	455	TFE/$CHCl_3$ (1:1)	20	4	-	sm
9	$Ir(dFCF_3ppy)_2(dtbbpy)PF_6$ (3 mol%)	455	PhMe	20	4	-	sm
10	XT (20 mol%)	366	CH_2Cl_2	20	5	50	full conversion
11	Benzophenone (20 mol%)	366	CH_2Cl_2	20	24	-	sm, degradation
12	XT (20 mol%)	366	MeCN	20	7.5	63	full conversion

13	XT (20 mol%)	366	TFT	20	16	62	full conversion
14	XT (20 mol%)	366	PhH	20	18	55	full conversion
15	Anthraquinone (20 mol%)	366	MeCN	20	15.5	0	sm, degradation
16	4CzIPN (3 mol%)	420	MeCN	20	15.5	0	sm, degradation
17	XT (20 mol%) + Thiophenol (50 mol%)	366	MeCN	20	16.5	15	no full conversion,degradation
18[1]	XT (20 mol%)	366	MeCN	20	24	64	full conversion
19	TXT (20 mol%)	420	MeCN	20	24	27	no full conversion, degradation
20	Acetone	350	acetone	10	23	51	full conversion
21	Acetophenone (25 mol%)	350	MeCN	10	4.5	41	full conversion
22	XT (10 mol%) + Naphthalene (20 mol%)	350	MeCN	10	18	56	full conversion
23	3,3‘-Ome-TXT (10 mol%)	366	MeCN	20	20	65	full conversion, catalyst not separable
24	XT (10 mol%)	366	MeCN	10	17	71	full conversion
25	XT (10 mol%)	350	MeCN	20	1.5	73	full conversion
26	XT (10 mol%)	350	MeCN	10	2.5	63	full conversion
27	XT (15 mol%)	350	MeCN	20	2	72	full conversion
28	XT (10 mol%)	350	MeCN	20	2	64	full conversion, *Freeze-Pump-Thaw* MeCN
29	3,3‘-TXT (10 mol%)	350	MeCN	20	3	64	full conversion, catalyst not separable
30	-	350	MeCN	20	24	0	sm, no reaction

[1] reaction performed at -10°C

6. UV/Vis-Spectra

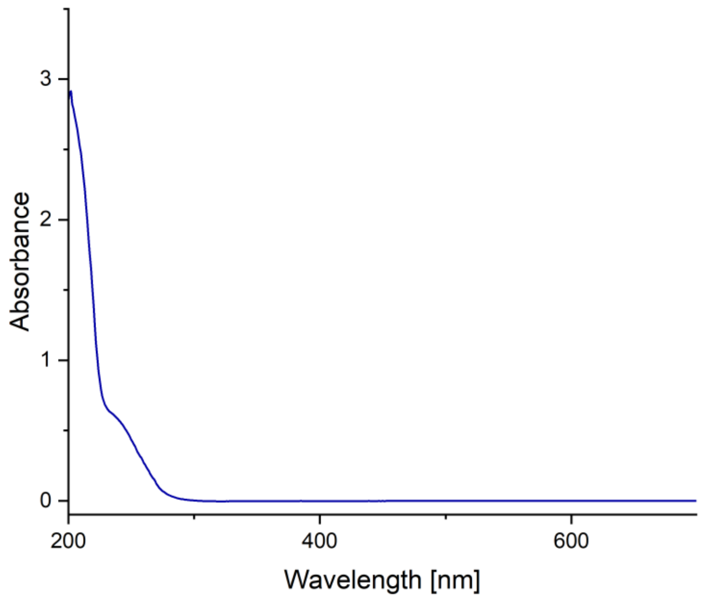

UV/Vis-spectrum of **7a** in MeCN (c = 1 mM) [measured in 1.0 mm quartz cuvette].

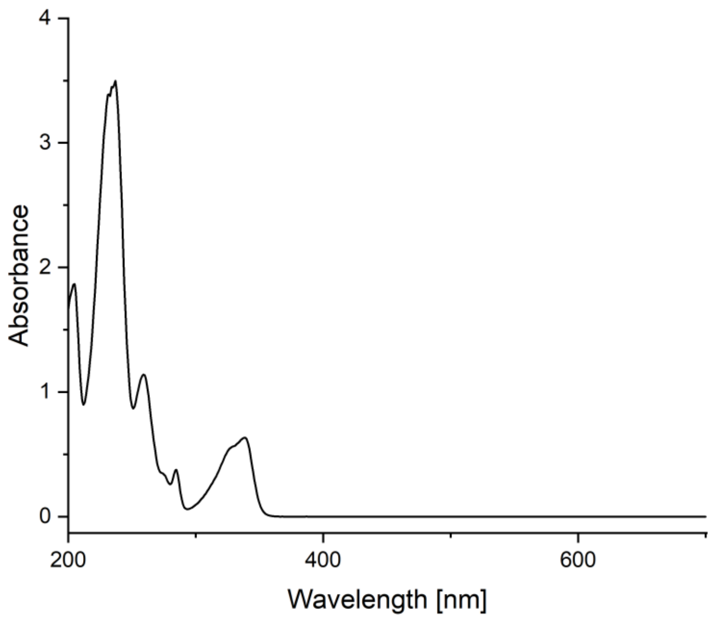

UV/Vis-spectrum of xanthone in MeCN (c = 1 mM) [measured in 1.0 mm quartz cuvette].

7. Experimental Procedures and Characterization

3,3’-Dimethoxythioxanthone was obtained following a multi-step procedure by L. D. Elliott et al.[5]

Bis(4-fluorobenzyl)amine was obtained following a procedure by R. Kumar et al.[6]

Bis(3,5-dimethylbenzyl)amine was obtained following a procedure by M. Horn et al.[7]

N-Methyl-1-phenylmethan-*d*-amine was obtained following a procedure by W. Pan et al.[8] from methylamine and benzaldehyde-α-d_1.

N-Methyl-1-phenylmethan-d_2-amine was obtained following a procedure by C. E. Hendrick et al.[9]

N-Methyl-1-(naphthalen-2-yl)methanamine was obtained following a procedure by C.-H. Ou Yang et al.[10]

5,5-Dimethylhexa-2,3-dienoic acid was obtained following a multistep procedure by Y. Deng et al.[11]

7.1 Synthesis of the Photosubstrates

General procedure for the two step synthesis of allene amides from 3-butynoic acid and secondary amines (GP-1)[12]

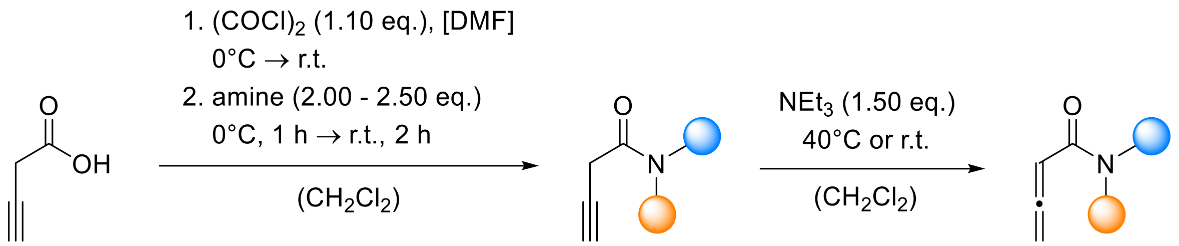

To a solution of 3-butynoic acid (1.00 g, 11.9 mmol, 1.00 eq.) in CH_2Cl_2 (30 mL) and DMF (100 µL) at 0°C was added oxalyl chloride (1.12 mL, 13.1 mmol, 1.10 eq.) dropwise. After complete addition the reaction solution was allowed to reach room temperature and was stirred for one to two hours until no gas formation was observed. The formed HCl gas was removed by bubbling argon gas through the stirring solution for 45 minutes. Then the solution was cooled to 0°C and the respective amine (2.00 – 2.50 eq.) was added slowly to the solution. After the addition the reaction mixture was stirred for one hour at 0°C and then for additional two hours at room temperature. The reaction was quenched by addition of aq. HCl-solution (1 M, 50 mL). After separation of the phases the organic layer was washed again with aq. HCl-solution (1 M, 50 mL). The organic layer was dried over Na_2SO_4, filtered and the solvent removed under reduced pressure. After chromatography on a short column (short path) the respective crude but-3-ynamide was used without further purification in the isomerization reaction.

To a solution of but-3-ynamide (1.00 eq.) in CH_2Cl_2 [100 mM] NEt_3 (1.50 eq.) were added. The reaction mixture was stirred at 40°C or room temperature until full conversion of the but-3-ynamide was observed by TLC reaction control. After completion the reaction mixture was washed with aq. HCl (1 M, 2 × 50 mL), the organic layer dried over Na_2SO_4, filtered and the solvent removed under reduced pressure. The respective buta-2,3-dienamides were obtained after column chromatography.

General procedure for the two step synthesis of allene amides from 3-butynoic acid and secondary amines (GP-2)[12]

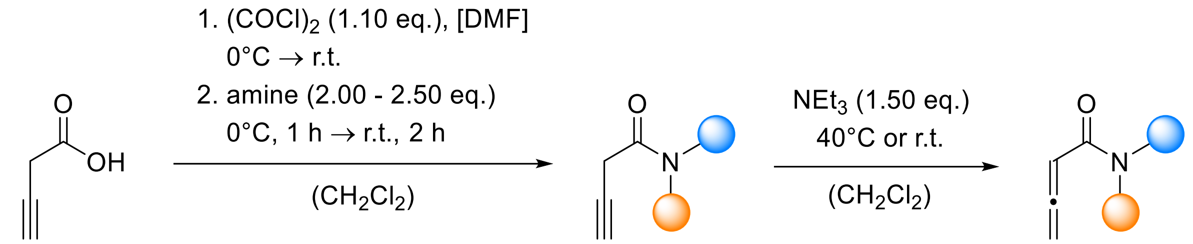

To a solution of 3-butynoic acid (1.00 g, 11.9 mmol, 1.00 eq.) in CH_2Cl_2 (30 mL) and DMF (100 µL) at 0°C was added oxalyl chloride (1.12 mL, 13.1 mmol, 1.10 eq.) dropwise. After complete addition the reaction solution was allowed to reach room temperature and was stirred for one to two hours until no gas formation was observed. The solvent and HCl were removed under reduced pressure at 0°C. After addition of CH_2Cl_2 (30 mL) the solution was cooled to 0°C and the respective amine (2.00 – 2.50 eq.) was added slowly to the solution. After the addition the reaction mixture was stirred for one hour at 0°C and then for additional two hours at room temperature. The reaction was quenched by addition of aq. HCl-solution (1 M, 50 mL). After separation of the phases the organic layer was washed again with aq. HCl-solution (1 M, 50 mL). The organic layer was dried over Na_2SO_4, filtered and the solvent removed under reduced pressure. After chromatography on a short column (short path) the respective crude but-3-ynamide was used without further purification in the isomerization reaction.

To a solution of but-3-ynamide (1.00 eq.) in CH_2Cl_2 [100 mM] NEt_3 (1.50 eq.) were added. The reaction mixture was stirred at 40°C or room temperature until full conversion of the but-3-ynamide was observed by TLC reaction control. After completion the reaction mixture was washed with aq. HCl (1 M, 2 × 50 mL), the organic layer dried over Na_2SO_4, filtered and the solvent removed under reduced pressure. The respective buta-2,3-dienamides were obtained after column chromatography.

N,N-Dibenzylbuta-2,3-dienamide (7a)

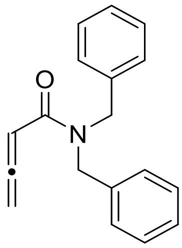

$C_{18}H_{17}NO$
M = 263,34 g/mol

Following **GP-1**, dibenzylamine (5.72 mL, 29.7 mmol, 2.50 eq.) was coupled with but-3-ynoic acid chloride to but-3-ynamide. After short path chromatography (SiO_2, 3 × 20 cm, EtOAc/Hex = 1/10) crude but-3-ynamide (1.23 g, 4.67 mmol, 41%) was obtained as orange oil and used without further purification. Following **GP-1**, but-3-ynamide was isomerized to buta-2,3-dienamide **7a** at room temperature for 24 hours. After column chromatography (SiO_2, 3 × 20 cm, P/Et_2O = 7/3) buta-2,3-dienamide **7a** (627 mg, 2.38 mmol, 51%) was obtained as a white solid.

M.p.: 64 °C.

TLC: R_f = 0.22 (P/Et_2O = 6/4) [UV, $KMnO_4$].

^{1}H-NMR (500 MHz, DMSO-d^6, 298 K): δ [ppm] = 4.51 (s, 2H, N-CH_2-Ph), 4.61 (s, 2H, N-CH_2-Ph), 5.32 (d, ^4J = 6.5 Hz, 2H, C-4-HH), 6.37 (t, ^4J = 6.5 Hz, 1H, C-2-H), 7.18–7.40 (m, 10H, H_{Ar}).

^{13}C-NMR (101 MHz, DMSO-d^6, 298 K): δ [ppm] = 48.5 (t, N-CH_2-Ph), 50.4 (t, N-CH_2-Ph), 79.1 (t, C-4), 87.1 (d, C-2), 126.7 (d, 2C, 2 × C_{ortho}), 127.1 (d, C_{para}), 127.3 (d, C_{para}), 127.7 (d, 2C, 2 × C_{ortho}), 128.4 (d, 2C, 2 × C_{meta}), 128.6 (d, 2C, 2 × C_{meta}), 137.4 (s, C_{ipso}), 137.5 (s, C_{ipso}), 164.4 (s, C-1), 214.4 (s, C-3).

IR (ATR): $\tilde{\nu}$ [cm^{-1}] = 3055 (w), 3028 (w), 2977 (w), 2948 (w), 1972 (w), 1939 (m, C=C=C), 1735 (s, C=O), 1627 (m), 1354 (m), 742 (w).

HRMS (ESI): [$M+H^+$]: calc.: 264.1383; found: 264.1381.

***N,N*-Bis(3-chlorobenzyl)buta-2,3-dienamide (7b)**

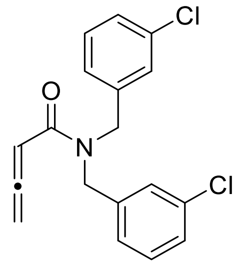

$C_{18}H_{15}Cl_2NO$
M = 332,22 g/mol

Following **GP-1** (but-3-ynoic acid: 458 mg, 5.45 mmol), bis(3-chlorobenzyl)amine (2.90 g, 10.9 mmol, 2.00 eq.) was coupled with but-3-ynoic acid chloride to but-3-ynamide. After short path chromatography (SiO_2, 3 × 20 cm, P/Et_2O = 1/1) crude but-3-ynamide (959 mg, 2.89 mmol, 53%) was obtained as yellow oil and used without further purification. Following **GP-1**, but-3-ynamide was isomerized to buta-2,3-dienamide **7b** at room temperature for five hours. After column chromatography (SiO_2, 3 × 25 cm, EtOAc/Hex = 1/10) buta-2,3-dienamide **7b** (739 mg, 2.22 mmol, 75%) was obtained as a white solid.

M.p.: 53°C.

TLC: R_f = 0.17 (EtOAc/Hex = 2/8) [UV, $KMnO_4$].

^{1}H-NMR (400 MHz, DMSO-d_6, 298 K): δ [ppm] = 4.55 (s, 2H, N-CH_2-Ar), 4.70 (s, 2H, N-CH_2-Ar), 5.33 (d, ^4J = 6.5 Hz, 2H, C-4-H_2), 6.36 (t, ^4J = 6.5 Hz, 1H, C-2-H), 7.14–7.43 (m, 8H, H_{Ar}).

^{13}C-NMR (101 MHz, DMSO-d_6, 300 K): δ [ppm] = 48.7 (t, N-*C*H_2-Ar), 50.4 (t, N-*C*H_2-Ar), 79.2 (t, C-4), 86.9 (d, C-2), 125.3 (d, C_{Ar}), 126.4 (d, C_{Ar}), 126.6 (d, C_{Ar}), 127.1 (d, C_{Ar}), 127.2 (d, C_{Ar}), 127.5 (d, C_{Ar}), 126.4 (d, C_{Ar}), 130.2 (d, C_{Ar}), 130.4 (d, C_{Ar}), 133.0 (s, C-Cl), 133.3 (s, C-Cl), 140.1 (s, 2C, 2 × C_{ipso}), 164.6 (s, C-1), 214.5 (s, C-3).

IR (ATR): $\tilde{\nu}$ [cm^{-1}] = 3035 (w), 2987 (w), 1965 (m, C=C=C), 1932 (m, C=C=C), 1619 (s, C=O), 1476 (s), 1454 (s), 1407 (s), 1213 (s), 1032 (s), 858 (s), 777 (s).

HRMS (ESI): [M+H^+]: calc.: 332.0603; found: 332.0602.

N,N-Bis(4-fluorobenzyl)buta-2,3-dienamide (7c)

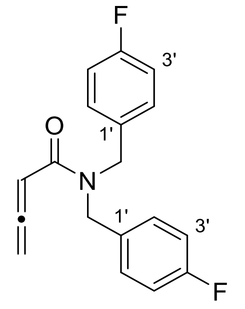

$C_{18}H_{15}F_2NO$
M = 299,32 g/mol

Following **GP-2**, bis(4-fluorobenzyl)amine (6.70 g, 28.7 mmol, 2.20 eq.) was coupled with but-3-ynoic acid chloride to but-3-ynamide. After short path chromatography (SiO_2, 3 × 20 cm, P/Et_2O = 8/2) crude but-3-ynamide (2.81 g, 9.39 mmol, 79%) was obtained as colorless oil and used without further purification. Following **GP-1**, but-3-ynamide was isomerized to buta-2,3-dienamide **7c** at 40°C for seven hours. After column chromatography (SiO_2, 3 × 20 cm, P/Et_2O = 9/1 → 7/3) buta-2,3-dienamide **7c** (1.10 g, 3.68 mmol, 39%) was obtained as a colorless oil.

TLC: R_f = 0.22 (P/Et_2O = 6/4) [UV, $KMnO_4$].

^{1}H-NMR (400 MHz, DMSO-d_6, 298 K): δ [ppm] = 4.48 (s, 2H, N-CH_2-Ar), 4.61 (s, 2H, N-CH_2-Ar), 5.32 (d, ^4J = 6.5 Hz, 2H, C-4-H_2), 6.37 (t, ^4J = 6.5 Hz, 1H, C-2-H), 7.10–7.31 (m, 8H, H_{Ar}).

^{13}C-NMR (101 MHz, DMSO-d_6, 298 K): δ [ppm] = 47.9 (t, N-*C*H_2-Ar), 49.8 (t, N-*C*H_2-Ar), 79.2 (t, C-4), 87.0 (d, C-2), 115.1 (d, $^2J_{CF}$ = 23.0 Hz, 2C, 2 × C-3'), 115.4 (d, $^2J_{CF}$ = 22.6 Hz, 2C, 2 × C-3'), 128.8 (d, $^3J_{CF}$ = 8.3 Hz, 2C, 2 × C-2'), 129.8 (d, $^3J_{CF}$ = 8.5 Hz, 2C, 2 × C-2'), 133.5 (s, C-1'), 133.7 (s, C-1'), 161.4 (d, $^1J_{CF}$ = 243.2 Hz, 2C, 2 × C-4'), 164.4 (s, C-1).

^{19}F-NMR (376 MHz, DMSO-d_6, 298 K): δ [ppm] = -117.5– -116.9 (m, 2F).

IR (ATR): $\tilde{\nu}$ [cm^{-1}] = 3068 (w), 1969 (m, C=C=C), 1944 (m, C=C=C), 1722 (w), 1630 (s), 1603 (m), 1508 (s), 1444 (m), 1407 (s), 1219 (s), 1155 (s), 1096 (m), 1015 (m), 925 (m), 818 (s), 753 (m).

HRMS (ESI): [M+H$^+$]: calc.: 300.1194; found: 300.1193.

***N,N*-Bis(3,5-dimethylbenzyl)buta-2,3-dienamide (7d)**

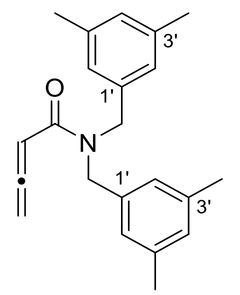

$C_{22}H_{25}NO$
M = 319,45 g/mol

Following **GP-2**, bis(3,5-dimethylbenzyl)amine (7.03 g, 27.8 mmol, 2.33 eq.) was coupled with but-3-ynoic acid chloride to but-3-ynamide. After short path chromatography (SiO_2, 3 × 20 cm, P/Et_2O = 10/1 → 8/2) crude but-3-ynamide (2.25 g, 7.05 mmol, 59%) was obtained as colorless oil and used without further purification. Following **GP-1**, but-3-ynamide was isomerized to buta-2,3-dienamide **7d** at 40°C for seven hours. After column chromatography (SiO_2, 3 × 20 cm, P/Et_2O = 8/2 → 7/3) buta-2,3-dienamide **7d** (1.09 g, 3.41 mmol, 48%) was obtained as a white solid.

M.p.: 76°C.

TLC: R_f = 0.30 (P/Et_2O = 2/1) [UV, $KMnO_4$].

^{1}H-NMR (400 MHz, DMSO-d_6, 298 K): δ [ppm] = 2.24 (s, 6H, 2 × C-3'-CH_3), 2.24 (s, 6H, 2 × C-3'-CH_3), 4.43 (s, 2H, N-CH_2-Ar), 4.50 (s, 2H, N-CH_2-Ar), 5.32 (d, ^4J = 6.5 Hz, 2H, C-4-H_2), 6.32 (t, ^4J = 6.5 Hz, 1H, C-2-H), 6.77 (s, 2H, 2 × C-2'-H), 6.81 (s, 2H, 2 × C-2'-H), 6.89 (s, 1H, C-4'-H), 6.91 (s, 1H, C-4'-H).

^{13}C-NMR (101 MHz, DMSO-d_6, 298 K): δ [ppm] = 20.9 (q, 4C, 4 × C-3'-CH$_3$), 48.4 (t, N-CH_2-Ar), 50.2 (t, N-CH_2-Ar), 79.1 (t, C-4), 87.2 (d, C-2), 124.4 (d, 2C, 2 × C-2'), 125.5 (d, 2C, 2 × C-2'), 128.5 (d, C-4'), 128.7 (d, C-4'), 137.4 (s, 3C, C-1', 2 × C-2'), 137.7 (s, 3C, C-1', 2 × C-2'), 164.3 (C-1), 214.5 (s, C-3).

IR (ATR): $\tilde{\nu}$ [cm^{-1}] = 3057 (w), 3006 (w), 2971 (w), 2919 (w), 2860 (w), 1951 (m, C=C=C), 1778 (w), 1714 (w), 1631 (s), 1601 (m), 1461 (m), 1447 (m), 1259 (w), 1195 (s), 1026 (m), 913 (w), 859 (m), 847 (s), 763 (m), 723 (s).

HRMS (ESI): [M+H$^+$]: calc.: 320.2009; found: 320.2007.

***N,N*-Bis(4-methoxybenzyl)buta-2,3-dienamide (7e)**

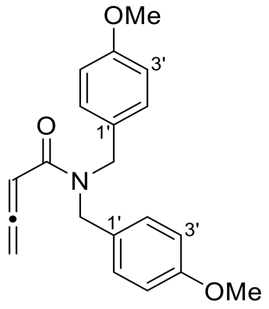

$C_{20}H_{21}NO_3$
M = 323,39 g/mol

Following **GP-1** [but-3-ynoic acid (700 mg, 8.32 mmol, 1.00 eq.)], bis(4-methoxybenzyl)amine (5.36 g, 20.8 mmol, 2.50 eq.) was coupled with but-3-ynoic acid chloride to but-3-ynamide. After short path chromatography (SiO_2, 4 × 20 cm, P/Et_2O = 1/1) crude but-3-ynamide (1.48 g, 4.58 mmol, 55%) was obtained as yellow oil and used without further purification. Following **GP-1**, but-3-ynamide was isomerized to buta-2,3-dienamide **7e** at room temperature for 43 hours. After column chromatography (SiO_2, 3 × 20 cm, P/Et_2O = 6/4) buta-2,3-dienamide **7e** (714 mg, 2.21 mmol, 48%) was obtained as a yellow oil.

TLC: R_f = 0.13 (P/Et_2O = 1/1) [UV, $KMnO_4$].

^{1}H-NMR (500 MHz, DMSO-d_6, 298 K): δ [ppm] = 3.73 (s, 3H, C-4'-OCH_3), 3.74 (s, 3H, C-4'-OCH_3), 4.40 (s, 2H, N-CH_2-Ar), 4.48 (s, 2H, N-CH_2-Ar), 5.32 (d, ^4J = 6.5 Hz, 2H, C-4-H_2), 6.38 (t, ^4J = 6.5 Hz, 1H, C-2-H), 6.89 (*virt.* d, J = 8.3 Hz, 2H, 2 × C-3'-H), 6.93 (*virt.* d, J = 8.3 Hz, 2H, 2 × C-3'-H), 7.12 (*virt.* d, J = 8.3 Hz, 2H, 2 × C-2'-H), 7.16 (*virt.* d, J = 8.3 Hz, 2H, 2 × C-2'-H).

^{13}C-NMR (126 MHz, DMSO-d_6, 298 K): δ [ppm] = 47.5 (t, N-*C*H_2-Ar), 49.4 (t, N-*C*H_2-Ar), 55.11 (q, C-4'-O*C*H_3), 55.14 (q, C-4'-O*C*H_3), 79.2 (t, C-4), 87.3 (d, C-2), 113.9 (d, 2C, 2 × C-3'), 114.1 (d, 2C, 2 × C-3'), 128.1 (d, 2C, 2 × C-2'), 129.2 (s, C-1'), 129.3 (d, 2C, 2 × C-2'), 129.5 (s, C-1'), 158.5 (s, C-4'), 158.6 (s, C-4'), 164.3 (s, C-1), 214.6 (s, C-3).

IR (ATR): $\tilde{\nu}$ [cm^{-1}] = 2996 (w), 2956 (w), 2933 (w), 2836 (w), 1968 (m, C=C=C), 1944 (m, C=C=C), 1629 (s, C=O), 1610 (s, C=O), 1510 (s), 1243 (s), 1173 (s), 1029 (s), 814 (s).

HRMS (ESI): [M+H$^+$]: calc.: 324.1594; found: 324.1580.

N-Benzyl-*N*-methylbuta-2,3-dienamide (7f)

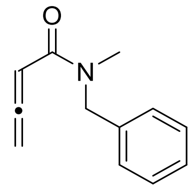

$C_{12}H_{13}NO$
M = 187,24 g/mol

Following **GP-2**, *N*-benzyl-*N*-methylamine (3.84 mL, 29.7 mmol, 2.5 eq.) was coupled with but-3-ynoic acid chloride to but-3-ynamide. After short path chromatography (SiO_2, 3 × 20 cm, P/Et_2O = 6/4) crude but-3-ynamide (1.30 g, 6.93 mmol, 58%) was obtained as yellow oil and used without further purification. Following **GP-1**, but-3-ynamide was isomerized to buta-2,3-dienamide **7f** at 40°C for seven hours. After column chromatography (SiO_2, 3 × 20 cm, P/Et_2O = 8/2 → 1/1) buta-2,3-dienamide **7f** (853 mg, 4.56 mmol, 66%) was obtained as a colorless oil.

TLC: R_f = 0.49 (Et_2O) [UV, $KMnO_4$].

Rotameric ratio: R1/R2 = 45/55.

^{1}H-NMR (400 MHz, DMSO-d_6, 298 K): δ [ppm] = 2.83 (s, 1.32H, N-CH_3, R1), 2.98 (s, 1.68H, N-CH_3, R2), 4.53 (s, 1.12H, N-CH_2-Ph, R2), 4.66 (s, 0.88H, N-CH_2-Ph, R1), 5.27 (d, ^4J = 6.5 Hz, 0.9H, C-4-H_2, R1), 5.31 (d, ^4J = 6.5 Hz, 1.1H, C-4-H_2, R2), 6.34 (t, ^4J = 6.5 Hz, 0.55H, C-2-H, R2), 6.39 (t, ^4J = 6.5 Hz, 0.45H, C-2-H, R1), 7.21 (t, ^3J = 8.2 Hz, 2H, 2 × C_{ortho}-H), 7.24–7.31 (m, 1H, C_{para}-H), 7.32–7.40 (m, 2H, 2 × C_{meta}-H).

^{13}C-NMR (101 MHz, DMSO-d_6, 298 K): δ [ppm] = 33.8 (q, 0.45C, N-CH_3, R1), 35.4 (q, 0.55C, N-CH_3, R2), 50.3 (t, 1.1C, N-CH_2-Ph, R2), 52.9 (t, 0.9C, N-CH_2-Ph, R1), 78.9 (t, 1.1C, C-4, R2), 79.0 (t, 0.9C, C-4, R1), 87.1 (d, 0.45C, C-2, R1), 87.2 (d, 0.55C, C-2, R2), 126.7 (d, 0.9C, 2 × C_{ortho}, R1), 127.2 (d, 1.1C, C_{para}, R2), 127.6 (d, 0.9C, C_{para}, R1), 127.6 (d, 1.1C, 2 × C_{ortho}, R2), 128.6 (d, 1.1C, 2 × C_{meta}, R2), 128.7 (d, 0.9C, 2 × C_{meta}, R1), 137.60 (s, 0.55C, C_{ipso}, R2), 137.62 (s, 0.45C, C_{ipso}, R1), 164.1 (s, 0.55C, C-1, R2), 164.2 (s, 0.45C, C-1, R1), 213.9 (s, 0.55C, C-3, R2), 214.1 (s, 0.45C, C-3, R1).

IR (ATR): $\tilde{\nu}$ [cm^{-1}] = 3062 (w), 3031 (w), 2986 (w), 2930 (w), 1968 (m, C=C=C), 1945 (m, C=C=C), 1615 (s), 1494 (m), 1480 (m), 1398 (s), 1078 (m), 851 (s), 734 (s), 698 (s).

HRMS (ESI): [M+H$^+$]: calc.: 188.1070; found: 188.1069.

N-Benzyl-N-methylbuta-2,3-dienamide-d_1 (7f-d_1)

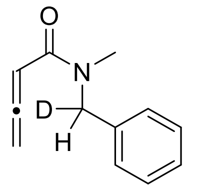

$C_{11}H_{12}DNO$
M = 176,24 g/mol

Following **GP-1**, *N*-methyl-1-phenylmethan-*d*-amine (3.31 g, 27.1 mmol, 2.28 eq., 95 %D) was coupled with but-3-ynoic acid chloride to but-3-ynamid. After short path chromatography (SiO_2, 3 × 20 cm, P/Et_2O = 6/4) crude but-3-ynamide (745 mg, 3.96 mmol, 33%) was obtained as yellow oil and used without further purification. Following **GP-1**, but-3-ynamide was isomerized to buta-2,3-dienamide **7f-d_1** at room temperature for 27 hours. After column chromatography (SiO_2, 3 × 20 cm, P/Et_2O = 8/2 → 1/1) buta-2,3-dienamide **7f-d_1** (539 mg, 2.86 mmol, 72%, 94 %D) was obtained as a colorless oil.

TLC: R_f = 0.49 (Et_2O) [UV, $KMnO_4$].

Rotameric ratio: R1/R2 = 45/55.

^{1}H-NMR (500 MHz, DMSO-d_6, 298 K): δ [ppm] = 2.82 (s, 1.32H, N-CH_3, R1), 2.97 (s, 1.68H, N-CH_3, R2), 4.51 (s, 0.55H, N-C*H*D-Ph, R2), 4.64 (s, 0.45H, N-CH_2-Ph, R1), 5.28 (d, ^4J = 6.5 Hz, 0.9H, C-4-H_2, R1), 5.31 (d, ^4J = 6.6 Hz, 1.1H, C-4-H_2, R2), 6.34 (t, ^4J = 6.6 Hz, 0.55H, C-2-H, R2), 6.39 (t, ^4J = 6.5 Hz, 0.45H, C-2-H, R1), 7.18–7.40 (m, 5H, 2 × C_{ortho}-H, 2 × C_{meta}-H, C_{para}-H).

^{13}C-NMR (126 MHz, DMSO-d_6, 298 K): δ [ppm] = 33.8 (q, 0.45C, N-CH_3, R1), 35.4 (q, 0.55C, N-CH_3, R2), 50.0 (t, $^1J_{CD}$ = 21.0 Hz, 1.1C, N-CHD-Ph, R2), 52.6 (t, $^1J_{CD}$ = 21.4 Hz, 0.9C, N-CHD-Ph, R1), 78.9 (t, 1.1C, C-4, R2), 79.0 (t, 0.9C, C-4, R1), 87.1 (d, 0.45C, C-2, R1), 87.2 (d, 0.55C, C-2, R2), 126.8 (d, 0.9C, 2 × C_{ortho}, R1), 127.2 (d, 1.1C, C_{para}, R2), 127.4 (d, 0.9C, C_{para}, R1), 127.6 (d, 1.1C, 2 × C_{ortho}, R2), 128.6 (d, 1.1C, 2 × C_{meta}, R2), 128.7 (d, 0.9C, 2 × C_{meta}, R1), 137.56 (s, 0.55C, C_{ipso}, R2), 137.58 (s, 0.45C, C_{ipso}, R1), 164.1 (s, 0.55C, C-1, R2), 164.2 (s, 0.45C, C-1, R1), 213.9 (s, 0.55C, C-3, R2), 214.2 (s, 0.45C, C-3, R1).

IR (ATR): $\tilde{\nu}$ [cm^{-1}] = 3029 (w), 2926 (w), 1968 (m, C=C=C), 1945 (m, C=C=C), 1623 (s, C=O), 1448 (s), 1395 (s), 1127 (m), 1077 (s), 846 (s), 699 (s).

HRMS (ESI): [M+H$^+$]: calc.: 189.1133; found: 189.1124.

***N*-Benzyl-*N*-methylbuta-2,3-dienamide-d_2 (7f-d_2)**

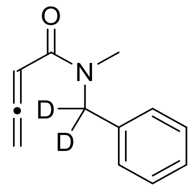

$C_{11}H_{11}D_2NO$
M = 177,24 g/mol

Following **GP-1** [but-3-ynoic acid (450 mg, 5.35 mmol, 1.00 eq.)], *N*-methyl-1-phenylmethan-d_2-amine (1.65 g, 13.4 mmol, 2.5 eq., 99 %D) was coupled with but-3-ynoic acid chloride to but-3-ynamide. After short path chromatography (SiO_2, 3 × 20 cm, P/Et_2O = 6/4) crude but-3-ynamide (753 mg, 3.98 mmol, 33%) was obtained as colorless oil and used without further purification. Following **GP-1**, but-3-ynamide was isomerized to buta-2,3-dienamide **7f-d_2** at room temperature for 24 hours. After column chromatography (SiO_2, 3 × 20 cm, P/Et_2O = 8/2 → 1/1) buta-2,3-dienamide **7f-d_2** (751 mg, 3.97 mmol, quant., 98 %D) was obtained as a yellow oil.

TLC: R_f = 0.49 (Et_2O) [UV, $KMnO_4$].

Rotameric ratio: R1/R2 = 45/55.

^{1}H-NMR (500 MHz, DMSO-d_6, 298 K): δ [ppm] = 2.82 (s, 1.35H, N-CH_3, R1), 2.97 (s, 1.65H, N-CH_3, R2), 5.27 (d, ^4J = 6.5 Hz, 0.9H, C-4-H_2, R1), 5.31 (d, ^4J = 6.5 Hz, 1.1H, C-4-H_2, R2), 6.33 (t, ^4J = 6.5 Hz, 0.55H, C-2-H, R2), 6.39 (t, ^4J = 6.5 Hz, 0.45H, C-2-H, R1), 7.19–7.39 (m, 5H, 2 × C_{ortho}-H, 2 × C_{meta}-H, C_{para}-H).

^{13}C-NMR (126 MHz, DMSO-d_6, 298 K): δ [ppm] = 33.7 (q, 0.45C, N-CH_3, R1), 35.3 (q, 0.55C, N-CH_3, R2), 49.7 (p, $^1J_{CD}$ = 24.1 Hz, 1.1C, N-CH_2-Ph, R2), 52.3 (p, $^1J_{CD}$ = 20.1 Hz, 0.9C, N-CH_2-Ph, R1), 78.9 (t, 1.1C, C-4, R2), 79.0 (t, 0.9C, C-4, R1), 87.1 (d, 0.45C, C-2, R1), 87.2 (d, 0.55C, C-2, R2), 126.8 (d, 0.9C, 2 × C_{ortho}, R1), 127.2 (d, 1.1C, C_{para}, R2), 127.4 (d, 0.9C, C_{para}, R1), 127.6 (d, 1.1C, 2 × C_{ortho}, R2), 128.5 (d, 1.1C, 2 × C_{meta}, R2), 128.7 (d, 0.9C, 2 × C_{meta}, R1), 137.5 (s, C_{ipso}), 164.1 (s, 0.55C, C-1, R2), 164.2 (s, 0.45C, C-1, R1), 213.9 (s, 0.55C, C-3, R2), 214.1 (s, 0.45C, C-3, R1).

IR (ATR): $\tilde{\nu}$ [cm^{-1}] = 3060 (w), 3028 (w), 1968 (m, C=C=C), 1945 (m, C=C=C), 1620 (s, C=O), 1447 (s), 1388 (s), 1130 (m), 846 (s), 717 (s), 700 (s).

HRMS (ESI): [M+H$^+$]: calc.: 190.1195; found: 190.1194.

N-(4-Fluorobenzyl)-*N*-methylbuta-2,3-dienamide (7g)

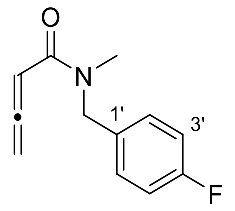

$C_{12}H_{12}FNO$
M = 205,23 g/mol

A solution of but-3-ynoic acid (1.00 g, 11.9 mmol, 1.00 eq.) and 2-chloro-1-methylpyridinium iodide (4.19 g, 16.4 mmol, 1.38 eq.) in MeCN (40 mL) was stirred for one hour at 85°C. Afterwards NEt_3 (2.50 mL, 17.9 mmol, 1.50 eq.) and *N*-(4-fluorobenzyl)-*N*-methylamine (1.75 mL, 13.3 mmol, 1.12 eq.) were added and the solution stirred for 40 min at 85°C and for 16 hours at room temperature. After removal of the solvent under reduced pressure the residue was redissolved in EtOAc (100 mL). The organic phase was washed with H_2O (2 × 100 mL), dried over Na_2SO_4, filtered and the solvent removed under reduced pressure. After column chromatography (SiO_2, 3 × 25 cm, P/Et_2O = 6/4 → 4/6) buta-2,3-dienamide **7g** (220 mg, 1.07 mmol, 9%) was obtained as yellow oil with impurities. The product was used for irradiation experiments without further purification.

TLC: R_f = 0.50 (Et_2O) [UV, $KMnO_4$].

Rotameric ratio: R1/R2 = 40/60.

^{1}H-NMR (400 MHz, DMSO-d_6, 298 K): δ [ppm] = 2.82 (s, 1.2H, N-CH_3, R1), 2.98 (s, 1.8H, N-CH_3, R2), 4.51 (s, 1.2H, N-CH_2-Ar, R2), 4.64 (s, 0.8H, N-CH_2-Ar, R1), 5.26 (d, ^4J = 6.6 Hz, 0.8H, C-4-H_2, R1), 5.30 (d, ^4J = 6.6 Hz, 1.2H, C-4-H_2, R2), 6.31 (d, ^4J = 6.6 Hz, 0.6H, C-2-H, R2), 6.37 (d, ^4J = 6.6 Hz, 0.4H, C-2-H, R1), 7.12–7.33 (m, 4H, 2 × C-2'-H, 2 × C-3'-H).

^{13}C-NMR (101 MHz, DMSO-d_6, 300 K): δ [ppm] = 30.1 (q, 0.4C, N-CH_3, R1), 35.3 (q, 0.6C, N-CH_3, R2), 49.5 (t, 0.6C, N-CH_2-Ar, R2), 52.1 (t, 0.4C, N-CH_2-Ar, R1), 78.77 (t, 0.6C, C-4, R2), 78.81 (t, 0.4C, C-4, R1), 87.0 (d, 0.4C, C-2, R1), 87.1 (d, 0.6C, C-2, R2), 115.2 (d, $^3J_{CF}$ = 20.8 Hz, 1.2C, 2 × C-3', R2), 115.4 (d, $^3J_{CF}$ = 21.2 Hz, 0.8C, 2 × C-3', R1), 128.8 (d, $^4J_{CF}$ = 8.4 Hz, 0.8C, 2 × C-2', R1), 129.6 (d, $^4J_{CF}$ = 8.2 Hz, 1.2C, 2 × C-2', R2), 133.7 (s, C-1'), 161.3 (d, $^2J_{CF}$ = 244 Hz, C-4'), 164.0 (s, C-1), 213.7 (s, 0.6C, C-3, R2), 213.9 (s, 0.4C, C-3, R1).

IR (ATR): $\tilde{\nu}$ [cm^{-1}] = 2928 (w), 1969 (m, C=C=C), 1946 (m, C=C=C), 1721 (C=O), 1604 (s), 1508 (s), 847 (s), 764 (s).

HRMS (ESI): [M+H$^+$]: calc.: 206.0976; found: 206.0975.

N-Methyl-N-(naphthalen-2-ylmethyl)buta-2,3-dienamide (7h)

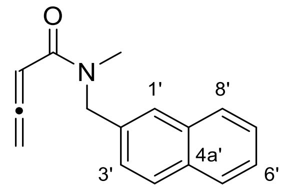

$C_{16}H_{15}NO$
M = 237,30 g/mol

Following **GP-1**, N-methyl-1-(naphthalen-2-yl)methanamine (4.39 g, 25.6 mmol, 2.16 eq.) was coupled with but-3-ynoic acid chloride to but-3-ynamide. After short path chromatography (SiO_2, 3 × 20 cm, EtOAc/Hex = 2/8 → 3/7) crude but-3-ynamide (2.12 g, 8.93 mmol, 75%) was obtained as yellow oil and used without further purification. Following **GP-1**, but-3-ynamide was isomerized to buta-2,3-dienamide **7h** at room temperature for 44 hours. After column chromatography (SiO_2, 3 × 20 cm, P/Et_2O = 4/6 → 7/3) buta-2,3-dienamide **7h** (1.51 g, 6.36 mmol, 71%) was obtained as a white solid.

TLC: R_f = 0.30 (P/Et_2O = 8/2) [UV, $KMnO_4$].

M.p.: 72°C.

Rotameric ratio: R1/R2 = 45/55.

^{1}H-NMR (500 MHz, DMSO-d_6, 298 K): δ [ppm] = 2.89 (s, 1.3H, N-CH_3, R1), 3.02 (s, 1.7H, N-CH_3, R2), 4.70 (s, 1.1H, N-CH_2-Ar, R2), 4.83 (s, 0.9H, N-CH_2-Ar, R1), 5.29 (d, ^3J = 6.5 Hz, 0.9H, C-4-H_2, R1), 5.34 (d, ^3J = 6.5 Hz, 1.1H, C-4-H_2, R2), 6.38 (t, ^3J = 6.5 Hz, 0.55H, C-2-H, R2), 6.44 (t, ^3J = 6.5 Hz, 0.45H, C-2-H, R1), 7.36 (td, ^3J = 8.5 Hz, ^4J = 1.8 Hz, 1H, C-3'), 7.47–7.54 (m, 2H, H_{Ar}), 7.68–7.75 (m, 1H, C-1'), 7.87–7.95 (m, 3H, H_{Ar}).

^{13}C-NMR (126 MHz, DMSO-d_6, 298 K): δ [ppm] = 33.0 (q, 0.45C, N-CH_3, R1), 35.4 (q, 0.55C, N-CH_3, R2), 50.4 (t, 1.1C, N-CH_2-Ar, R2), 53.1 (t, 0.9C, N-CH_2-Ar, R1), 79.0 (t, 1.1C, C-4, R2), 79.1 (t, 0.9C, C-4, R1), 87.2 (d, 0.45C, C-2, R1), 87.3 (d, 0.55C, C-2, R2), 125.07 (d, C_{Ar})*, 125.11 (d, C_{Ar})*, 125.9 (d, C_{Ar})*, 126.0 (d, C_{Ar})*, 126.1 (d, C_{Ar})*, 126.3 (d, C_{Ar})*, 126.5 (d, C_{Ar})*, 127.61 (d, C_{Ar})*, 127.62 (d, C_{Ar})*, 127.64 (d, C_{Ar})*, 127.7 (d, C_{Ar})*, 128.3 (d, C_{Ar})*, 128.4 (d, C_{Ar})*, 132.28 (s, 0.55C, C-4a', R2), 132.33 (s, 0.45C, C-4a', R1), 132.9 (s, 0.55C, C-8a', R2), 133.0 (s, 0.45C, C-8a', R1), 135.25 (s, 0.55C, C-2', R2), 135.29 (s, 0.45C, C-2', R1), 164.2 (s, 0.55C, C-1, R2), 164.3 (s, 0.45C, C-1, R1), 214.0 (s, 0.55C, C-3, R2), 214.2 (s,0.45C, C-3, R1).

* exact assignment not possible, every signal corresponds to a rotamer.

IR (ATR): $\tilde{\nu}$ [cm^{-1}] = 3051 (w), 2974 (w), 1967 (m, C=C=C), 1936 (m, C=C=C), 1619 (s, C=O), 1397 (s), 1245 (s), 1128 (s), 870 (s), 854 (s), 817 (s), 760 (s), 662 (s).

HRMS (ESI): [$M+H^+$]: calc.: 238.1226; found: 238.1215.

N-(4-Bromobenzyl)-N-methylbuta-2,3-dienamide (7i)

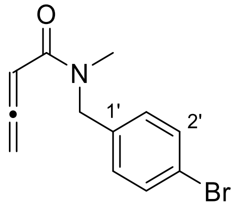

$C_{12}H_{12}BrNO$
M = 266,14 g/mol

Following **GP-1**, *N*-(4-bromobenzyl)-*N*-methylamine (5.00 mL, 25.0 mmol, 2.1 eq.) was coupled with but-3-ynoic acid chloride to but-3-ynamide. After short path chromatography (SiO_2, 4 × 25 cm, P/Et_2O = 8/2) crude but-3-ynamide (1.80 g, 6.76 mmol, 57%) was obtained as yellow oil and used without further purification. Following **GP-1**, but-3-ynamide was isomerized to buta-2,3-dienamide **7i** at room temperature for 27 hours. After column chromatography (SiO_2, 2.5 × 20 cm, P/Et_2O = 8/2) buta-2,3-dienamide **7i** (1.52 g, 5.71 mmol, 85%) was obtained as a colorless oil.

TLC: R_f = 0.41 (Et_2O) [UV, $KMnO_4$].

Rotameric ratio: R1/R2 = 40/60.

^{1}H-NMR (500 MHz, DMSO-d_6, 298 K): δ [ppm] = 2.82 (s, 1.2H, N-CH_3, R1), 2.98 (s, 1.8H, N-CH_3, R2), 4.50 (s, 1.2H, N-CH_2-Ar, R2), 4.64 (s, 0.8H, N-CH_2-Ar, R1), 5.27 (d, ^3J = 6.5 Hz, 0.8H, C-4-H_2, R1), 5.31 (d, ^3J = 6.5 Hz, 1.2H, C-4-H_2, R2), 6.31–6.37 (m, 1H, C-2-H), 7.16 (*virt.* d, J = 8.1 Hz, 0.8H, 2 × C-2'-H, R1), 7.19 (*virt.* d, J = 8.1 Hz, 1.2H, 2 × C-2'-H, R2), 7.53 (*virt.* d, J = 8.1 Hz, 1.2H, 2 × C-3'-H, R2), 7.57 (*virt.* d, J = 8.1 Hz, 0.8H, 2 × C-3'-H, R1).

^{13}C-NMR (126 MHz, DMSO-d_6, 298 K): δ [ppm] = 33.8 (q, 0.4C, N-CH_3, R1), 35.4 (q, 0.6C, N-CH_3, R2), 49.7 (t, 1.2C, N-CH_2-Ar, R2), 52.2 (t, 0.8C, N-CH_2-Ar, R1), 78.86 (t, 1.2C, C-4, R2), 78.92 (t, 0.8C, C-4, R1), 87.0 (d, 0.44C, C-2, R1), 87.1 (d, 0.6C, C-2, R2), 120.2 (s, 0.6C, C-4', R2), 120.3 (s, 0.4C, C-4', R1), 129.0 (d, 0.8C, 2 × C-2', R1), 129.8 (d, 1.2C, 2 × C-2', R2), 131.4 (d, 1.2C, 2 × C-3', R2), 131.5 (d, 0.8C, 2 × C-3', R1), 137.1 (s, C-1'), 164.1 (s, C-1), 213.9 (s, 0.6C, C-3, R2), 214.0 (s, 0.4C, C-3, R1).

IR (ATR): $\tilde{\nu}$ [cm^{-1}] = 3058 (w), 2979 (w), 2928 (w), 1969 (m, C=C=C), 1944 (m, C=C=C), 1624 (s, C=O), 1486 (s), 1394 (s), 1070 (s), 1010 (s), 849 (s), 793 (s).

HRMS (ESI): [M+H$^+$]: calc.: 266.0175; found: 266.0167.

_N_-Benzyl-_N_-(_tert_-butyl)buta-2,3-dienamide (7j)

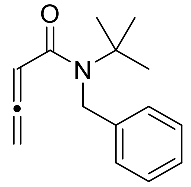

$C_{15}H_{19}NO$
M = 229,32 g/mol

Following **GP-2**, *N*-benzyl-*N*-*tert*-butylamine (5.40 mL, 29.8 mmol, 2.50 eq.) was coupled with but-3-ynoic acid chloride to buta-2,3-dienamide **7j**. After column chromatography (SiO_2, 3 × 20 cm, P/Et_2O = 8/2) buta-2,3-dienamide **7j** (201 mg, 800 µmol, 6%) was obtained as a yellow oil.

TLC: R_f = 0.23 (P/Et_2O = 7/3) [UV, $KMnO_4$].

^{1}H-NMR (400 MHz, DMSO-d_6, 298 K): δ [ppm] = 1.35 [s, 9H, N-C(CH_3)$_3$], 4.69 (s, 2H, N-CH_2-Ph), 5.19 (d, ^4J = 6.5 Hz, 2H, C-4-H_2), 5.96 (t, ^4J = 6.5 Hz, 1H, C-2-H), 7.21–7.28 (m, 3H, 2 × C_{ortho}-H, C_{para}-H), 7.21–7.28 (m, 2H, 2 × C_{meta}-H).

^{13}C-NMR (101 MHz, DMSO-d_6, 298 K): δ [ppm] = 28.1 [q, 3C, N-C(*C*H_3)$_3$], 48.6 (t, N-*C*H_2-Ph), 57.3 (s, N-*C*(CH_3)$_3$), 78.8 (t, C-4), 90.1 (d, C-2), 125.7 (d, 2C, 2 × C_{ortho}), 126.7 (d, C_{para}), 128.5 (d, 2C, 2 × C_{meta}), 139.9 (s, C_{ipso}), 165.4 (s, C-1), 213.7 (s, C-3).

IR (ATR): $\tilde{\nu}$ [cm^{-1}] = 3046 (w), 2987 (w), 2965 (m), 2929 (w), 1941 (m, C=C=C), 1612 (s, C=O), 1495 (m), 1192 (m), 841 (w).

HRMS (ESI): [M+H^+]: calc.: 230.1539; found: 230.1539.

***N*-Benzyl-*N*-isopropylbuta-2,3-dienamide (7k)**

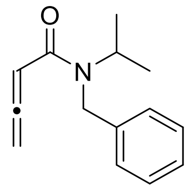

$C_{14}H_{17}NO$
M = 215,30g/mol

Following **GP-1**, *N*-benzyl-*N*-isopropylamine (4.97 mL, 29.7 mmol, 2.5 eq.) was coupled with but-3-ynoic acid chloride to but-3-ynamide. After short path chromatography (SiO_2, 4 × 25 cm, EtOAc/Hex = 1/9) crude but-3-ynamide (430 mg, 2.00 mmol, 36%) was obtained as yellow oil and used without further purification. Following **GP-1**, but-3-ynamide was isomerized to buta-2,3-dienamide **7k** at room temperature for 42 hours. After column chromatography (SiO_2, 3 × 20 cm, EtOAc/Hex = 4/6) buta-2,3-dienamide **7k** (121 mg, 562 µmol, 26%) was obtained as a yellow oil.

TLC: R_f = 0.26 (EtOAc/Hex = 4/6) [UV, $KMnO_4$].

Rotameric ratio: R1/R2 ≈ 55/45.

^{1}H-NMR (500 MHz, DMSO-d_6, 298 K): δ [ppm] = 1.03 [d, ^3J = 6.8 Hz, 3H, N-CH(CH_3)$_2$, R1], 1.07 [d, ^3J = 6.8 Hz, 3H, N-CH(CH_3)$_2$, R2], 4.35–4.41 [m, 0.45H, N-CH(CH$_3$)$_2$, R2], 4.49 [s, 0.9H, N-CH_2-Ph, R2], 4.60 (s, 1.1H, N-CH_2-Ph, R1), 4.61–4.66 [m, 0.55H, N-CH(CH$_3$)$_2$, R1], 5.25 (d, ^4J = 6.5 Hz, 1.1H, C-4-H$_2$, R1), 5.29 (d, ^4J = 6.6 Hz, 0.9H, C-4-H$_2$, R2), 6.00 (t, ^4J = 6.5 Hz, 1.1H, C-2-H, R1), 6.45 (t, ^4J = 6.6 Hz, 1.1H, C-2-H, R2), 7.16–7.30 (m, 4H, 2 × C_{ortho}-H, 2 × C_{meta}-H), 7.36 (*virt.* t, $^3J \approx {}^3J$ = 7.5 Hz, 1H, C_{para}-H).

^{13}C-NMR (126 MHz, DMSO-d_6, 298 K): δ [ppm] = 20.0 [q, 1.1C, N-CH(CH$_3$)$_2$, R1], 21.4 [q, 0.9C, N-CH(CH$_3$)$_2$, R2], 43.4 (t, 0.45C, N-CH$_2$-Ph, R2), 45.7 [d, 0.55C, N-CH(CH$_3$)$_2$, R1], 45.8 (t, 0.55C, N-CH$_2$-Ph, R1), 49.0 [d, 0.45C, N-CH(CH$_3$)$_2$, R2], 78.8 (t, 0.45C, C-4, R2), 79.1 (t, 0.55C, C-4, R1), 87.5 (d, 0.45C, C-2, R2), 88.3 (d, 0.55C, C-2, R1), 126.1 (d, 1.1C, 2 × C_{ortho}, R1), 126.3 (d, 0.45C, C_{para}, R2), 126.7 (d, 0.9C, 2 × C_{ortho}, R2), 126.9 (d, 0.55C, C_{para}, R1), 128.1 (d, 0.9C, 2 × C_{meta}, R2), 128.5 (d, 1.1C, 2 × C_{meta}, R1), 139.5 (s, 0.55C, C_{ipso}, R1), 139.9 (s, 0.55C, C_{ipso}, R2), 164.17 (s, 0.55C, C-1, R1), 164.24 (s, 0.45C, C-1, R2), 213.6 (s, 0.45C, C-3, R2), 214.5 (s, 0.55C, C-3, R1).

IR (ATR): $\tilde{\nu}$ [cm^{-1}] = 3062 (w), 3031 (w), 2976 (w), 2934 (w), 2876 (w), 1968 (m, C=C=C), 1945 (m, C=C=C), 1623 (s, C=O), 1434 (s), 1406 (s), 1196 (s), 1183 (s), 843 (s), 729 (s), 697 (s).

HRMS (ESI): [M+H$^+$]: calc.: 216.1383; found: 216.1376.

***N*-Benzyl-*N*-[(trimethylsilyl)methyl]buta-2,3-dienamide (7l)**

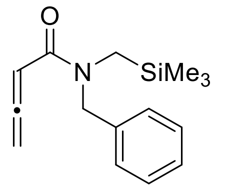

$C_{15}H_{21}NOSi$
M = 259,42 g/mol

Following **GP-1**, *N*-benzyl-1-(trimethylsilyl)methanamine (6.50 mL, 29.7 mmol, 2.5 eq.) was coupled with but-3-ynoic acid chloride to but-3-ynamide. After short path chromatography (SiO_2, 3 × 25 cm, P/Et_2O = 7/3) crude but-3-ynamide (1.67 g, 6.42 mmol, 54%) was obtained as yellow oil and used without further purification. Following **GP-1**, but-3-ynamide was isomerized to buta-2,3-dienamide **7l** at room temperature for 48 hours. After column chromatography (SiO_2, 1.5 × 20 cm, P/Et_2O = 9/1 → 8/2) buta-2,3-dienamide **7l** (987 mg, 3.80 mmol, 59%) was obtained as a colorless oil.

TLC: R_f = 0.20 (Et_2O/P = 4/6) [UV, $KMnO_4$].

Rotameric ratio: R1/R2 = 73/27.

^{1}H-NMR (400 MHz, DMSO-d_6, 298 K): δ [ppm] = -0.04 [s, 6.6H, Si(CH_3)$_3$, R1], 0.05 [s, 2.4H, Si(CH_3)$_3$, R2], 2.77 (s, 1.46H, N-CH_2-SiMe$_3$, R1), 2.95 (s, 0.54H, N-CH_2-SiMe$_3$, R2), 4.51 (s, 0.54H, N-CH_2-Ph, R2), 4.63 (s, 1.46H, N-CH_2-Ph, R1), 5.25 (d, ^4J = 6.6 Hz, 1.46H, C-4-H_2, R1), 5.30 (d, ^4J = 6.5 Hz, 0.54H, C-4-H_2, R2), 6.31–6.39 (m, 1H, C-2-H), 7.19–7.40 (m, 5H, H_{Ar}).

^{13}C-NMR (101 MHz, DMSO-d_6, 298 K): δ [ppm] = -1.69 [q, 0.81C, Si(CH$_3$)$_3$, R2], -1.11 [q, 2.19C, Si(CH$_3$)$_3$, R1], 38.6 (t, N-CH$_2$-SiMe$_3$), 50.1 (t, 0.27C, N-CH$_2$-Ph, R2), 53.2 (t, 0.73C, N-CH$_2$-Ph, R1), 78.8 (t, C-4), 87.0 (d, 0.73C, C-2, R1), 87.5 (d, 0.27C, C-2, R2), 126.9 (d, 1.46C, 2 × C-2’, R1), 127.0 (d, 0.54C, 2 × C-2’, R2), 127.3 (d, 0.73C, C-4’, R1), 127.5 (d, 0.27C, C-4’, R2), 128.4 (d, 0.54C, 2 × C-3’, R2), 128.6 (d, 1.46C, 2 × C-3’, R1), 137.6 (s, C-1’), 163.0 (s, C-1), 213.8 (s, 0.73C, C-2, R1), 214.1 (s, 0.27C, C-2, R2).

IR (ATR): $\tilde{\nu}$ [cm^{-1}] = 3240 (w), 3030 (w), 2951 (w), 2896 (w), 1945 (m), 1620 (s, C=O), 1495 (w), 1448 (m), 1396 (s), 1356 (m), 1247 (m), 1205 (m), 1147 (m), 1078 (m), 1029 (m), 1000 (m), 839 (s), 732 (m), 696 (m).

HRMS (ESI): [M+H$^+$]: calc.: 260.1465; found: 260.1456.

_N_-Benzyl-_N_-[2-(benzyloxy)ethyl]buta-2,3-dienamide (7m)

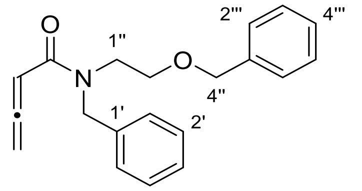

$C_{20}H_{21}NO_2$
M = 307,39 g/mol

Following **GP-1**, *N*-(4-bromobenzyl)-*N*-methylamine (7.20 g, 29.7 mmol, 2.5 eq.) was coupled with but-3-ynoic acid chloride to but-3-ynamide. After short path chromatography (SiO_2, 4 × 25 cm, P/Et_2O = 8/2) crude but-3-ynamide (2.25 g, 7.32 mmol, 61%) was obtained as yellow oil and used without further purification. Following **GP-1**, but-3-ynamide was isomerized to buta-2,3-dienamide **7m** at room temperature for 25 hours. After column chromatography (SiO_2, 4 × 20 cm, Hex/EtOAc = 8/2) buta-2,3-dienamide **7m** (1.19 g, 3.87 mmol, 54%) was obtained as a colorless oil.

TLC: R_f = 0.35 (EtOAc/Hex = 2/8) [UV, $KMnO_4$].

Rotameric ratio: R1/R2 = 59/41.

^{1}H-NMR (400 MHz, DMSO-d_6, 298 K): δ [ppm] = 3.48–3.59 (m, 4H, C-1''-H_2, C-2''-H_2), 4.44 (s, 0.82H, C-4''-H_2, R2), 4.46 (s, 1.18H, C-4''-H_2, R1), 4.59 (s, 1.18H, N-CH_2-Ph, R1), 4.74 (s, 0.82H, N-CH_2-Ph, R2), 5.27 (d, ^4J = 6.5 Hz, 2H, C-4-H_2), 6.28 (t, ^4J = 6.5 Hz, 0.41H, C-2-H, R2), 6.42 (t, ^4J = 6.5 Hz, 0.59H, C-2-H, R1), 7.19–7.39 (m, 10H, H_{Ar}).

^{13}C-NMR (101 MHz, DMSO-d_6, 298 K): δ [ppm] = 45.8 (t, 0.41C, C-1'', R2), 47.1 (t, 0.59C, C-1'', R1), 48.7 (t, 0.59C, N-CH$_2$-Ph, R1), 51.8 (t, 0.41C, N-CH$_2$-Ph, R2), 67.6 (t, 0.41C, C-2'', R2), 68.1 (t, 0.59C, C-2'', R1), 72.0 (t, 0.41C, C-4'', R2), 72.2 (t, 0.59C, C-4'', R1), 79.0 (t, 0.59C, C-4, R1), 79.2 (t, 0.41C, C-4, R2), 87.3 (d, 0.41C, C-2, R2), 87.6 (d, 0.59C, C-2, R1), 126.6 (d, C_{Ar}), 127.1 (d, C_{Ar}), 127.3 (d, C_{Ar}), 127.3 (d, C_{Ar}), 127.47 (d, C_{Ar}), 127.53 (d, C_{Ar}), 127.6 (d, C_{Ar}), 128.25 (d, C_{Ar}), 128.33 (d, C_{Ar}), 128.5 (d, C_{Ar}), 128.7 (d, C_{Ar}), 138.0 (s, 0.41C, C-1', R2), 138.1 (s, 0.59C, C-1', R1), 138.3 (s, 0.59C, C-1''', R1), 138.4 (s, 0.41C, C-1''', R2), 164.3 (s, 0.41C, C-1, R2), 164.4 (s, 0.59C, C-1, R1), 214.2 (s, 0.59C, C-3, R1), 214.4 (s, 0.41C, C-3, R2).

IR (ATR): $\tilde{\nu}$ [cm^{-1}] = 3260 (w), 3030 (w), 2859 (w), 1968 (m), 1945 (m), 1630 (s, C=O), 1496 (m), 1444 (m), 1408 (m), 1359 (m), 1313 (m), 1261 (m), 1205 (m), 1104 (m), 1079 (m), 1028 (m), 846 (m), 734 (m), 697 (m), 614 (m).

HRMS (ESI): [M+H$^+$]: calc.:308.1645; found: 308.1630.

N-Allyl-*N*-phenylbuta-2,3-dienamide (7n)

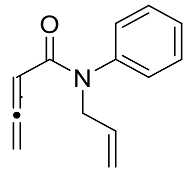

$C_{13}H_{13}NO$
M = 199,25 g/mol

Following **GP-2**, *N*-allyl-*N*-phenylamine (5.00 mL, 36.8 mmol, 3.00 eq.) was coupled with but-3-ynoic acid chloride to but-3-ynamide. After short path chromatography (SiO_2, 4 × 20 cm, EtOAc/P = 2/8) crude but-3-ynamide (1.44 g, 7.25 mmol, 61%) was obtained as orange oil and used without further purification. Following **GP-1**, but-3-ynamide was isomerized to buta-2,3-dienamide **7n** at 40°C for 18 hours. After column chromatography (SiO_2, 3 × 20 cm, EtOAc/P = 1/9) buta-2,3-dienamide **7n** (1.19 g, 5.97 mmol, 82%) was obtained as a yellow oil.

TLC: R_f = 0.55 (EtOAc/P = 3/7) [UV, $KMnO_4$].

^{1}H-NMR (500 MHz, DMSO-d_6, 298 K): δ [ppm] = 4.30 (*virt.* dt, ^3J = 5.9 Hz, $^4J \approx {}^4J$ = 1.5 Hz, 2H, N-CH_2-CH=CH_2), 5.04–5.11 (m, 2H, N-CH_2-CH=CH_2), 5.24 (d, ^4J = 6.5 Hz, 2H, C-4-H_2), 5.51 (bs, 1H, C-2-H), 5.80 (*virt.* ddt, ^3J = 17.7 Hz, ^3J = 9.7 Hz, $^3J \approx {}^3J$ = 5.9 Hz, 1H, N-CH_2-C*H*=CH_2), 7.24–7.28 (m, 2H, 2 × C_{meta}-H), 7.33–7.38 (m, 1H, C_{para}-H), 7.44 (td, ^3J = 7.4 Hz, ^4J = 1.3 Hz, 2H, 2 × C_{ortho}-H).

^{13}C-NMR (101 MHz, DMSO-d_6, 298 K): δ [ppm] = 51.5 (t, N-*C*H_2-CH=CH_2), 79.4 (t, C-4), 87.9 (d, C-2), 117.4 (t, N-CH_2-CH=*C*H_2), 127.6 (d, C_{para}), 128.1 (d, 2C, 2 × C_{ortho}), 129.5 (d, 2C, 2 × C_{meta}), 133.5 (d, N-CH_2-*C*H=CH_2), 141.9 (s, C_{ipso}), 162.9 (s, C-1), 213.9 (s, C-3).

Analytical data matched those previously reported in the literature.[13]

1-(Thiazolidin-3'-yl)buta-2,3-dien-1-one (7o)

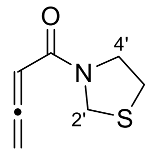

C_7H_9NOS
M = 155,22 g/mol

Following **GP-1**, thiazolidine (2.35 mL, 29.7 mmol, 2.50 eq.) was coupled with but-3-ynoic acid chloride to but-3-ynamide. After short path chromatography (SiO_2, 3 × 20 cm, P/Et_2O = 1/1) crude but-3-ynamide (711 mg, 4.58 mmol, 38%) was obtained as yellow oil and used without further purification. Following **GP-1**, but-3-ynamide was isomerized to buta-2,3-dienamide **7o** at room temperature for 24 hours. After column chromatography (SiO_2, 3 × 20 cm, P/Et_2O = 4/6 →3/7 →2/8) buta-2,3-dienamide **7o** (465 mg, 3.00 mmol, 65%) was obtained as a yellow oil.

TLC: R_f = 0.31 (Et_2O) [UV, $KMnO_4$].

Rotameric ratio: R1/R2 ≈ 50/50.

^{1}H-NMR (500 MHz, DMSO-d_6, 298 K): δ [ppm] = 2.99 (t, ^3J = 6.2 Hz, 1H, C-4'-H_2, R1), 3.09 (t, ^3J = 6.3 Hz, 1H, C-4'-H_2, R2), 3.67 (t, ^3J = 6.2 Hz, 1H, C-5'-H_2, R1), 3.79 (t, ^3J = 6.3 Hz, 1H, C-5'-H_2, R2), 4.48 (s, 1H, C-2'-H_2, R2), 4.62 (s, 1H, C-2'-H_2, R1), 5.34 (d, ^4J = 6.6 Hz, 2H, C-4-H_2), 6.20 (t, ^4J = 6.6 Hz, 1H, C-2-H).

^{13}C-NMR (101 MHz, DMSO-d_6, 298 K): δ [ppm] = 28.9 (t, 0.5C, C-4', R1), 30.7 (t, 0.5C, C-4', R2), 48.1 (t, 0.5C, C-2', R2), 48.5 (t, 0.5C+0.5C, C-2', C-5', R1), 49.2 (t, 0.5C, C-5', R2), 79.19 (t, 0.5C, C-4, R2), 79.22 (t, 0.5C, C-4, R1), 88.3 (d, 0.5C, C-2, R1), 88.4 (d, 0.5C, C-2, R2), 162.1 (s, C-1), 213.5 (s, 0.5C, C-3, R1), 213.6 (s, 0.5C, C-3, R2).

IR (ATR): $\tilde{\nu}$ [cm^{-1}] = 3539 (bw), 3056 (w), 2978 (w), 2935 (w), 2875 (w), 1966 (m, C=C=C), 1942 (m, C=C=C), 1615 (s, C=O), 1433 (s), 1392 (s), 845 (s).

HRMS (ESI): [M+H$^+$]: calc.:156.0478; found: 156.0471.

_N,N_-Dibenzyl-5,5-dimethylhexa-2,3-dienamide (11)

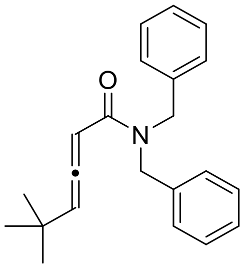

$C_{22}H_{25}NO$
M = 319,45 g/mol

To a solution of 5,5-dimethylhexa-2,3-dienoic acid (650 mg, 4.64 mmol, 1.00 eq.) in CH_2Cl_2 (25 mL) and DMF (100 µL) at 0°C was added oxalyl chloride (418 µL, 4.87 mmol, 1.05 eq.) dropwise. After complete addition the reaction solution was allowed to reach room temperature and stir for one hour until no gas formation was observed. The formed HCl gas was removed by bubbling argon gas through the stirring solution for 45 minutes. Then the solution was cooled to 0°C and dibenzylamine (2.22 mL, 11.6 mmol, 2.50 eq.) was added slowly to the solution. After the addition the reaction mixture was stirred for one hour at 0°C and then for additional two hours at room temperature. The reaction was stopped by addition of aq. HCl-solution (1 M, 50 mL). After separation of the phases the organic phase was washed again with aq. HCl-solution (1 M, 50 mL). The organic phase was dried over Na_2SO_4, filtered and the solvent removed under reduced pressure. After column chromatography (SiO_2, 3 × 25 cm, P/Et_2O = 20/1) a mixture of hexa-2,3-dienamide **11** and *N*,*N*-dibenzyl-5,5-dimethylhex-3-ynamide (476 mg, 1.49 mmol, 32%, **11**/alkyne = 88/12) was obtained as a colorless oil.

TLC: R_f = 0.21 (P/Et_2O = 9/1) [UV, $KMnO_4$].

^{1}H-NMR (400 MHz, DMSO-d_6, 298 K): δ [ppm] = 1.01 [s, 9H, C-4-C(CH_3)$_3$], 4.36–4.70 (m, 4H, 2 × N-CH_2-Ph), 5.66 (d, ^4J = 6.2 Hz, 1H, C-4-H), 6.30 (d, ^4J = 6.2 Hz, 1H, C-2-H), 7.16–7.41 (m, 10H, H_{Ar}).

^{13}C-NMR (101 MHz, DMSO-d_6, 298 K): δ [ppm] = 29.7 [q, 3C, C-4-C(CH_3)$_3$], 32.1 [s, C-4-*C*(CH_3)$_3$], 48.4 (t, N-*C*H_2-Ph, R1), 50.4 (t, N-*C*H_2-Ph, R2), 89.6 (d, C-2), 105.7 (d, C-4), 126.7 (d, 2C, 2 × C_{ortho}, R1), 127.1 (d, C_{para}), 127.2 (d, C_{para}), 127.7 (d, 2C, 2 × C_{ortho}, R2), 128.4 (d, 2C, 2 × C_{meta}, R1), 128.6 (d, 2C, 2 × C_{meta}, R2), 137.4 (s, C_{ipso}), 137.6 (s, C_{ipso}), 164.8 (s, C-1), 208.0 (s, C-3).

_N_-Benzhydryl-_N_-methylbuta-2,3-dienamide (13b)

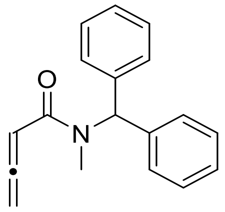

$C_{18}H_{17}NO$
M = 263,34 g/mol

Following **GP-1** [but-3-ynoic acid (600 mg, 7.14 mmol, 1.00 eq.)], *N*-methyl-1,1-diphenylmethanamine (3.52 g, 17.8 mmol, 2.50 eq.) was coupled with but-3-ynoic acid chloride to but-3-ynamide. After short path chromatography (SiO_2, 3 × 30 cm, P/Et_2O = 6/4) crude but-3-ynamide (856 mg, 3.25 mmol, 46%) was obtained as yellow oil and used without further purification. Following **GP-1**, but-3-ynamide was isomerized to buta-2,3-dienamide **13b** at room temperature for 24 hours. After column chromatography (SiO_2, 1.5 × 25 cm, P/Et_2O = 85/15 → 8/2) buta-2,3-dienamide **13b** (452 mg, 1.72 mmol, 53%) was obtained as an off-white solid.

TLC: R_f = 0.38 (P/Et_2O = 1/1) [UV, $KMnO_4$].

M.p.: 99°C.

Rotameric ratio: R1/R2 ≈ 30/70.

^{1}H-NMR (500 MHz, DMSO-d_6, 298 K): δ [ppm] = 2.63 (s, 0.9H, N-CH_3, R1), 2.82 (s, 2.1H, N-CH_3, R2), 5.31 (s, 2H, C-4-H_2), 6.38 (s, 0.7H, C-2-H, R2), 6.45 (s, 0.3H, C-2-H, R1), 6.66 (s, 0.3H, N-C*H*Ph_2, R1), 6.94 (s, 0.7H, N-C*H*Ph_2, R2), 7.15 (d, ^3J = 7.6 Hz, 4H, 4 × C_{ortho}-H), 7.33 (*virt.* t, $^3J \approx {}^3J$ = 7.3 Hz, 2H, 2 × C_{para}-H), 7.15 (dd, ^3J = 7.4 Hz, ^3J = 7.4 Hz, 4H, 4 × C_{meta}-H).

^{13}C-NMR (101 MHz, DMSO-d_6, 298 K): δ [ppm] = 32.6 (q, N-CH_3), 60.2 (d, N-*C*H$(Ph)_2$), 78.9 (t, C-4), 87.4 (d, C-2), 127.4 (d, 2C, 2 × C_{para}), 128.3 (d, 4C, 4 × C_{meta}), 128.5 (d, 4C, 4 × C_{ortho}), 139.1 (s, 2C, 2 × C_{ipso}), 164.5 (s, C-1), 214.0 (s, C-3).

IR (ATR): $\tilde{\nu}$ [cm^{-1}] = 3051 (w), 3026 (w), 2974 (m), 2922 (w), 1966 (m, C=C=C), 1937 (m, C=C=C), 1629 (s, C=O), 1391 (s), 1121 (s), 853 (s), 700 (s).

HRMS (ESI): [M+H^+]: calc.: 264.1383; found: 264.1374.

***N,N*-Diisopropylbuta-2,3-dienamide (13c)**

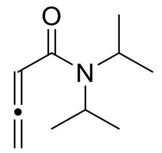

$C_{10}H_{17}NO$
M = 167,25 g/mol

Following **GP-1**, diisopropylamine (4.18 mL, 29.7 mmol, 2.50 eq.) was coupled with but-3-ynoic acid chloride to buta-2,3-dienamide **13c**. After column chromatography (SiO_2, 3 × 20 cm, P/Et_2O = 9/1 → 8/2) buta-2,3-dienamide **13c** (864 mg, 5.17 mmol, 43%) was obtained as a yellow oil.

TLC: R_f = 0.47 (P/Et_2O = 1/1) [UV, $KMnO_4$].

^{1}H-NMR (500 MHz, DMSO-d_6, 298 K): δ [ppm] = 1.15 [d, ^3J = 6.9 Hz, 6H, N-CH(CH_3)$_2$] 1.27 [d, ^3J = 6.8 Hz, 6H, N-CH(CH_3)$_2$], 3.58 [bs, 1H, N-CH(CH$_3$)$_2$], 4.13 [bs, 1H, N-CH(CH$_3$)$_2$], 5.18 (d, ^4J = 6.6 Hz, 2H, C-4-H$_2$), 6.15 (t, ^4J = 6.6 Hz, 1H, C-2-H).

^{13}C-NMR (126 MHz, DMSO-d_6, 298 K): δ [ppm] = 20.5 [q, 2C, N-CH(CH$_3$)$_2$], 20.9 [q, 2C, N-CH(CH$_3$)$_2$], 45.1 [d, N-CH(CH$_3$)$_2$], 48.8 [d, N-CH(CH$_3$)$_2$], 78.4 (t, C-4), 89.1 (d, C-2), 162.8 (s, C-1), 212.5 (s, C-3).

IR (ATR): $\tilde{\nu}$ [cm^{-1}] = 2968 (m), 2934 (w), 2876 (w), 1948 (m, C=C=C), 1623 (s, C=O), 1440 (s), 1333 (s), 1041 (s), 841 (bs).

HRMS (ESI): [M+H$^+$]: calc.: 168.1383; found: 168.1376.

***N,N*-Dicyclohexylbuta-2,3-dienamide (13d)**

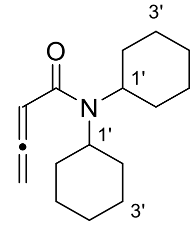

$C_{16}H_{25}NO$
M = 247,38 g/mol

Following **GP-1**, dicyclohexylamine (5.93 mL, 29.7 mmol, 2.50 eq.) was coupled with but-3-ynoic acid chloride to buta-2,3-dienamide **13d**. After column chromatography (SiO_2, 3 × 20 cm, P/Et_2O = 8/2 → 7/3) buta-2,3-dienamide **13d** (701 mg, 2.83 mmol, 24%) was obtained as a yellow oil.

TLC: R_f = 0.29 (P/Et_2O = 7/3) [UV, $KMnO_4$].

^{1}H-NMR (500 MHz, DMSO-d_6, 298 K): δ [ppm] = 0.96–1.76 (m, 18H, H_{cHex}), 2.13–2.38 (m, 2H, H_{cHex}), 3.08 (bs, 1H, C-1'-H), 3.67 (bs, 1H, C-1'-H), 5.18 (d, ^4J = 6.6 Hz, 2H, C-4-H_2), 6.14 (d, ^4J = 6.6 Hz, 1H, C-2-H).

^{13}C-NMR (126 MHz, DMSO-d_6, 298 K): δ [ppm] = 24.7 (t, C-4'), 25.1 (t, C-4'), 25.4 (t, 2C, 2 × C-3'), 25.9 (t, 2C, 2 × C-3'), 29.8 (t, 2C, 2 × C-2'), 30.9 (t, 2C, 2 × C-2'), 54.9 (d, 1C, C-1'), 57.7 (d, 1C, C-1'), 78.2 (t, C-4), 89.3 (d, C-2), 163.1 (s, C-1), 212.2 (s, C-3).

IR (ATR): $\tilde{\nu}$ [cm^{-1}] = 2927 (bs), 2852 (s), 1948 (m, C=C=C), 1621 (s, C=O), 1436 (s), 1364 (s), 1230 (s), 1182 (s), 895 (s), 846 (s), 839 (s), 702 (m).

HRMS (ESI): [M+H$^+$]: calc.: 248.2009; found: 248.1998.

7.2 Intramolecular Cyclization of Tertiary Buta-2,3-dienamides to β-Lactams

General procedure for the intramolecular, photochemical cyclization of buta-2,3-dienamides with XT as photocatalyst (GP-3)

A solution of the corresponding buta-2,3-dienamides **7** (400 μM) and XT (7.85mg, 40.0 μM, 10 mol%) in degassed MeCN (20 mL) was irradiated (λ = 350 nm) in a flame dried *Duran* phototube under Ar atmosphere at 30°C.

***rac*-1-Benzyl-4-phenyl-3-vinylazetidin-2-one (8a)**

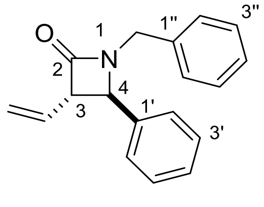

$C_{18}H_{17}NO$
M = 263,34 g/mol

Following **GP-3**, buta-2,3-dienamide **7a** (105 mg, 400 μmol, 1.00 eq.) was converted to β-lactam **8a** by irradiation for one and a half hours. After column chromatography (SiO_2, 1 × 20 cm, P/Et_2O = 1/1 → 7/3) β-lactam **8a** (76.9 mg, 292 μmol, 73%) was obtained as colorless oil.

TLC: R_f = 0.45 (P/Et_2O = 6/4) [UV, $KMnO_4$].

^{1}H-NMR (500 MHz, $CDCl_3$, 298 K): δ [ppm] = 3.68 (ddd, ^3J = 7.8 Hz, ^3J = 2.2 Hz, ^4J = 1.1 Hz, 1H, C-3-H), 3.78 (d, ^2J = 15.1 Hz, 1H, C*H*H-Ph), 4.20 (d, ^3J = 2.2 Hz, 1H, C-4-H), 4.87 (d, ^2J = 15.1 Hz, 1H, CH*H*-Ph), 5.24 (*virt.* dt, ^3J = 10.3 Hz, $^2J \approx {}^4J$ = 1.2 Hz, 1H, C-3-CH=CHH_Z), 5.29 (*virt.* dt, ^3J = 17.2 Hz, $^2J \approx {}^4J$ = 1.2 Hz, 1H, C-3-CH=CHH_E), 5.92 (ddd, ^3J = 17.2 Hz, ^3J = 10.3 Hz, ^3J = 7.8 Hz, 1H, C-3-C*H*=CH_2), 7.12–7.16 (m, 2H, 2 × C-2''-H), 7.22–7.39 (m, 8H, 2 × C-2'-H, 2 × C-3'-H, C-4'-H, 2 × C-3''-H, C-4'').

^{13}C-NMR (126 MHz, $CDCl_3$, 298 K): δ [ppm] = 44.6 (t, N-CH_2-Ph), 60.7 (d, C-4), 64.2 (d, C-3), 119.5 (t, C-3-CH=CH_2), 126.6 (d, 2C, 2 × C-2'), 127.9 (d, C-4'), 128.5 (d, 2C, 2 × C-2''), 128.7 (d, C-4''), 128.9 (d, 2C, 2 × C-3'), 129.2 (d, 2C, 2 × C-3''), 131.0 (d, C-3-*C*H=CH_2), 135.6 (s, C-1''), 137.2 (s, C-1'), 168.2 (s, C-2).

Analytical data matched those previously reported in the literature.[14]

^{1}H,^{1}H-NOESY-spectrum:

NOE-contact between C-4-H and C-3-C*H*=CH2.

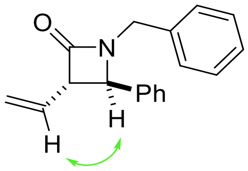

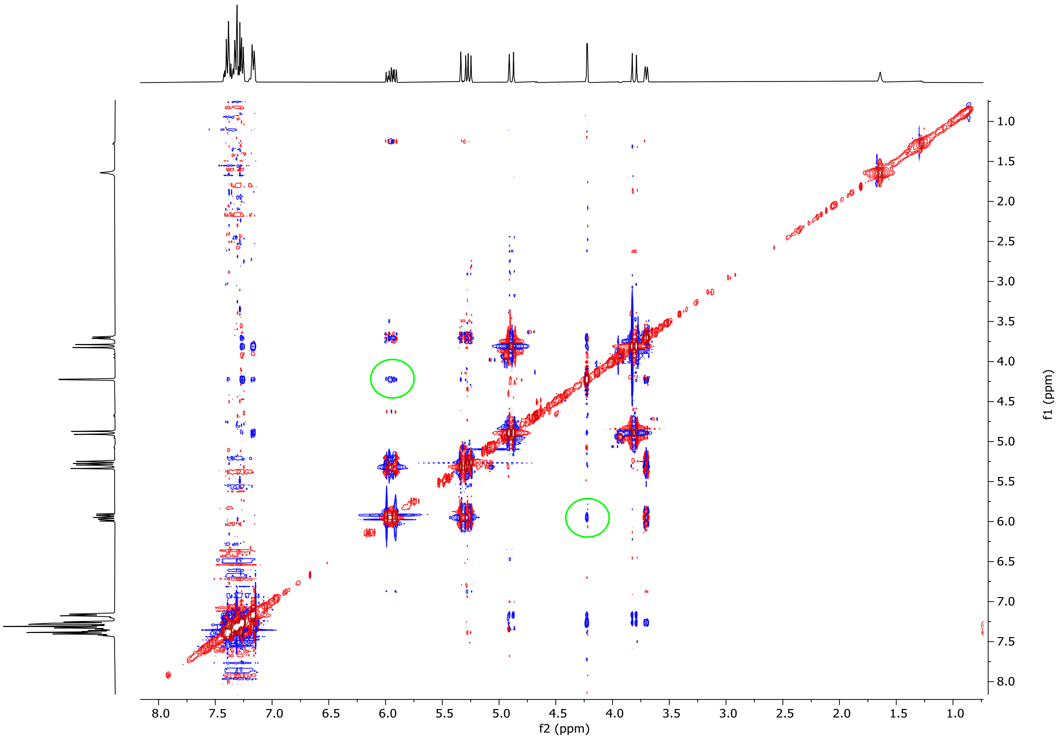

***rac*- 1-(3-Chlorobenzyl)-4-(3-chlorophenyl)-3-vinylazetidin-2-one (8b)**

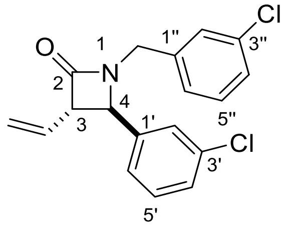

$C_{18}H_{15}Cl_2NO$
M = 332,22 g/mol

Following **GP-3**, buta-2,3-dienamide **7b** (133 mg, 400 µmol, 1.00 eq.) was converted to β-lactam **8b** by irradiation for three hours. After column chromatography (SiO_2, 1 × 20 cm, P/Et_2O = 2/1) β-lactam **8b** (83.7 mg, 252 µmol, 63%) was obtained as colorless oil.

TLC: R_f = 0.46 (P/Et_2O = 1/1) [UV, $KMnO_4$].

^{1}H-NMR (500 MHz, DMSO-d_6, 298 K): δ [ppm] = 3.80 (ddt, ^3J = 7.7 Hz, ^3J = 2.3 Hz, ^4J = 1.2 Hz, 1H, C-3), 4.06 (d, ^2J = 15.7 Hz, 1H, N-C*H*H-Ar), 4.50 (d, ^3J = 2.3 Hz, 1H, C-4-H), 4.55 (d, ^2J = 15.7 Hz, 1H, N-CH*H*-Ar), 5.22 (*virt.* dt, ^3J = 10.4 Hz, $^2J \approx {}^4J$ = 1.4 Hz, 1H, C-3-CH=CHH_Z), 5.31 (*virt.* dt, ^3J = 17.2 Hz, $^2J \approx {}^4J$ = 1.4 Hz, 1H, C-3-CH=CHH_E), 5.98 (ddd, ^3J = 17.2 Hz, ^3J = 10.4 Hz, ^3J = 7.7 Hz, 1H, C-3-C*H*=CH_2), 7.14 (*virt.* dt, ^3J = 7.0 Hz, $^4J \approx {}^4J$ = 1.8 Hz, 1H, H_{Ar}), 7.19–7.21 (m, 1H, H_{Ar}), 7.27–7.40 (m, 6H, H_{Ar}).

^{13}C-NMR (126 MHz, DMSO-d_6, 298 K): δ [ppm] = 43.8 (t, N-CH_2-Ar), 59.5 (d, C-4), 63.3 (d, C-3), 119.1 (t, C-3-CH=*C*H_2), 125.5 (d, C-4’), 126.6 (d, C-2’), 126.7 (d, C-6’), 127.4 (d, C-2’)*, 127.9 (d, C-6’)*, 128.3 (d, C-4’’), 130.5 (d, C-5’’)**, 130.7 (d, C-5’)**, 131.7 (d, C-3-*C*H=CH_2), 133.2 (s, C-3’)***, 133.5 (s, C-3’’)***, 138.6 (s, C-1’’), 140.1 (s, C-1’), 167.3 (s, C-2).

*, **, *** signals are interchangeable

IR (ATR): $\tilde{\nu}$ [cm^{-1}] = 3493 (bw), 3064 (w), 3009 (w), 2919 (w), 1749 (s, C=O), 1598 (m), 1575 (m), 1475 (m), 1435 (m), 1387 (m), 1342 (m), 1294 (m), 1207 (m), 1119 (m), 1077 (m), 925 (m), 787 (s), 717 (s), 690 (s), 683 (s).

HRMS (ESI): [M+H^+]: calc.: 332.0603; found: 332.0604.

^{1}**H,**^{1}**H-NOESY**-spectrum:

NOE-contact between C-4-H and C-3-C*H*=CH_2.

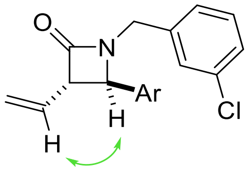

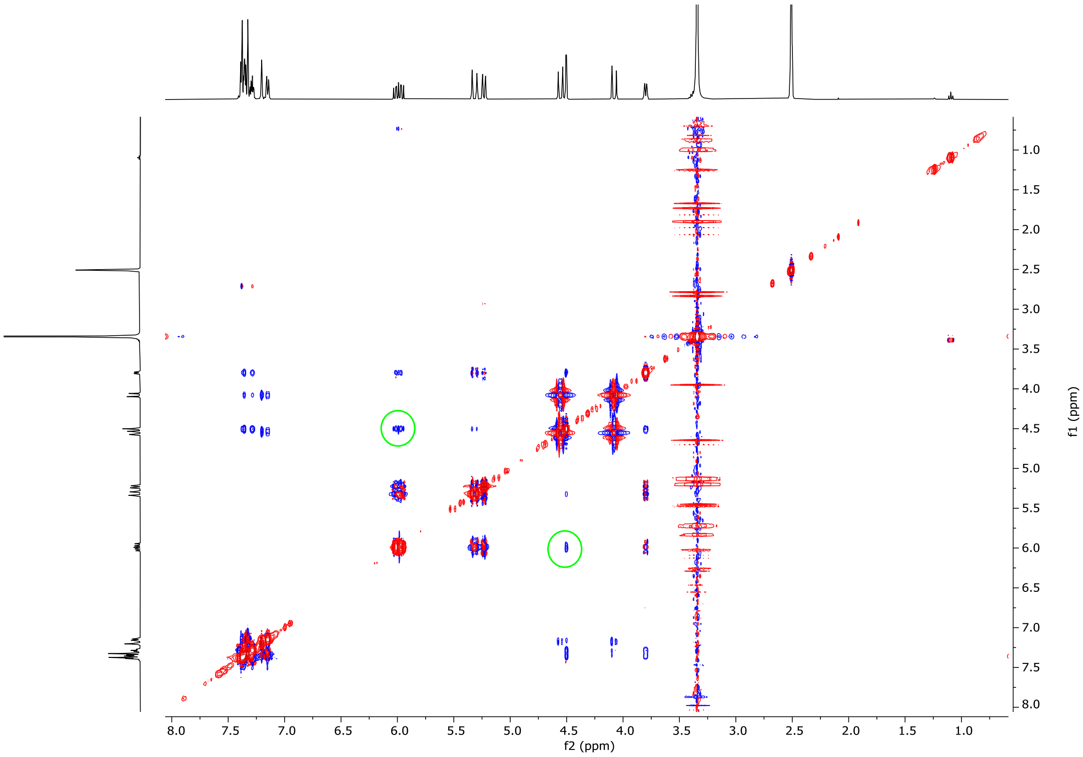

***rac*-1-(4-Fluorobenzyl)-4-(4-fluorophenyl)-3-vinylazetidin-2-one (8c)**

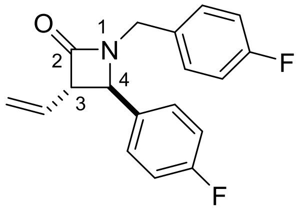

$C_{18}H_{15}F_2NO$
M = 299,32 g/mol

Following **GP-3**, buta-2,3-dienamide **7c** (120 mg, 400 µmol, 1.00 eq.) was converted to β-lactam **8c** by irradiation for two and a half hours. After column chromatography (SiO_2, 1 × 20 cm, P/Et_2O = 9/1 → 7/3) β-lactam **8c** (69.4 mg, 232 µmol, 58%) was obtained as colorless oil.

TLC: R_f = 0.48 (P/Et_2O = 1/1) [UV, $KMnO_4$].

^{1}H-NMR (500 MHz, DMSO-d_6, 298 K): δ [ppm] = 3.70–3.74 (m, 1H, C-3), 3.93 (d, ^2J = 15.5 Hz, 1H, N-C*H*H-Ar), 4.40 (d, ^3J = 2.3 Hz, 1H, C-4-H), 4.56 (d, ^2J = 15.5 Hz, 1H, N-CH*H*-Ar), 5.20 (ddd, ^3J = 10.4 Hz, ^2J = 1.6 Hz, ^4J = 1.0 Hz, 1H, C-3-CH=CHH_Z), 5.28 (*virt.* dt, ^3J = 17.2 Hz, $^2J \approx {}^4J$ = 1.5 Hz, 1H, C-3-CH=CHH_E), 5.96 (ddd, ^3J = 17.2 Hz, ^3J = 10.4 Hz, ^3J = 7.8 Hz, 1H, C-3-C*H*=CH_2), 7.10–7.15 (m, 2H, 2 × C-3''-H), 7.15–7.21 (m, 4H, 2 × C-2''-H, 2 × C-3'-H), 7.33–7.37 (m, 2H, 2 × C-2'-H).

^{13}C-NMR (101 MHz, DMSO-d_6, 298 K): δ [ppm] = 43.3 (t, N-CH_2-Ar), 59.2 (d, C-4), 63.2 (d, C-3), 115.5 (*virt.* t, $^2J_{CF} \approx {}^2J_{CF}$ = 23.1 Hz, 4C, 2 × C-3', 2 × C-3''), 118.8 (t, C-3-CH=CH_2), 128.8 (d, $^3J_{CF}$ = 8.4 Hz, 2C, 2 × C-2'), 130.0 (d, $^3J_{CF}$ = 8.4 Hz, 2C, 2 × C-2''), 131.8 (s, C-1'), 132.2 (d, C-3-*C*H=CH_2), 133.5 (s, C-1''), 161.4 (d, $^1J_{CF}$ = 244 Hz, C-4'), 162.0 (d, $^1J_{CF}$ = 245 Hz, C-4'), 167.1 (s, C-2).

^{19}F-NMR (376 MHz, DMSO-d_6, 298 K): δ [ppm] = -116.9– -116.7 (m, 1F), -115.7– -115. (m, 1F).

IR (ATR): $\tilde{\nu}$ [cm^{-1}] = 3072 (w), 3010 (w), 1971 (w), 1747 (s), 1639 (w), 1604 (m), 1509 (s), 1426 (w), 1391 (m), 1222 (s), 1157 (m), 1054 (m), 924 (m), 834 (s), 765 (w).

HRMS (ESI): [M+H$^+$]: calc.: 300.1194; found: 300.1192.

^{1}H,^{1}H-NOESY-spectrum:

NOE-contact between C-4-H and C-3-C*H*=CH_2.

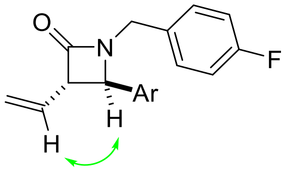

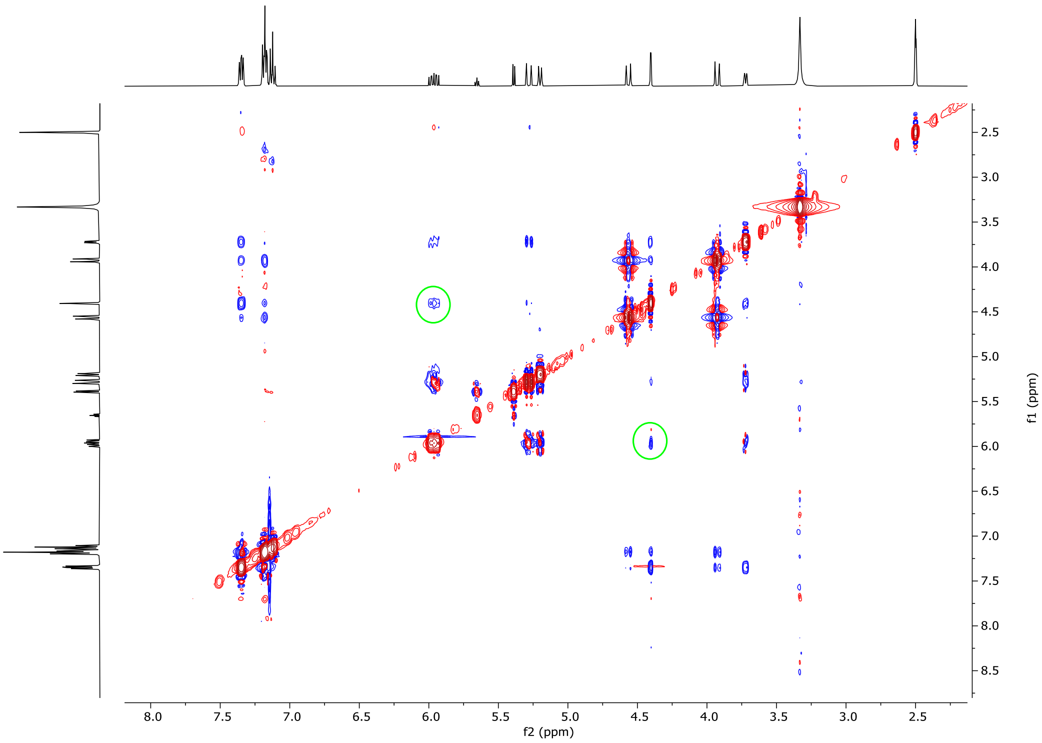

***rac*-1-(3,5-Dimethylbenzyl)-4-(3,5-dimethylphenyl)-3-vinylazetidin-2-one (8d)**

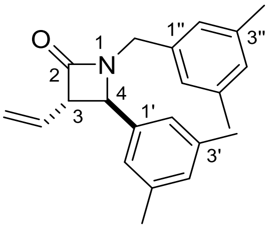

$C_{22}H_{25}NO$
M = 319,45 g/mol

Following **GP-3**, buta-2,3-dienamide **7d** (128 mg, 400 µmol, 1.00 eq.) was converted to β-lactam **8d** by irradiation for five hours. After column chromatography (SiO_2, 1 × 20 cm, P/Et_2O = 9/1) β-lactam **8d** (39.5 mg, 124 µmol, 31%) was obtained as white solid.

TLC: R_f = 0.48 (P/Et_2O = 1/1) [UV, $KMnO_4$].

M.p.: 97°C.

^{1}H-NMR (500 MHz, DMSO-d_6, 298 K): δ [ppm] = 2.21 (s, 6H, 2 × C-3''-CH_3), 2.24 (s, 6H, 2 × C-3'-CH_3), 3.63–3.67 (m, 1H, C-3-H), 3.82 (d, ^2J = 15.4 Hz, 1H, N-C*H*H-Ar), 4.28 (d, ^3J = 2.2 Hz, 1H, C-4), 4.51 (d, ^2J = 15.4 Hz, 1H, N-CH*H*-Ar), 5.18–5.22 (m, 1H, C-3-CH=CHH_Z), 5.28 (*virt.* dt, ^3J = 17.2 Hz, $^2J \approx {}^4J$ = 1.5 Hz, 1H, C-3-CH=CHH_E), 5.96 (ddd, ^3J = 17.2 Hz, ^3J = 10.3 Hz, ^3J = 8.0 Hz, 1H, C-3-C*H*=CH_2), 6.72 (d, ^4J = 1.6 Hz, 2H, 2 × C-2''-H), 6.88 (d, ^4J = 1.6 Hz, 3H, 2 × C-2''-H, C-4''-H), 6.95 (s, 1H, C-4'-H).

^{13}C-NMR (126 MHz, DMSO-d_6, 298 K): δ [ppm] = 20.9 (q, 4C, 2 × C-3'-*C*H_3, 2 × C-3''-*C*H_3), 43.9 (t, N-*C*H_2-Ar), 60.0 (d, C-4), 63.4 (d, C-3), 118.9 (t, C-3-CH=*C*H_2), 124.3 (d, 2C, 2 × C-2'), 125.6 (d, 2C, 2 × C-2''), 128.8 (d, C-4''), 129.8 (d, C-4'), 132.1 (d, C-3-*C*H=CH_2), 135.9 (s, C-1''), 137.3 (s, C-1'), 137.6 (s, 2C, 2 × C-3''), 137.9 (s, 2C, 2 × C-3'), 167.2 (s, C-2).

IR (ATR): $\tilde{\nu}$ [cm^{-1}] = 3079 (w), 3012 (w), 2863 (w), 1743 (s, C=O), 1649 (w), 1603 (w), 1460 (w), 1397 (m), 1344 (m), 1258 (w), 1148 (w), 1064 (w), 945 (w), 842 (m), 715 (m), 683 (m).

HRMS (ESI): [M+H$^+$]: calc.: 320.2009; found: 320.2008.

^{1}H,^{1}H-NOESY-spectrum:

NOE-contact between C-4-H and C-3-C*H*=CH_2.

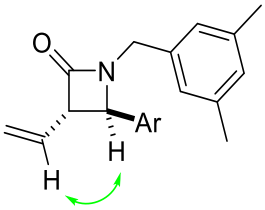

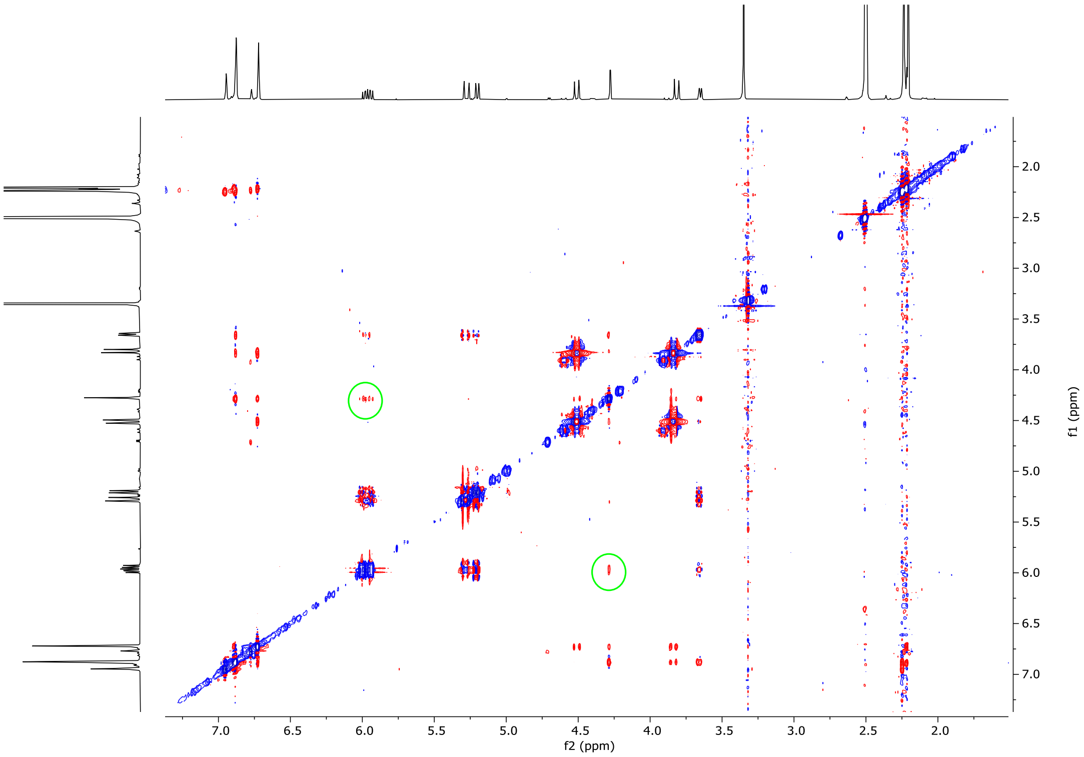

***rac*-1-(4-Methoxybenzyl)-4-(4-methoxyphenyl)-3-vinylazetidin-2-one (8e)**

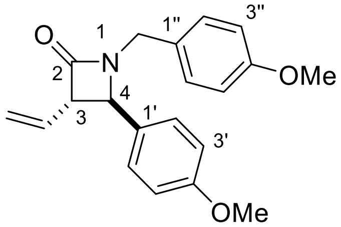

$C_{20}H_{21}NO_3$
M = 323,39 g/mol

Following **GP-3**, buta-2,3-dienamide **7e** (129 mg, 400 µmol, 1.00 eq.) was converted to β-lactam **8e** by irradiation for 16 hours. After column chromatography (SiO_2, 1 × 20 cm, P/Et_2O = 7/3 → 1/1) β-lactam **8e** (6.30 mg, 19.5 µmol, 5%) was obtained as colorless oil.

TLC: R_f = 0.63 (P/Et_2O = 8/2) [$KMnO_4$].

^{1}H-NMR (500 MHz, DMSO-d_6, 298 K): δ [ppm] = 3.64–3.68 (m, 1H, C-3-H), 3.72–3.76 (m, 7H, N-C*H*H-Ar, 2 × OCH_3), 4.25 (d, ^3J = 2.2 Hz, 1H, C-4-H), 4.56 (d, ^2J = 15.3 Hz, 1H, N-CH*H*-Ar), 5.18 (*virt.* dt, ^3J = 10.3 Hz, $^2J \approx {}^4J$ = 1.3 Hz, 1H, C-3-CH=CHH_Z), 5.25 (*virt.* dt, ^3J = 17.1 Hz, $^2J \approx {}^4J$ = 1.5 Hz, 1H, C-3-CH=CHH_E), 5.93 (ddd, ^3J = 17.1 Hz, ^3J = 10.3 Hz, ^3J = 7.9 Hz, 1H, C-3-C*H*=CH_2), 6.87 (*virt.* d, J = 8.7 Hz, 2H, 2 × C-3''-H), 6.93 (*virt.* d, J = 8.7 Hz, 2H, 2 × C-3'-H), 7.05 (*virt.* d, J = 8.7 Hz, 2H, 2 × C-2''-H), 7.23 (*virt.* d, J = 8.7 Hz, 2H, 2 × C-2'-H).

^{13}C-NMR (126 MHz, DMSO-d_6, 298 K): δ [ppm] = 43.1 (t, N-CH_2-Ar), 55.1 (q, C-4''-OCH_3), 55.2 (q, C-4'-OCH_3), 59.3 (d, C-4), 63.2 (d, C-3), 114.1 (d, 2C, 2 × C-3''), 114.3 (d, 2C, 2 × C-3'), 118.7 (t, C-3-CH=CH_2), 127.9 (s, C-1''), 128.0 (d, 2C, 2 × C-2'), 129.0 (s, C-1'), 129.2 (d, 2C, 2 × C-2''), 132.1 (d, C-3-*C*H=CH_2), 158.6 (s, C-4''), 159.3 (s, C-4'), 167.1 (s, C-2).

***rac*-1-Methyl-4-phenyl-3-vinylazetidin-2-one (8f)**

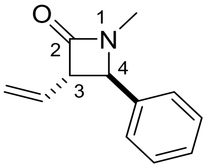

Following **GP-3**, buta-2,3-dienamide **7f** (74.9 mg, 400 µmol, 1.00 eq.) was converted to β-lactam **8f** by irradiation for five hours. After column chromatography (SiO_2, 1 × 20 cm, P/Et_2O = 7/3 → 1/1) β-lactam **8f** (37.9 mg, 202 µmol, 51%) was obtained as a colorless oil.

TLC: R_f = 0.31 (P/Et_2O = 1/1) [$KMnO_4$].

^{1}H-NMR (500 MHz, DMSO-d_6, 298 K): δ [ppm] = 2.67 (s, 1.5H, N-CH_3), 2.67 (s, 1.5H, N-CH_3), 3.60 (ddd, ^3J = 8.1 Hz, ^3J = 2.3 Hz, ^4J = 1.1 Hz, 1H, C-3), 4.46 (d, ^3J = 2.3 Hz, 1H, C-4), 5.20 (ddd, ^3J = 10.4 Hz, ^2J = 1.8 Hz, ^4J = 1.0 Hz, 1H, C-3-CH=CHH_Z), 5.27 (*virt.* dt, ^3J = 17.2 Hz, $^2J \approx {}^4J$ = 1.5 Hz, 1H, C-3-CH=CHH_E), 6.00 (ddd, ^3J = 17.2 Hz, ^3J = 10.4 Hz, ^3J = 8.1 Hz, 1H, C-3-C*H*=CH_2), 7.32–7.39 (m, 3H, 2 × $H_{Ar,meta}$, $H_{Ar,para}$), 7.40–7.45 (m, 2H, 2 × $H_{Ar,ortho}$).

^{13}C-NMR (101 MHz, DMSO-d_6, 298 K): δ [ppm] = 27.2 (q, N-CH_3), 61.7 (d, C-4), 64.4 (d, C-3), 119.2 (t, C-3-CH=*C*H_2), 126.8 (d, 2C, 2 × C_{ortho}), 128.8 (d, C_{para}), 129.4 (d, 2C, 2 × C_{meta}), 132.6 (d, C-3-*C*H=CH_2), 138.1 (s, C_{ipso}), 167.5 (s, C-2).

Analytical data matched those previously reported in the literature.[15]

rac-1-Methyl-4-phenyl-3-vinylazetidin-2-one-d_1 (8f-d_1) and _rac_-1-methyl-4-phenyl-3-(vinyl-1-_d_)azetidin-2-one (8f'-d_1)

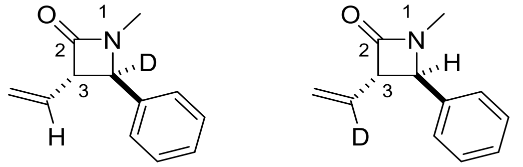

8f-d_1 **8f'-d_1**

$C_{12}H_{13}NO$
M = 187,24 g/mol

Following **GP-3**, buta-2,3-dienamide **7f-d_1** (75.3 mg, 400 µmol, 1.00 eq., 95%D) was converted to β-lactam **8f-d_1**/8f'-d_1 by irradiation for 18 hours. After column chromatography (SiO_2, 1 × 20 cm, P/Et_2O = 7/3 → 1/1) β-lactam **8f-d_1/8f'-d_1** (37.6 mg, 200 µmol, 50%, **8f/8f'** = 3.43/1.00) was obtained as a colorless oil.

TLC: R_f = 0.31 (P/Et_2O = 1/1) [$KMnO_4$].

^{1}H-NMR (500 MHz, DMSO-d_6, 298 K): δ [ppm] = 2.66 (s, 1.5H, N-CH_3), 2.66 (s, 1.5H, N-CH_3), 3.59 (ddd, ^3J = 7.1 Hz, ^3J = 2.2 Hz, ^4J = 1.0 Hz, 1H, C-3-H), 4.46 (d, ^3J = 2.3 Hz, 1H, C-4-H, **8f'-d_1**), 5.19 (ddd, ^3J = 10.4 Hz, ^2J = 1.8 Hz, ^4J = 1.0 Hz, 1H, C-3-CH=CHH_Z), 5.27 (*virt.* dt, ^3J = 17.1 Hz, $^2J \approx {}^4J$ = 1.5 Hz, 1H, C-3-CH=CHH_E), 5.99 (ddd, ^3J = 17.1 Hz, ^3J = 10.4 Hz, ^3J = 8.1 Hz, 1H, C-3-C*H*=CH_2, **8f-d_1**), 7.32–7.39 (m, 3H, 2 × $H_{Ar,meta}$, $H_{Ar,para}$), 7.40–7.45 (m, 2H, 2 × $H_{Ar,ortho}$).

^{13}C-NMR (126 MHz, DMSO-d_6, 298 K): δ [ppm] = 26.78 (q, N-CH_3, **8f-d_1**), 26.81 (q, N-CH_3, **8f'-d_1**), 60.8 (t, $^2J_{CD}$ = 23.6 Hz, C-4), 61.2 (d, C-4, **8f-d_1**), 63.9 (d, C-3, **8f-d_1**), 64.0 (d, C-3, **8f'-d_1**), 118.7 (t, C-3-CD=*C*H_2, **8f'-d_1**), 118.9 (t, C-3-CH=*C*H_2, **8f-d_1**), 126.4 (d, 2C, 2 × C_{ortho}), 128.3 (d, C_{para}), 129.0 (d, 2C, 2 × C_{meta}), 131.9 (t, $^2J_{CD}$ = 23.5 Hz, C-3-*C*D=CH_2, **8f'-d_1**), 132.2 (d, C-3-*C*H=CH_2, **8f-d_1**), 137.6 (s, C_{ipso}, **8f-d_1**), 137.7 (s, C_{ipso}, **8f'-d_1**), 167.09 (s, C-2, **8f'-d_1**), 167.11 (s, C-2, **8f-d_1**).

IR (ATR): $\tilde{\nu}$ [cm^{-1}] = 3086 (w), 3070 (w), 3023 (w), 2983 (w), 2962 (w), 2911 (w), 1735 (s, C=O), 1423 (s), 1387 (s), 988 (s), 937 (s), 751 (s), 702 (s).

HRMS (ESI): [M+H^+]: calc.:189.1133; found:189.1125.

rac-1-Methyl-4-phenyl-3-(vinyl-1-*d*)azetidin-2-one-*d* (8f-*d*2)

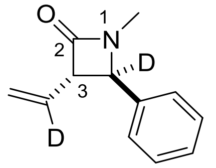

$C_{12}H_{11}D_2NO$
M = 189,25 g/mol

Following **GP-3**, buta-2,3-dienamide **7f-*d*2** (75.7 mg, 400 µmol, 1.00 eq., 98%D) was converted to β-lactam **8f-*d*2** by irradiation for 18 hours. After column chromatography (SiO_2, 1 × 20 cm, P/Et_2O = 7/3 → 1/1) β-lactam **8f-*d*2** (29.5 mg, 156 µmol, 39%, **8f-*d*2**/**8f-*d*1** = 89/11) was obtained as a colorless oil.

TLC: R_f = 0.31 (P/Et_2O = 1/1) [$KMnO_4$].

^{1}H-NMR (500 MHz, DMSO-d_6, 298 K): δ [ppm] = 2.66 (s, 1.5H, N-CH_3), 2.66 (s, 1.5H, N-CH_3), 3.58 (d, ^4J = 1.8 Hz, 1H, C-3-H), 5.19 (d, ^4J = 1.8 Hz, 1H, C-3-CD=CHH_Z), 5.26 (d, ^4J = 1.8 Hz, 1H, C-3-CD=CHH_E), 7.31–7.38 (m, 3H, 2 × $H_{Ar,meta}$, $H_{Ar,para}$), 7.39–7.44 (m, 2H, 2 × $H_{Ar,ortho}$).

^{13}C-NMR (126 MHz, DMSO-d_6, 298 K): δ [ppm] = 26.8 (q, N-CH_3), 60.8 (t, $^2J_{CD}$ = 23.0 Hz, C-4), 63.9 (d, C-3), 118.8 (t, C-3-CD=CH_2), 126.4 (d, 2C, 2 × C_{ortho}), 128.4 (d, C_{para}), 129.0 (d, 2C, 2 × C_{meta}), 131.9 (t, $^2J_{CD}$ = 23.7 Hz, C-3-*C*D=CH_2), 137.7 (s, C_{ipso}), 167.2 (s, C-2).

IR (ATR): $\tilde{\nu}$ [cm^{-1}] = 3086 (w), 3069 (w), 3023 (w), 3006 (w), 2983 (w), 2961 (w), 2910 (w), 1735 (s, C=O), 1423 (s), 1387 (s), 987 (s), 937 (s), 767 (s), 702 (s).

HRMS (ESI): [$M+H^+$]: calc.:190.1195; found:190.1194.

rac-4-(4-Fluorophenyl)-1-methyl-3-vinylazetidin-2-one (8g)

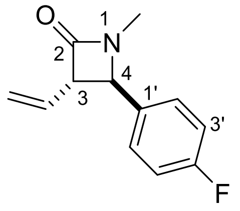

$C_{12}H_{12}FNO$
M = 205,23 g/mol

Following **GP-3**, buta-2,3-dienamide **7g** (68.1 mg, 332 µmol, 1.00 eq.) was converted to β-lactam **8g** by irradiation for three hours. After column chromatography (SiO_2, 1 × 20 cm, P/Et_2O = 1/1) β-lactam **8g** (45.5 mg, 222 µmol, 67%) was obtained as a yellow oil.

TLC: R_f = 0.78 (Et_2O) [$KMnO_4$].

^{1}H-NMR (500 MHz, $CDCl_3$, 298 K): δ [ppm] = 2.77 (s, 1.5H, N-CH_3), 2.77 (s, 1.5H, N-CH_3), 3.56–3.60 (m, 1H, C-3), 4.28 (d, ^3J = 2.2 Hz, 1H, C-4), 5.25 (*virt.* dt, ^3J = 10.2 Hz, $^2J \approx {}^4J$ = 1.3 Hz 1H, C-3-CH=CHH_Z), 5.29 (*virt.* dt, ^3J = 17.1 Hz, $^2J \approx {}^4J$ = 1.3 Hz, 1H, C-3-CH=CHH_E), 5.96 (ddd, ^3J = 17.1 Hz, ^3J = 10.3 Hz, ^3J = 7.9 Hz, 1H, C-3-C*H*=CH_2), 7.06–7.11 (m, 2H, 2 × C-3'-H), 7.24–7.28 (m, 2H, 2 × C-2'-H).

^{13}C-NMR (101 MHz, $CDCl_3$, 298 K): δ [ppm] = 27.2 (q, N-CH_3), 62.5 (d, C-4), 64.9 (d, C-3), 116.3 (d, $^2J_{CF}$ = 21.8 Hz, 2C, 2 × C-3'), 119.5 (t, C-3-CH=*C*H_2), 128.1 (d, $^3J_{CF}$ = 8.2 Hz, 2C, 2 × C-2'), 131.0 (d, C-3-*C*H=CH_2), 133.2 (d, $^4J_{CF}$ = 3.1 Hz, C-1'), 163.0 (d, $^1J_{CF}$ = 247.4 Hz, C-4'), 168.2 (s, C-2).

^{19}F-NMR (376 MHz, $CDCl_3$, 298 K): δ [ppm] = -117.9 (s).

IR (ATR): $\tilde{\nu}$ [cm^{-1}] = 2930 (w), 1749 (s, C=O), 1639 (m), 1509 (m), 1429 (m), 1223 (m), 1158 (m), 816 (w), 733 (w).

HRMS (ESI): [M+H$^+$]: calc.: 206.0976; found: 206.0976.

rac-1-Methyl-4-(naphthalen-2’-yl)-3-vinylazetidin-2-one (8h)

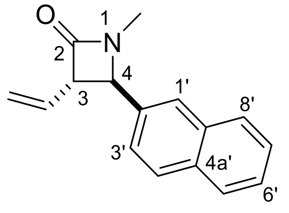

$C_{16}H_{15}NO$
M = 237,30 g/mol

Following **GP-3**, buta-2,3-dienamide **7h** (94.9 mg, 400 µmol, 1.00 eq.) was converted to β-lactam **8h** by irradiation for 30 hours. After column chromatography (SiO_2, 1 × 20 cm, P/Et_2O = 7/3 → 6/4) β-lactam **8h** (41.7 mg, 176 µmol, 44%) was obtained as a white solid.

TLC: R_f = 0.40 (P/Et_2O = 1/1) [UV, $KMnO_4$].

M.p.: 70°C.

^{1}H-NMR (500 MHz, DMSO-d_6, 298 K): δ [ppm] = 2.72 (s, 1.5H, N-CH_3), 2.72 (s, 1.5H, N-CH_3), 3.70 (ddd, ^3J = 8.2 Hz, ^3J = 2.3 Hz, ^4J = 1.1 Hz, 1H, C-3), 4.63 (d, ^3J = 2.3 Hz, 1H, C-4), 5.22 (ddd, ^3J = 10.3 Hz, ^4J = 1.5 Hz, ^2J = 0.9 Hz 1H, C-3-CH=CHH_Z), 5.29 (*virt.* dt, ^3J = 17.1 Hz, $^2J \approx {}^4J$ = 1.5 Hz, 1H, C-3-CH=CHH_E), 6.05 (ddd, ^3J = 17.2 Hz, ^3J = 10.3 Hz, ^3J = 8.2 Hz, 1H, C-3-C*H*=CH_2), 7.45 (dd, ^3J = 8.5 Hz, ^4J = 1.8 Hz, 1H, C-3’-H), 7.06–7.11 (m, 2H, 2 × C-3’-H), 7.51–7.57 (m, 2H, C-6’-H, C-7’-H), 7.90–8.00 (m, 4H, C-1’-H, C-4’-H, C-5’-H, C-8’-H).

^{13}C-NMR (126 MHz, DMSO-d_6, 298 K): δ [ppm] = 27.0 (q, N-CH_3), 61.4 (d, C-4), 64.0 (d, C-3), 119.0 (t, C-3-CH=*C*H_2), 123.8 (d, C-1’), 125.7 (d, C-3’), 126.4 (d, C-7’), 126.6 (d, C-6’), 127.7 (d, C-8’), 127.9 (d, C-5’), 128.8 (d, C-4’), 132.2 (d, C-3-*C*H=CH_2), 132.9 (s, C-8a’), 133.0 (s, C-4a’), 135.3 (s, C-2’), 167.1 (s, C-2).

IR (ATR): $\tilde{\nu}$ [cm^{-1}] = 3082 (w), 3064 (w), 2937 (w), 2905 (w), 1752 (bs, C=O), 1386 (s), 1002 (s), 929 (s), 816 (s), 737 (s).

HRMS (ESI): [M+H$^+$]: calc.:238.1226; found: 238.1216.

^{1}H,^{1}H-NOESY-spectrum:

NOE-contact between C-4-H and C-3-C*H*=CH_2.

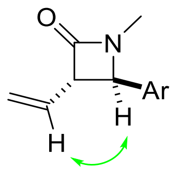

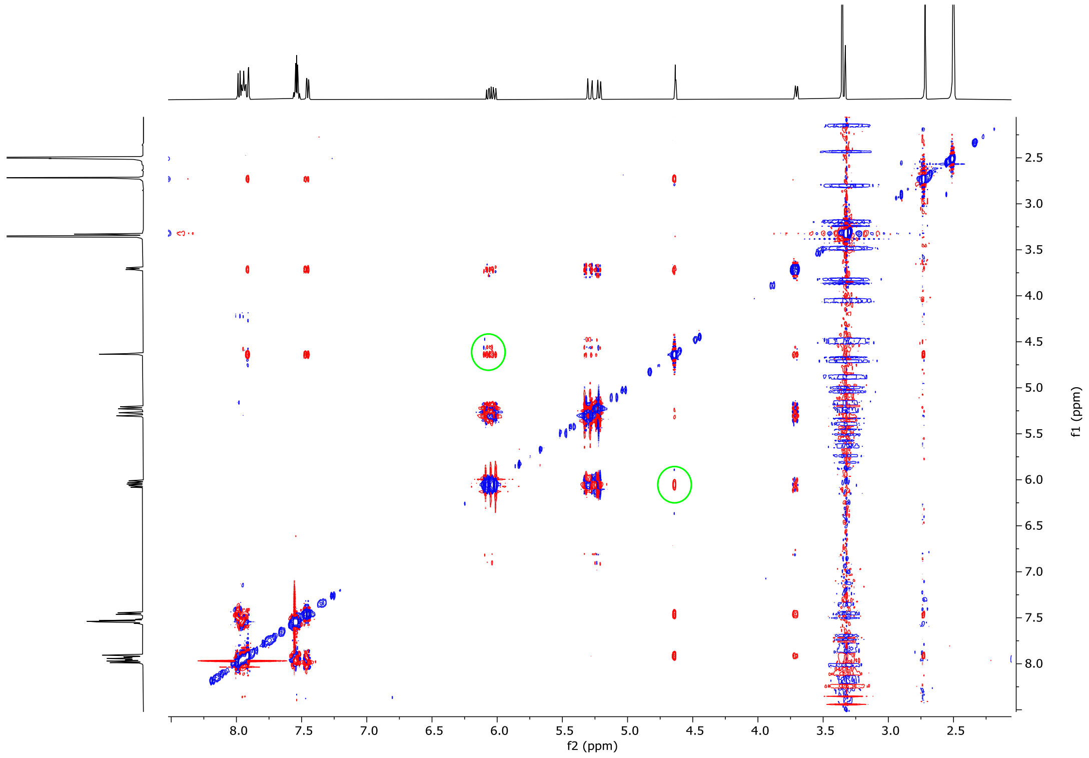

rac-4-(4-Bromophenyl)-1-methyl-3-vinylazetidin-2-one (8i)

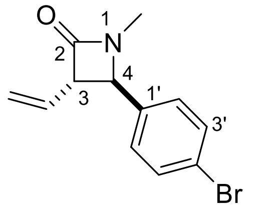

$C_{12}H_{12}BrNO$
M = 266,14 g/mol

Following **GP-3**, buta-2,3-dienamide **7i** (106 mg, 400 µmol, 1.00 eq.) was converted to β-lactam **8i** by irradiation for six hours. After column chromatography (SiO_2, 1 × 20 cm, P/Et_2O = 7/3) β-lactam **8i** (56.8 mg, 212 µmol, 53%) was obtained as a white solid.

TLC: R_f = 0.32 (P/Et_2O = 1/1) [$KMnO_4$].

M.p.: 62°C.

^{1}H-NMR (500 MHz, DMSO-d_6, 298 K): δ [ppm] = 2.66 (s, 1.5H, N-CH_3), 2.66 (s, 1.5H, N-CH_3), 3.60 (ddd, ^3J = 8.1 Hz, ^3J = 2.3 Hz, ^4J = 1.1 Hz, 1H, C-3-H), 4.47 (d, ^3J = 2.3 Hz, 1H, C-4-H), 5.20 (ddd, ^3J = 10.3 Hz, ^2J = 1.7 Hz, ^4J = 1.1 Hz, 1H, C-3-CH=CHH_Z), 5.27 (ddd, ^3J = 17.1 Hz, ^2J = 1.7 Hz, ^4J = 1.1 Hz, 1H, C-3-CH=CHH_E), 5.98 (ddd, ^3J = 17.2 Hz, ^3J = 10.3 Hz, ^3J = 8.1 Hz, 1H, C-3-C*H*=CH_2), 7.31 (*virt.* d, J = 8.5 Hz, 2H, 2 × C-2'-H), 7.61 (*virt.* d, J = 8.5 Hz, 2H, 2 × C-3'-H).

^{13}C-NMR (126 MHz, DMSO-d_6, 298 K): δ [ppm] = 26.8 (q, N-CH_3), 60.5 (d, C-4), 63.9 (d, C-3), 119.0 (t, C-3-CH=*C*H_2), 121.4 (s, C-4'), 128.7 (d, 2C, 2 × C-2'), 131.9 (d, 2C, 2 × C-3'), 132.0 (d, C-3-*C*H=CH_2), 137.2 (s, C-1'), 167.0 (s, C-2).

IR (ATR): $\tilde{\nu}$ [cm^{-1}] = 3493 (w), 3085 (w), 2958 (w), 2903 (w), 1760 (s, C=O), 1637 (m), 1428 (s), 1386 (s), 996 (s), 941 (s), 813 (s).

HRMS (ESI): [M+H^+]: calc.: 266.0175; found: 266.0163.

^{1}H,^{1}H-NOESY-spectrum:

NOE-contact between C-4-H and C-3-C*H*=CH$_2$.

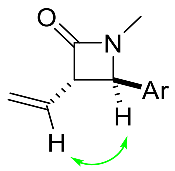

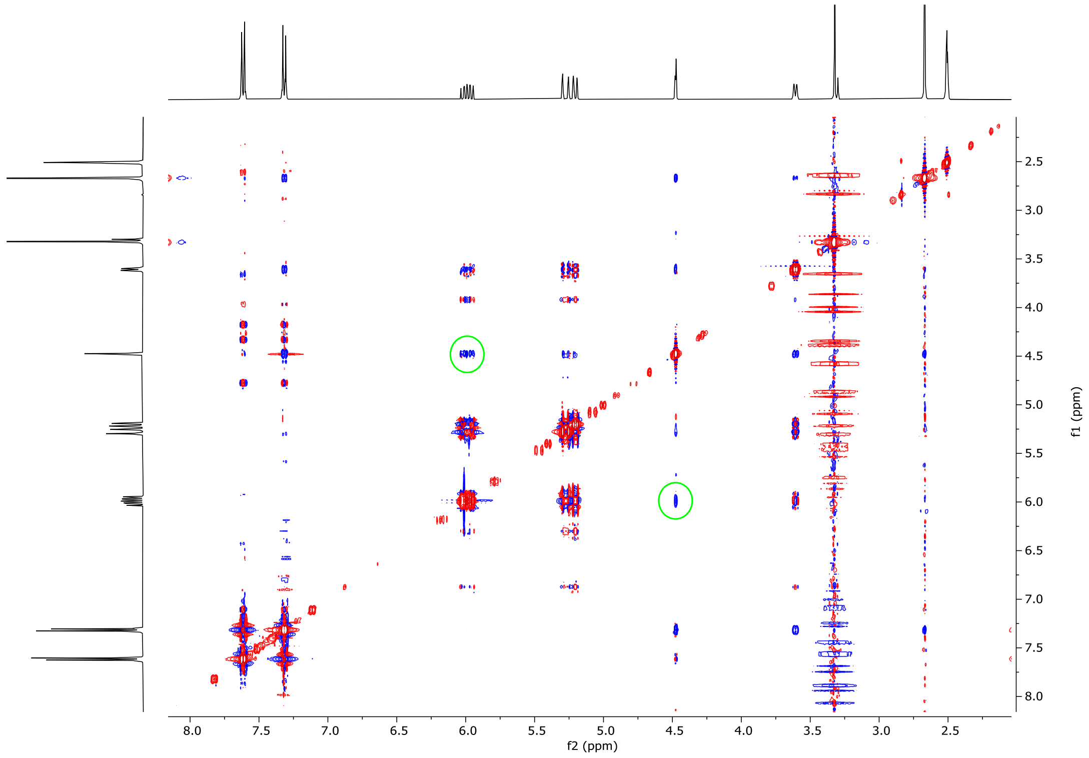

***rac*-1-(*Tert*-butyl)-4-phenyl-3-vinylazetidin-2-one (8j)**

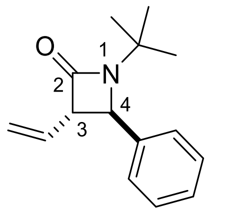

$C_{15}H_{19}NO$
M = 229,32 g/mol

Following **GP-3**, buta-2,3-dienamide **7j** (91.3 mg, 398 µmol, 1.00 eq.) was converted to β-lactam **8j** by irradiation for six hours. After column chromatography (SiO_2, 1 × 20 cm, P/Et_2O = 7/3) β-lactam **8j** (34.2 mg, 149 µmol, 37%) was obtained as a yellow oil.

TLC: R_f = 0.45 (P/Et_2O = 6/4) [$KMnO_4$].

^{1}H-NMR (500 MHz, $CDCl_3$, 298 K): δ [ppm] = 1.26 (s, 9H, N-C(CH_3)$_3$), 3.45 (ddt, ^3J = 7.9 Hz, ^3J =2.2 Hz, ^4J = 1.1 Hz, 1H, C-3-H), 4.33 (d, ^3J = 2.2 Hz, 1H, C-4-H), 5.23 (*virt.* dt, ^3J = 10.4 Hz, $^2J \approx {}^4J$ = 1.1 Hz, 1H, C-3-CH=HH_Z), 5.27 (*virt.* dt, ^3J = 17.1 Hz, $^2J \approx {}^4J$ = 1.3 Hz, 1H, C-3-CH=HH_E), 5.92 (ddd, ^3J = 17.1 Hz, ^3J =10.4 Hz, ^3J = 7.9 Hz, 1H, C-3-C*H*=CH_2), 7.30–7.34 (m, 1H, C-4'-H), 7.36–7.40 (m, 4H, 2 × C-2'-H, 2 × C-3'-H).

^{13}C-NMR (126 MHz, $CDCl_3$, 298 K): δ [ppm] = 28.3 (q, 3C, N-C(CH$_3$)$_3$), 54.7 (s, N-C(CH_3)$_3$), 60.7 (d, C-4), 62.8 (d, C-3), 119.1 (t, C-3-CH=CH$_2$), 126.5 (d, 2C, 2 × C-2'), 128.4 (d, C-4'), 129.0 (d, 2C, 2 × C-3'), 131.4 (d, C-3-CH=CH_2), 140.4 (s, C-1'), 168.4 (C-2).

Analytical data matched those previously reported in the literature.[16]

***rac*-4-phenyl-1-[(trimethylsilyl)methyl]-3-vinylazetidin-2-one (8l)**

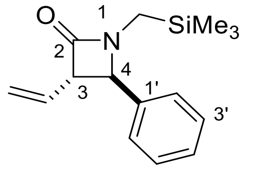

$C_{15}H_{21}NOSi$
M = 259,42 g/mol

Following **GP-3**, buta-2,3-dienamide **7l** (104 mg, 400 µmol, 1.00 eq.) was converted to β-lactam **8l** by irradiation for five hours. After column chromatography (SiO_2, 1 × 20 cm, P/Et_2O = 9/1 → 8/2) β-lactam **8l** (22.3 mg, 86.0 µmol, 21%) was obtained as a colorless oil.

TLC: R_f = 0.47 (P/Et_2O = 6/4) [UV, $KMnO_4$].

^{1}H-NMR (400 MHz, DMSO-d_6, 298 K): δ [ppm] =0.04 [s, 9H, Si(CH_3)$_3$], 2.28 (dd, ^2J = 15.3 Hz, ^5J = 1.1 Hz, 1H, N-C*H*H-SiMe$_3$), 2.68 (dd, dd, ^2J = 15.3 Hz, ^5J = 0.7 Hz, 1H, N-CH*H*-SiMe$_3$), 3.55 (ddd, ^3J = 7.9 Hz, ^3J = 2.2 Hz, ^4J = 1.1 Hz, 1H, C-3-H), 4.39 (d, ^3J = 2.2 Hz, 1H, C-4-H), 5.20 (ddd, ^3J = 10.4 Hz, ^2J = 1.7 Hz, ^4J = 1.0 Hz, 1H, C-3-CH=CHH_Z), 5.27 (ddd, ^3J = 17.2 Hz, ^2J = 1.7 Hz, ^4J = 1.1 Hz, 1H, C-3-CH=CHH_E), 5.98 (ddd, ^3J = 17.2 Hz, ^3J = 10.4 Hz, ^3J = 7.9 Hz, 1H, C-3-C*H*=CH$_2$), 7.30–7.38 (m, 3H, 2 × C-2'-H, C-4'-H), 7.40–7.45 (m, 2H, 2 × C-3'-H).

^{13}C-NMR (101 MHz, DMSO-d_6, 298 K): δ [ppm] = -1.76 [q, 3C, Si(*C*H$_3$)$_3$], 32.0 (t, N-*C*H$_2$-SiMe$_3$), 62.0 (d, C-4), 63.2 (d, C-3), 118.4 (t, C-3-CH=*C*H$_2$), 126.6 (d, 2C, 2 × C-2'), 128.3 (d, C-4'), 128.9 (d, 2C, 2 × C-3'), 132.6 (d, C-3-*C*H=CH$_2$), 137.6 (s, C-1'), 166.6 (s, C-2).

IR (ATR): $\tilde{\nu}$ [cm^{-1}] = 3490 (w), 2953 (w), 2899 (w), 1745 (s, C=O), 1639 (w), 1496 (w), 1456 (w), 1385 (w), 1357 (w), 1248 (m), 1105 (w), 1075 (w), 1028 (w), 988 (w), 922 (w), 856 (s), 751 (m), 699 (s), 648 (w).

HRMS (ESI): [M+H$^+$]: calc.: 260.1465; found: 260.1457.

^{1}**H,**^{1}**H-NOESY**-spectrum:

NOE-contact between C-4-H and C-3-C*H*=CH$_2$.

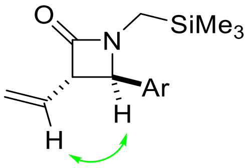

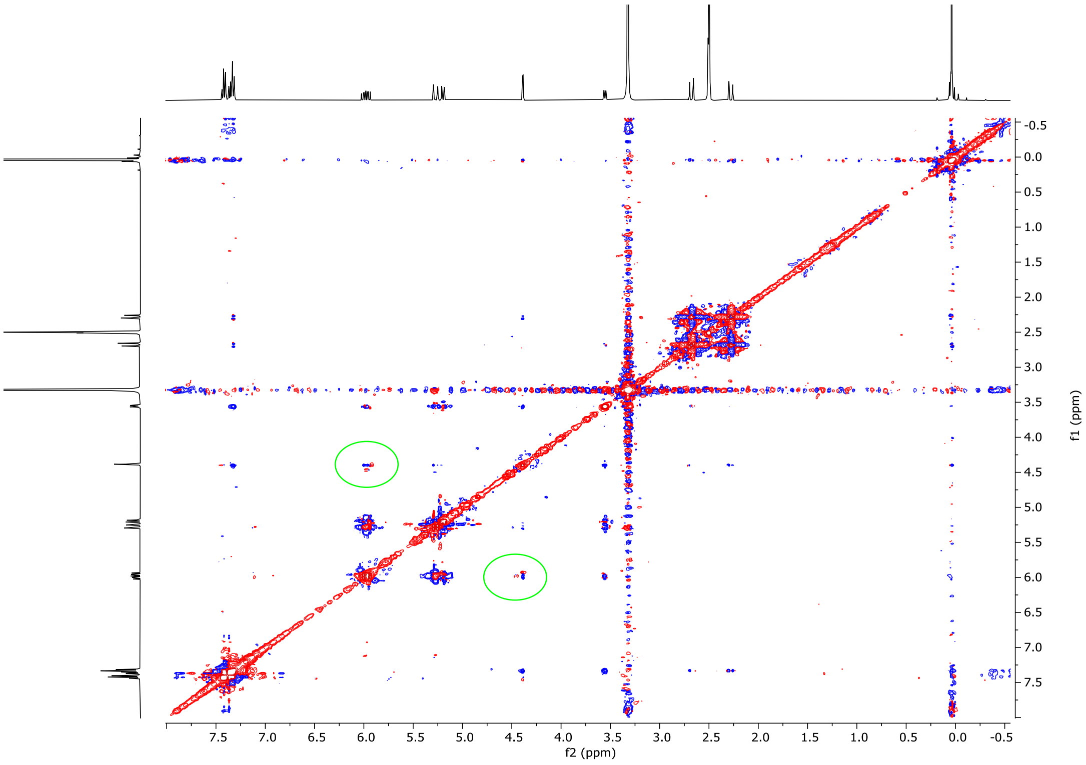

rac-1-[2-(benzyloxy)ethyl]-4-phenyl-3-vinylazetidin-2-one (8m)

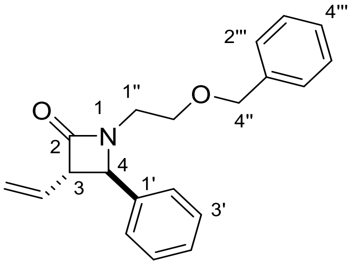

$C_{20}H_{21}NO_2$
M = 307,39 g/mol

Following **GP-3**, buta-2,3-dienamide **7m** (123 mg, 400 µmol, 1.00 eq.) was converted to β-lactam **8m** by irradiation for 20 hours. After column chromatography (SiO_2, 1 × 20 cm, P/Et_2O = 1/1) β-lactam **8m** (22.6 mg, 73.5 µmol, 18%) was obtained as a colorless oil.

TLC: R_f = 0.80 (P/Et_2O = 1/9) [$KMnO_4$].

^{1}H-NMR (500 MHz, DMSO-d_6, 298 K): δ [ppm] = 2.95–3.00 (m, 1H, C-1''-*H*H), 3.44–3.47 (m, 2H, C-3-H, C-2''-*H*H), 3.49–3.56 (m, 1H, C-1''-H*H*), 3.56–3.62 (m, 1H, C-2''-H*H*), 4.36–4.43 (m, 2H, C-4''-H_2), 4.49 (d, ^3J = 2.3 Hz, C-4-H), 5.20 (ddd, ^3J = 10.4 Hz, ^2J = 1.7 Hz, ^4J = 1.0 Hz, 1H, C-3-CH=CHH_Z), 5.26 (ddd, ^3J = 17.1 Hz, ^2J = 1.7 Hz, ^4J = 1.7 Hz, 1H, C-3-CH=CHH_E), 5.95 (ddd, ^3J = 17.1 Hz, ^3J = 10.4 Hz, ^3J = 8.0 Hz, 1H, C-3-C*H*=CH_2), 7.27–7.40 (m, 10H, H_{Ar}).

^{13}C-NMR (126 MHz, DMSO-d_6, 298 K): δ [ppm] = 40.4 (t, C-1''), 60.5 (d, C-4), 63.5 (t, C-2''), 66.7 (d, C-3), 118.9 (t, C-3-CH=*C*H_2), 126.5 (d, 2C, 2 × C-2')*, 127.55 (d, C-4')**, 127.63 (d, 2C, 2 × C-2'')*, 128.30 (d, 2C, 2 × C-3')***, 128.31 (d, C-4'')**, 128.9 (d, 2C, 2 × C-3'')***, 138.0 (s, C-1')****, 138.2 (s, C-1'')****, 167.3 (s, C-1).

IR (ATR): $\tilde{\nu}$ [cm^{-1}] = 3492 (w), 3030 (w), 2860 (w), 1748 (s, C=O), 1639 (w), 1496 (w), 1454 (w), 1395 (w), 1357 (w), 1274 (w), 1206 (w), 1102 (m), 1027 (w), 990 (w), 924 (w), 850 (w), 738 (m), 698 (s), 660 (w).

HRMS (ESI): [M+H$^+$]: calc.: 308.1645; found: 308.1631.

^{1}H,^{1}H-NOESY-spectrum:

NOE-contact between C-4-H and C-3-C*H*=CH$_2$.

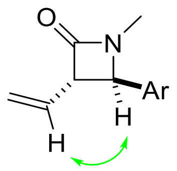

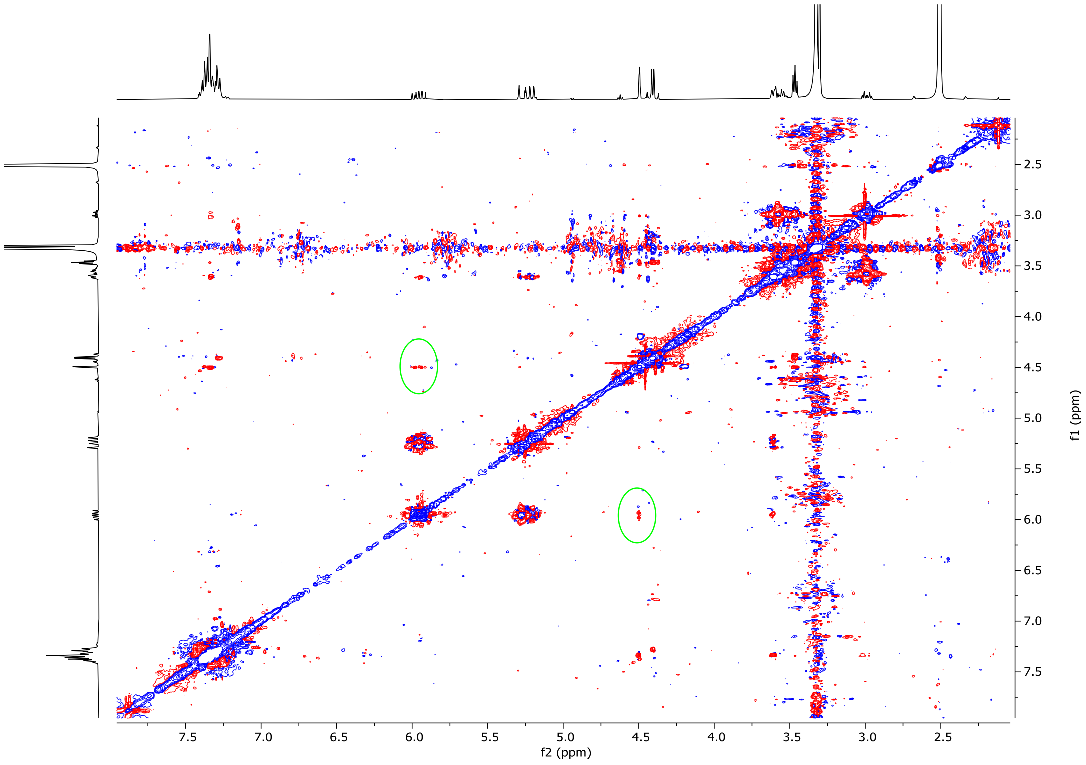

***rac*-1-Isopropyl-4-phenyl-3-vinylazetidin-2-one (8k) and *rac*-1-benzyl-4,4-dimethyl-3-vinylazetidin-2-one (9a)**

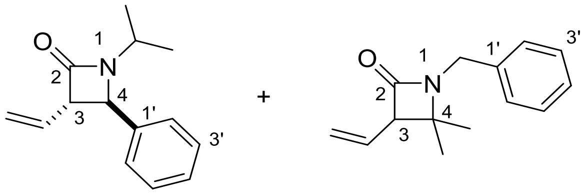

8k **9a**

$C_{14}H_{17}NO$
M = 215,30 g/mol

Following **GP-3**, buta-2,3-dienamide **7k** (82.1 mg, 400 µmol, 1.00 eq.) was converted to β-lactam **8k** and **9a** by irradiation for eight hours. After column chromatography (SiO_2, 1 × 20 cm, P/Et_2O = 9/1) β-lactam **8k** (16.4 mg, 76.0 µmol, 19%) and β-lactam **9a** (12.0 mg, 56.0 µmol, 14%) were obtained as colorless oils.

8k:

TLC: R_f = 0.54 (P/Et_2O = 1/1) [$KMnO_4$].

^{1}H-NMR (400 MHz, DMSO-d_6, 298 K): δ [ppm] = 0.95 [d, ^3J = 6.7 Hz, 3H, N-CH(CH_3)$_2$], 1.18 [d, ^3J = 6.7 Hz, 3H, N-CH(CH_3)$_2$], 3.52–3.63 [m, 2H, C-3-H, N-C*H*(CH$_3$)$_2$], 4.51 (d, ^3J = 2.3 Hz, 1H, C-4-H), 5.18 (ddd, ^3J = 10.4 Hz, ^2J = 1.8 Hz, ^4J = 1.0 Hz, 1H, C-3-CH=CHH_Z), 5.25 (*virt.* dt, ^3J = 17.1 Hz, $^2J \approx {}^4J$ = 1.5 Hz, 1H, C-3-CH=CHH_E), 5.95 (ddd, ^3J = 17.1 Hz, ^3J = 10.4 Hz, ^3J = 8.0 Hz, 1H, C-3-C*H*=CH$_2$), 7.31–7.36 (m, 1H, C-4'-H), 7.37–7.44 (m, 4H, 2 × C-2'-H, 2 × C-3'-H).

^{13}C-NMR (101 MHz, DMSO-d_6, 298 K): δ [ppm] = 20.1 [q, N-CH(*C*H$_3$)$_2$], 20.9 [q, N-CH(*C*H$_3$)$_2$], 44.4 [d, N-*C*H(CH$_3$)$_2$], 58.6 (d, C-4), 62.4 (d, C-3), 118.5 (t, C-3-CH=*C*H$_2$), 126.6 (d, 2C, 2 × C-2'), 128.2 (d, C-4'), 128.7 (d, 2C, 2 × C-3'), 132.1 (d, C-3-*C*H=CH$_2$), 139.2 (s, C-1'), 166.8 (s, C-2).

IR (ATR): $\tilde{\nu}$ [cm^{-1}] = 3086 (w), 3065 (w), 3032 (w), 2973 (w), 2933 (w), 2876 (w), 1742 (s), 1456 (m), 1381 (m), 1366 (m), 1334 (m), 923 (m), 747 (m), 700 (s).

HRMS (ESI): [M+H$^+$]: calc.: 216.1383; found: 216.1383.

9a:

TLC: R_f = 0.31 (P/Et_2O = 1/1) [$KMnO_4$].

^{1}H-NMR (400 MHz, DMSO-d_6, 298 K): δ [ppm] = 1.06 (s, 3H, C-4-CH_3), 1.23 (s, 3H, C-4-CH_3), 3.55 (d, ^{3}J = 7.8 Hz, 1H, C-3), 4.24–4.27 (m, 2H, N-CH_2-Ph), 5.22 (ddd, ^{3}J = 10.3 Hz, ^{2}J = 2.1 Hz, ^{4}J = 1.0 Hz, 1H, C-3-CH=CHH_Z), 5.28 (ddd, ^{3}J = 17.2 Hz, ^{2}J = 2.1 Hz, ^{4}J = 1.3 Hz, 1H, C-3-CH=CHH_E), 5.85 (ddd, ^{3}J = 17.2 Hz, ^{3}J = 10.3 Hz, ^{3}J = 7.8 Hz, 1H, C-3-C*H*=CH_2), 7.23–7.40 (m, 5H, H_{Ar}).

^{13}C-NMR (101 MHz, DMSO-d_6, 298 K): δ [ppm] = 22.0 (q, C-4-*C*H_3), 26.0 (q, C-4-*C*H_3), 42.5 (t, N-CH_2-Ph), 60.0 (s, C-4), 63.4 (d, C-3), 119.9 (t, C-3-CH=*C*H_2), 127.7 (d, C-4’), 128.4 (d, 2C, 2 × C-2’), 128.9 (d, 2C, 2 × C-3’), 131.5 (d, C-3-*C*H=CH_2), 138.2 (s, C-1’), 166.6 (s, C-2).

IR (ATR): $\tilde{\nu}$ [cm^{-1}] = 3089 (w), 3066 (w), 3033 (w), 3017 (w), 2975 (w), 2920 (w), 2870 (w), 1722 (s, C=O), 1393 (s), 1347 (s), 934 (s), 751 (s), 702 (s).

HRMS (ESI): [$M+H^{+}$]: calc.: 216.1383; found: 216.1383.

1-Allyl-4,5-dihydro-5,7a-ethenoindol-2(1*H*)-one (10)

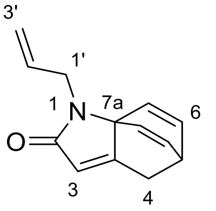

$C_{13}H_{13}NO$
M = 199,25 g/mol

Following **GP-3**, buta-2,3-dienamide **7n** (80.1 mg, 402 µmol, 1.00 eq.) was converted to 1-allyl-4,5-dihydro-5,7a-ethenoindol-2(1*H*)-one (**10**) by irradiation for 16 hours. After column chromatography (SiO_2, 1 × 20 cm, P/Et_2O = 4/6) **10** (17.3 mg, 85.5 µmol, 21%) was obtained as a yellow oil.

TLC: R_f = 0.13 (P/Et_2O = 1/1) [UV, $KMnO_4$].

^{1}H-NMR (500 MHz, $CDCl_3$, 298 K): δ [ppm] = 2.28 (t, ^3J = 2.2 Hz, 2H, C-4-H_2), 4.04–4.09 (m, 1H, C-5-H), 4.28 (d, ^3J = 6.1 Hz, 2H, C-1'-H_2), 5.21 (dd, ^3J = 10.1 Hz, ^4J = 1.5 Hz, 1H, C-3'-HH_Z), 5.21 (dd, ^3J = 16.8 Hz, ^4J = 1.5 Hz, 1H, C-3'-HH_E), 5.81 (s, 1H, C-3-H), 6.00 (ddt, ^3J = 16.8 Hz, ^3J = 10.1 Hz, ^3J = 6.1 Hz, 1H, C-2'-H), 6.29 (dd, ^3J = 7.2 Hz, ^4J = 1.6 Hz, 2H, 2 × C-7-H), 6.44 (dd, ^3J = 7.2 Hz, ^3J = 6.1 Hz, 2H, 2 × C-6-H).

^{13}C-NMR (126 MHz, $CDCl_3$, 298 K): δ [ppm] = 30.5 (d, C-5), 39.4 (t, C-4), 44.3 (t, C-1'), 75.3 (s, C-7a), 114.5 (d, C-3), 118.0 (t, C-3'), 130.8 (d, 2C, 2 × C-6), 133.8 (d, C-2'), 134.2 (d, 2C, 2 × C-7), 161.3 (s, C-3a), 173.9 (s, C-2).

Analytical data matched those previously reported in the literature.[17]

***rac*-1-Methyl-4,4-diphenyl-3-vinylazetidin-2-one (9b)**

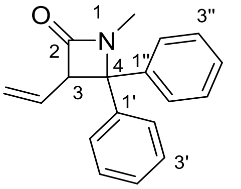

$C_{18}H_{17}NO$
M = 263,34 g/mol

Following **GP-3**, buta-2,3-dienamide **13b** (105 mg, 400 µmol, 1.00 eq.) was converted to β-lactam **9b** by irradiation for 24 hours. After column chromatography (SiO_2, 1 × 20 cm, P/Et_2O = 8/2) β-lactam **9b** (52.6 mg, 200 µmol, 50%) was obtained as a white solid.

TLC: R_f = 0.38 (P/Et_2O = 1/1) [UV, $KMnO_4$].

M.p.: 89°C.

^{1}H-NMR (400 MHz, DMSO-d_6, 298 K): δ [ppm] = 2.84 (bs, 3H, N-CH_3), 4.62–4.68 (m, 1H, C-3-H), 5.00 (ddd, ^3J = 10.2 Hz, ^2J = 2.2 Hz, ^4J = 0.8 Hz, 1H, C-3-CH=CHH_Z), 5.14 (ddd, ^3J = 17.0 Hz, ^3J = 10.2 Hz, ^3J = 8.1 Hz, 1H, C-3-CH=CH_2), 5.32 (ddd, ^3J = 17.0 Hz, ^2J = 2.2 Hz, ^4J = 1.0 Hz, 1H, C-3-CH=CHH_E), 6.97–7.03 (m, 2H, 2 × C-2'-H), 7.28–7.34 (m, 1H, C-4''-H)*, 7.35–7.48 (m, 7H, 2 × C-3'-H, C-4'-H*, 2 × C-2''-H, 2 × C-3''-H).

^{13}C-NMR (101 MHz, DMSO-d_6, 298 K): δ [ppm] = 26.3 (q, N-CH_3), 64.1 (d, C-3), 70.3 (s, C-4), 119.9 (t, C-3-CH=CH_2), 127.40 (d, C-4'')*, 127.42 (d, 2C, 2 × C-2''), 127.6 (d, 2C, 2 × C-2'), 127.9 (d, C-4')*, 128.3 (d, 2C, 2 × C-3'), 128.6 (d, 2C, 2 × C-3''), 130.7 (d, C-3-CH=CH_2), 139.4 (s, C-1'), 140.3 (s, C-1''), 167.3 (s, C-2).

* signals are interchangeable

IR (ATR): $\tilde{\nu}$ [cm^{-1}] = 3074 (w), 3027 (w), 2969 (w), 2915 (w), 1743 (s, C=O), 1447 (s), 1382 (s), 990 (s), 926 (s), 753 (s), 727 (s), 697 (s), 675 (s).

HRMS (ESI): [M+H$^+$]: calc.: 264.1383; found: 264.1374.

rac-1-Isopropyl-4,4-dimethyl-3-vinylazetidin-2-one (9c)

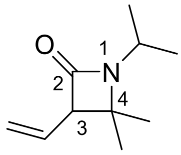

$C_{10}H_{17}NO$
M = 167,25 g/mol

Following **GP-3**, buta-2,3-dienamide **13c** (66.9 mg, 400 µmol, 1.00 eq.) was converted to β-lactam **9c** by irradiation for 18 hours. After column chromatography (SiO_2, 1 × 20 cm, P/Et_2O = 1/1 → 4/6) β-lactam **9c** (16.2 mg, 96.9 µmol, 24%) was obtained as a colorless oil.

TLC: R_f = 0.40 (P/Et_2O = 4/6) [$KMnO_4$].

^{1}H-NMR (500 MHz, DMSO-d_6, 298 K): δ [ppm] = 1.20 (s, 3H, C-4-CH_3), 1.21 (d, ^3J = 6.8 Hz, 3H, N-CH(CH_3)$_2$), 1.22 (d, ^3J = 6.8 Hz, 3H, N-CH(CH_3)$_2$), 1.37 (s, 3H, C-4-CH_3), 3.39 (*virt.* dt, ^3J = 8.0 Hz, $^4J \approx {}^4J$ = 1.0 Hz, 1H, C-3-H), 3.49 (hept, ^3J = 6.8 Hz, 1H, N-CH(CH_3)$_2$), 5.19 (ddd, ^3J = 10.3 Hz, ^2J = 2.2 Hz, ^4J = 1.1 Hz, 1H, C-3-CH=CHH_Z), 5.23 (ddd, ^3J = 17.2 Hz, ^2J = 2.2 Hz, ^4J = 1.3 Hz, 1H, C-3-CH=CHH_E), 5.82 (ddd, ^3J = 17.2 Hz, ^3J = 10.3 Hz, ^3J = 8.0 Hz, 1H, C-3-CH=CH_2).

^{13}C-NMR (126 MHz, DMSO-d_6, 298 K): δ [ppm] = 21.6 (q, N-CH(CH_3)$_2$), 21.7 (q, N-CH(CH_3)$_2$), 22.4 (q, C-4-CH_3), 26.4 (q, C-4-CH_3), 43.2 (d, N-CH(CH_3)$_2$), 59.3 (s, C-4), 62.6 (d, C-3), 119.4 (t, C-3-CH=CH_2), 131.4 (d, C-3-CH=CH_2), 165.2 (s, C-2).

IR (ATR): $\tilde{\nu}$ [cm^{-1}] = 2974 (m), 2935 (w), 2876 (w), 1730 (s, C=O), 1380 (s), 1371 (s), 1343 (s), 1248 (s), 923 (m).

HRMS (ESI): [M+H$^+$]: calc.: 168.1383; found: 168.1376.

rac-1-Cyclohexyl-3-vinyl-1-azaspiro[3.5]nonan-2-one (9d) and N-cyclohexylbut-3-enamide (16d)

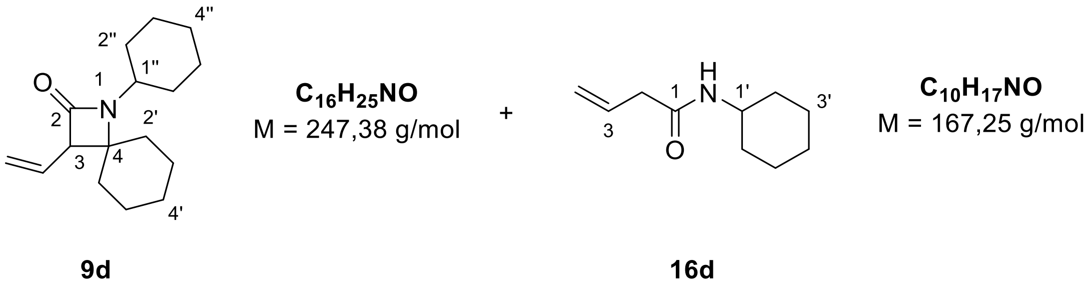

Following **GP-3**, buta-2,3-dienamide **13c** (99.0 mg, 400 µmol, 1.00 eq.) was converted to β-lactam **9d** by irradiation for 22 hours. After column chromatography (SiO_2, 1 × 20 cm, P/Et_2O = 7/3 → 6/4) β-lactam **9d** (31.4 mg, 127 µmol, 32%) and amide **16d** (13.3 mg, 80.0 µmol, 20%) were obtained as a yellow solid (**9d**) and yellow oil (**16d**).

9d:

TLC: R_f = 0.42 (P/Et_2O = 1/1) [$KMnO_4$].

M.p.: 80°C.

^{1}H-NMR (500 MHz, $CDCl_3$, 298 K): δ [ppm] = 1.06–1.38 (m, 6H, H_{alkyl}), 1.57–1.84 (m, 14H, H_{alkyl}), 3.01–3.12 (m, 1H, C-1''-H), 3.33 (d, ^3J = 8.9 Hz, 1H, C-3-H), 5.28 (*virt.* dd, ^3J = 10.3 Hz, $^2J \approx {}^4J$ = 1.6 Hz, 1H, C-3-CH=CHH_Z), 5.33 (d, ^3J = 17.1 Hz. 1H, C-3-CH=CHH_E), 5.85–5.94 (m, 1H, C-3-C*H*=CH_2).

^{13}C-NMR (126 MHz, $CDCl_3$, 298 K): δ [ppm] = 23.6 (t, C_{alykl}), 24.5 (t, C_{alykl}), 25.0 (t, C_{alykl}), 25.3 (t, C_{alykl}), 26.0 (t, C_{alykl}), 26.0 (t, C_{alykl}), 31.0 (t, C_{alykl}), 32.5 (t, C_{alykl}), 32.6 (t, C_{alykl}), 37.8 (t, C_{alykl}), 52.1 (d, C-1''), 62.9 (d, C-3), 63.6 (s, C4), 120.6 (t, C-3-CH=*C*H_2), 130.6 (d, C-3-*C*H=CH_2), 167.0 (s, C-2).

IR (ATR): $\tilde{\nu}$ [cm^{-1}] = 2929 (s), 2857 (s), 1725 (s, C=O), 1447 (s), 1373 (s), 1291 (m), 1171 (m), 1095 (m), 989 (s), 914 (s),

HRMS (ESI): [M+H$^+$]: calc.: 248.2009; found: 248.1999.

16d:

TLC: R_f = 0.15 (P/Et_2O = 1/1) [$KMnO_4$].

^{1}H-NMR (500 MHz, $CDCl_3$, 298 K): δ [ppm] = 1.05–1.20 (m, 3H, 2 × C-2'-H, C-3'-H), 1.28–1.40 (m, 2H, C-3'-H, C-4'-H), 1.56–1.63 (m, 1H, C-3'-H), 1.65–1.72 (m, 2H, C-4'-H, C-3'-H), 1.85–1.92 (m, 2H, 2 × C-2'-H), 2.98 (dt, ^3J = 7.1 Hz, ^4J = 1.3 Hz, 2H, C-2-H_2), 3.70–3.79 (m, 1H, C-1'-H), 5.18–5.25 (m, 2H, C-4-H_2), 5.57 (bs, 1H, NH), 5.91 (*virt.* ddt, ^3J = 17.2 Hz, ^3J = 10.2 Hz, $^3J \approx {}^3J$ = 7.1 Hz, 1H, C-3-H).

^{13}C-NMR (126 MHz, $CDCl_3$, 298 K): δ [ppm] = 24.9 (t, C-4'), 25.6 (t, 2C, 2 × C-3'), 33.2 (t, 2C, 2 × C-2'), 41.9 (t, C-2), 48.3 (d, C-1'), 119.9 (t, C-4), 131.7 (d, C-3), 169.8 (s, C-1).

Analytical data matched those previously reported in the literature.[18]

rac-1-benzyl-3-[(E)-3,3-dimethylbut-1-en-1-yl]-4-phenylazetidin-2-one (12)

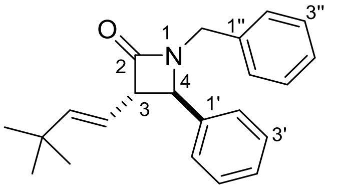

$C_{22}H_{25}NO$
M = 319,45 g/mol

Following **GP-3**, buta-2,3-dienamide **11** (112 mg, 400 µmol, 1.00 eq.)* was converted to β-lactam ***E*-12** by irradiation for two and a half hours. After column chromatography (SiO_2, 1 × 20 cm, P/Et_2O = 1/1 → 4/6) β-lactam ***E*-12** (48.3 mg, 151 µmol, 43%) was obtained as a colorless oil. ***Z*-12** (13.5 mg, 42.3 µmol, 12%) was isolated as a mixture with inseparable *N,N*-dibenzyl-5,5-dimethylhex-3-ynamide.

*a inseparable mixture of buta-2,3-dienamide **11** and *N,N*-dibenzyl-5,5-dimethylhex-3-ynamide was irradiated

***E*-12:**

TLC: R_f = 0.35 (P/Et_2O = 4/6) [UV, $KMnO_4$].

^{1}H-NMR (500 MHz, DMSO-d_6, 298 K): δ [ppm] = 0.98 (s, 9H, C-3-CH=CH-C(C*H*$_3$)$_3$), 3.63 (dd, ^3J = 8.2 Hz, ^3J = 2.3 Hz, 1H, C-3-H), 3.85 (d, ^2J = 15.6 Hz, 1H, N-C*H*H-Ph), 4.28 (d, ^3J = 2.3 Hz, 1H, C-4-H), 4.64 (d, ^2J = 15.6 Hz, 1H, N-CH*H*-Ph), 5.47 (dd, ^3J = 15.7 Hz, ^3J = 8.2 Hz, 1H, C-3-C*H*=CH-^{t}Bu), 5.47 (dd, ^3J = 15.7 Hz, ^4J = 1.0 Hz, 1H, C-3-CH=C*H*-^{t}Bu), 7.13–7.17 (m, 2H, 2 × C-2''-H), 7.24–7.39 (m, 8H, 2 × C-2'-H, 2 × C-3'-H, C-4'-H, 2 × C-3''-H, C-4''-H).

^{13}C-NMR (126 MHz, DMSO-d_6, 298 K): δ [ppm] = 29.3 (q, 3C, C-3-CH=CH-C(*C*H$_3$)$_3$), 33.0 (s, C-3-CH=CH-*C*(CH$_3$)$_3$), 43.9 (t, N-CH_2-Ph), 60.8 (d, C-4), 62.7 (d, C-3), 118.2 (d, C-3-*C*H=CH-^{t}Bu), 126.7 (d, 2C, 2 × C-2'), 127.4 (d, C-4''), 127.8 (d, 2C, 2 × C-2''), 128.3 (d, C-4'), 128.7 (d, 2C, 2 × C-3'), 128.9 (d, 2C, 2 × C-3''), 136.1 (s, C-1'), 137.5 (s, C-1''), 145.4 (d, C-3-CH=*C*H-^{t}Bu), 168.0 (s, C-2).

IR (ATR): $\tilde{\nu}$ [cm^{-1}] = 3031 (w), 2958 (m), 2905 (w), 2866 (w), 1750 (s, C=O), 1456 (m), 1391 (m), 1360 (m), 970 (m), 759 (m), 732 (m), 698 (s).

HRMS (ESI): [M+H$^+$]: calc.: 320.2009; found: 320.1998.

7.3 Reactions of β-Lactam 8a

***rac*-1-Benzyl-4-phenyl-3-[2'-(4,4,5,5-tetramethyl-1,3,2-dioxaborolan-2-yl)ethyl]azetidin-2-one (18)**

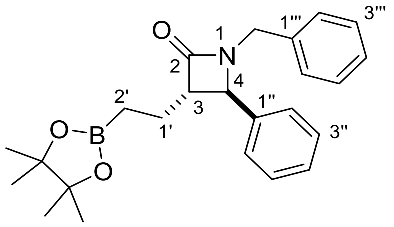

$C_{24}H_{30}BNO_3$
M = 391,32 g/mol

Following a modified procedure,[19] to a solution of bis(1,5-cyclooctadiene)diiridium(I) dichloride (8.10 mg, 12.0 µmol, 4 mol%) and (ethane-1,2-diyl)bis(diphenyl phosphane) (12.0 mg, 30.0 µmol, 10 mol%) in CH_2Cl_2 (2 mL) was added 4,4,5,5-tetramethyl-1,3,2-dioxaborolane (100 µL, 670 µmol, 2.25 eq.) slowly at room temperature. The solution was stirred for ten minutes at room temperature. Then a solution of β-lactam **8a** (79.0 mg, 300 µmol, 1.00 eq.) in CH_2Cl_2 (2 mL) was added slowly. After stirring for 16 hours at room temperature, MeOH (2 mL) and H_2O (10 mL) were added at 0°C to stop the reaction. After warming up to room temperature the phases were separated and the aq. solution extracted with CH_2Cl_2 (2 × 10 mL), the combined organic layers dried over Na_2SO_4, filtered and the solvent removed under reduced pressure. After column chromatography (SiO_2, 2 × 20 cm, EtOAc/Hex = 1/9) the crude was diluted in EtOAc (10 mL) and washed with a basic solution of D-mannitol and Na_2CO_3 (1 M, 10 mL) to remove the boronic acid. After removing the solvent under reduced pressure, β-lactam **18** (62.2 mg, 159 µmol, 53%) was obtained as a colorless oil.

TLC: R_f = 0.23 (EtOAc/Hex = 2/8) [$KMnO_4$].

^{1}H-NMR (500 MHz, DMSO-d_6, 298 K): δ [ppm] = 0.76–0.81 (m, 2H, C-2'-H_2), 1.08 (s, 6H, $C(CH_3)_2$-$C(CH_3)_2$), 1.09 (s, 6H, C(CH_3)2-$C(CH_3)_2$), 1.62–1.78 (m, 2H, C-1'-H_2), 2.95 (ddd, ^3J = 8.5 Hz, ^3J = 6.0 Hz, ^3J = 2.1 Hz, 1H, C-3-H), 3.78 (d, ^2J = 15.5 Hz, 1H, N-C*H*H-Ph), 4.19 (d, ^3J = 2.1 Hz, 1H, C-4-H), 4.66 (d, ^2J = 15.5 Hz, 1H, N-CH*H*-Ph), 7.12–7.16 (m, 2H, H_{Ar}), 7.24–7.39 (m, 8H, H_{Ar}).

^{13}C-NMR (126 MHz, DMSO-d_6, 298 K): δ [ppm] = 8.11 (t, C-2'), 22.8 (t, C-1'), 24.55 (q, 2C, 2 × CH_3), 24.61 (q, 2C, 2 × CH_3), 43.7 (t, N-*C*H_2-Ph), 59.4 (d, C-3), 61.7 (d, C-4), 82.8 (s, 2C, *C*$(CH_3)_2$-*C*$(CH_3)_2$), 126.4 (d, 2C, 2 × C-2''), 127.4 (d, C-4'''), 127.9 (d, 2C, 2 × C-2'''),

128.0 (d, C-4’’), 128.7 (d, 2C, 2 × C-3’’), 128.8 (d, 2C, 2 × C-3’’’), 136.3 (s, C-1’’), 138.4 (s, C-1’’’), 169.5 (s, C-2).

IR (ATR): $\tilde{\nu}$ [cm^{-1}] = 3485 (bw), 3064 (w), 3031 (w), 2978 (w), 2932 (w), 1747 (s, C=O), 1371 (s), 1322 (s), 1143 (s), 846 (m), 699 (s).

HRMS (ESI): [$M+H^+$]: calc.: 392.2392; found: 392.2385.

***rac*-1-Benzyl-3-(2'-hydroxyethyl)-4-phenylazetidin-2-one (19)**

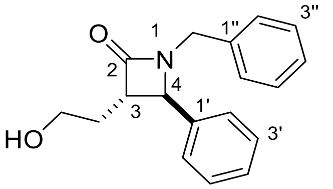

$C_{18}H_{19}NO_2$
M = 281,36 g/mol

Following a literature-known procedure,[20] to a solution of β-lactam **18** (36.0 mg, 92.0 µmol, 1.00 eq.) in THF (1 mL) at 0°C was added aq. NaOH-solution (3 M, 400 µL) and aq. H_2O_2-solution (35%, 200 µL). The solution was allowed to reach room temperature and stirred for 40 minutes. After addition of H_2O (5 mL) the phases were separated and the aq. solution extracted with EtOAc (3 × 5 mL). The combined organic layers were dried over Na_2SO_4, filtered and the solvents removed under reduced pressure. After column chromatography (SiO_2, 0.5 × 10 cm, P/Et_2O = 1/9) alcohol **19** (20.3 mg, 72.2 µmol, 78%) was obtained as colorless oil.

TLC: R_f = 0.23 (P/Et_2O = 1/9) [$KMnO_4$].

^{1}H-NMR (500 MHz, DMSO-d_6, 298 K): δ [ppm] = 1.75–1.90 (m, 2H, C-3-CH_2-CH_2-OH), 3.03 (ddd, ^3J = 8.5 Hz, ^3J = 5.9 Hz, ^3J = 2.1 Hz, 1H, C-3-H), 3.44–3.49 (m, 2H, C-3-CH_2-CH_2-OH), 3.79 (d, ^2J = 15.5 Hz, 1H, N-C*H*H-Ph), 4.25 (d, ^3J = 2.1 Hz, 1H, C-4-H), 4.53 (t, ^3J = 5.0 Hz, 1H, OH), 4.67 (d, ^2J = 15.5 Hz, 1H, N-CH*H*-Ph), 7.13–7.17 (m, 2H, H_{Ar}), 7.25–7.38 (m, 8H, H_{Ar}).

^{13}C-NMR (126 MHz, DMSO-d_6, 298 K): δ [ppm] = 31.7 (t, C-3-*C*H_2-CH_2-OH), 43.8 (t, N-*C*H_2-Ph), 57.4 (d, C-3), 58.7 (t, C-3-CH_2-*C*H_2-OH), 60.1 (d, C-4), 126.5 (d, 2C, 2 × C-2'), 127.4 (d, C-4''), 127.8 (d, 2C, 2 × C-2''), 128.1 (d, C-4'), 128.7 (d, 2C, 2 × C-3'), 128.8 (d, 2C, 2 × C-3''), 136.3 (s, C-1'), 138.3 (s, C-1''), 169.7 (s, C-2).

IR (ATR): $\tilde{\nu}$ [cm^{-1}] = 3421 (bm, OH), 3064 (w), 3031 (w), 2917 (bw), 1724 (s, C=O), 1456 (m), 1402 (m), 1355 (m), 1052 (s), 698 (s).

HRMS (ESI): [M+H^+]: calc.: 282.1489; found: 282.1482.

***rac*-2'-(-1-benzyl-2-oxo-4-phenylazetidin-3-yl)acetaldehyde (20)**

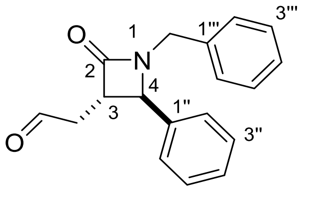

$C_{18}H_{17}NO_2$
M = 279,34 g/mol

Following a modified procedure,[21] a suspension of $PdCl_2$ (7.09 mg, 40.0 µmol, 20 mol%) and CuCl (19.8 mg, 200 µmol, 1.00 eq.) in DMF (4 mL) and water (800 µL) was degassed with oxygen-gas for 15 minutes. The suspension was stirred for one hour. Afterwards a solution of *β*-lactam **8a** (52.7 mg, 200 µmol, 1.00 eq.) in DMF (1.6 mL) was added to the suspension and the reaction mixture stirred for 20 hours at room temperature under oxygen-atmosphere. The reaction was stopped by the addition of EtOAc (200 mL) and the layers separated. The organic layer was washed with water (3 × 20 mL), NaCl-solution (20 mL) dried over Na_2SO_4, filtered and the solvents removed under reduced pressure. After column chromatography (SiO_2, 0.5 × 15 cm, P/Et_2O = 2/8) aldehyde **20** (35.9 mg, 129 µmol, 68%) was obtained as colorless oil.

TLC: R_f = 0.46 (Et_2O) [$KMnO_4$].

^{1}H-NMR (500 MHz, $CDCl_3$, 298 K): δ [ppm] = 2.80 (ddd, ^2J = 18.4 Hz, ^3J = 9.7 Hz, ^3J = 1.2 Hz, 1H, C-3-CH*H*-CHO), 3.05 (ddd, ^2J = 18.4 Hz, ^3J = 4.7 Hz, ^3J = 1.0 Hz, 1H, C-3-C*H*H-CHO), 3.37 (dddd, ^3J = 9.7 Hz, ^3J = 4.7 Hz, ^3J = 2.2 Hz, ^5J = 0.9 Hz, 1H, C-3-H), 3.77 (dd, ^2J = 14.9 Hz, ^5J = 1.0 Hz, 1H, N-CH*H*-Ph), 4.08 (d, ^3J = 2.2 Hz, 1H, C-4-H), 4.84 (d, ^2J = 14.9 Hz, 1H, N-C*H*H-Ph), 7.11–7.16 (m, 2H, 2 × C-2'''-H), 7.22–7.41 (m, 8H, 2 × C-2''-H, 2 × C-3''-H, 2 × C-3'''-H, C-4''-H, C-4'''-H), 9.75 (t, ^3J = 1.0 Hz, 1H, CHO).

^{13}C-NMR (126 MHz, $CDCl_3$, 298 K): δ [ppm] = 42.3 (t, C-3-*C*H_2-CHO), 44.6 (t, N-*C*H_2-Ph), 53.9 (d, C-3), 60.5 (d, C-4), 126.6 (d, 2C, 2 × C-2''), 127.9 (d, C-4'''), 128.6 (d, 2C, 2 × C-2'''), 128.7 (d, C-4''), 128.9 (d, 2C, 2 × C-3'''), 129.1 (d, 2C, 2 × C-3''), 135.4 (s, C-1'''), 137.0 (s, C-1''), 168.6 (s, C-2). 198.9 (d, CHO).

IR (ATR): $\tilde{\nu}$ [cm^{-1}] = 3425 (bw), 3088 (w), 3064 (w), 3032 (w), 2917 (w), 2835 (w), 2728 (w), 1742 (s, C=O), 1720 (s, C=O), 1456 (m), 1395 (m), 752 (m), 698 (s).

HRMS (ESI): [M+H$^+$]: calc.: 280.1332; found: 280.1321.

***rac*-1-benzyl-3-ethyl-4-phenylazetidin-2-one (21)**

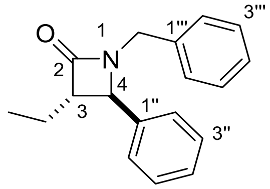

$C_{18}H_{19}NO$
M = 265,36 g/mol

Following a modified procedure,[22] to a solution of *β*-lactam **8a** (37.8 mg, 144 µmol, 1.00 eq.) in EtOAc (4 mL) was added Pd/C (5 wt.%, 10 mg, 25% w/w) and the suspension degassed three times with H_2 by *freeze-pump-thaw*. Afterwards the suspension was stirred for 20 hours at room temperature. The reaction mixture was filtered over a pad of celite, washed with EtOAc and the solvents removed under reduced pressure. *β*-lactam **21** (38.2 mg, 144 µmol, quant.) was obtained as colorless oil.

TLC: R_f = 0.37 (Et_2O/P = 3/7) [$KMnO_4$].

^{1}H-NMR (400 MHz, $CDCl_3$, 298 K): *δ* [ppm] = 0.99 (t, ^3J = 7.4 Hz, 3H, C-3-CH2C*H*3), 1.66–1.91 (m, 2H, C-3-C*H*2CH3), 3.00 (ddd, ^3J = 8.3 Hz, *J* = 5.9 Hz, ^3J = 2.1 Hz, 1H, C-3-H), 3.73 (d, ^2J = 14.9 Hz, 1H, N-C*H*H-Ph), 4.05 (d, ^3J = 2.1 Hz, 1H, C-4-H), 4.86 (d, ^2J = 14.9 Hz, 1H, N-CH*H*-Ph), 7.12–7.16 (m, 2H, 2 × C-2'''-H), 7.21–7.40 (m, 8H, 2 × C-2''-H, 2 × C-3''-H, 2 × C-3'''-H, C-4''-H, C-4'''-H).

^{13}C-NMR (101 MHz, $CDCl_3$, 298 K): *δ* [ppm] = 11.5 (q, C-3-CH2*C*H3), 21.8 (t, C-3-*C*H2CH3), 44.4 (t, N-*C*H2-Ph), 60.1 (d, C-4), 62.1 (d, C-3), 126.6 (d, 2C, 2 × C-2'''), 127.7 (d, C-4'''), 128.4 (d, C-4''), 128.6 (d, 2C, 2 × C-2'''), 128.8 (d, 2C, 2 × C-3''), 129.1 (d, 2C, 2 × C-3'''), 135.9 (s, C-1'''), 138.2 (s, C-1''), 170.5 (s, C-2).

IR (ATR): $\tilde{\nu}$ [cm^{-1}] = 3064 (w), 3031 (w), 2963 (w), 2933 (w), 2876 (w), 1748 (s, C=O), 1456 (m), 700 (m).

HRMS (ESI): [M+H$^+$]: calc.: 266.1539; found: 266.1536.

8. Deuteration Experiments

8.1 Deuteration Experiment with 7f-d_2

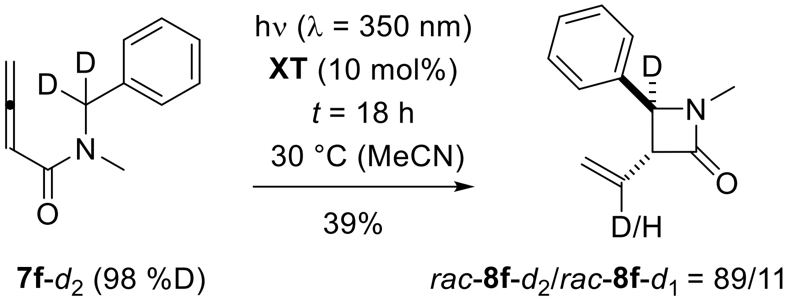

Following **GP-3**, buta-2,3-dienamide **7f-*d*₂** (75.7 mg, 400 µmol, 1.00 eq., 98%D) was converted to β-lactam **8f-d_2** by irradiation for 18 hours. After column chromatography (SiO_2, 1 × 20 cm, P/Et_2O = 7/3 → 1/1) β-lactam **8f-d_2** (29.5 mg, 156 µmol, 39%, **8f-d_2/8f-d_1** = 89/11) was obtained as a colorless oil.

^{1}H-spectrum of **8f-d_2/8f-d_1:**

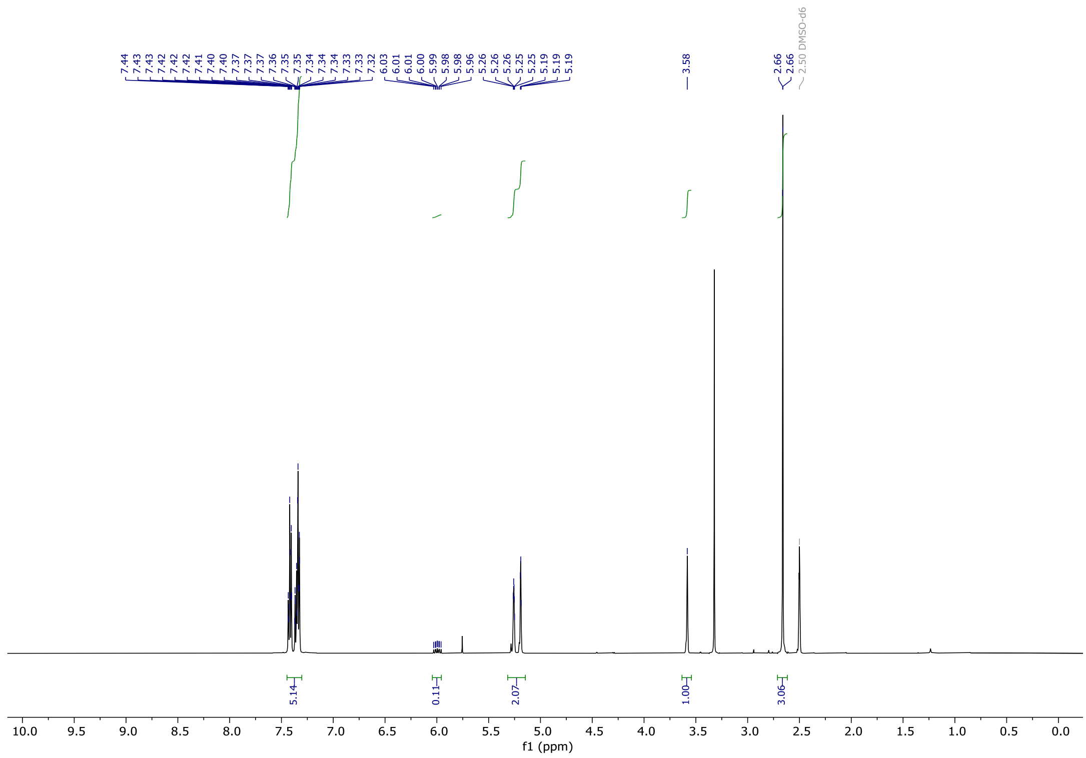

8.2 Deuteration Experiment with 7f-d_1

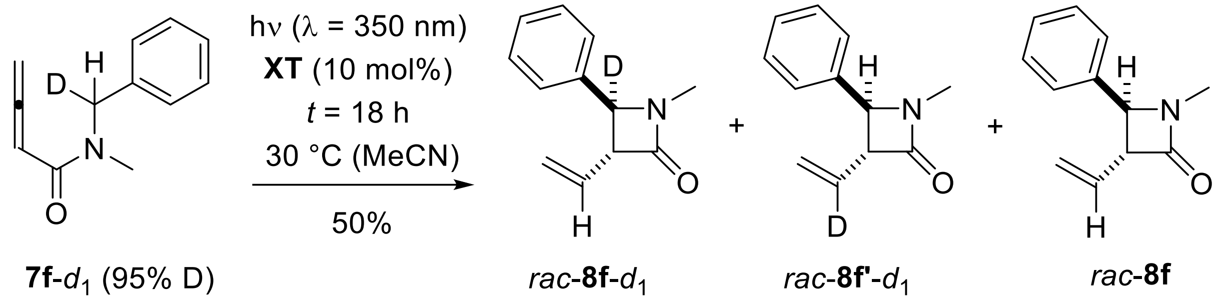

Following **GP-3**, buta-2,3-dienamide **7f-*d*1** (75.3 mg, 400 µmol, 1.00 eq., 95%D) was converted to β-lactam **8f-*d*1**/**8f'-*d*1** by irradiation for 18 hours. After column chromatography (SiO_2, 1 × 20 cm, P/Et_2O = 7/3 → 1/1) β-lactam **8f-*d*1**/**8f'-*d*1** (37.6 mg, 200 µmol, 50%, r.r. = **8f/8f'** = 3.43/1.00) was obtained as a colorless oil.

$$\text{Primary kinetic isotope effect: } \frac{\mathbf{8}f}{\mathbf{8}f'} = \frac{77}{23} = 3.43.$$

^{1}H-spectrum of **8f-*d*1/8f'-*d*1/8f:**

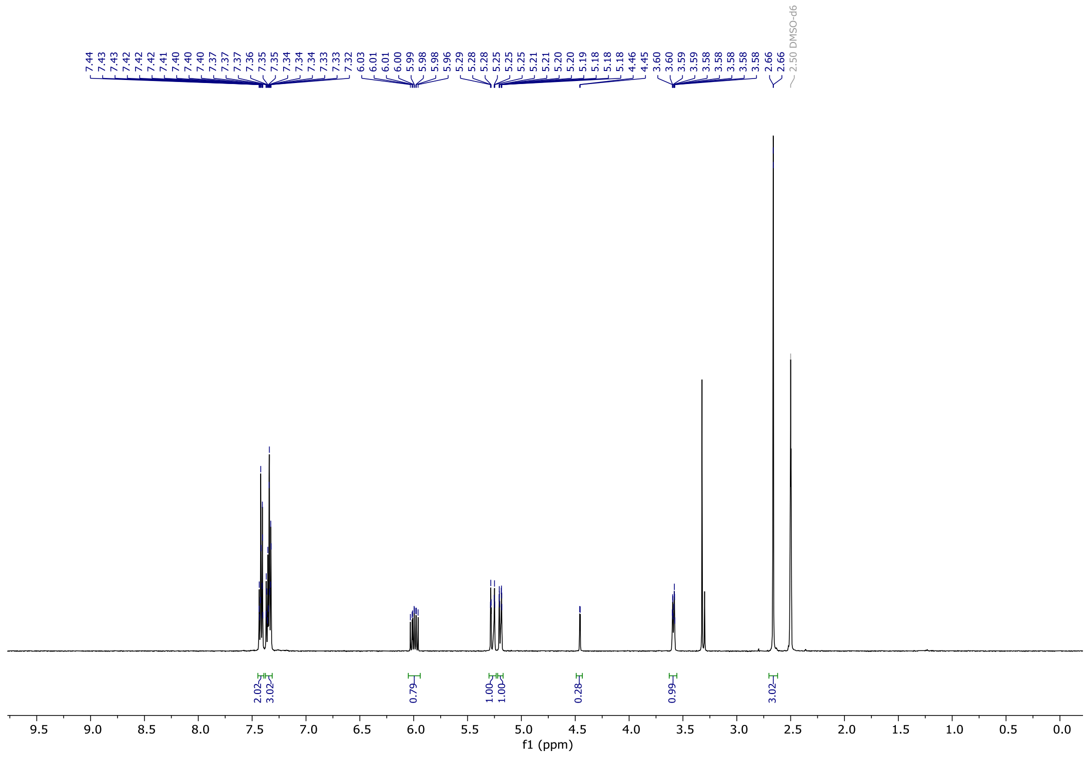

8.3 Photoreaction of 7a to 8a in Deuterated Solvent

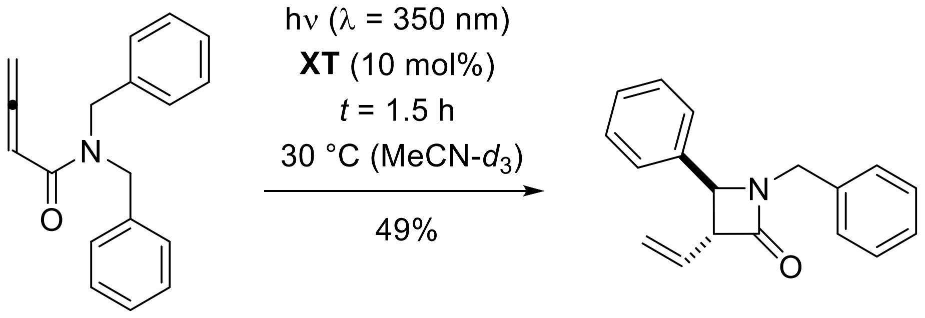

7a **8a** (0% D)

No incorporation of deuterium from the solvent (MeCN-d_3) was observed during the photocyclization of **7a** to **8a**.

9. Crude NMR of the Photoreaction

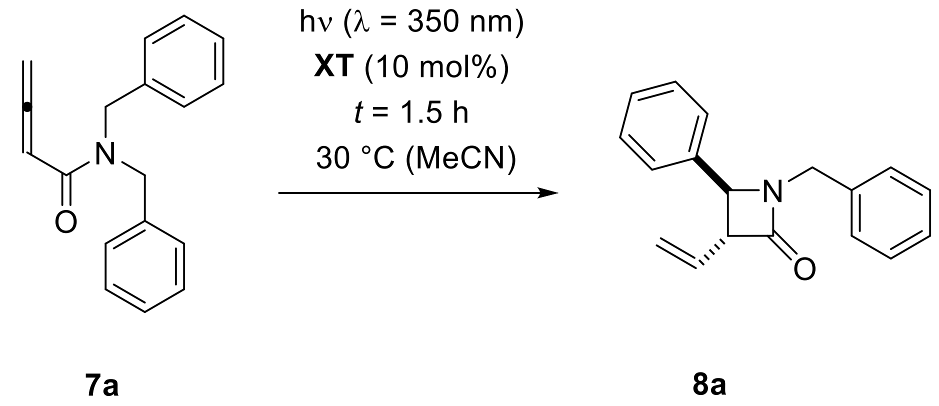

The solvent (MeCN) of the reaction solution was removed under reduced pressure and the resulting crude material measured in $CDCl_3$. The crude ^{1}H-NMR spectrum shows only signals of *anti*-**8a** as the photoproduct.

^{1}H-NMR (500 MHz, $CDCl_3$, 298 K):

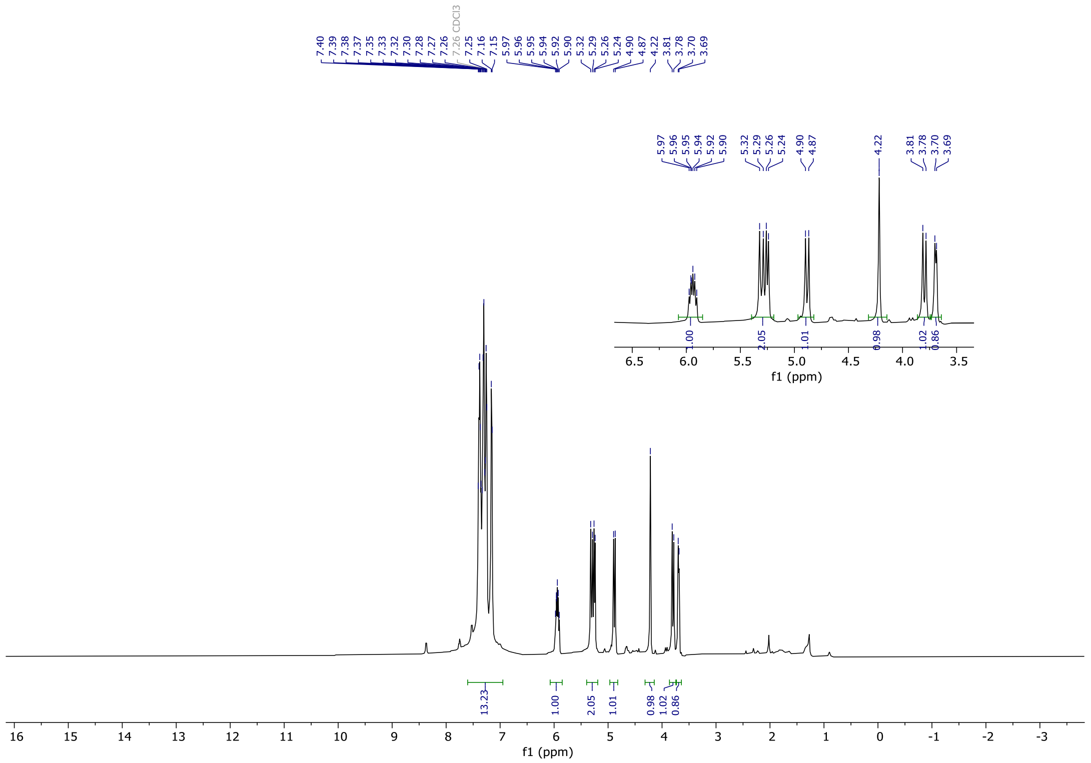

10. Stability of Photoproduct 8a under Irradiation Conditions

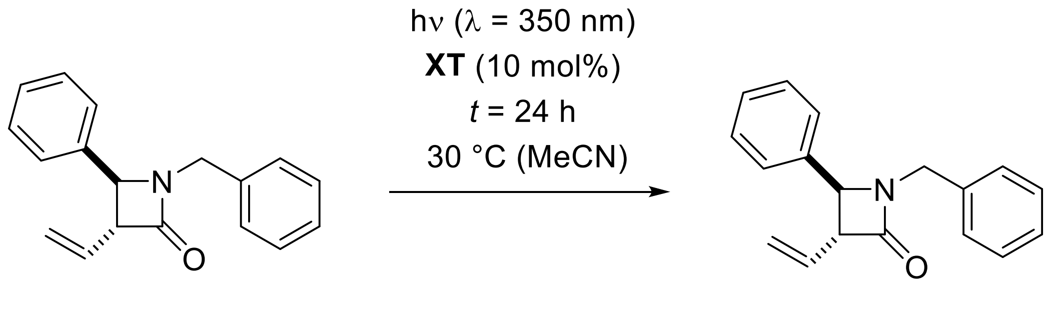

8a **8a** (68%)

Photoproduct **8a** (39.5 mg, 150 µmol, 1.00 eq.) was exposed to the irradiation conditions (GP-3) under which the photoreaction from amides **7** to β-lactams **8** were routinely performed. After an irradiation time of 24 hours only 68% of the photoproduct (27.0 mg, 102 µmol, 68%) was re-isolated chromatographically. TLC of the crude reaction mixture showed product **8a** and a spot of undefined degradation at the baseline.

11. Transient Absorption Experiments

11.1 Triplet Excited State of Xanthone:

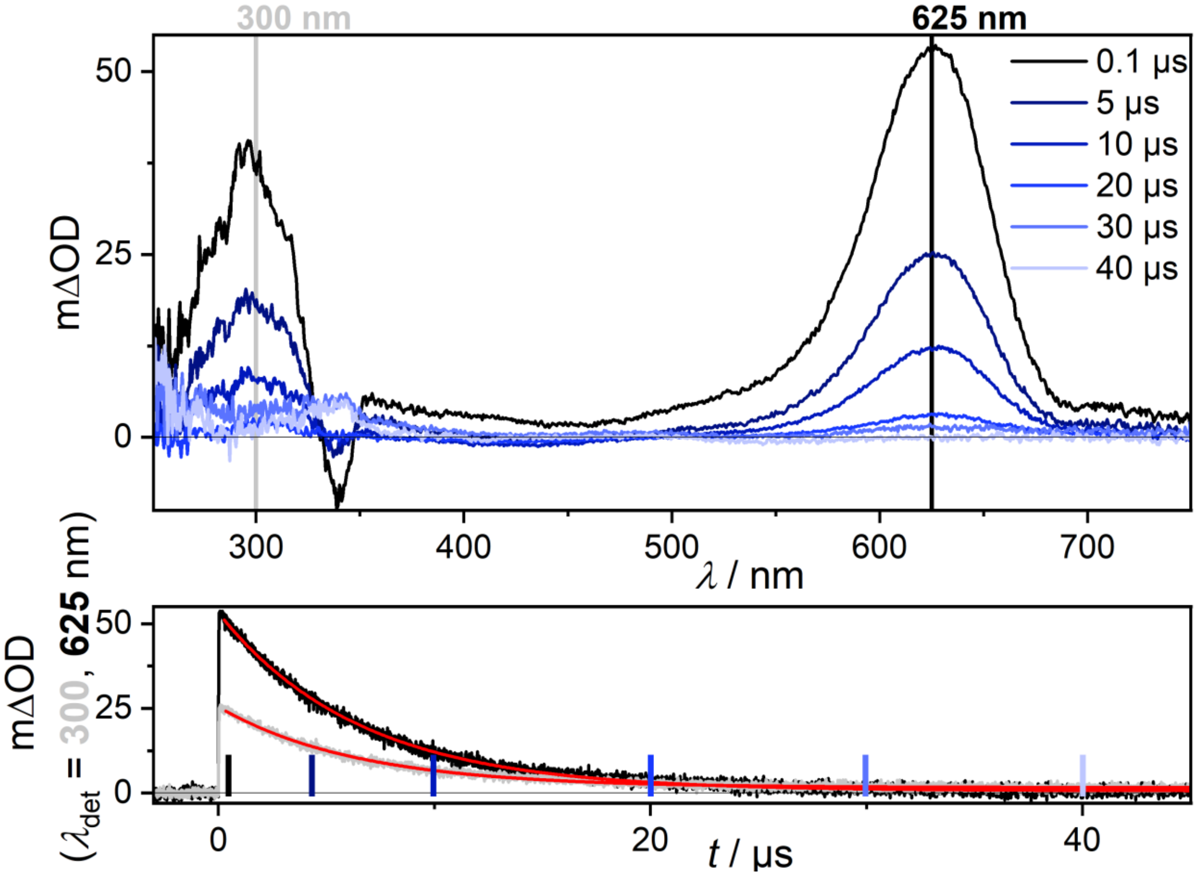

Figure S 1. TA data for ^{3}XT using a stock solution of XT (63 µM) in argon-saturated acetonitrile and a 355 nm laser (25.7 mJ) for excitation. Upper panel: Spectrum of ^{3}XT at different delay times. Lower panel: Kinetic decay traces detected at two different detection wavelengths (gray data 300 nm, black data 625 nm). The blue lines indicate the delay times that were chosen for the detection of the TA spectra (same color code). The results of the mono-exponential fits are shown in red.

Both the spectrum and lifetime of the ^{3}XT are in good agreement with literature data[23] (Figure S 1). The ^{3}XT shows two bands with maxima centered around 300 nm and 625 nm. The lifetime is highly dependent on the conditions such as the chosen solvent, oxygen concentration and laser intensity, and our measured lifetime in Figure S 1 exceeds that reported in the literature in acetonitrile solution [τ(625nm) = 6.6µs, τ(300nm) = 6.4µs][3,23,24].

11.2 HAT Reactivity of Xanthone:

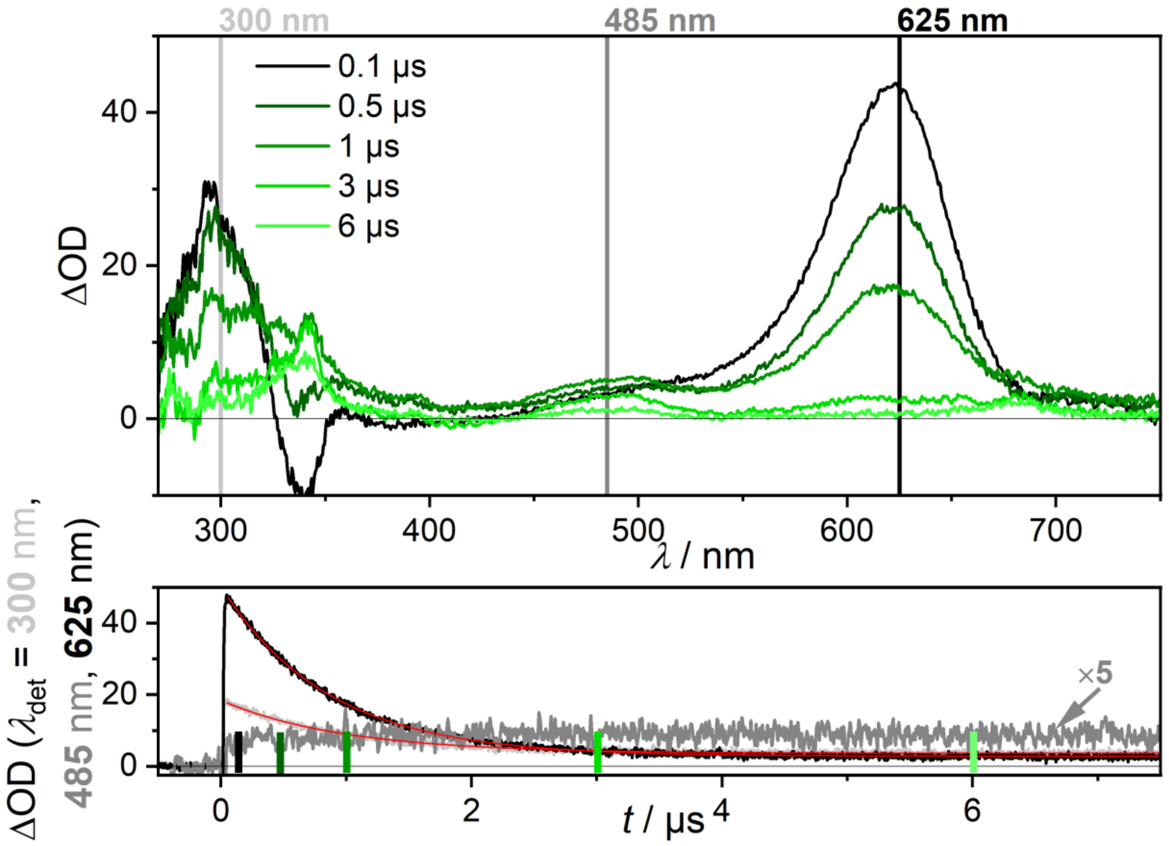

Figure S 2. TA data for the quenching of ^{3}XT followed by a HAT using a stock solution of XT (57 µM) with *i*-PrOH (10 vol%) in argon-saturated acetonitrile and a 355 nm laser (25.7 mJ) for excitation. Upper panel: Spectrum of ^{3}XT at different delay times with a band centered around 485 nm, which is assigned to XTH$^•$.[25] Lower panel: Kinetic decay traces detected at three different detection wavelengths (^{3}XT: light gray data 300 nm, black data 625 nm; XTH$^•$: dark gray data 485 nm). The green lines indicate the delay times that were chosen for the detection of the TA spectra (same color code). The results of the mono-exponential fits are shown in red.

This experiment (Figure S 2) was performed to obtain a reference spectrum of XTH$^•$, which would allow us to exclude any HAT reactivity between ^{3}XT and the substrate **7a**. The spectroscopic features of XTH$^•$ are literature-known and can be observed in our case by adding *i*-PrOH into the sample solution. The lifetime of ^{3}XT decreases [τ(625nm) = 852 ns, τ(300nm) = 983 ns] and a quenching efficiency of η ~ 85 % could be determined under our conditions. The resulting species shows three absorption bands, one centered around 485 nm and a second one, which overlaps with the 625 nm band of the of ^{3}XT, and another band in the near UV region. Those bands are also reported in literature for the HAT product XTH$^•$.[25,26]

11.3 Additional Time-Resolved Experiments with Thioxanthone:

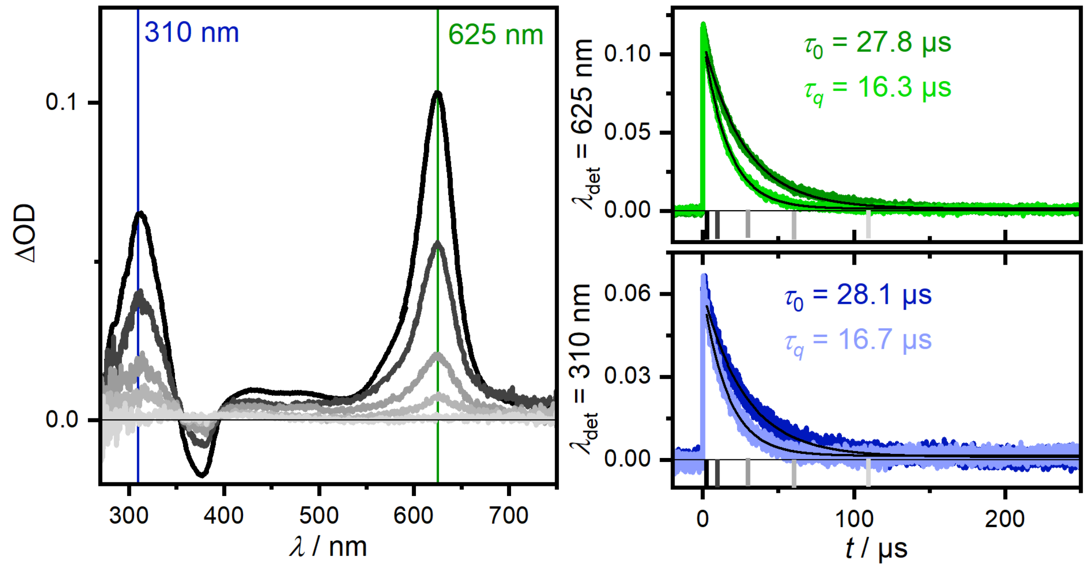

Figure S 3. TA data for quenching of ^{3}TXT with **7a**. A stock solution of TXT (26 µM) in MeCN was excited by a 355 nm pulsed laser (5.0 mJ). Left: TA spectra of ^{3}TXT in the presence of **7a** (1.0 mM). The delay times are also indicated in the kinetic decay traces using the same color code. Right: Kinetic decay traces of ^{3}TXT detected at 635 nm (upper) and 310 nm (lower). The dark green and blue traces correspond to ^{3}TXT in the absence of a quencher and the lighter colored traces correspond to the solutions with **7a** added.

Figure S 3 shows the ns-TA study of ^{3}TXT with the allene amide **7a** as a quencher in acetonitrile. The quenching is inefficient under chosen conditions (η ~ 40 %) and the quenching rate constant was estimated as $k_q \sim 3 \times 10^7$ M^{-1} s^{-1}, which is almost three orders of magnitude below the diffusion limit in acetonitrile.[4] The TA spectra decay back to the baseline and no new absorption bands are formed.

12. UV/Vis Control Experiments and Optimization of TA Conditions

12.1 Absorbance of XT at 355 nm

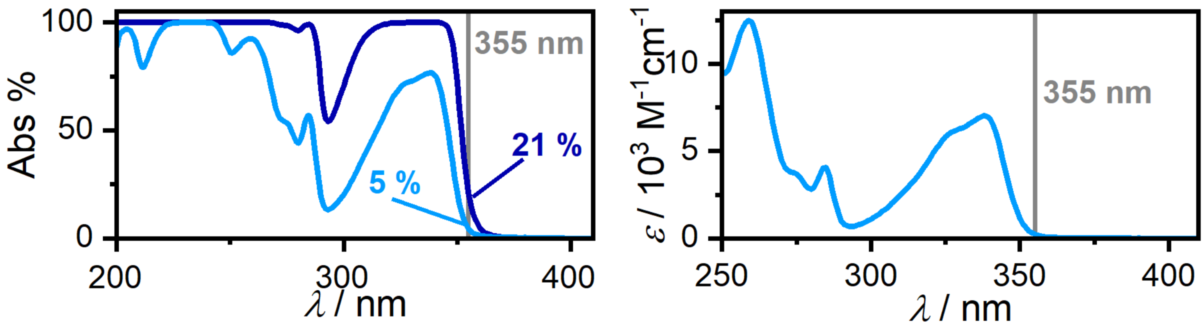

Figure S 4. Left: Percentage of absorbed photons of XT in MeCN (500 µM dark blue and 90 µM light blue). Right: Calibrated UV/vis spectrum of XT using a 90 µM solution of XT in MeCN. The wavelength of the excitation laser is indicated by the gray line (λ_{exc} = 355 nm).

Different concentrations of XT were chosen for the LFP measurements. The extinction coefficient of XT in MeCN at the excitation wavelength provided by the frequency-tripled Nd:YAG laser (355 nm) is ~ 200 $M^{-1}cm^{-1}$ (right part of Figure S 4, also in accordance with literature)[27]. The left part of Figure S 4 shows the percentage of absorbed photons at 90 µM or 500 µM XT in MeCN. High optical density at 355 nm can only be achieved at high concentrations (~ 500 µM) which facilitates self-quenching and causes filter effects in the spectral area below 350 nm. At lower sensitizer concentrations (e.g. 90 µM) a higher number of repetitions is needed for good data quality. This, however, also resulted in the sample being exposed to several laser pulses which might lead to decomposition of the sample. Those aspects were considered for all measurements and therefore sample preparation as well as instrument settings were chosen carefully.

The percentage of the absorbed photons in Figure S 4 was calculated using a rearranged representation of the Beer-Lambert law.[28] A sample calculation confirming the 21 % of absorbed photons indicated in the left part of Figure S 4 is given below.

$$Abs_{\%} = I_0 - I_1 = I_0 - I_0 \cdot 10^{-\varepsilon c d} = 1 - (1 \cdot 10^{-(200\ \mathrm{M}^{-1}\ \mathrm{cm}^{-1}) \cdot 0.0005\ \mathrm{M} \cdot 1\ \mathrm{cm})}) = 0.206$$

Here, $Abs_{\%} = I_0 - I_1$ gives the percentage (after its multiplication by 100) of absorbed photons by a given initial light intensity at the detector I_0 set as 1.

To limit the decomposition of XT throughout the measurements to a minimum, the samples were exchanged after each measurement and control UV/vis measurements were carried out before and after each laser experiment.

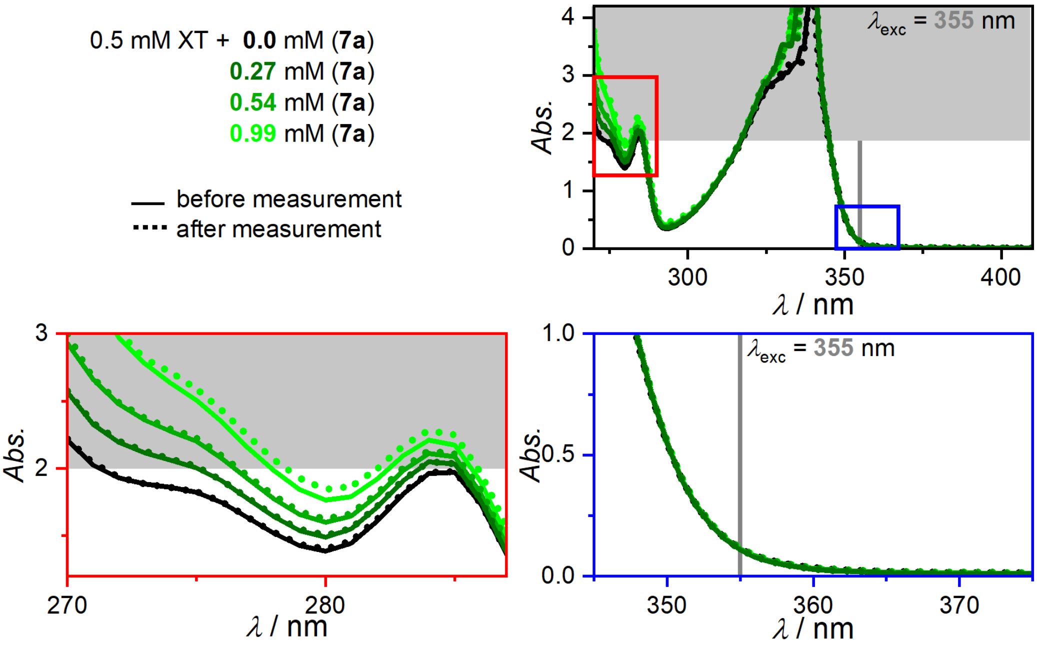

Figure S 5. UV/vis spectra of XT (0.5 mM) and quencher (**7a**) in MeCN before (solid lines) and after (dotted) the TA laser measurements for the Stern-Volmer plot (λ_{exc} = 355 nm, 15 mJ, see also Figure 1 in the main part). Two areas are enlarged representing the isolated XT absorption (blue box) and the combined XT and **7a** absorption bands (red box). The areas with gray background indicate the non-linear detection regime.

Figure S 5 shows the control UV/vis spectra related to the experiments of the Stern-Volmer plot of ^{3}XT which is quenched by **7a** (Figure 2 main paper) before (solid lines) and after (dashed

lines) the measurements using a frequency-tripled Nd:YAG laser for excitation (355 nm, 15 mJ). Two areas are enlarged representing the isolated XT absorption (blue box) or the combined XT and **7a** absorption signals (red box). Those enlarged areas highlight two important observations: i) the absorption of XT is unaffected by the laser excitation (blue box) and ii) the UV/vis signals of **7a** (red box) are essentially unaffected as well. A minor signal increase in the light green spectrum is most likely due to the cyclisation that occurs after the energy transfer; for that sample some additional exploratory and control measurements were performed (after recording the kinetic decay that was used for the Stern-Volmer analysis) and for that reason the solution was exposed to more laser shots thus showing more pronounced changes in the UV/vis spectrum and slight decomposition of the quencher. The samples were exposed to 10 laser pulses for the kinetic measurements and additional 10-20 pulses for further control measurements. Generally, a fresh sample of a 500 µM XT stock solution in MeCN was used for each concentration of **7a**. The areas with gray background (*Abs.* >2) indicate the limits for a linear resolution of the spectrometer.

Although the four independent measurements used for our Stern-Volmer analysis do not permit a statistical analysis and a highly reliable quantitative value for the bimolecular rate constant, it allows us to conclude that the energy transfer step is highly efficient under our typical conditions used for photocatalysis (see main part for details).[29-32]

12.2 Different Excitation Intensities

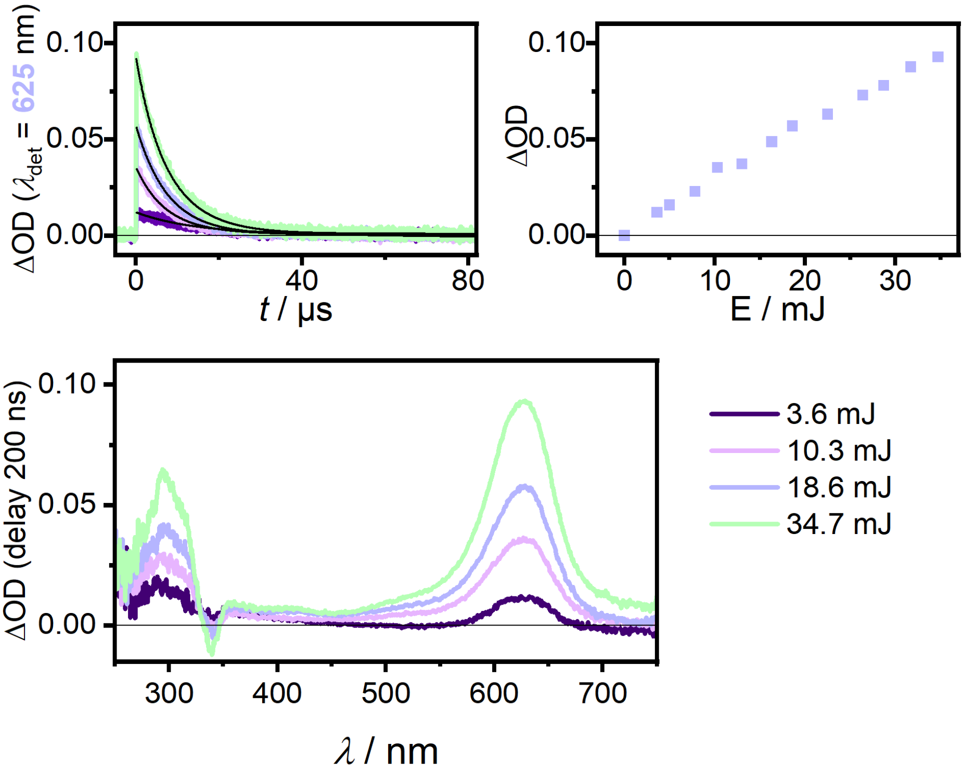

Figure S 6. Transient absorption data of XT (90 µM) in MeCN at different laser energies (color coded). Selected kinetic decay traces measured at 625 nm and the resulting ΔOD was plotted against the employed laser intensity (upper panel). At selected excitation intensities (3.6 mJ, 10.3 mJ, 18.6 mJ and 34.7 mJ) TA spectra were recorded (lower panel).

The LFP measurements using of XT (90 µM in Figure S 6 or 500 µM in Figure S 8) as a photosensitizer were performed to investigate the stability of XT at different excitation

intensities and to investigate possible saturation effects. A stock solution (XT, 90 µM or 500 µM) was prepared which provided three samples that were used for stability studies at different excitation intensities. A stream of argon was bubbled through each solution for 10 min to provide oxygen-free conditions and then the samples were placed inside the cuvette holder for 2 min before the measurement for temperature equilibration. The excited state absorption band centered around 310 nm is strongly affected by filter effects, because of the high molar absorption coefficient of the ground state at wavelengths < 350 nm (Figure S 4). Kinetic decay traces were recorded at 625 nm and fitted using a biexponential fitting function of the type $y = A_1 e^{-\left(\frac{x}{t_1}\right)} + A_2 e^{-\left(\frac{x}{t_2}\right)} + A_0$ (due to better representation of the initial amplitude depending on the excitation laser intensity). The resulting initial intensity (sum of the scaling factors of the two exponentials A_1 and A_2 and the constant A_0) was plotted against the employed laser intensity. This plot shows a linear correlation between ΔOD and excitation intensity and no saturation.[33, 34] Similar results were obtained for a stock solution containing 500 µM of XT in MeCN (Figure S 8) and even at high intensities of the excitation laser no saturation was observed (for 34.7 mJ less than 25 % of the molecules were excited, as described in the tables below).

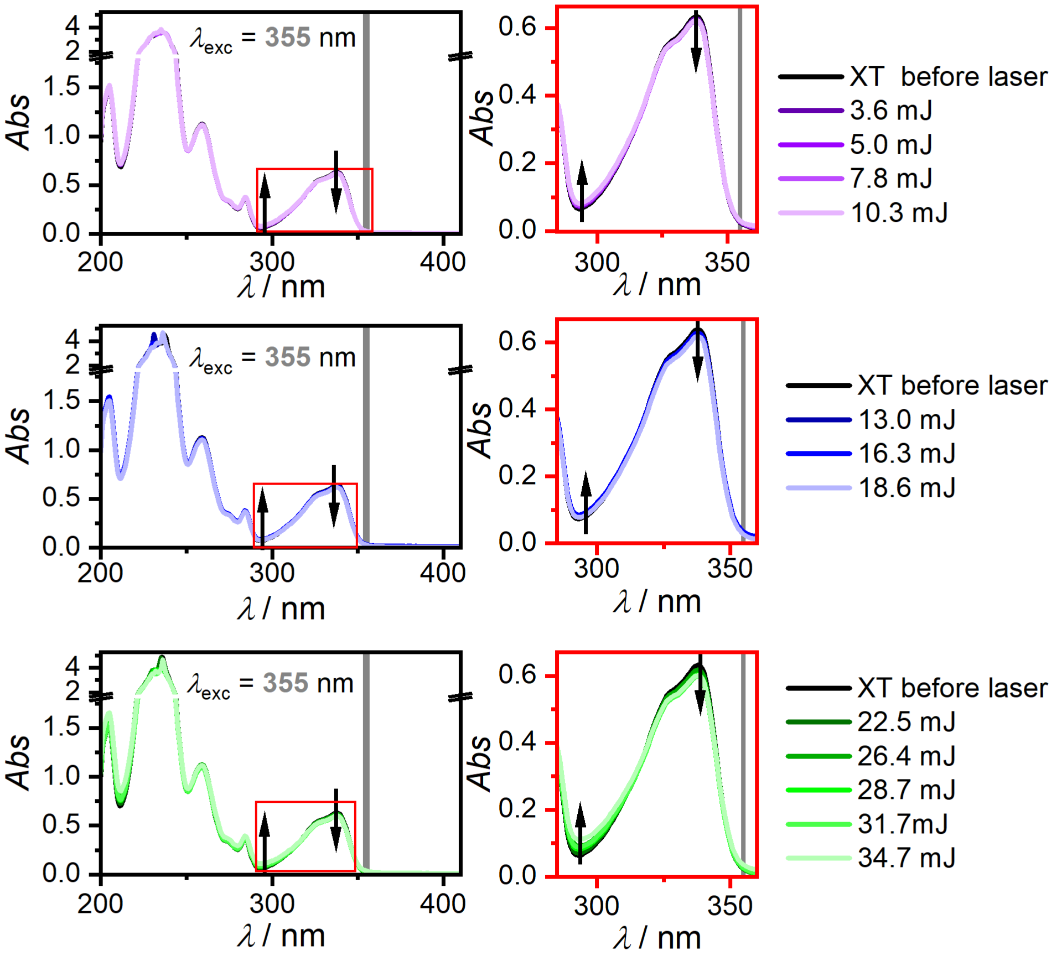

Figure S 7. UV/vis spectra of the XT solutions used for the intensity-dependent studies in Figure S 6. Three samples out of one stock solution (XT, 90 µM) were prepared (first sample for 3.6 mJ – 10.3 mJ measurements, second sample for 13.0 mJ – 18.6 mJ measurements and third sample for 22.5 mJ – 34.7 mJ measurements) and the UV/vis spectra were recorded before (black data) and after the respective laser measurement. The left column presents the full spectrum, and the middle column presents the enlarged area (red box). A gray line indicates the excitation wavelength (355 nm), and the arrows highlight the changes of the absorption bands.

The stability of XT in MeCN was investigated in detail under the conditions employed for the intensity-dependent LFP measurements. Absorption spectra were measured before (black spectra in Figure S 7) and after each intensity setting of the laser (color-coded in Figure S 7). The samples were exposed to 15 laser pulses for the kinetic decay measurements and additional 10 pulses if spectra were measured. Especially in the enlarged area (Figure S 7 middle column) it becomes apparent that the sample decomposes over the course of the measurement. However, even at the highest laser intensities, and after multiple laser pulses (bottom panel in Figure S 7, 22.5 mJ – 34.5 mJ) the decomposition was below 10 % (90 µM, about 5 % XT signal decrease at 340nm). Similar results were obtained at high concentrations of XT (500 µM in MeCN) and the corresponding control measurements are presented in Figure S 8 and Figure S 9. The stability was carefully considered for the data presented in Figure 2 of the main part and the solution was exchanged for each quencher (**7a**) concentration (see also Figure S 5).

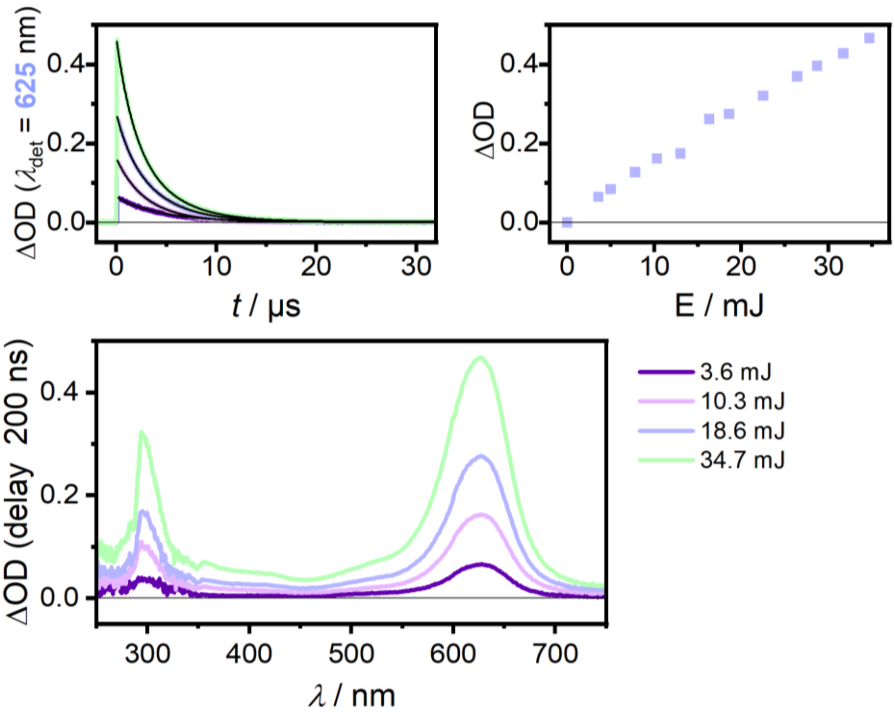

Figure S 8. Transient absorption data of XT (500 µM) in MeCN at different laser energies (color coded). Selected kinetic decay traces measured at 625 nm and the resulting ΔOD was plotted against the employed laser intensity (upper panel). At selected excitation intensities (3.6 mJ, 10.3 mJ, 18.6 mJ and 34.7 mJ) TA spectra were recorded (lower panel).

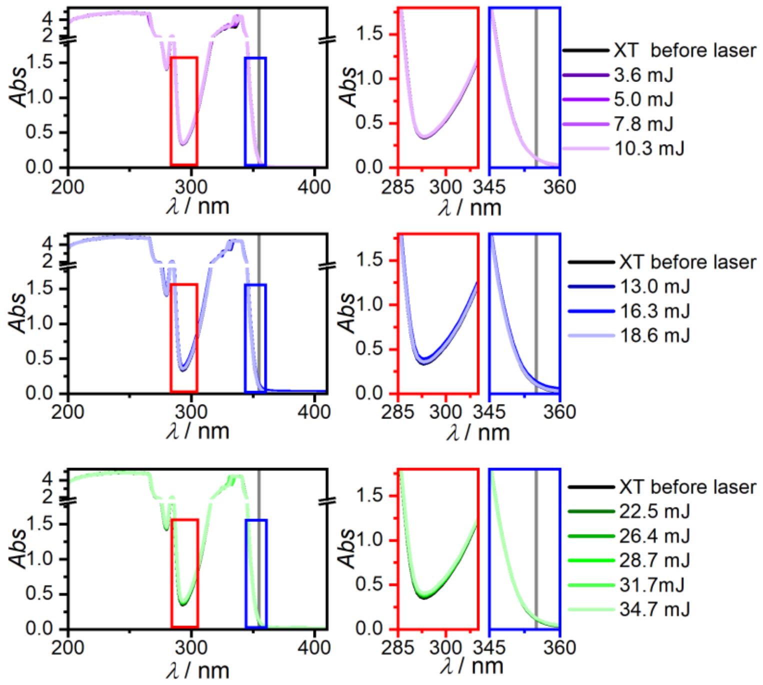

Figure S 9. UV/vis spectra of the XT solutions used for the intensity study in Figure S 8. Three samples out of one stock solution (XT, 500 µM) were prepared (first sample for 3.6 mJ – 10.3 mJ measurements, second sample for 13.0 mJ – 18.6 mJ measurements and third sample for 22.5 mJ – 34.7 mJ measurements) and the UV/vis spectra were recorded before (black data) and after the respective laser measurement. The left column presents the full spectrum, and the middle column presents the enlarged areas (red or blue box). A gray line indicates the excitation wavelength (355 nm).

Based on our measured ΔOD values right after the laser pulse, the pathlength *d* of the pump-probe overlap region (0.8 cm, limited by the laser beam as explained above), as well as the range of molar absorption coefficients of the **^{3}XT** absorption band in the red spectral region that can be found in the literature (ε_T between 5300 M^{-1} cm^{-1} [35] and 18800 M^{-1} cm^{-1} [27], we conclude that the fraction of excited triplet molecules relative to the ground state XT molecules must be between 2.6 and 21.2 % throughout our study. The tables shown below display the corresponding calculations for the highest and the lowest ε_T.

Calculated with ε_T = 5300 M^{-1} cm^{-1}

Figure in paper	ground state XT concentration / M	laser pulse energy / mJ	max. ΔOD ^{3}XT (625 nm, t = 0)	max. ^{3}XT concentration	^{3}XT / ground state XT
**2 (left)**	9.00E-05	10.5	0.035	8.25472E-06	9.2%
**2 (right)**	5.00E-04	15	0.26	6.13208E-05	12.3%
**S1**	6.30E-05	25.7	0.052	1.22642E-05	19.5%
**S2**	5.70E-05	25.7	0.044	1.03774E-05	18.2%
**S8**	5.00E-04	34.7	0.45	0.000106132	21.2%

Calculated with ε_T = 18800M^{-1} cm^{-1}

Figure in paper	ground state XT concentration / M	laser pulse energy / mJ	max. ΔOD ^{3}XT (625 nm, t = 0)	max. ^{3}XT concentration	^{3}XT / ground state XT
**2 (left)**	9.00E-05	10.5	0.035	2.32713E-06	2.6%
**2 (right)**	5.00E-04	15	0.26	1.72872E-05	3.5%
**S1**	6.30E-05	25.7	0.052	3.45745E-06	5.5%
**S2**	5.70E-05	25.7	0.044	2.92553E-06	5.1%
**S8**	5.00E-04	34.7	0.45	2.99202E-05	6.0%

13. DFT Calculations

13.1 General Information

The DFT calculations for structure optimization and spin density plots were carried out with the program package Orca 6.0[36] using the B3LYP functional combined with a 6-31g basis set. Compound **7a** was optimized in the singlet ground state and the resulting energy was used as reference to calculate the energy differences to the triplet states and the diradicals (Figure 3 of the main part). For the structures for **7a**, ^{3}**7a**$_{\mathbf{bent}}$ and ^{3}**17a** geometry optimizations were performed and vibrational frequencies were calculated. No negative frequencies were observed in the optimized structures indicating the successful convergence to the minimum structures. The optimized ground-state structure of **7a** was then used as a starting geometry to optimize ^{3}**7a***. For the vertical triplet energy (^{3}**7a**$_{\mathbf{vert}}$) the single point energy of **7a** in a triplet manifold was calculated. Then the structure of the HAT product ^{3}**17a** was optimized with a triplet multiplicity. The optimized structure was then used as the starting point for optimizing ^{3}**7**$_{\mathbf{bent}}$.
Further calculations were performed using two extended basis sets (6-31g(d) and 6-311++g(d,p)). The ground state **7a** was optimized for each basis set and the resulting energy was used as reference point for the energy differences to the triplet states and the diradical. The optimized geometries of the ground states obtained with the respective basis sets were used for further calculations of ^{3}**7a*** (with geometry optimization for the adiabatic triplet) and ^{3}**7a**$_{\mathbf{vert}}$ (without geometry optimization for the vertical triplet). As initial structures for compound (^{3}**7a**$_{\mathbf{bent}}$) and the diradical (^{3}**17a**) the output data from the respective calculations using 6-31g basis set were used and for both compounds and basis sets the geometry optimizations were performed.
The pseudo-vertical triplet structures using 6-31g(d) and 6-311++g(d,p) as basis sets did already result in bent structures, which was not the case for the optimized triplet using 6-31g (see Tables S1 – S3). For that reason, we also optimized (using all three basis sets) a structure restricting the angle of the allene carbons (^{3}**7a**$_{\mathbf{restr}}$), which was taken from the vertical triplet structures. For 6-31(d) and 6-311g(d,p) as basis sets, the optimized structures showed one imaginary frequency, which is the bending motion of the angle-restricted allene carbons.
Additional single point calculations were used to calculate the spin densities of the optimized structures. To plot the spin densities, Avogadro1.2.0.[37] was used. Positive spin density is represented in blue and negative spin density in red using an iso value of 0.002 in Figure 3 of the main part.
Except for the three structures with the restrained angles of the allene carbon atoms, no further conditions were set for the DFT calculations and the default settings of Orca 6.0 were used (*i. e.* grid: “Grid4”, convergence criteria: “tight”, no solvent model, details can be found in the Orca manual and the output files).[38]

13.2 Mulliken Spin Density Analysis

The spin density analysis of the triplet states and diradicals are summarized in Table S 1. The sum of the spin populations was 2 for all structures, which is expected for triplet structures. **^{3}7a*** is the pseudo-vertical triplet, with the highest spin density located on the oxygen atom (0.7325), followed by the carbon atoms listed in Table S 1.

For the bent structure **^{3}7a$_{bent}$**, the highest spin densities can be found delocalized over the bent allene system. Furthermore, the spin density at C2 points towards the benzylic hydrogen atoms, whereas those for C1 and C5 are almost orthogonally oriented.

In structure **^{3}17a**, the highest contributions can be found at C5 and C9, the two carbons that are involved in the cyclization step. C5 and C1 are both at the edges of the former allene moiety, and for that reason spin density is also located at C1.

Table S 1. Structures of ^{3}**7a***$_{vert}$, ^{3}**7a***, ^{3}**7a***$_{restr}$, ^{3}**7a**$_{bent}$ and ^{3}**17a**, their energies relative to the singlet ground state energy of **7a** using 6-31g as a basis set. The spin densities are given for the highlighted and numbered carbon atoms. The spin density plots show positive spin density in blue and negative spin density in red.

**Species (relative energy/eV)**	**Optimized structure**	**Spin density[a]**	**Spin density at C# (sum of spin populations)**
**^{3}7a*$_{vert}$ (4.03)**	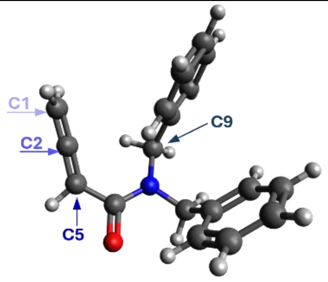	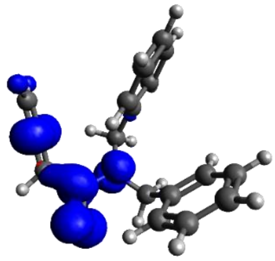	C1: -0.048462<br>C2: 0.491140<br>C5: -0.005505<br>C9: -0.023285<br><br>(2)
**^{3}7a* (3.00)**	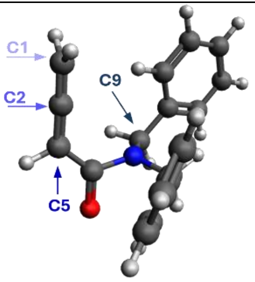	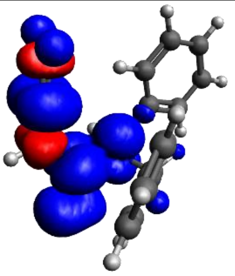	C1: -0.127964<br>C2: 0.578077<br>C5: -0.244777<br>C9: -0.022916<br><br>(2)
**^{3}7a*$_{restr}$ (3.00)**	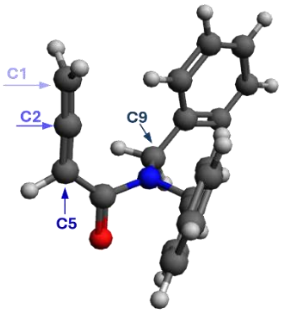	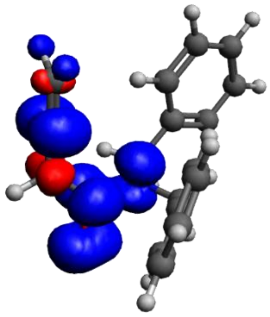	C1: -0.128181<br>C2: 0.578009<br>C5: -0.244743<br>C9: -0.022886<br><br>(2)
**^{3}7a$_{bent}$ (1.98)**	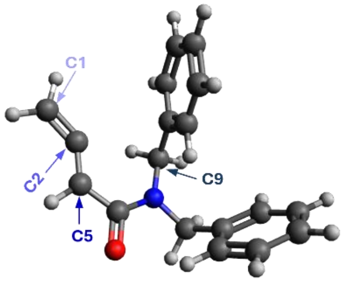	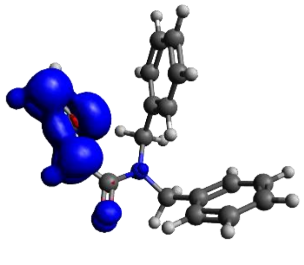	C1: 0.560966 [b]<br>C2: 0.683678 [b]<br>C5: 0.546088 [b]<br>C9: 0.006304<br><br>(2)
**^{3}17a (0.84)**	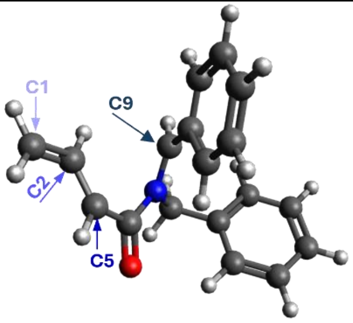	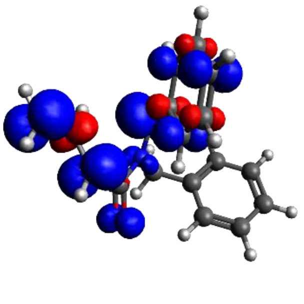	C1: 0.635293 [b]<br>C2: -0.285328<br>C5: 0.652379 [b]<br>C9: 0.710468 [b]<br><br>(2)

[a] Iso value 0.005.
[b] Highest contributions out of all atoms.

Table S 2. Structures of ^{3}**7a***$_{vert}$, ^{3}**7a***, ^{3}**7a***$_{restr}$, ^{3}**7a**$_{bent}$ and ^{3}**17a**, their energies relative to the singlet ground state energy of **7a** using 6-31g(d) as a basis set. The spin densities are given for the highlighted and numbered carbon atoms. The spin density plots show positive spin density in blue and negative spin density in red.

Species (relative energy/eV)	Optimized structure	Spin density[a]	Spin density at C# (sum of spin populations)
^{3}**7a***$_{vert}$ (4.18)	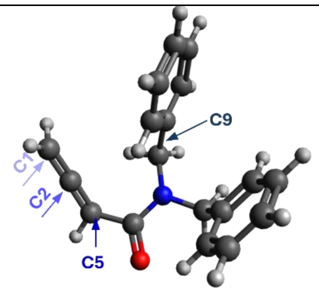	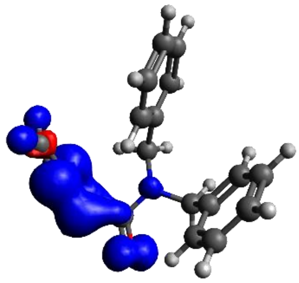	C1: -0.121924<br>C2: 0.806218<br>C5: 0.613078<br>C9: 0.007740<br><br>(2)
^{3}**7a*** (2.08)	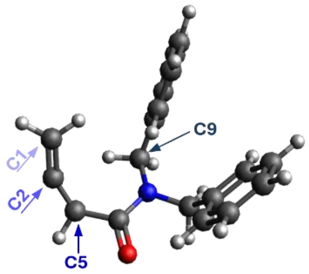	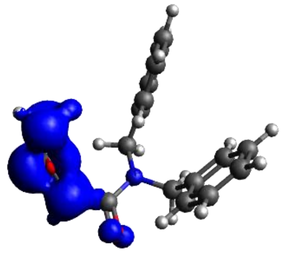	C1: 0.552189 [b]<br>C2: 0.704339 [b<br>C5: 0.552749 [b]<br>C9: 0.000656<br><br>(2)
^{3}**7a***$_{restr}$ (2.14)	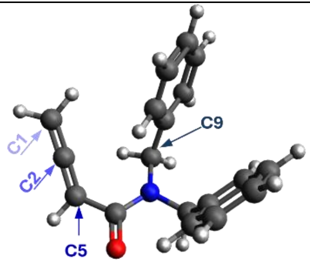	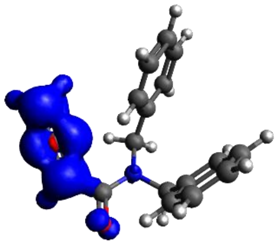	C1: 0.527068 [b]<br>C2: 0.697313 [b<br>C5: 0.515288 [b]<br>C9: 0.006509<br><br>(2)
^{3}**7a**$_{bent}$ (1.98)	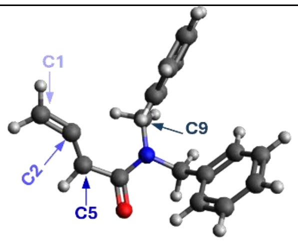	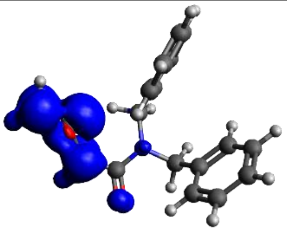	C1: 0.554166 [b]<br>C2: 0.691101 [b]<br>C5: 0.546542 [b]<br>C9: 0.015892<br><br>(2)
^{3}**17a** (0.89)	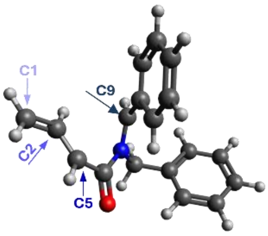	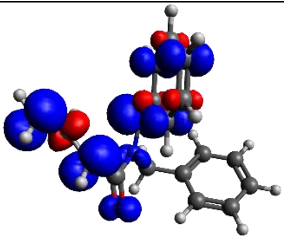	C1: 0.620348 [b]<br>C2: -0.251239<br>C5: 0.625900 [b]<br>C9: 0.672944 [b]<br><br>(2)

[a] Iso value 0.005.
[b] Highest contributions out of all atoms.

Table S 3. Structures of ^{3}**7a***$_{vert}$, ^{3}**7a***, ^{3}**7a***$_{restr}$, ^{3}**7a**$_{bent}$ and ^{3}**17a**, their energies relative to the singlet ground state energy of **7a** using 6-311++g(d,p) as a basis set. The spin densities are given for the highlighted and numbered carbon atoms. The spin density plots show positive spin density in blue and negative spin density in red.

**Species (relative energy/eV)**	**Optimized structure**	**Spin density[a]**	**Spin density at C# (sum of spin populations)**
^{3}**7a***$_{vert}$ **(4.21)**	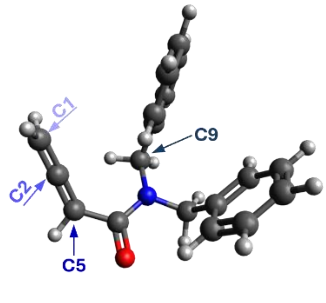	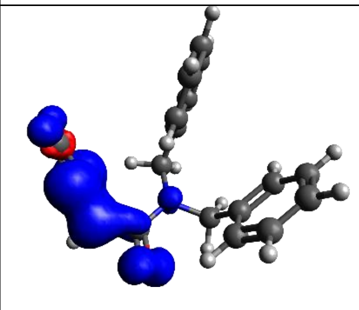	C1: -0.059443<br>C2: 0.751002<br>C5: 0.610522<br>C9: -0.112474<br>(2)
^{3}**7a*** **(2.00)**	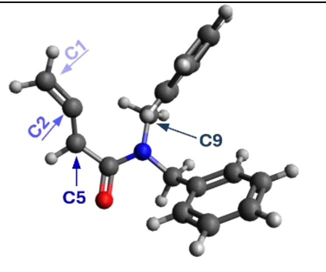	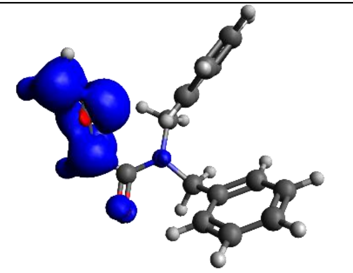	C1: 0.508756 [b]<br>C2: 0.754478 [b]<br>C5: 0.578251 [b]<br>C9: -0.049634<br>(2)
^{3}**7a***$_{restr}$ **(2.17)**	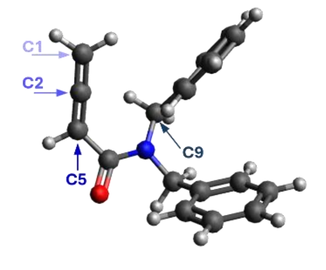	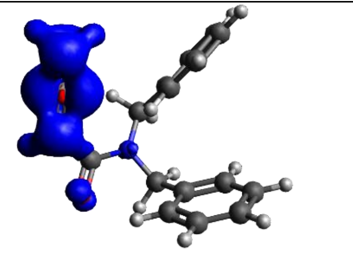	C1: 0.528817 [b]<br>C2: 0.737518 [b]<br>C5: 0.543836 [b]<br>C9: -0.056784<br>(2)
^{3}**7a**$_{bent}$ **(2.00)**	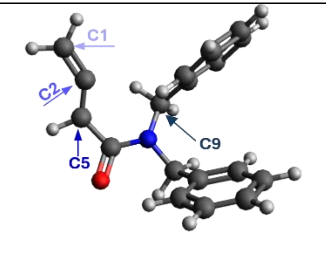	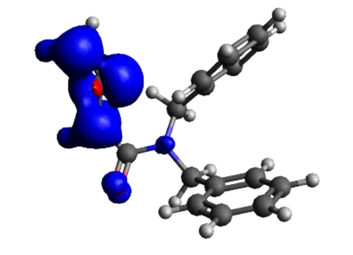	C1: 0.498715 [b]<br>C2: 0.748953 [b]<br>C5: 0.596772 [b]<br>C9: -0.056379<br>(2)
^{3}**17a** **(0.91)**	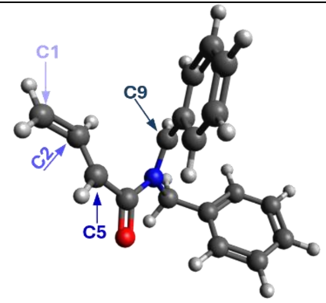	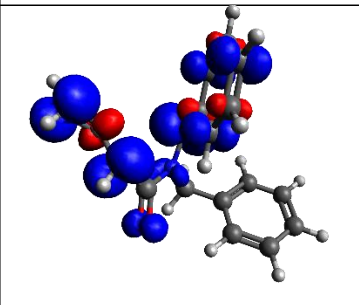	C1: 0.648137 [b]<br>C2: -0.276429<br>C5: 0.654339 [b]<br>C9: 0.699296 [b]<br>(2)

[a] Iso value 0.005.
[b] Highest contributions out of all atoms.

Similar results in spin density distributions and relative energies were obtained for the two more elaborate basis sets including diffuse and polarization functions (Tables S2 and S3). The only

exception is the pseudo-vertical triplet **^{3}7a***. For the structure obtained with the 6-31g basis set, the allene moiety of the structure is still linear and the spin density is very different from the bent form **^{3}7a*$_{bent}$**. Including polarization and diffuse functions lead to a different bent structure (6-31g(d)) or a structure that is essentially identical to the bent triplet (6-311++g(d,p)). In addition to the clear deviation from the linear structure, the spin densities for those adiabatic structures are already highly resembling the bent triplet structure. This is also reflected in the resulting energies that are much closer to those of the bent triplet structures (Figure S 10). The two triplet structures (vertical and adiabatic) only provide information about the upper and lower limits for a range within an experimental triplet energy is usually found.[39, 40] Finally, we attempted to restrict the allene bending (**^{3}7a*$_{restr}$**) to the ~180° angle provided by the ground state structure and optimize the structures. Like for the optimized triplet state **^{3}7a*** and despite the restriction, the spin densities and triplet energies highly resemble those of the bent structure for the 6-31g(d) and 6-311++g(d,p) basis sets (6-31g was calculated as reference). Compared to the pseudo-vertical triplets (**^{3}7a***) those structures do not provide new information for the discussion of the triplet energy for the substrate **7a**.

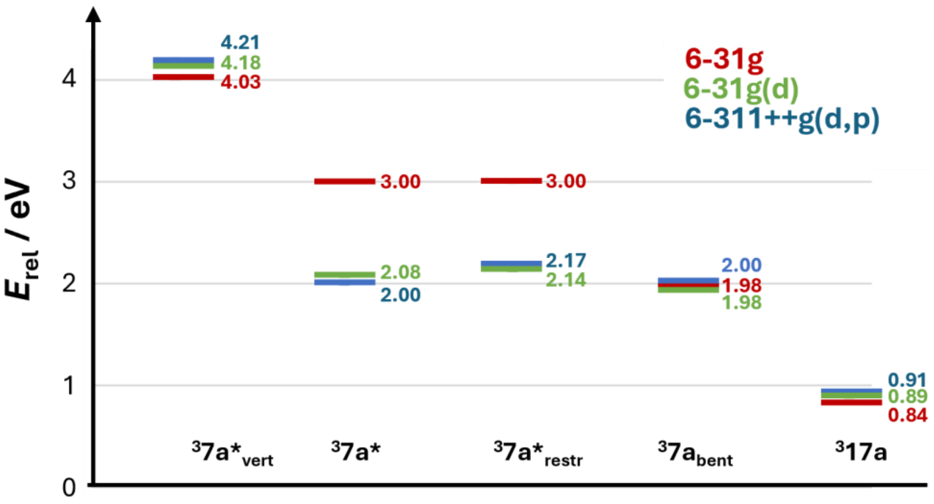

*Figure S 10 Extended version of figure 4 of the main part including the pseudo-vertical triplet state ^{3}**7a***. Comparison of calculated energies of triplet species using B3LYP as functional and different basis sets (color coded). The energy difference is given relative to the optimized ground state structure (**7a**) using the respective basis set for geometry optimization.*

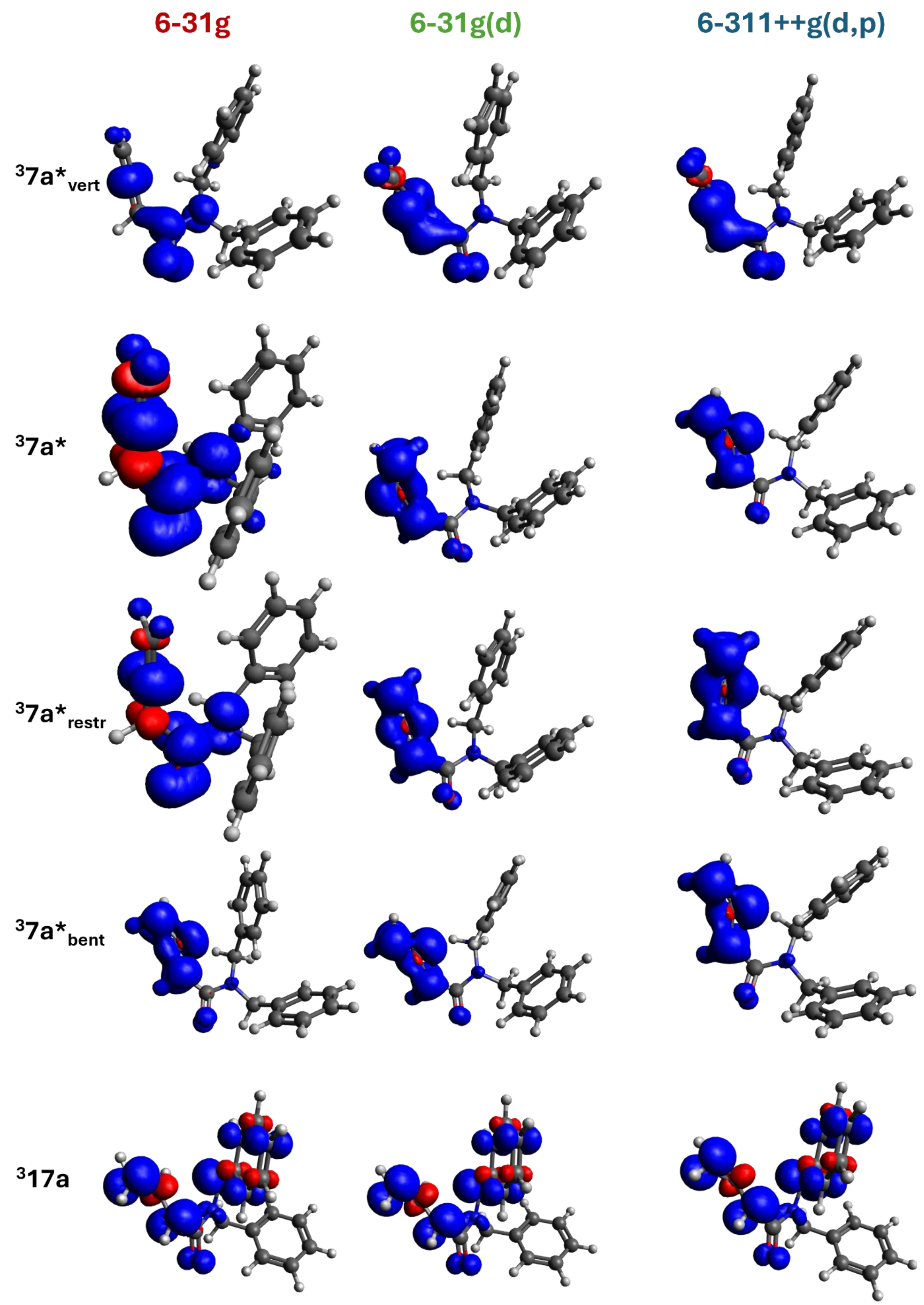

Figure S 11. Summary of all structures and spin densities from Tables S1-S3.

14. NMR Spectra of New Compounds

N,N-Dibenzylbuta-2,3-dienamide (7a)

^{1}H-NMR (500 MHz, DMSO-d^6, 298 K):

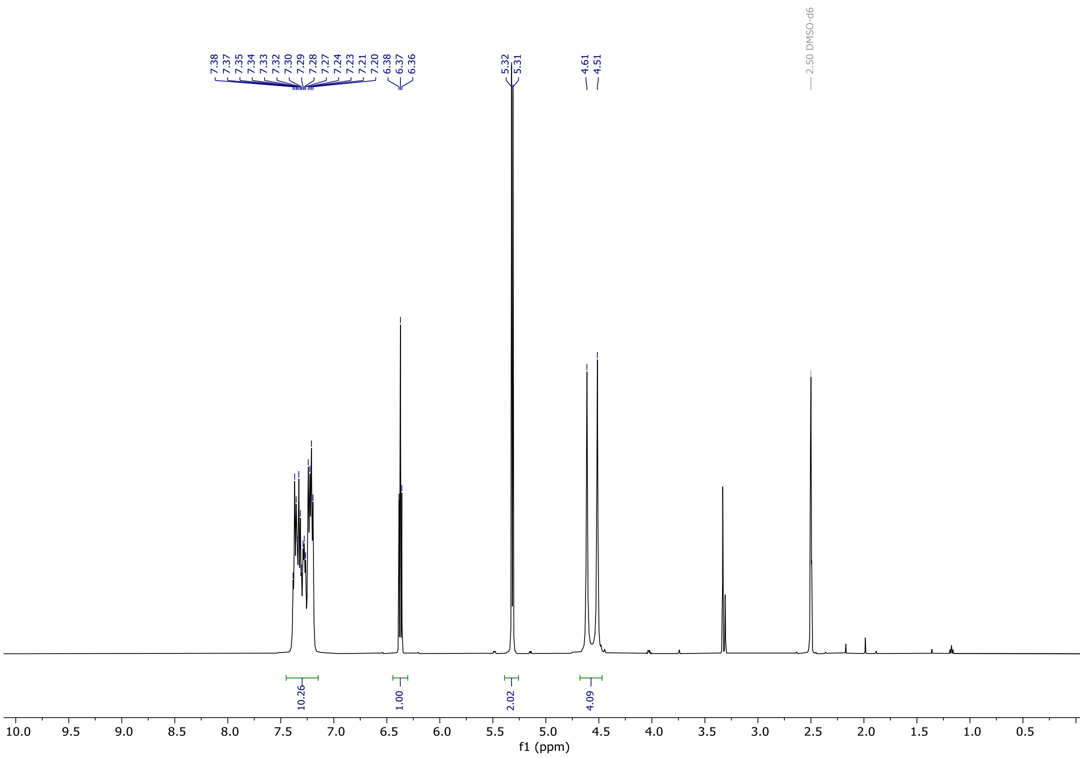

^{13}C-NMR (101 MHz, DMSO-d^6, 298 K):

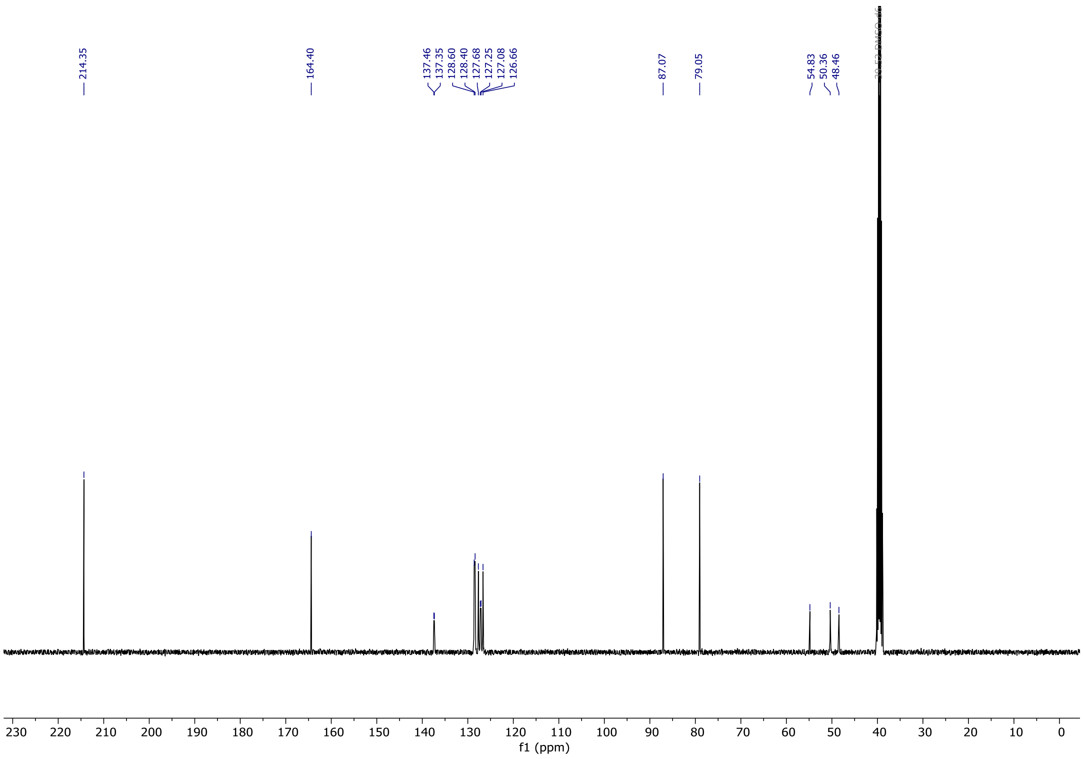

***N,N*-Bis(3-chlorobenzyl)buta-2,3-dienamide (7b)**

^{1}H-NMR (400 MHz, DMSO-d_6, 298 K):

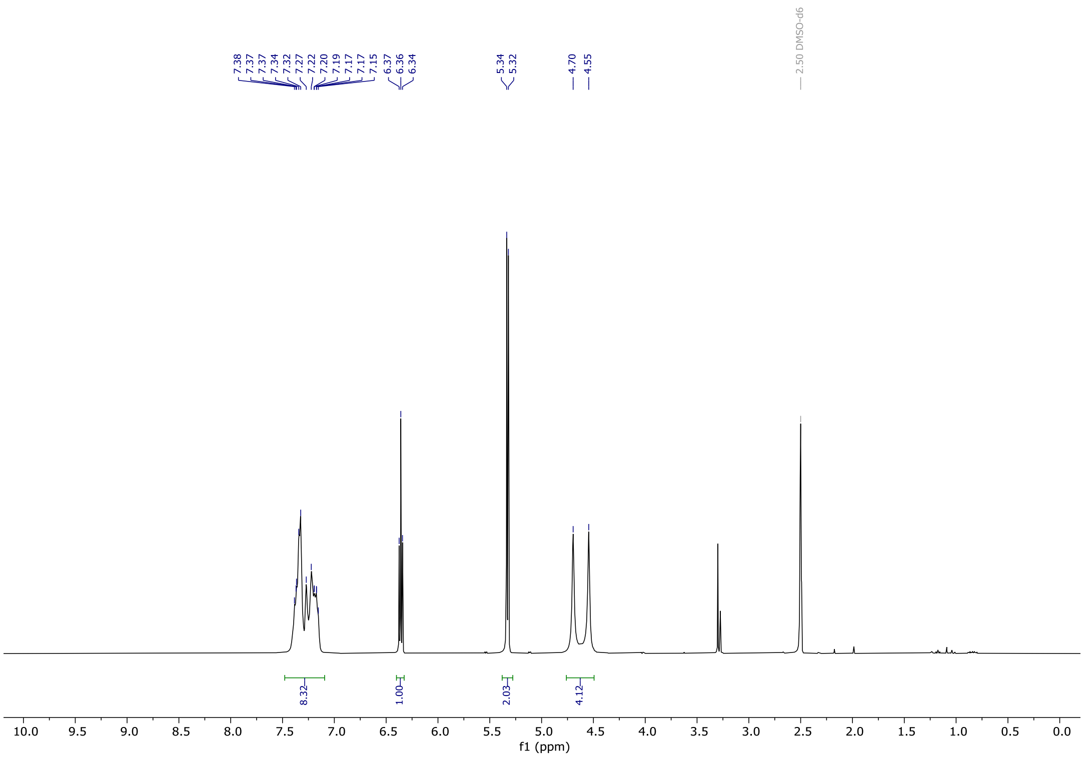

^{13}C-NMR (101 MHz, DMSO-d_6, 300 K):

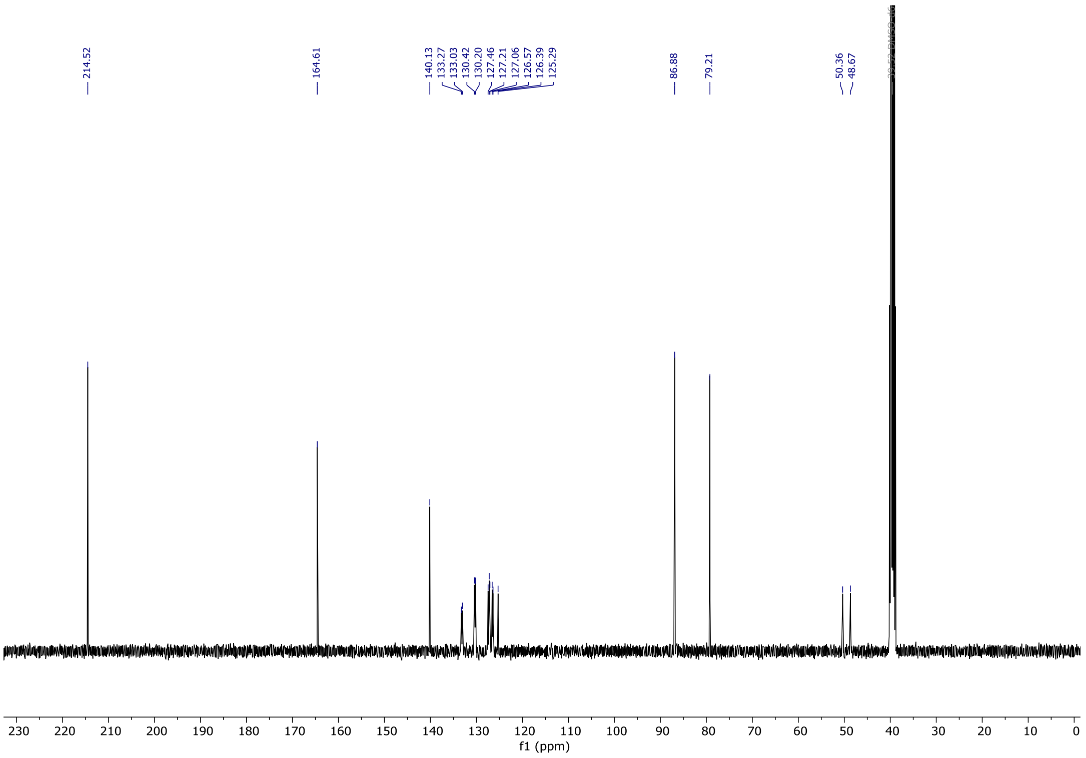

***N,N*-bis(4-fluorobenzyl)buta-2,3-dienamide (7c)**

^{1}**H-NMR** (400 MHz, DMSO-d_6, 298 K):

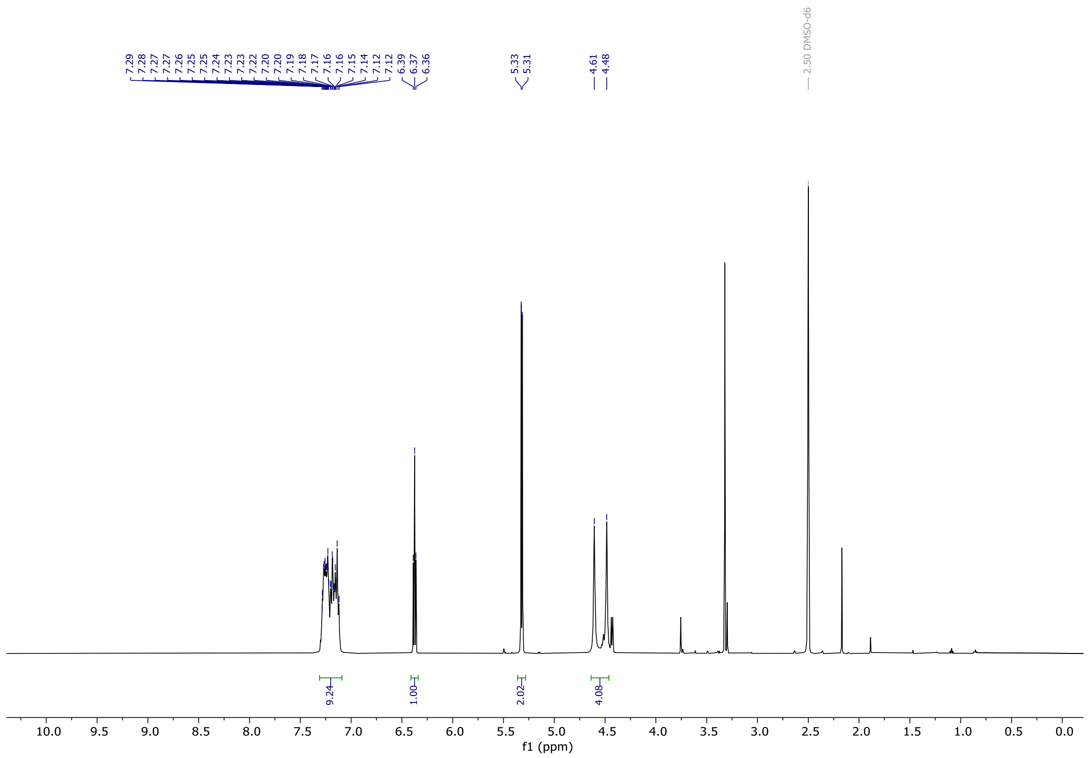

^{13}**C-NMR** (101 MHz, DMSO-d_6, 298 K):

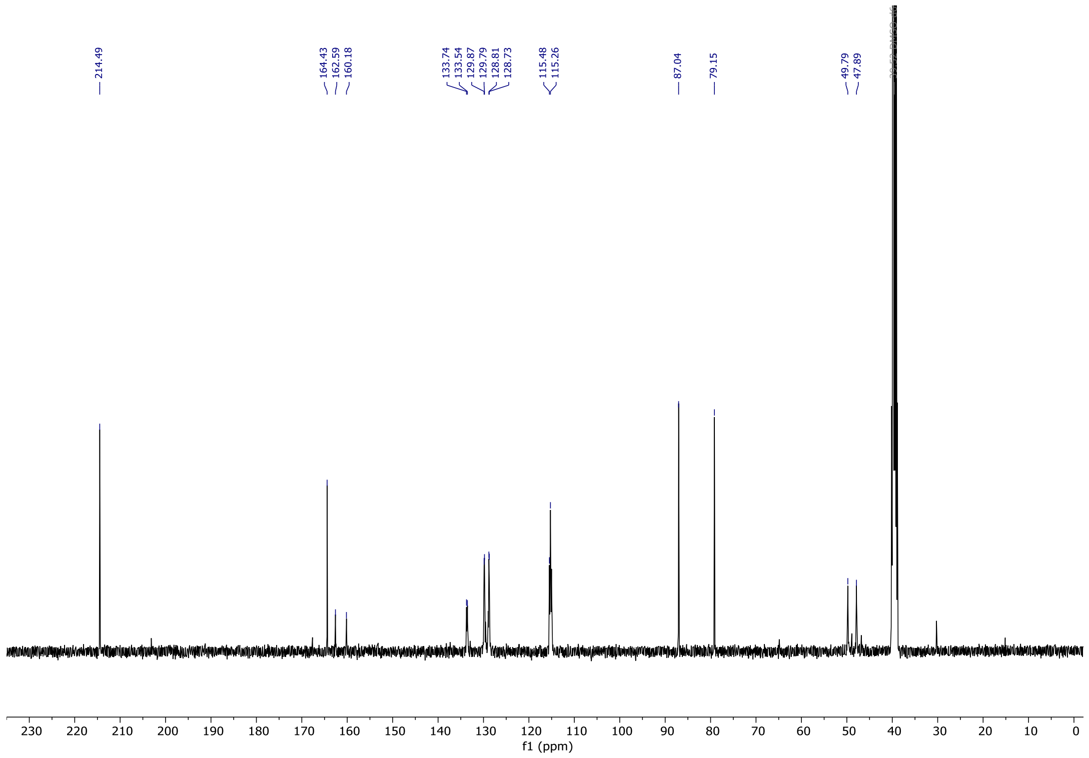

^{19}**F-NMR** (376 MHz, DMSO-d_6, 298 K):

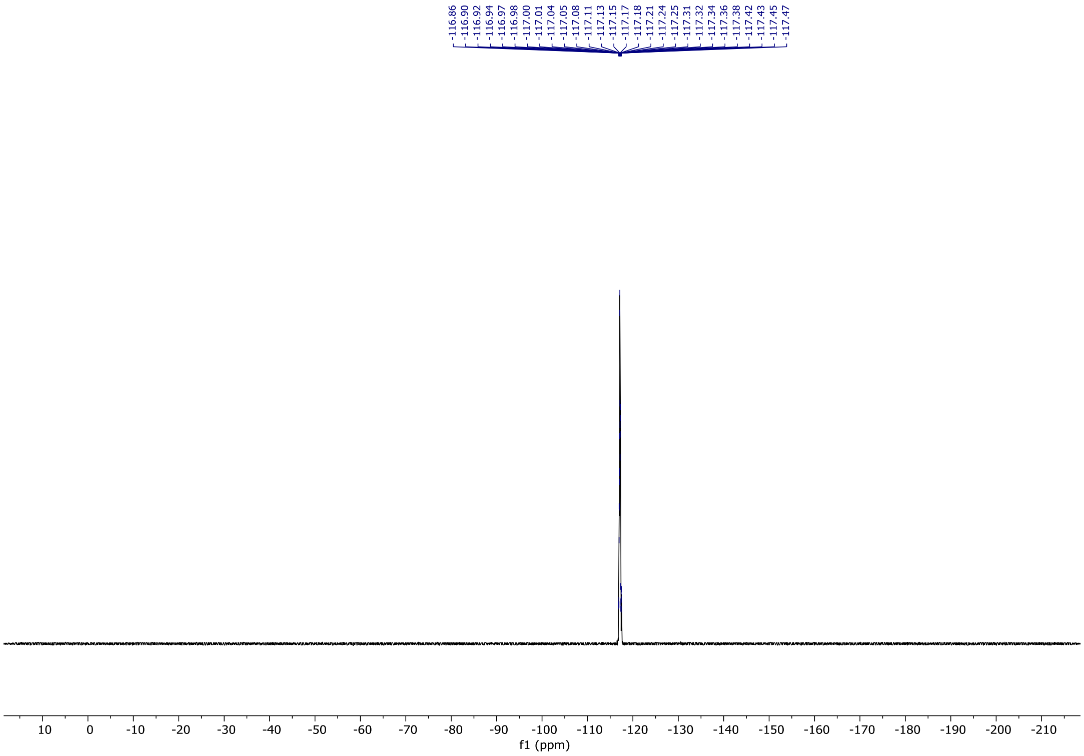
-116.86
-116.90
-116.92
-116.94
-116.97
-116.98
-117.00
-117.01
-117.04
-117.05
-117.08
-117.11
-117.13
-117.15
-117.17
-117.18
-117.21
-117.24
-117.25
-117.31
-117.32
-117.34
-117.36
-117.38
-117.42
-117.43
-117.45
-117.47
10 0 -10 -20 -30 -40 -50 -60 -70 -80 -90 -100 -110 -120 -130 -140 -150 -160 -170 -180 -190 -200 -210
f1 (ppm)

***N,N*-Bis(3,5-dimethylbenzyl)buta-2,3-dienamide (7d)**

^{1}H-NMR (400 MHz, DMSO-d_6, 298 K):

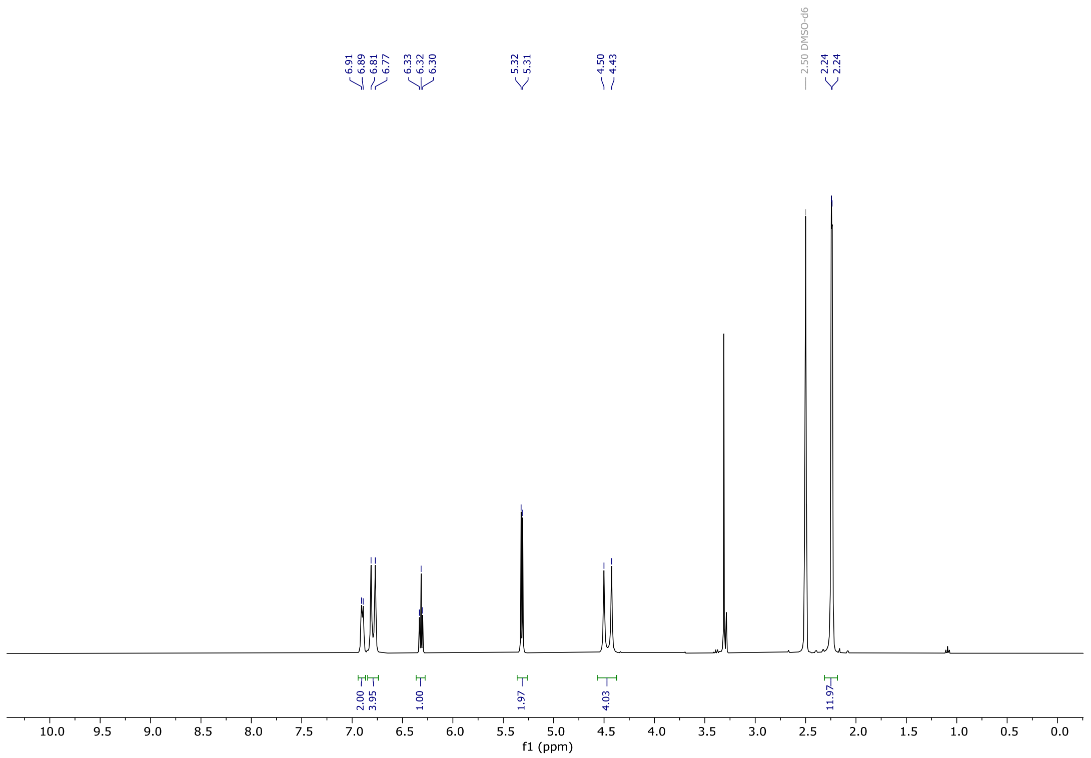

^{13}C-NMR (101 MHz, DMSO-d_6, 298 K):

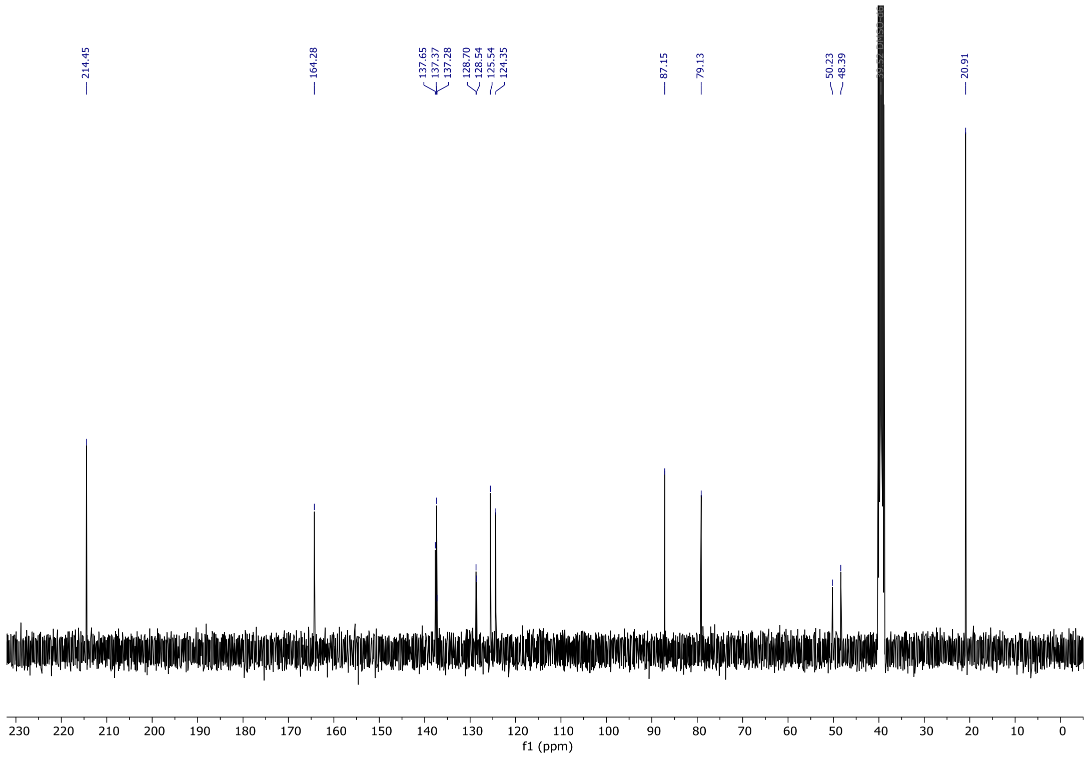

***N,N*-Bis(4-methoxybenzyl)buta-2,3-dienamide (7e)**

^{1}H-NMR (500 MHz, DMSO-d_6, 298 K):

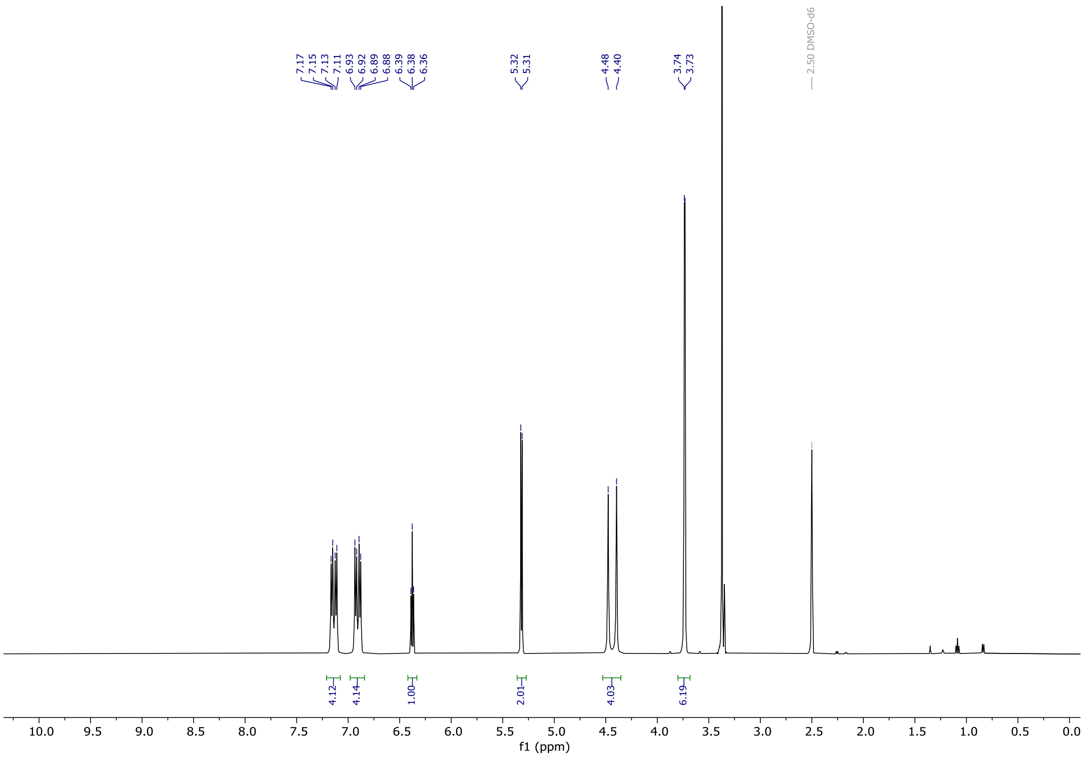

^{13}C-NMR (126 MHz, DMSO-d_6, 298 K):

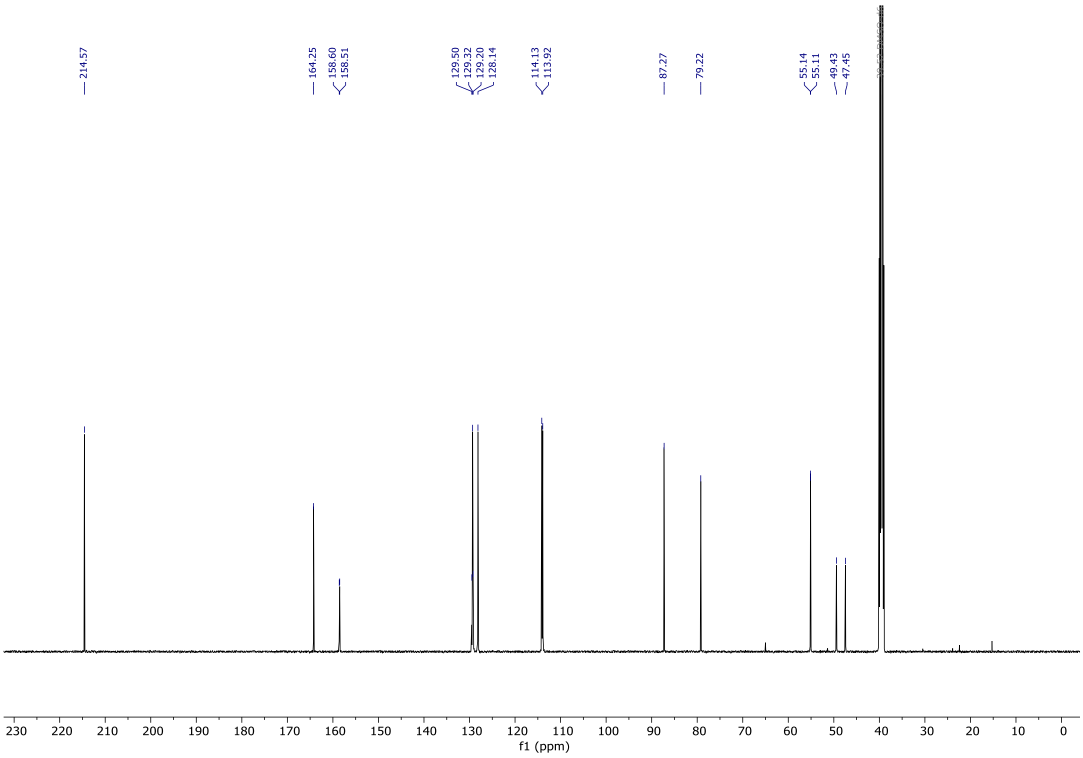

***N*-Benzyl-*N*-methylbuta-2,3-dienamide (7f)**

^{1}H-NMR (400 MHz, DMSO-d_6, 298 K):

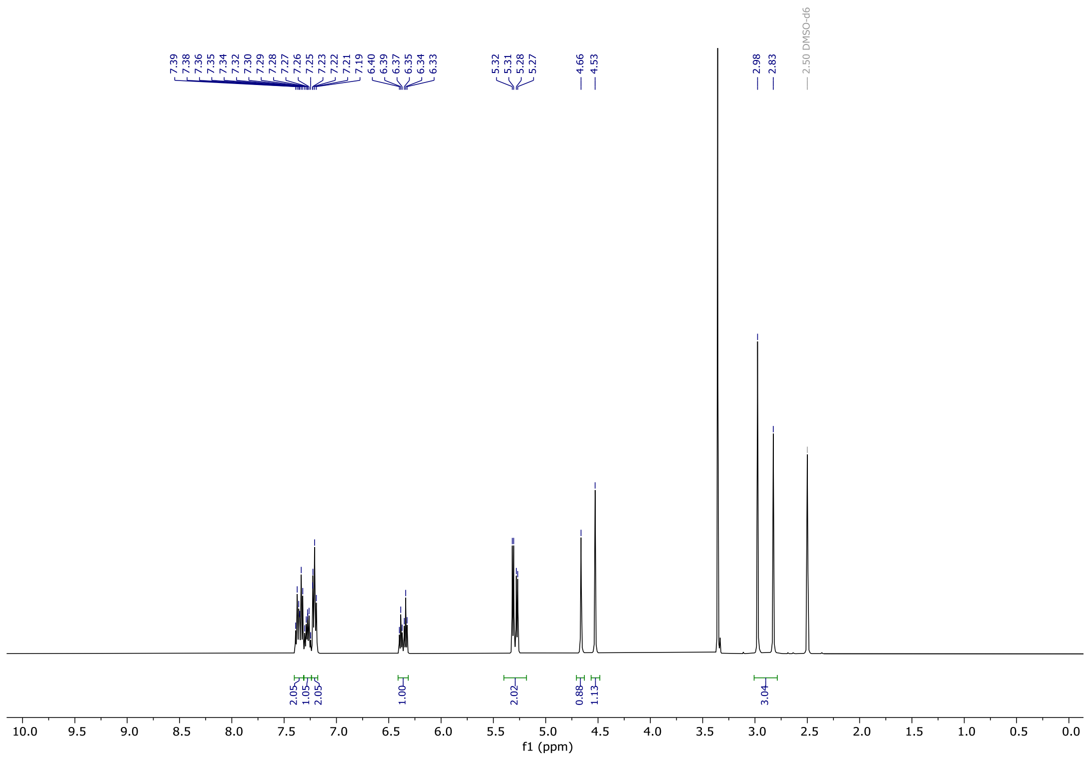

^{13}C-NMR (101 MHz, DMSO-d_6, 298 K):

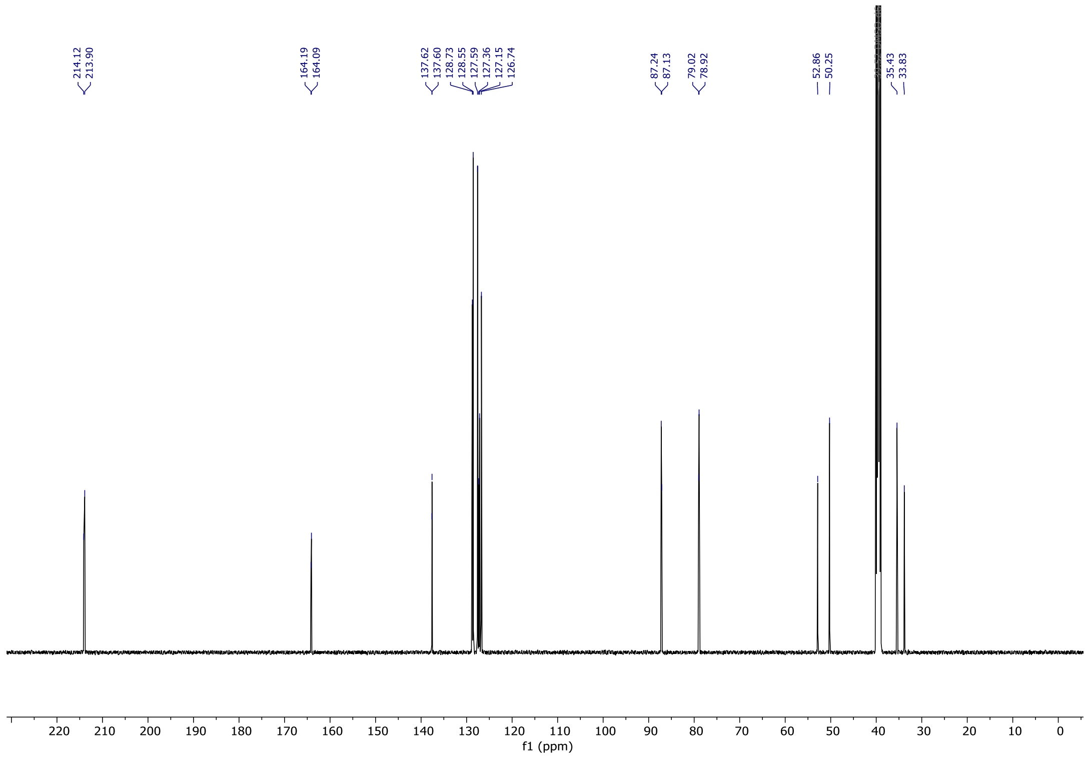

N-Benzyl-*N*-methylbuta-2,3-dienamide-d_1 (7f-d_1)

^{1}H-NMR (500 MHz, DMSO-d_6, 298 K):

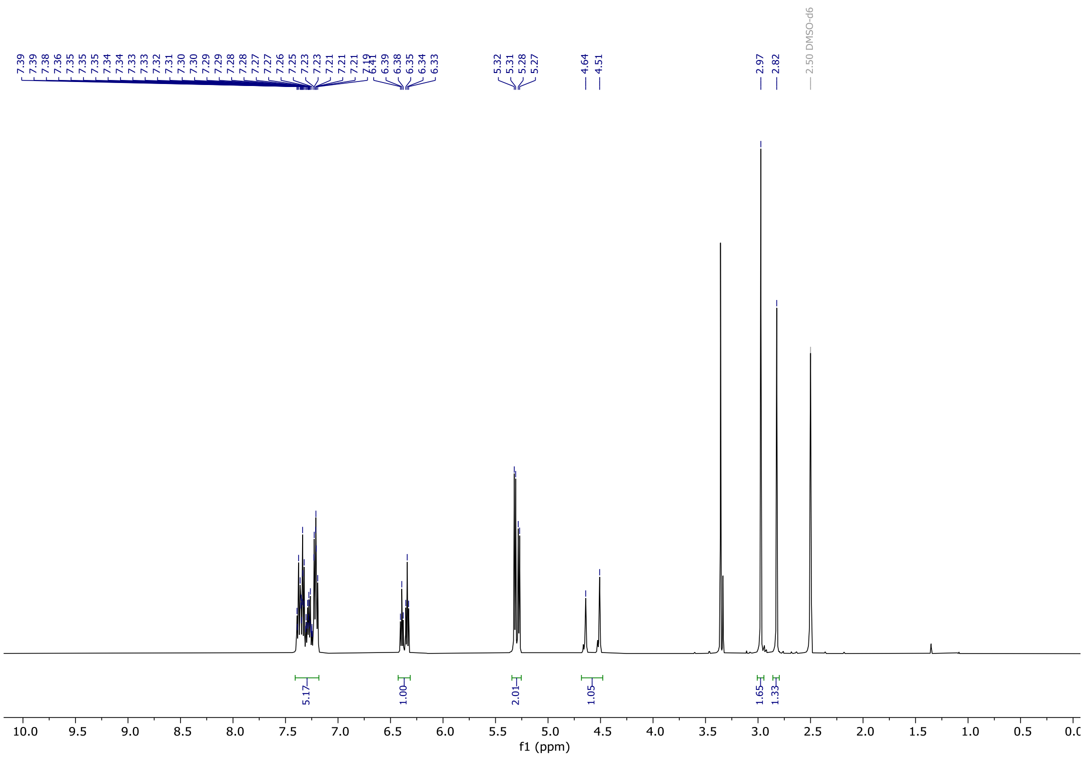

^{13}C-NMR (126 MHz, DMSO-d_6, 298 K):

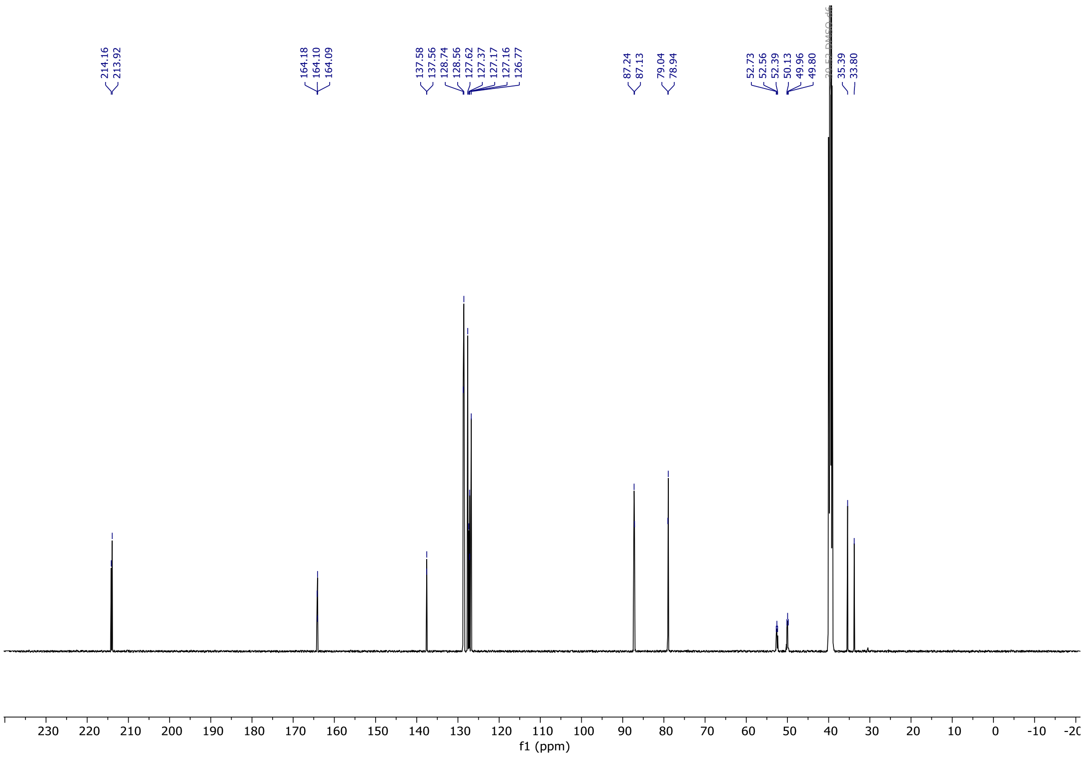

N-Benzyl-*N*-methylbuta-2,3-dienamide-d_2 (7f-d_2)

^{1}H-NMR (500 MHz, DMSO-d_6, 298 K):

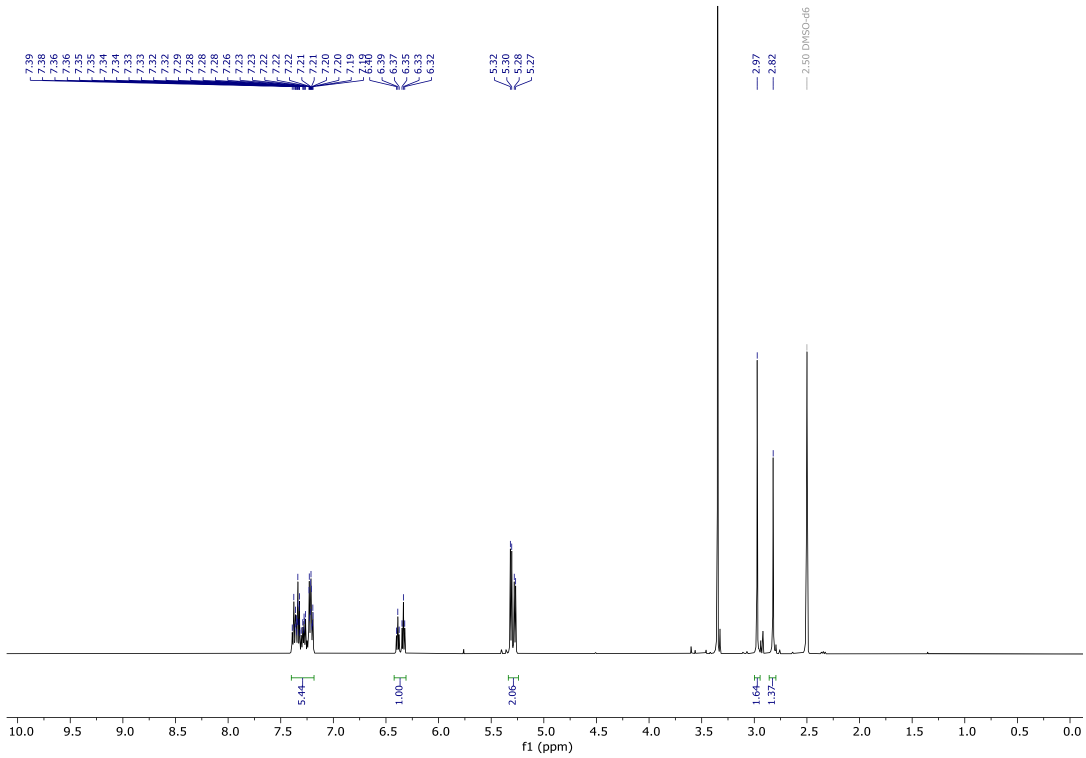

^{13}C-NMR (126 MHz, DMSO-d_6, 298 K):

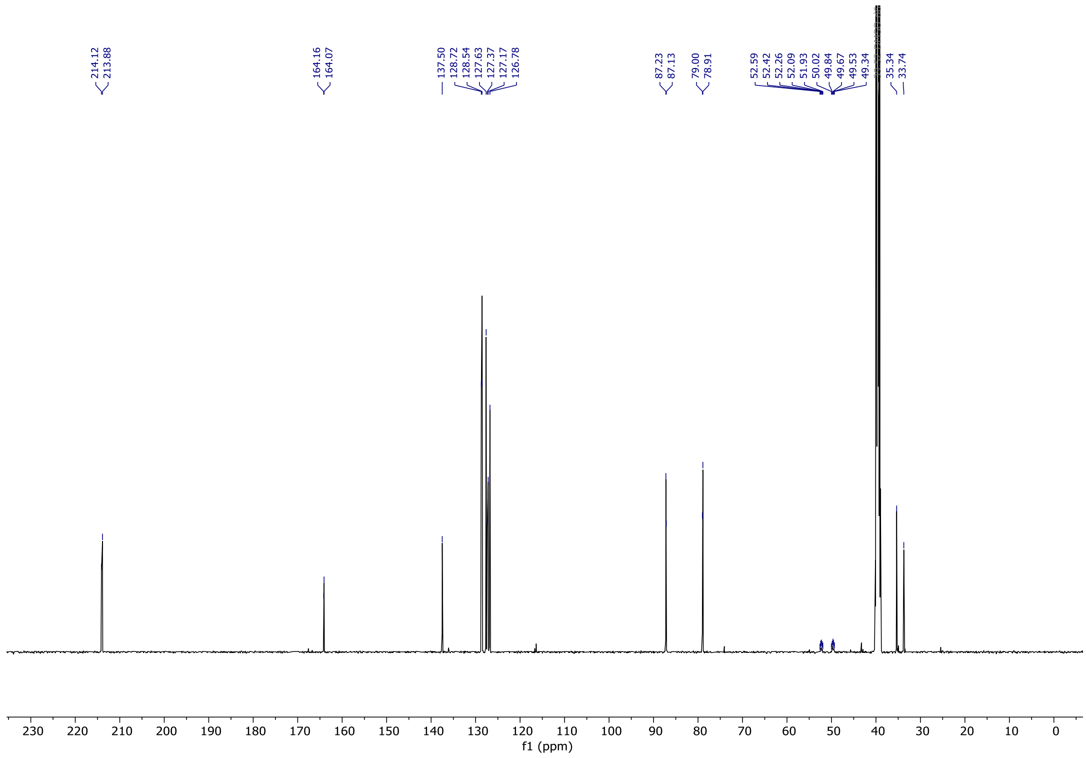

N-(4-Fluorobenzyl)-*N*-methylbuta-2,3-dienamide (7g)

^{1}H-NMR (400 MHz, DMSO-d_6, 298 K):

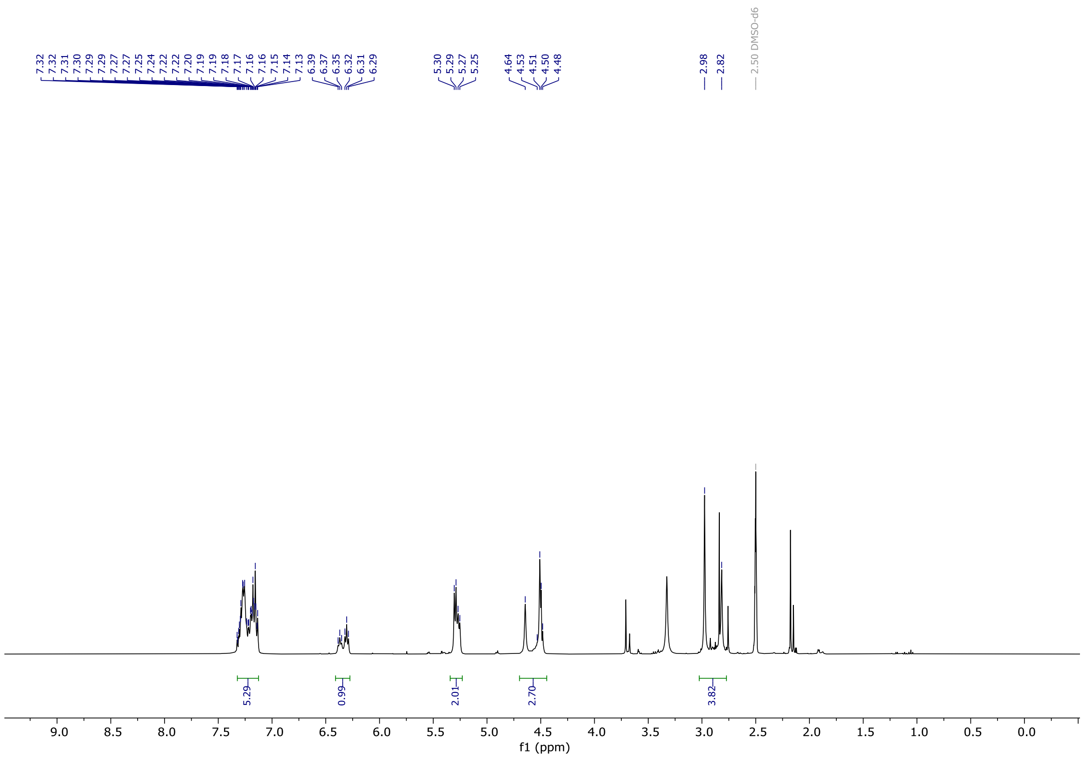

^{13}C-NMR (101 MHz, DMSO-d_6, 298 K):

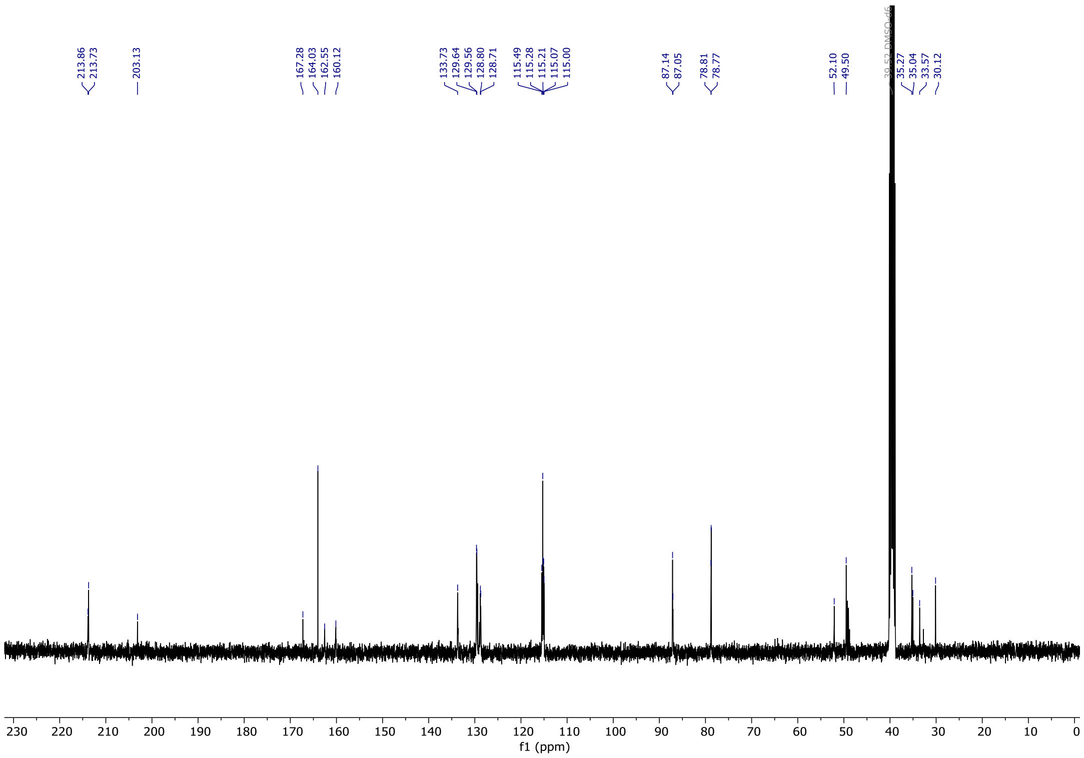

***N*-Methyl-*N*-(naphthalen-2-ylmethyl)buta-2,3-dienamide (7h)**

^{1}H-NMR (500 MHz, DMSO-d_6, 298 K):

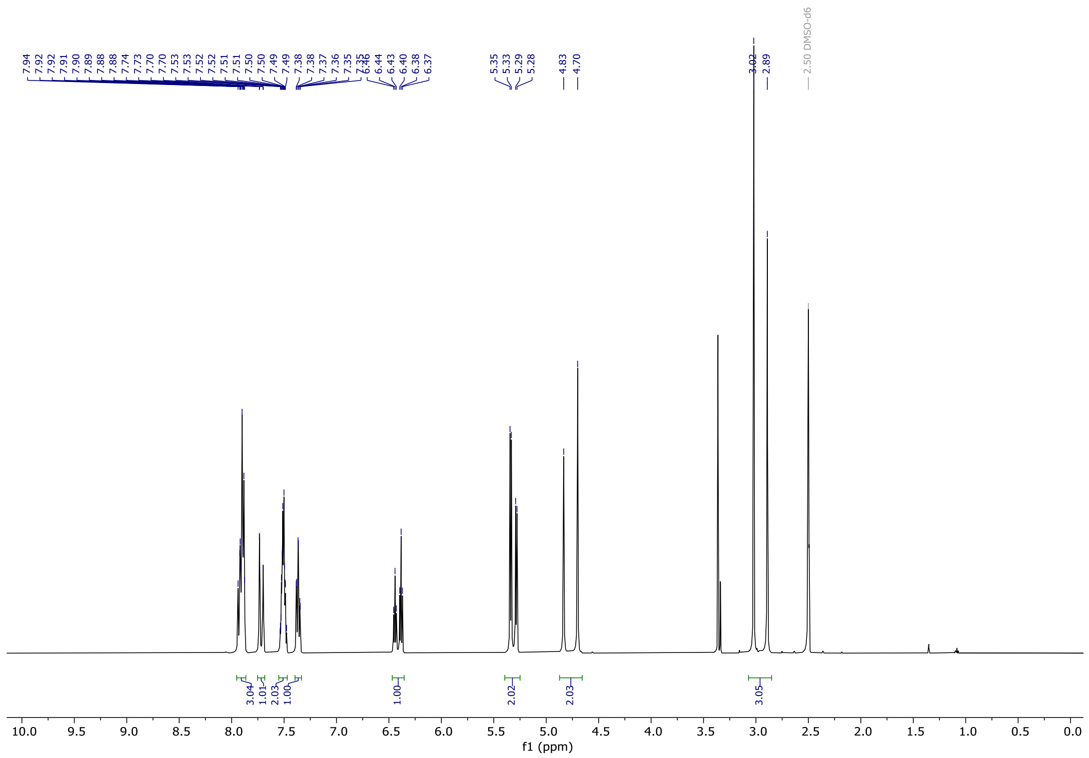

^{13}C-NMR (126 MHz, DMSO-d_6, 298 K):

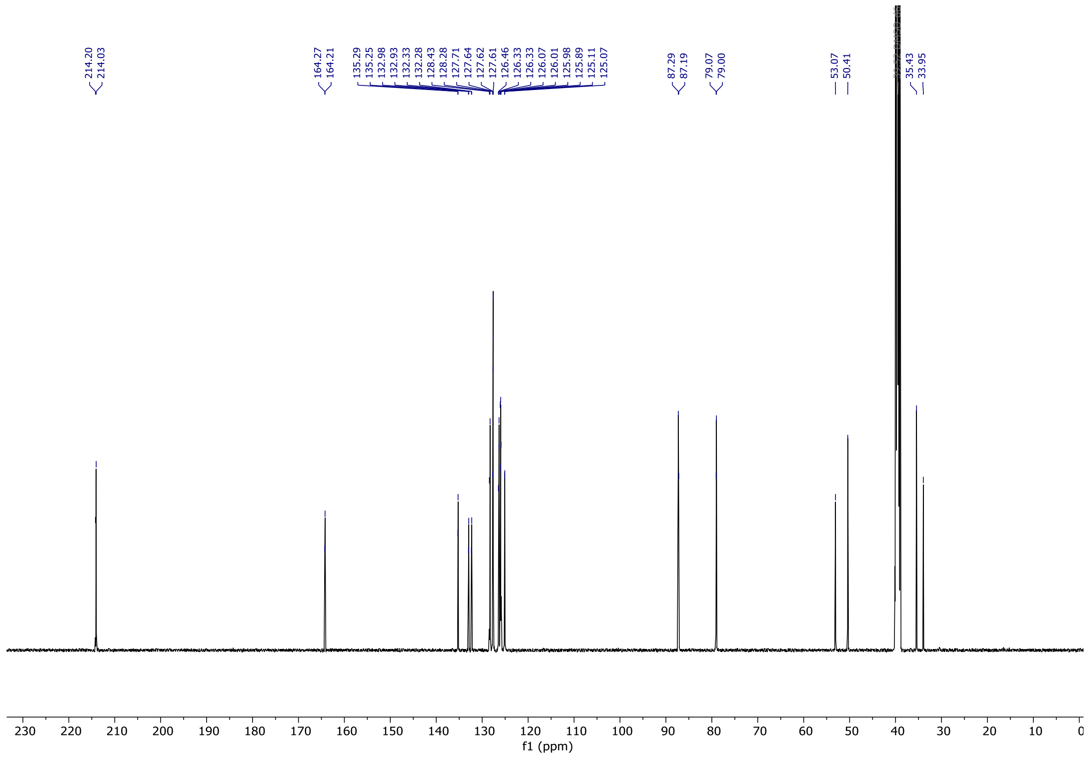

N-(4-Bromobenzyl)-*N*-methylbuta-2,3-dienamide (7i)

^{1}H-NMR (500 MHz, DMSO-d_6, 298 K):

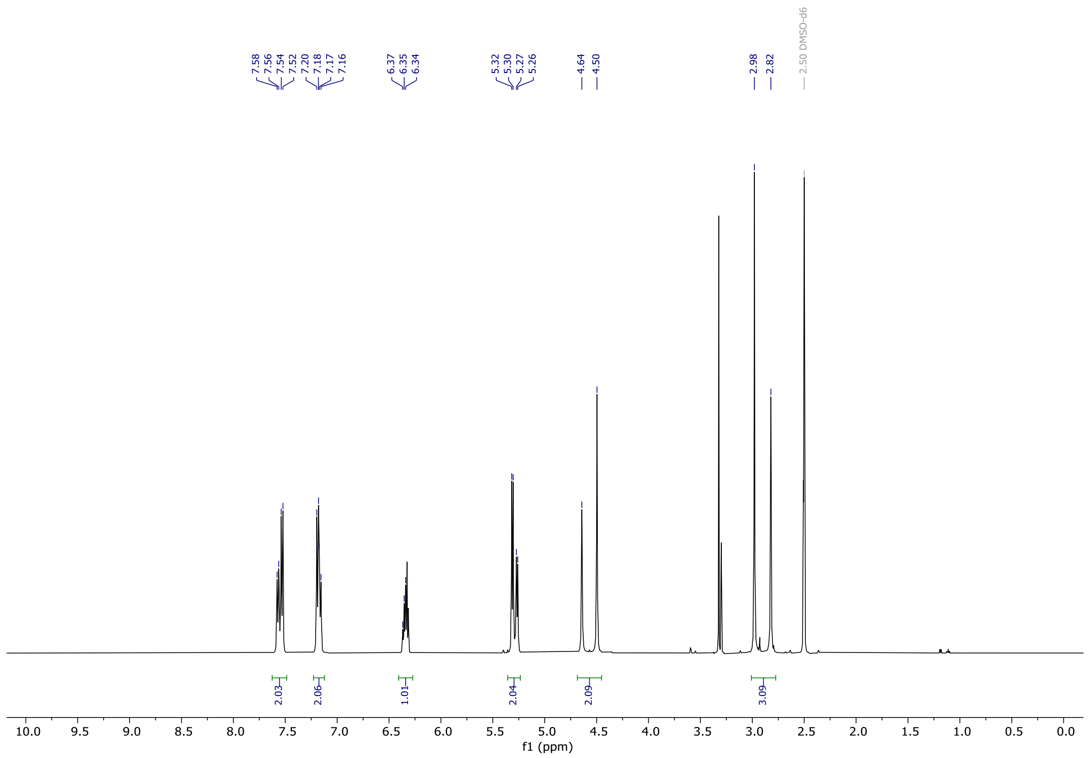

^{13}C-NMR (126 MHz, DMSO-d_6, 298 K):

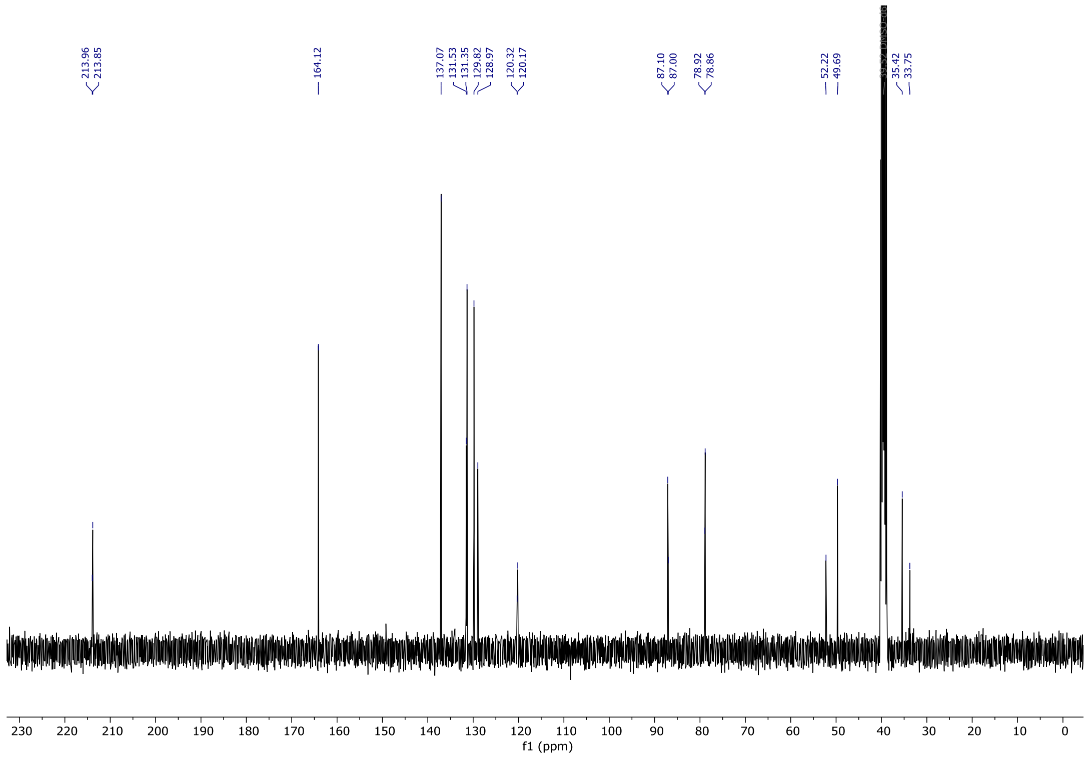

***N*-Benzyl-*N*-(*tert*-butyl)buta-2,3-dienamide (7j)**

^{1}H-NMR (400 MHz, DMSO-d_6, 298 K):

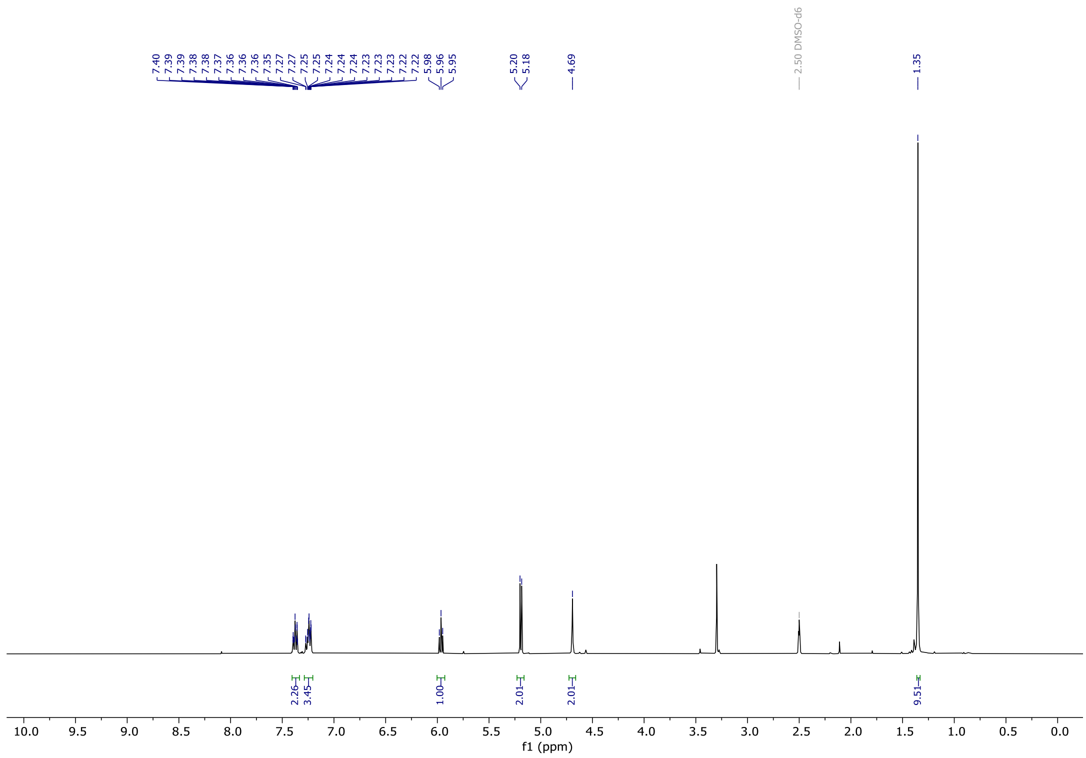

^{13}C-NMR (101 MHz, DMSO-d_6, 298 K):

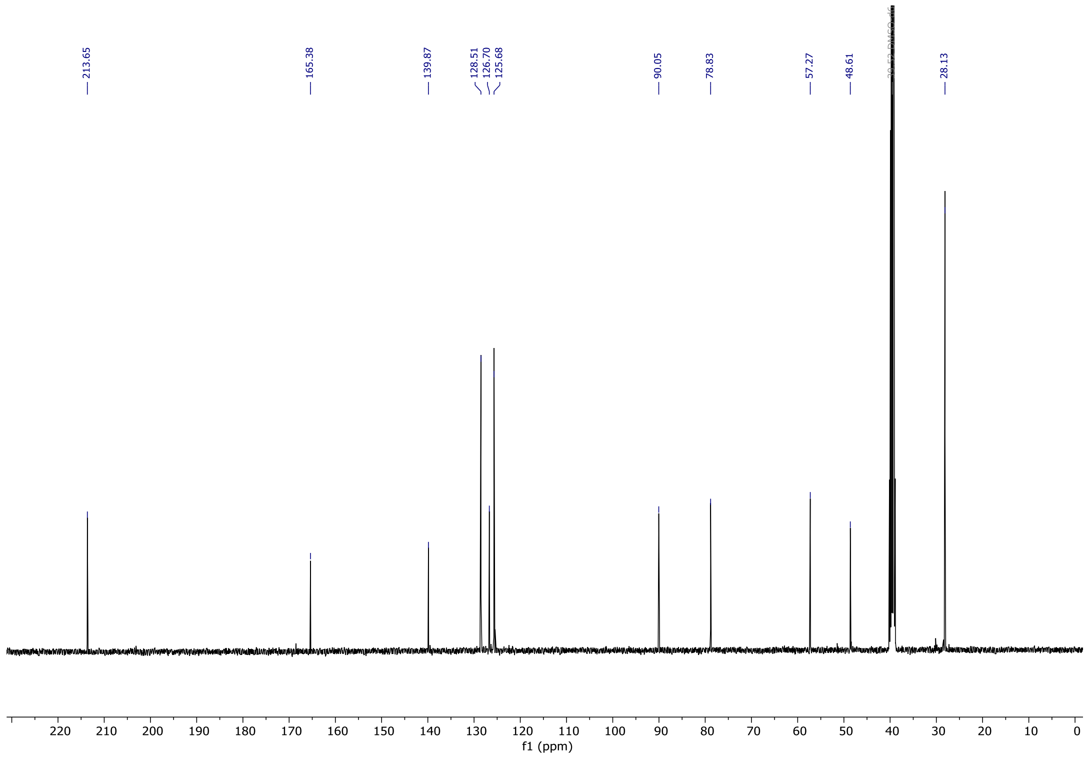

***N*-Benzyl-*N*-isopropylbuta-2,3-dienamide (7k)**

^{1}H-NMR (500 MHz, DMSO-d_6, 298 K):

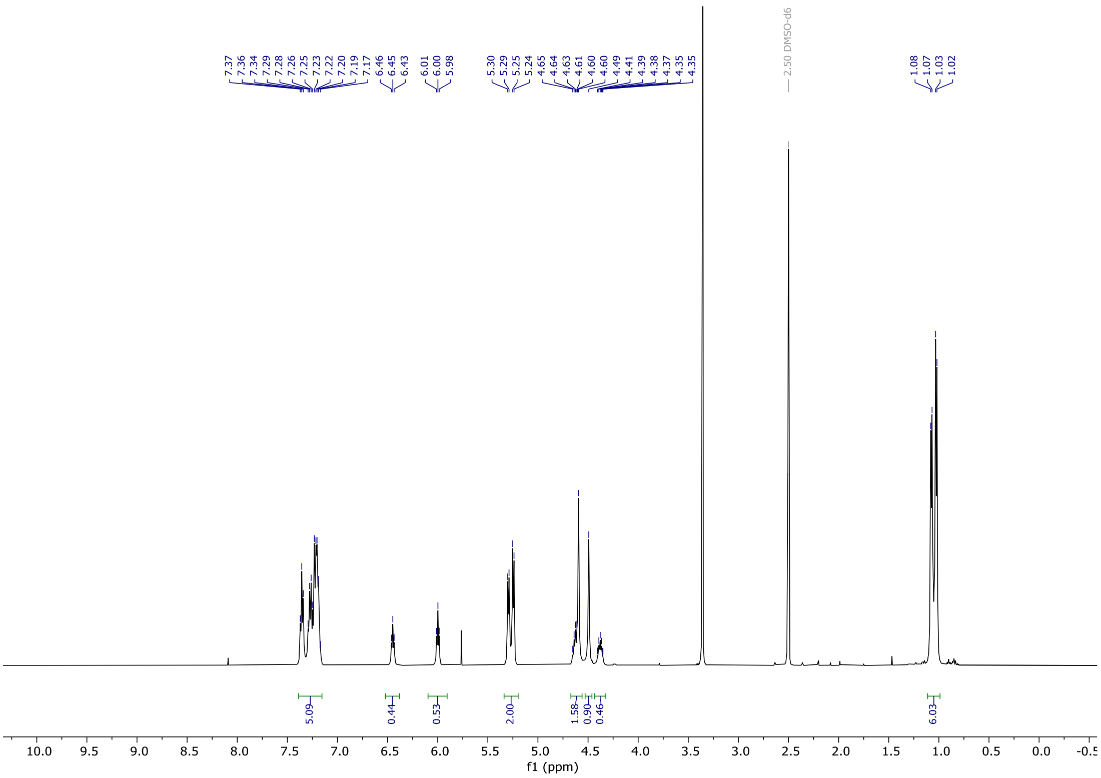

^{13}C-NMR (126 MHz, DMSO-d_6, 298 K):

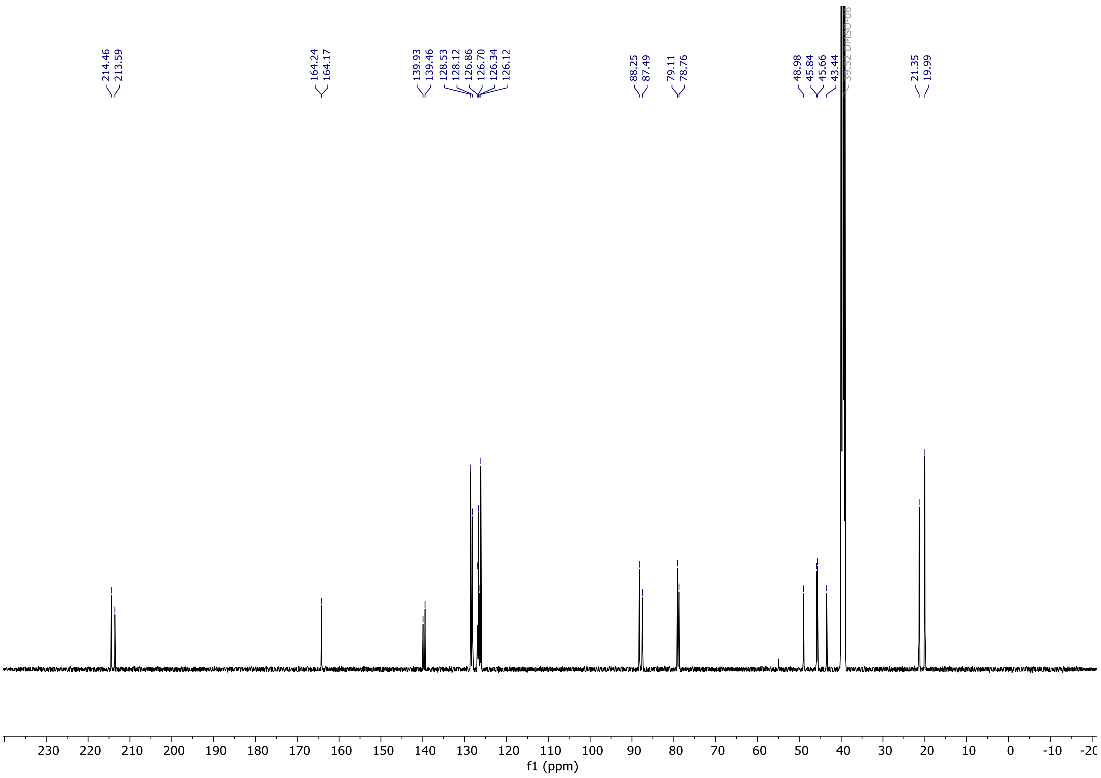

***N*-benzyl-*N*-[(trimethylsilyl)methyl]buta-2,3-dienamide (7l)**

^{1}H-NMR (400 MHz, DMSO-d_6, 298 K):

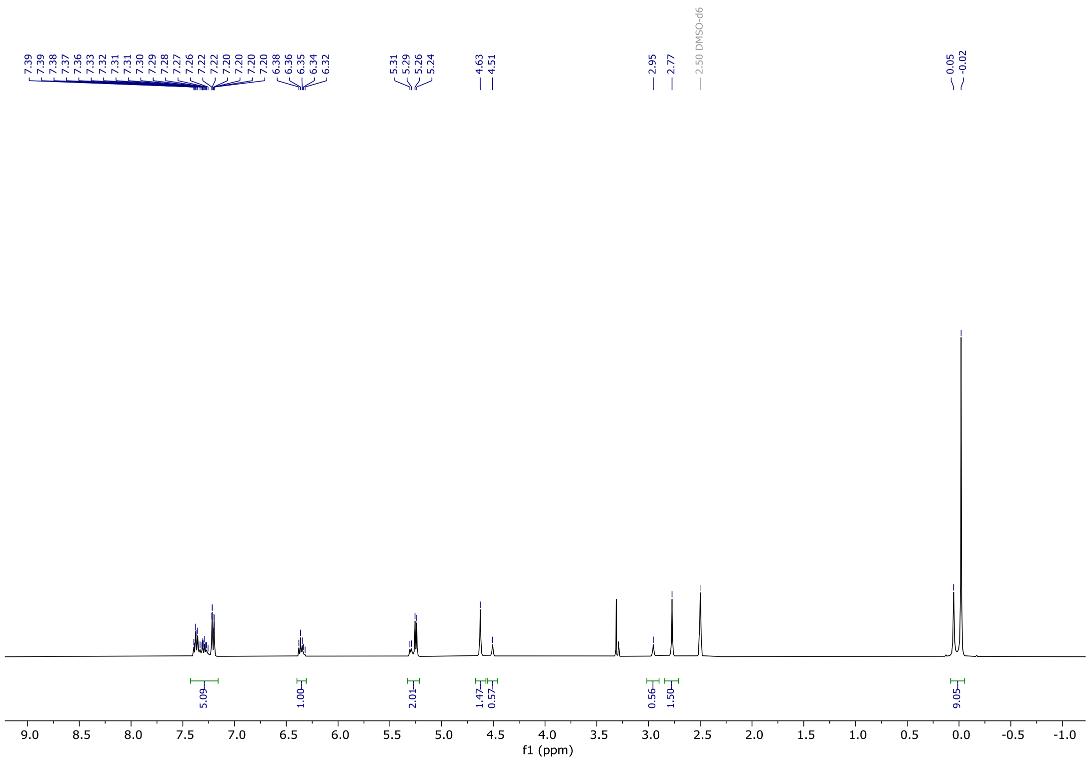

^{13}C-NMR (101 MHz, DMSO-d_6, 298 K):

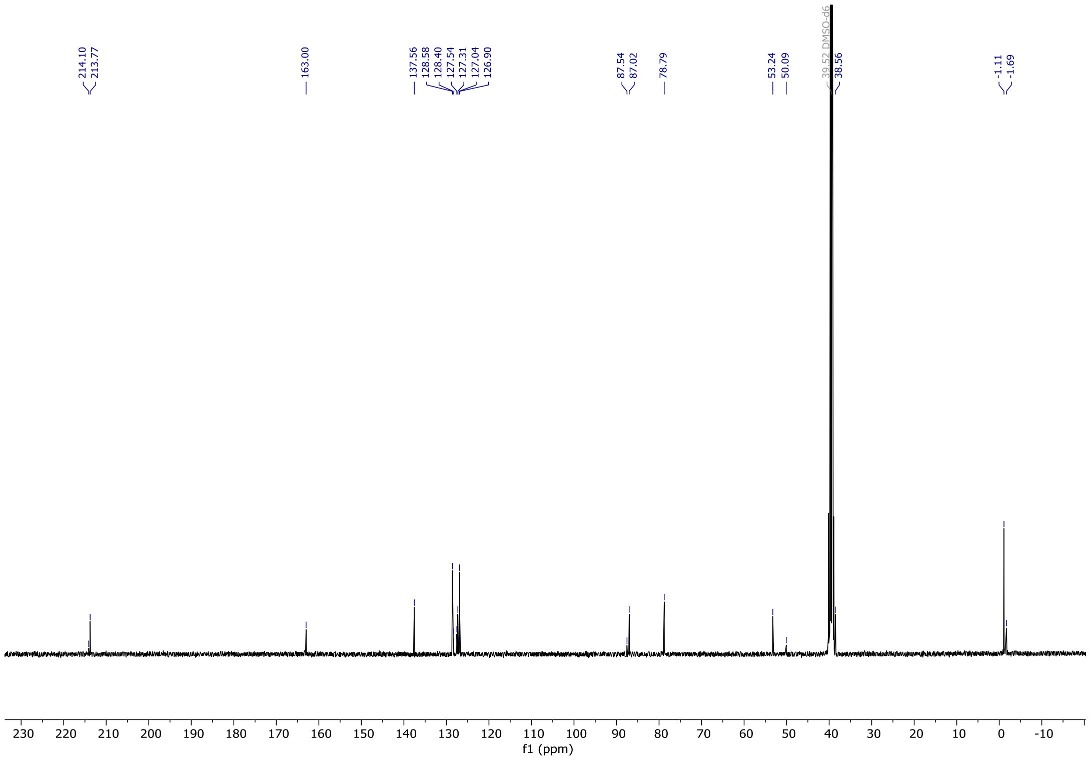

***N*-benzyl-*N*-[2-(benzyloxy)ethyl]buta-2,3-dienamide (7m)**

^{1}H-NMR (400 MHz, DMSO-d_6, 298 K):

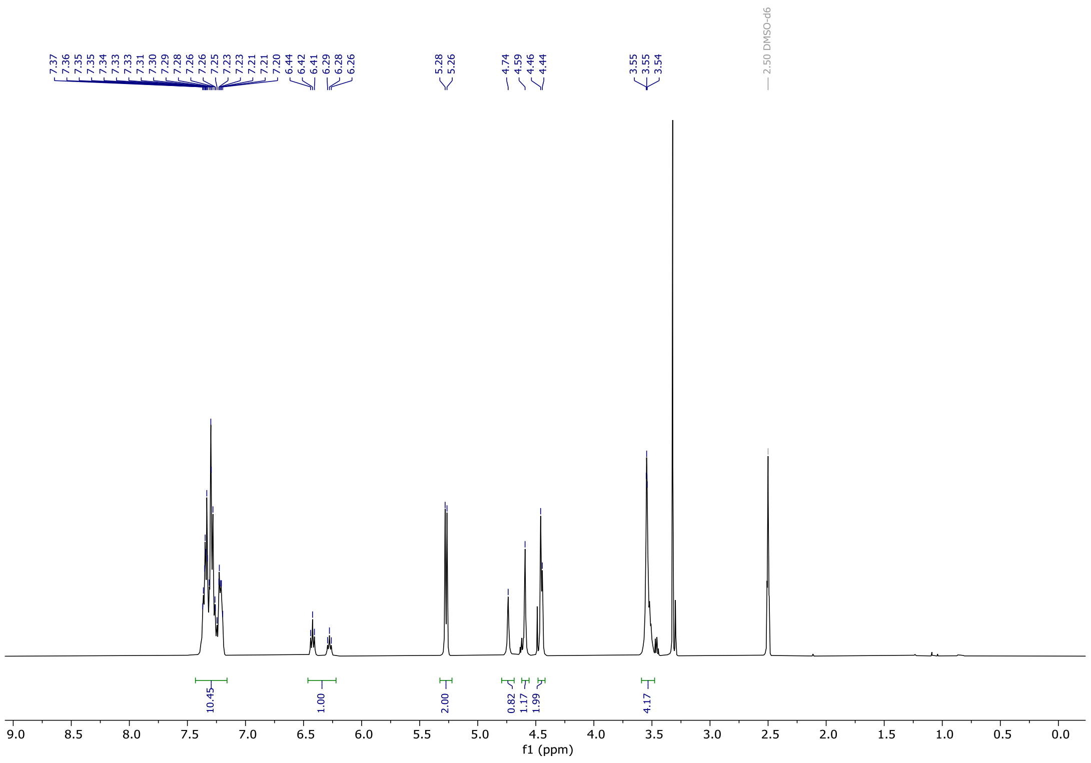

^{13}C-NMR (101 MHz, DMSO-d_6, 298 K):

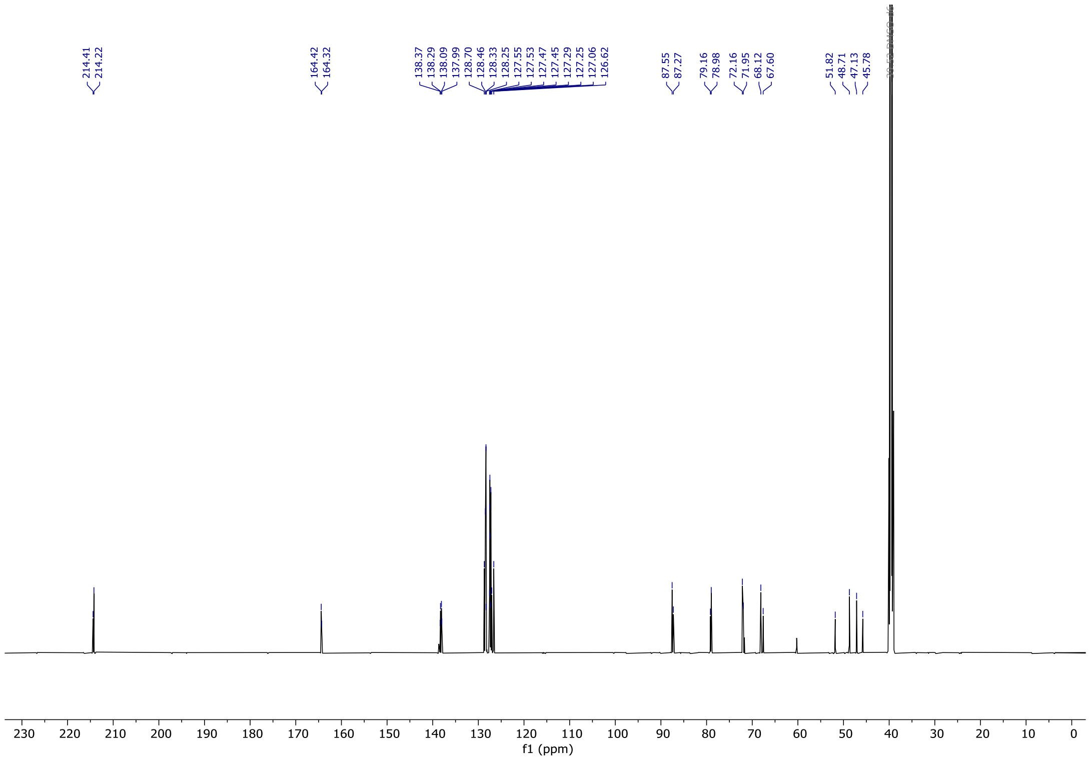

1-(Thiazolidin-3’-yl)buta-2,3-dien-1-one (7o)

^{1}H-NMR (500 MHz, DMSO-d_6, 298 K):

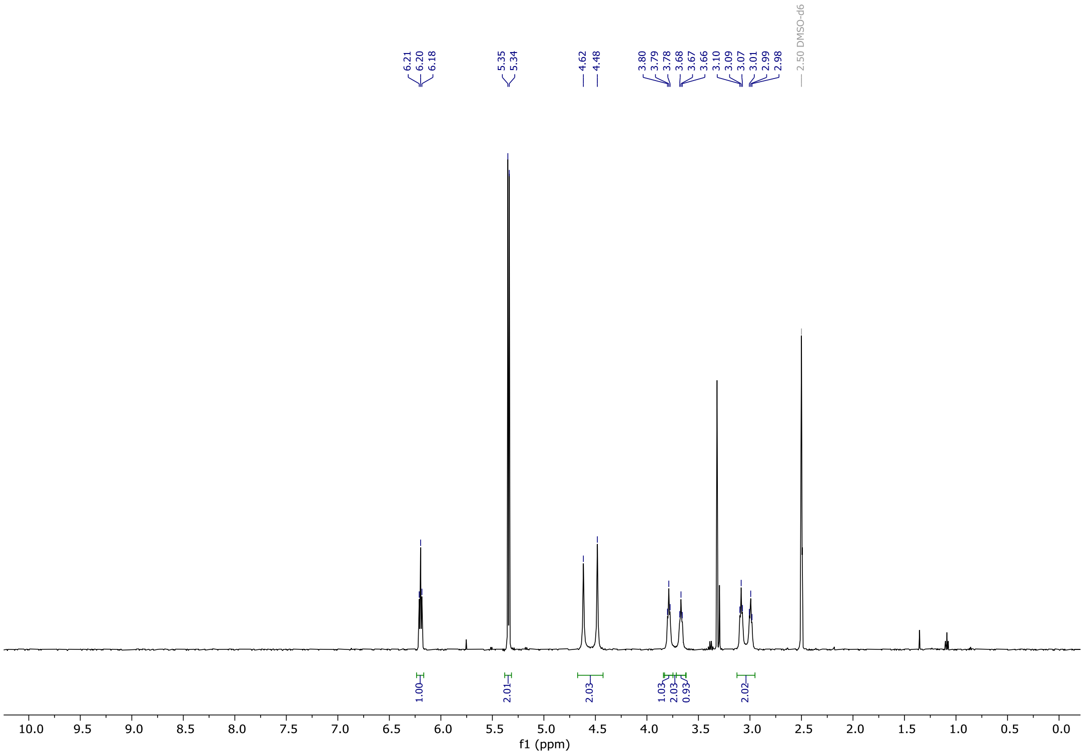

^{13}C-NMR (101 MHz, DMSO-d_6, 298 K):

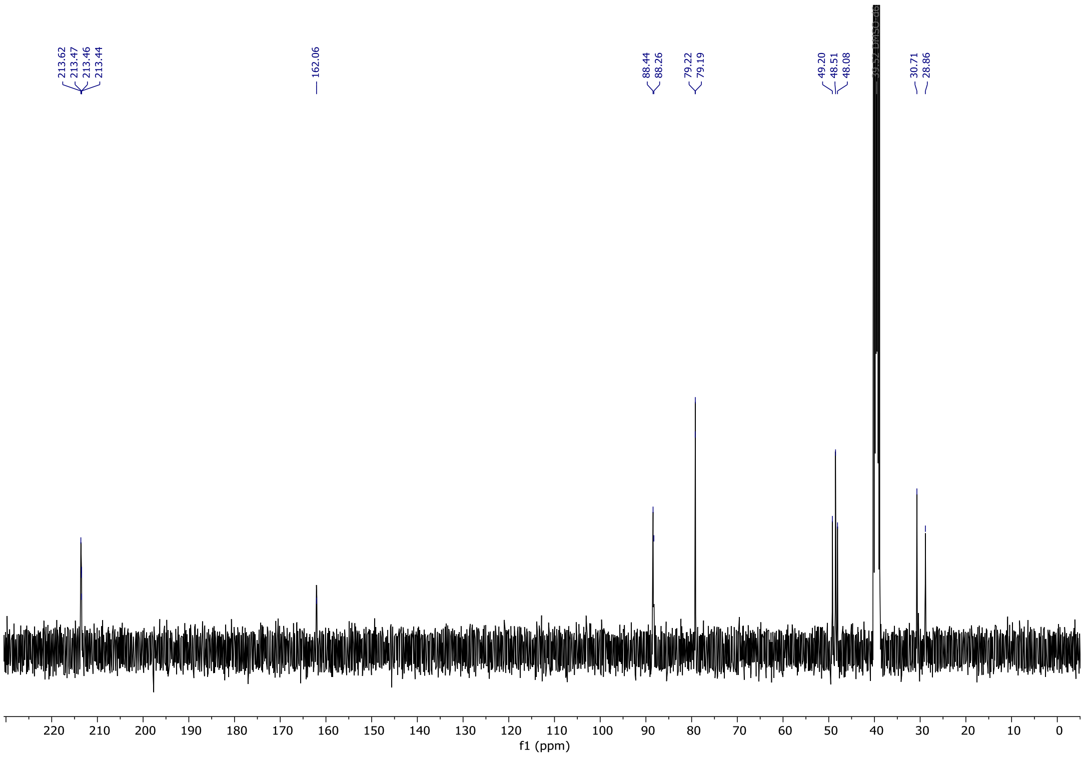

N,N-Dibenzyl-5,5-dimethylhexa-2,3-dienamide (11)

^{1}H-NMR (400 MHz, DMSO-d_6, 298 K):

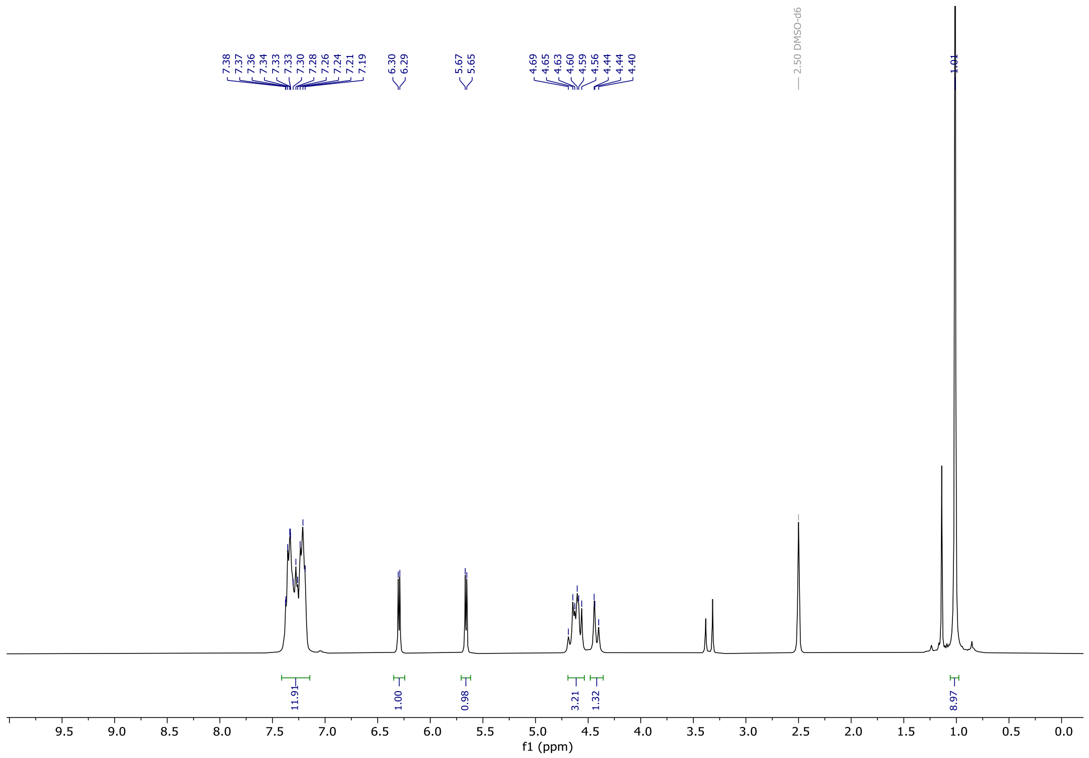

^{13}C-NMR (101 MHz, DMSO-d_6, 298 K):

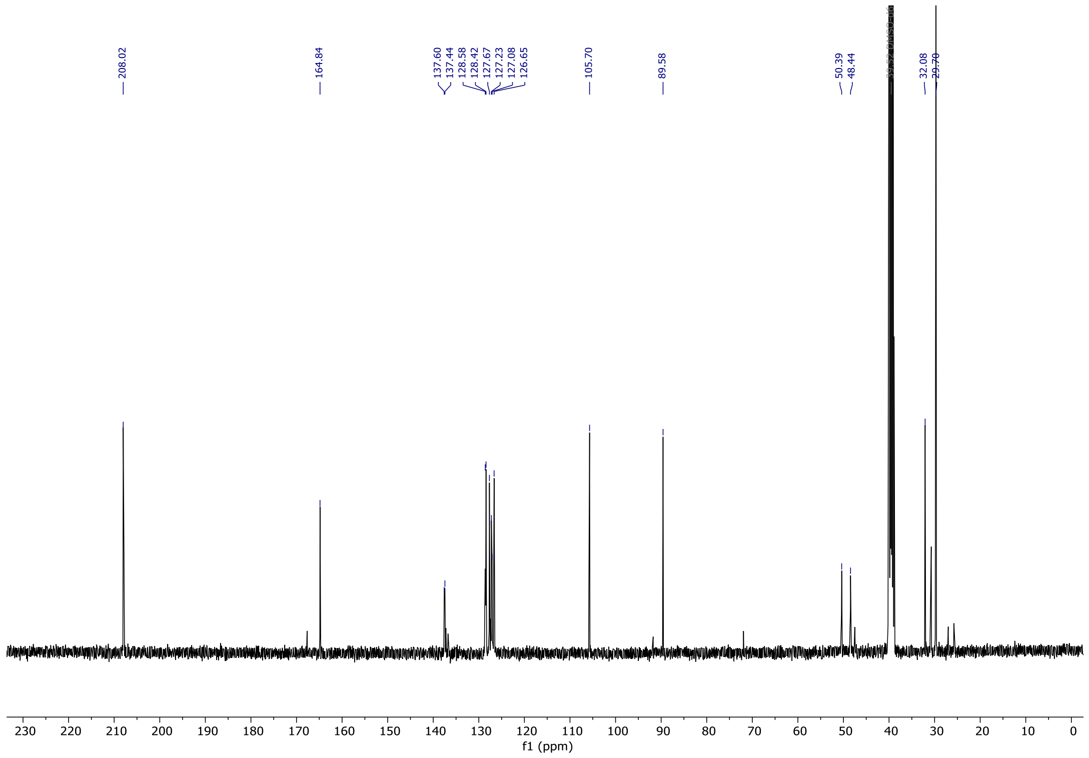

N-Benzhydryl-*N*-methylbuta-2,3-dienamide (13b)

^{1}H-NMR (500 MHz, DMSO-d_6, 298 K):

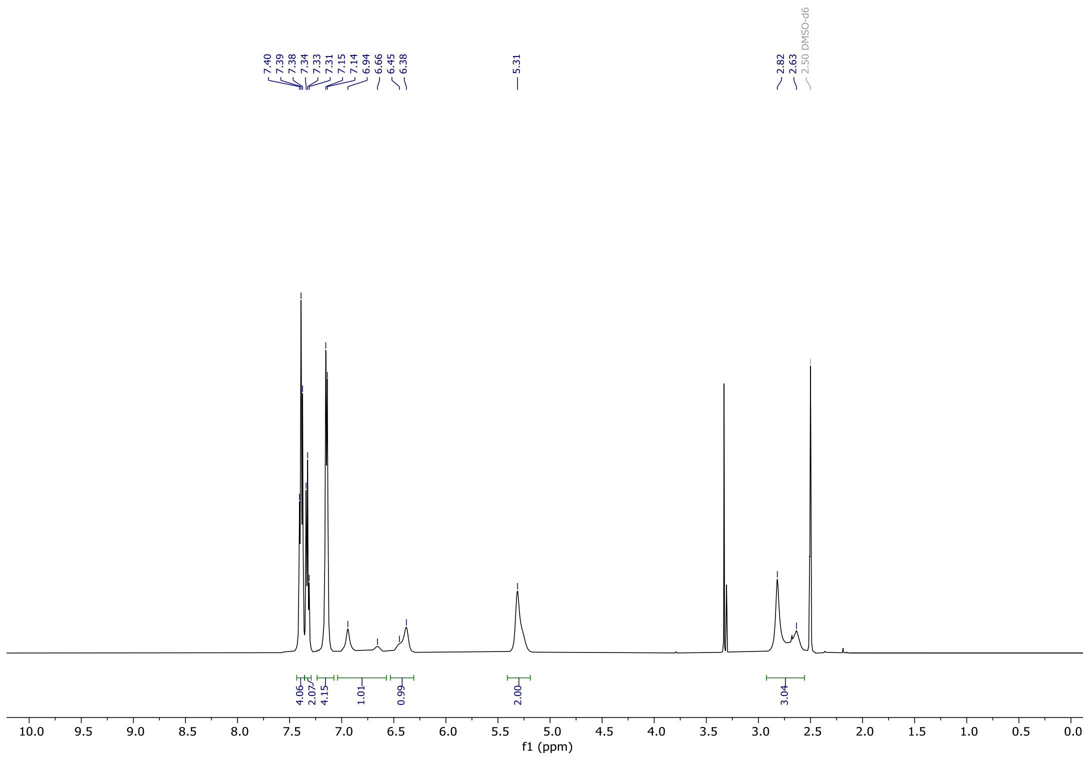

^{13}C-NMR (101 MHz, DMSO-d_6, 298 K):

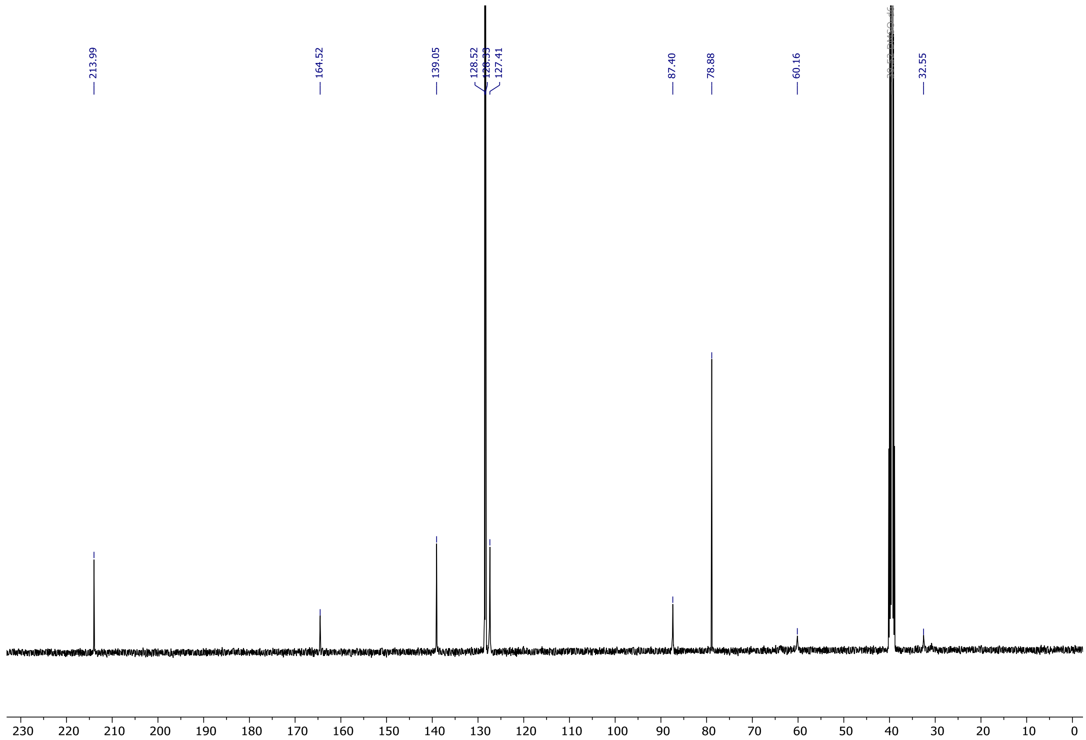

N,N-Diisopropylbuta-2,3-dienamide (13c)

^{1}H-NMR (500 MHz, DMSO-d_6, 298 K):

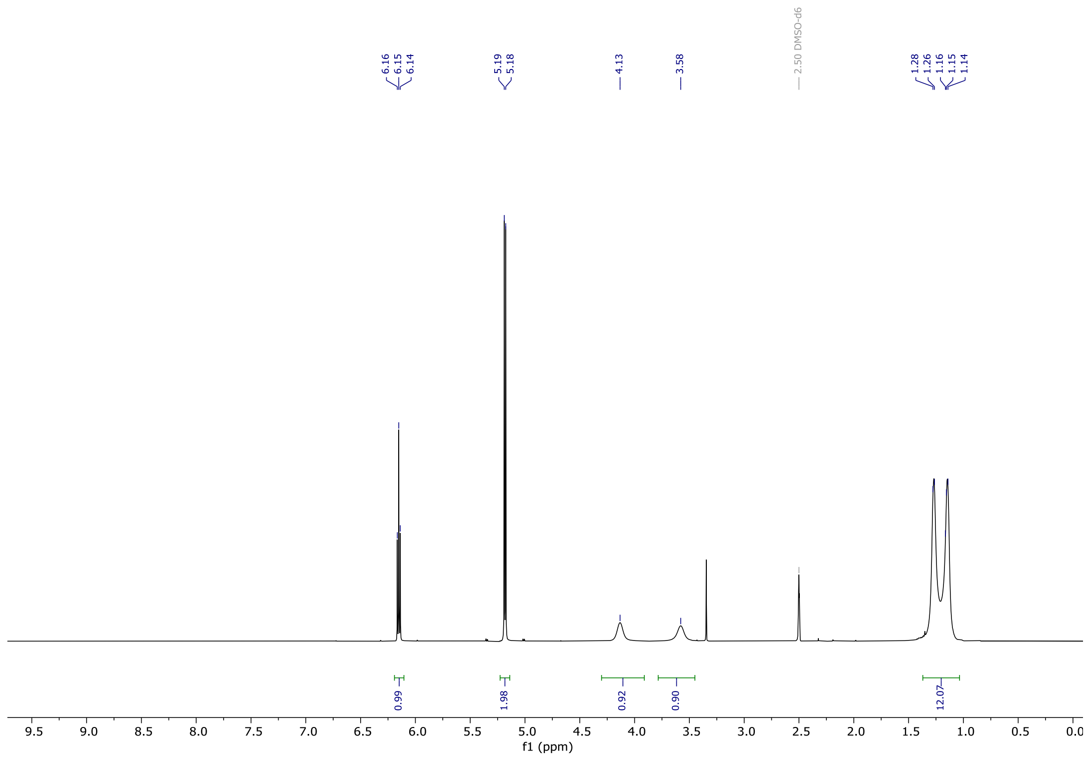

^{13}C-NMR (126 MHz, DMSO-d_6, 298 K):

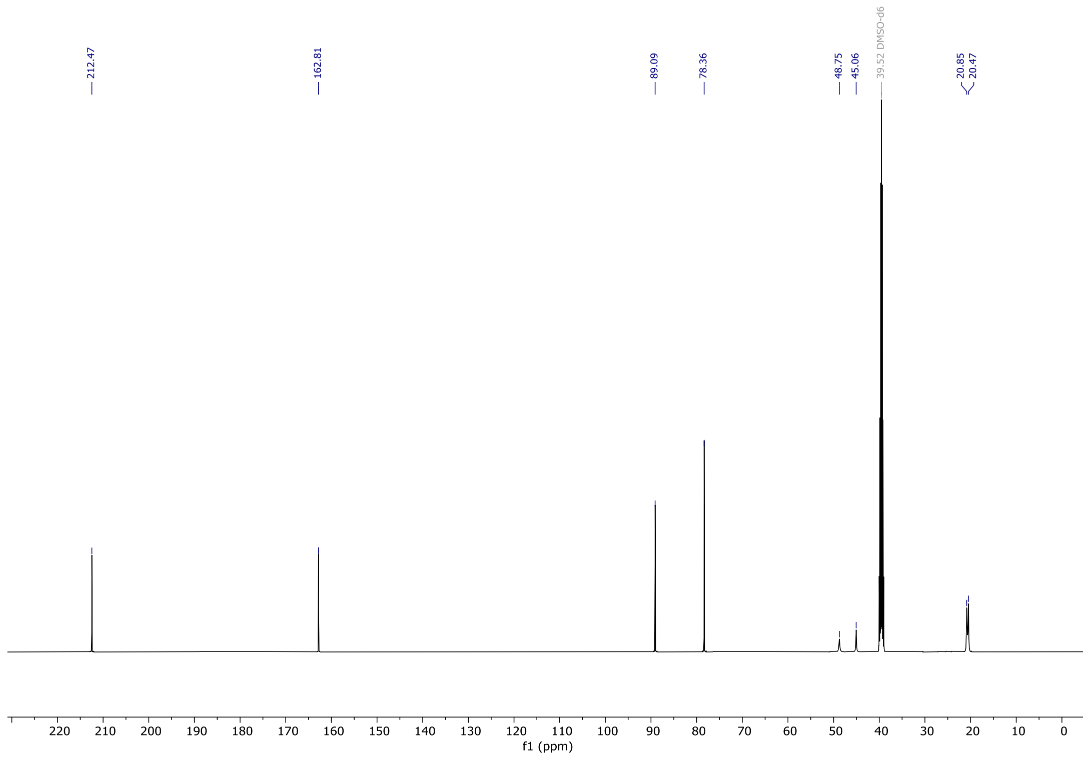

N,N-dicyclohexylbuta-2,3-dienamide (13d)

^{1}H-NMR (500 MHz, DMSO-d_6, 298 K):

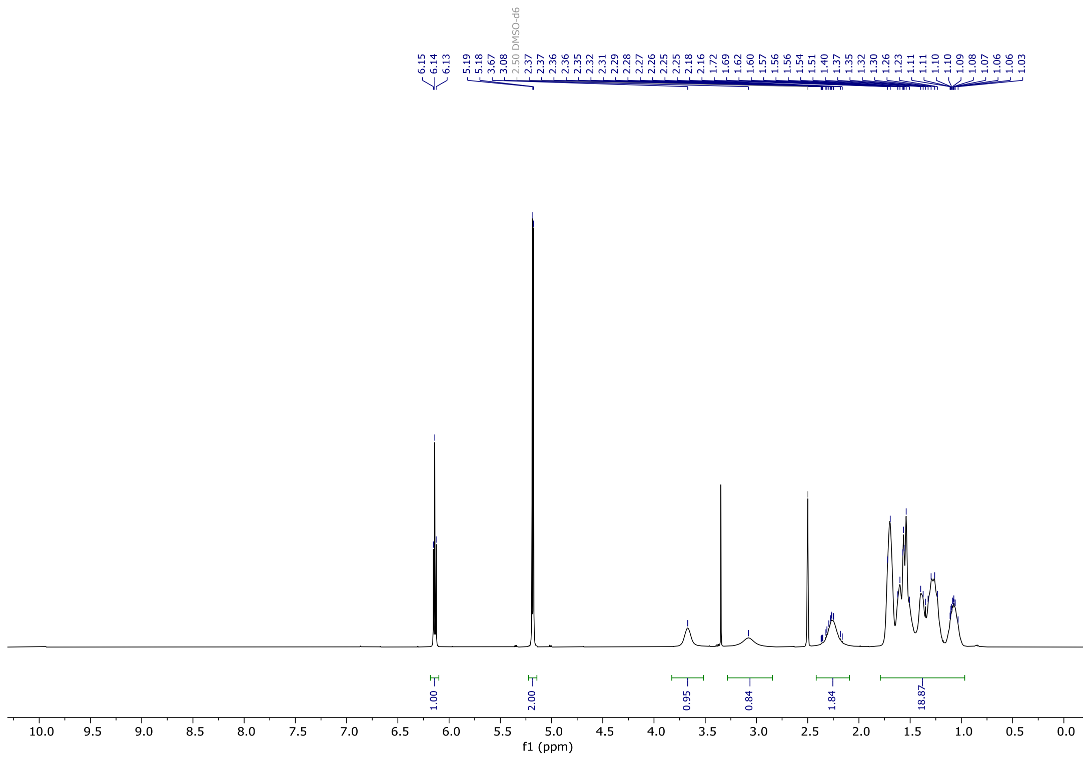

^{13}C-NMR (126 MHz, DMSO-d_6, 298 K):

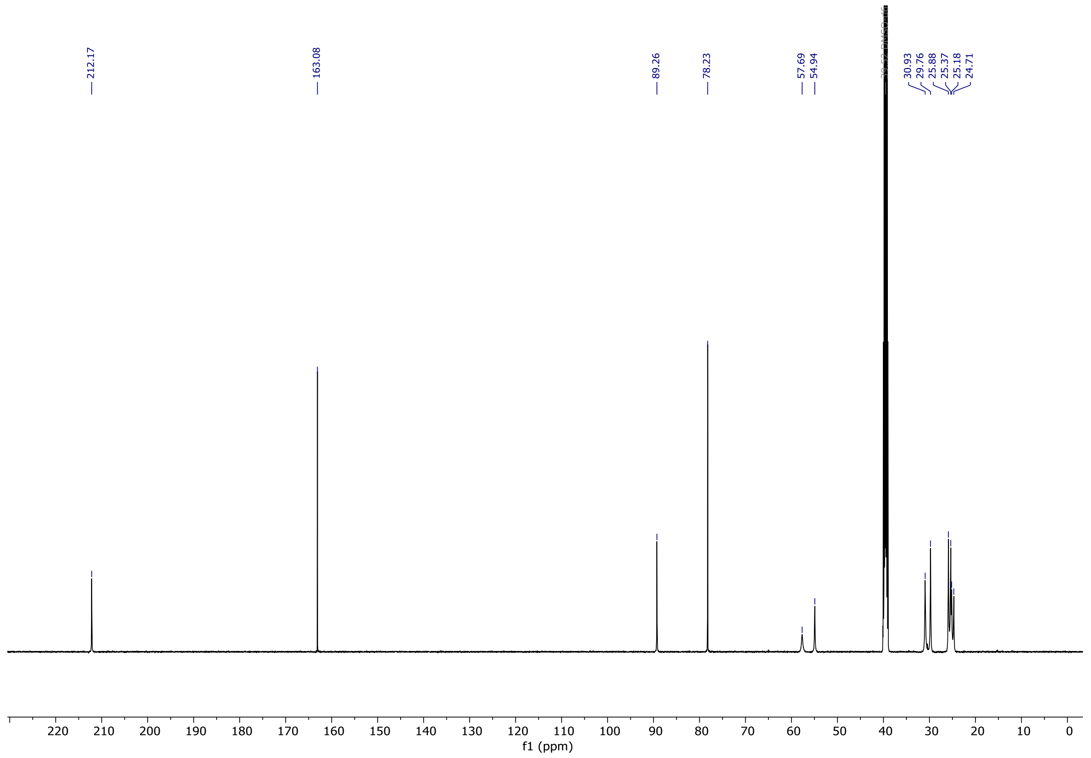

rac-1-benzyl-4-phenyl-3-vinylazetidin-2-one (8a)

^{1}H-NMR (500 MHz, $CDCl_3$, 298 K):

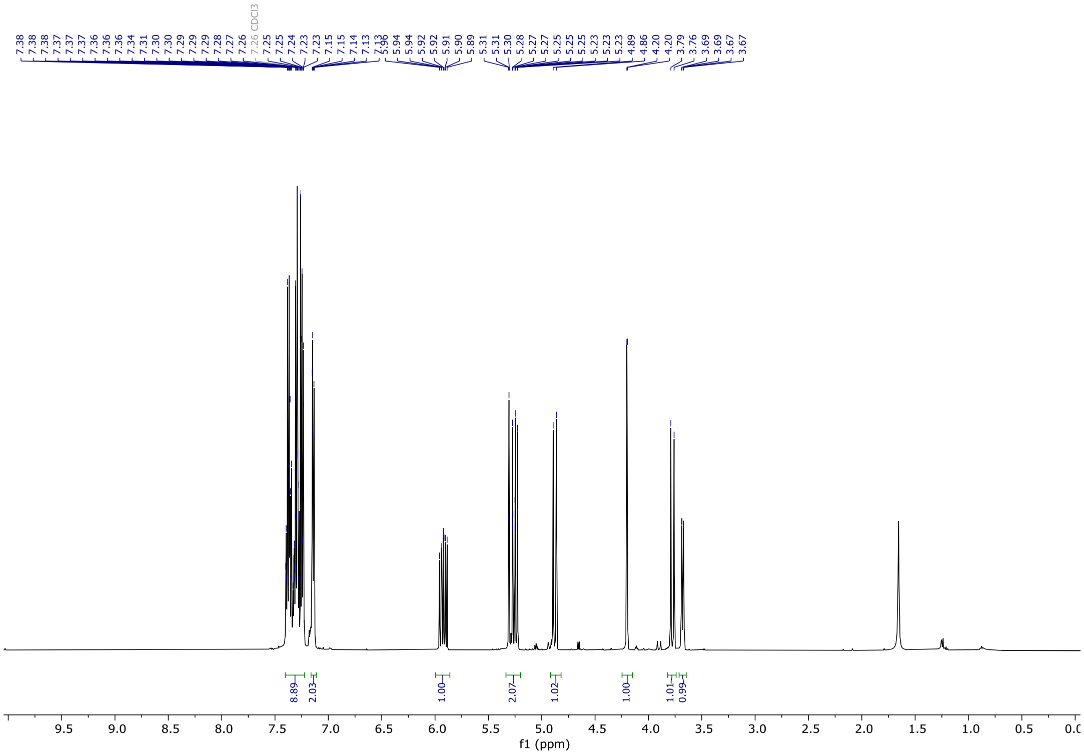

^{13}C-NMR (126 MHz, $CDCl_3$, 298 K):

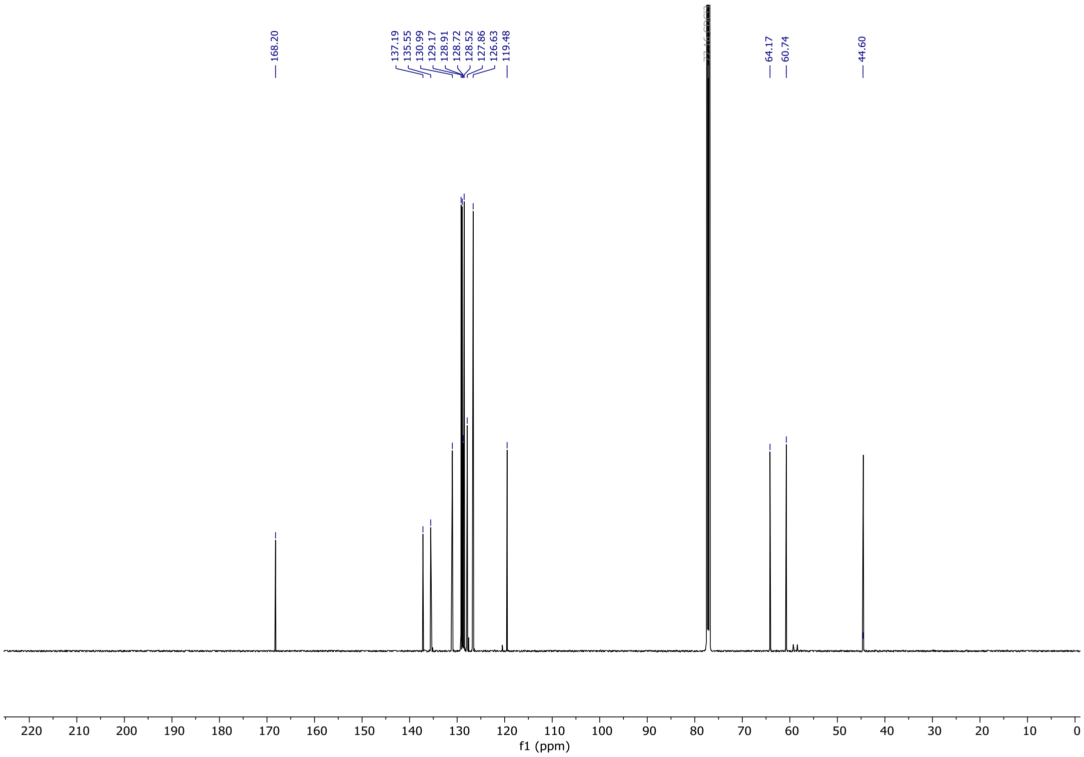

***rac*- 1-(3-Chlorobenzyl)-4-(3-chlorophenyl)-3-vinylazetidin-2-one (8b)**

^{1}H-NMR (500 MHz, $CDCl_3$, 298 K):

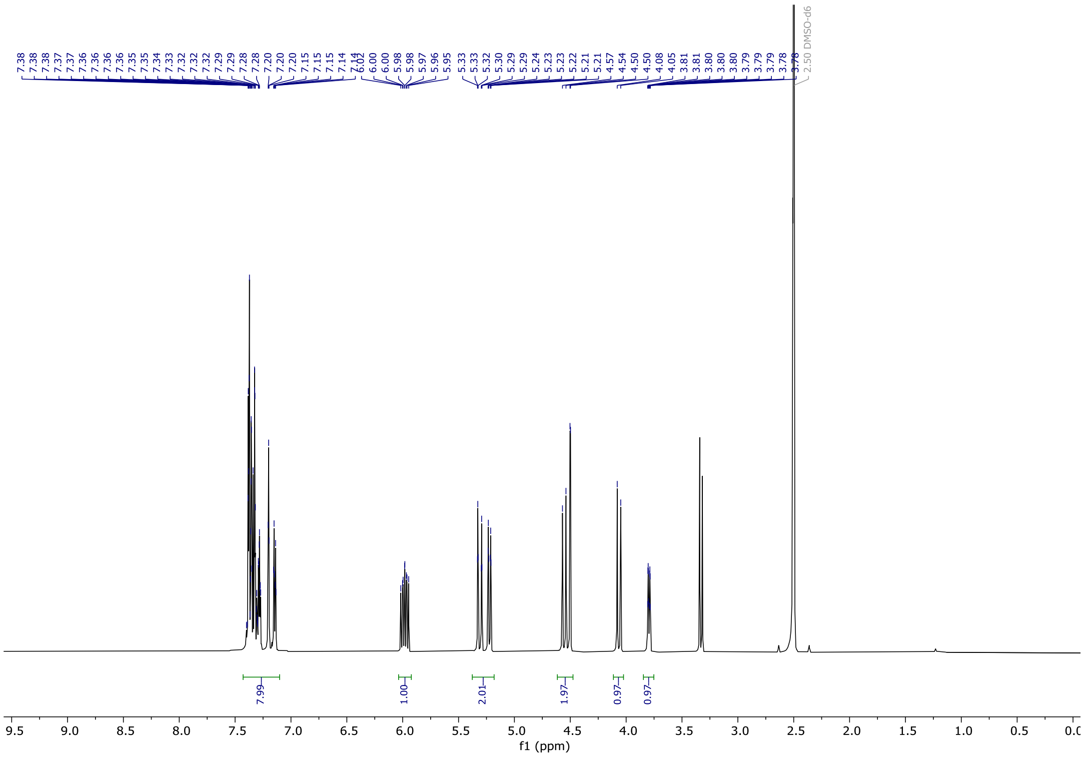

^{13}C-NMR (126 MHz, $CDCl_3$, 298 K):

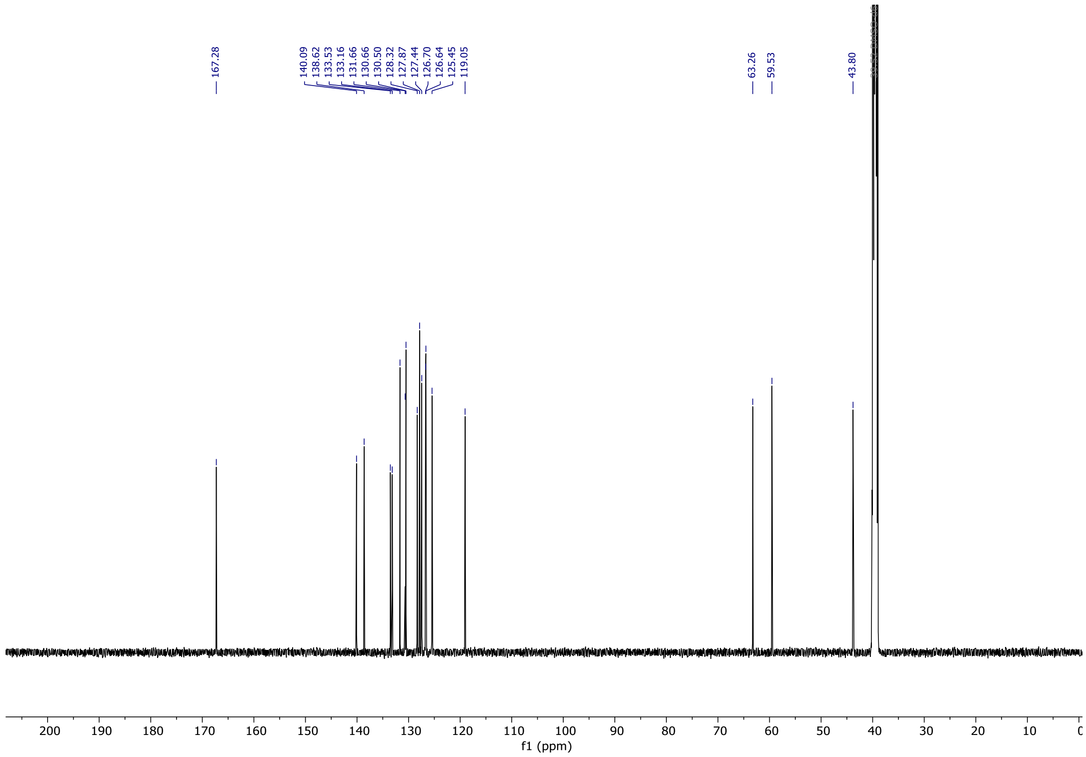

***rac*-1-(4-Fluorobenzyl)-4-(4-fluorophenyl)-3-vinylazetidin-2-one (8c)**

^{1}H-NMR (500 MHz, DMSO-d_6, 298 K):

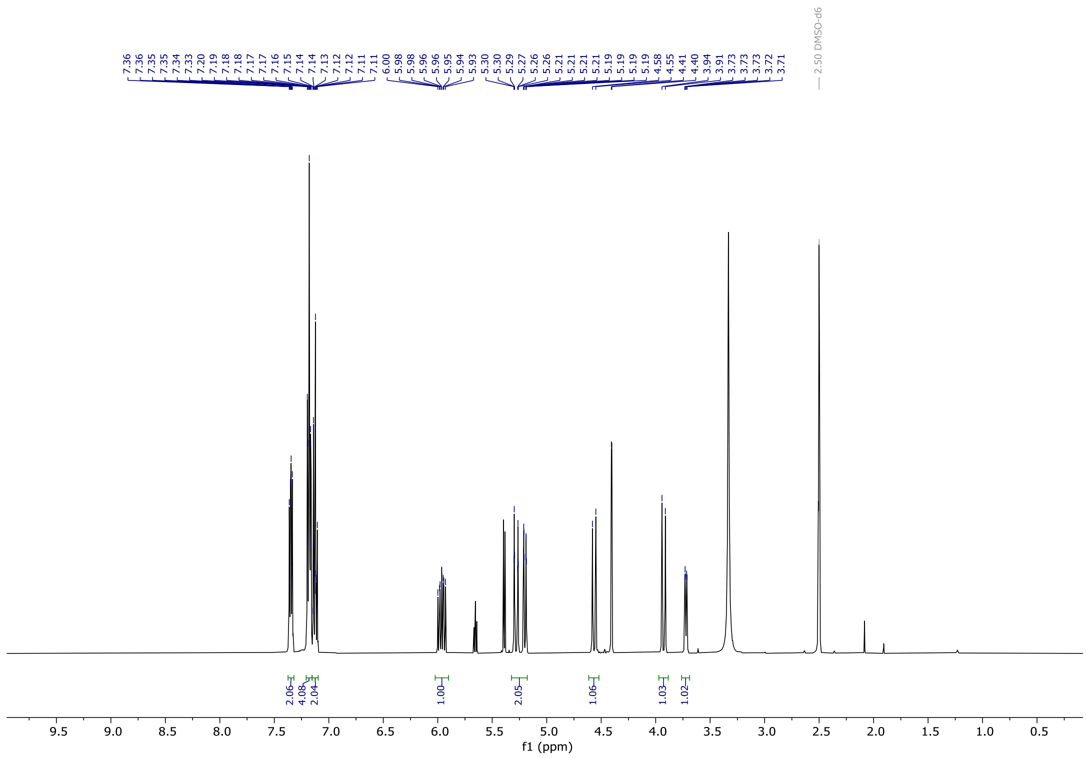

^{13}C-NMR (101 MHz, DMSO-d_6, 298 K):

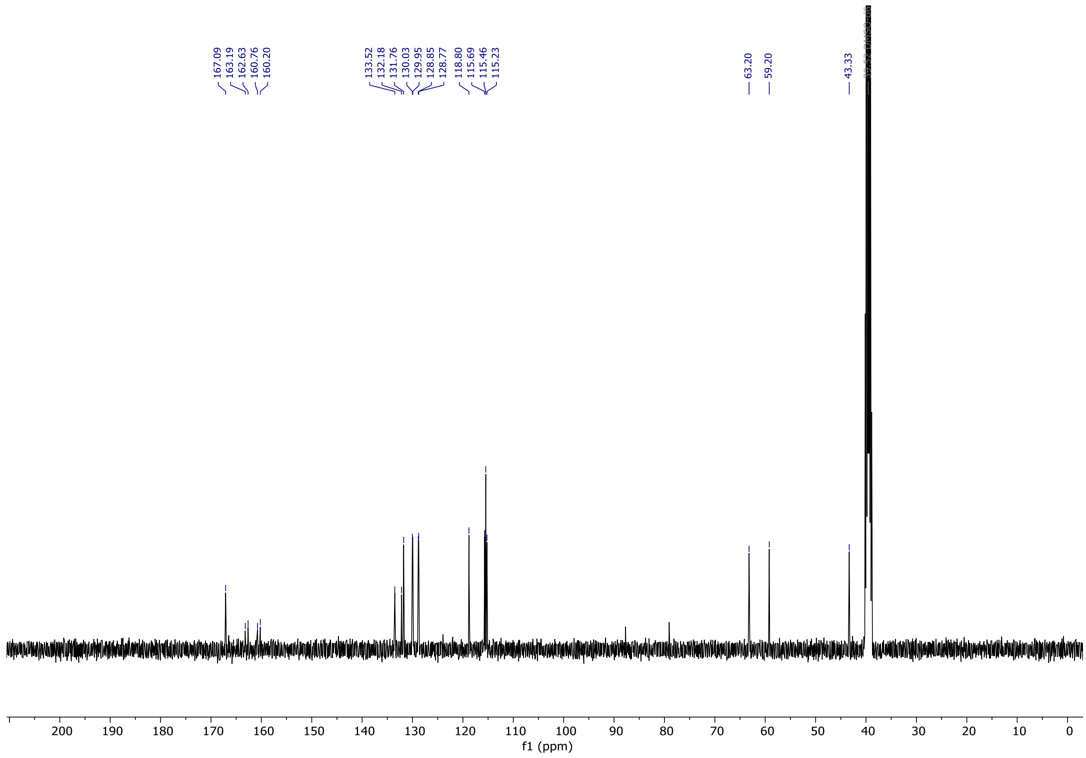

^{19}F-NMR (376 MHz, DMSO-d_6, 298 K):

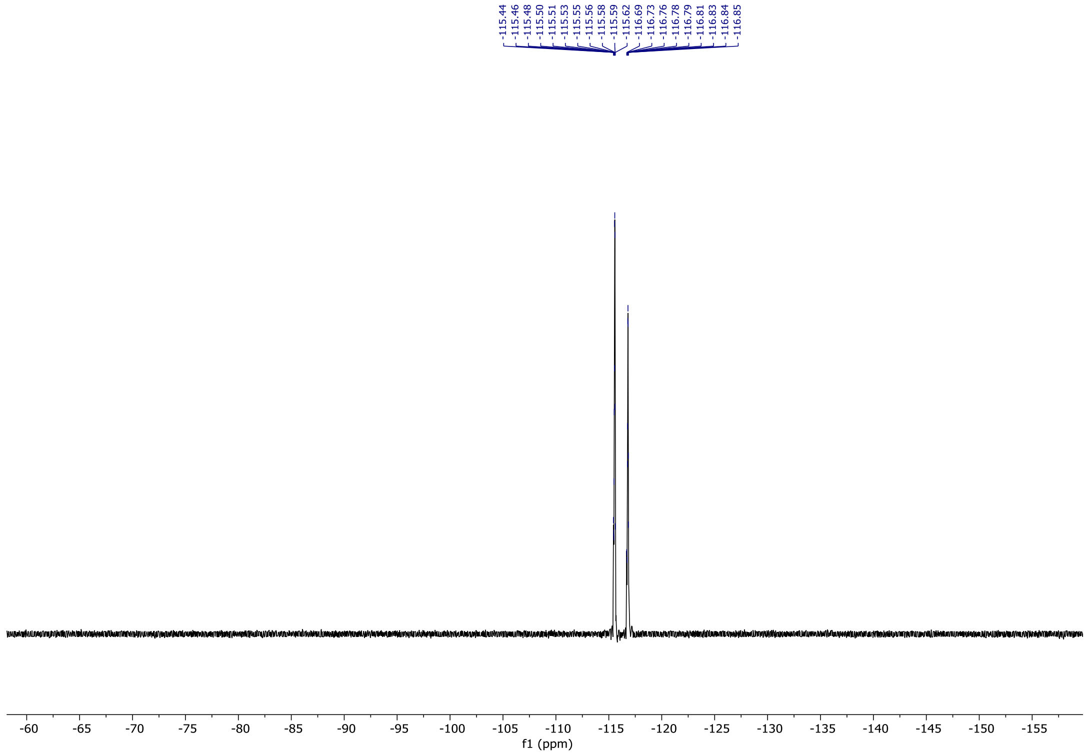

rac-1-(3,5-Dimethylbenzyl)-4-(3,5-dimethylphenyl)-3-vinylazetidin-2-one (8d)

¹H-NMR (500 MHz, DMSO-d_6, 298 K):

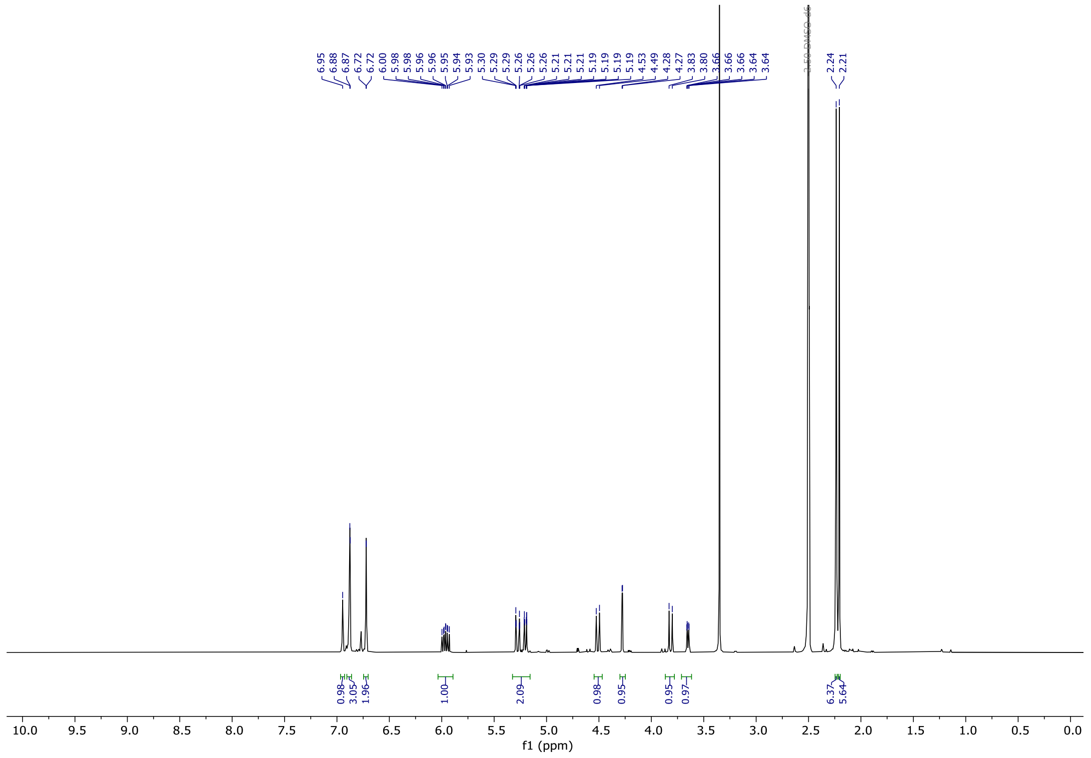

¹³C-NMR (126 MHz, DMSO-d_6, 298 K):

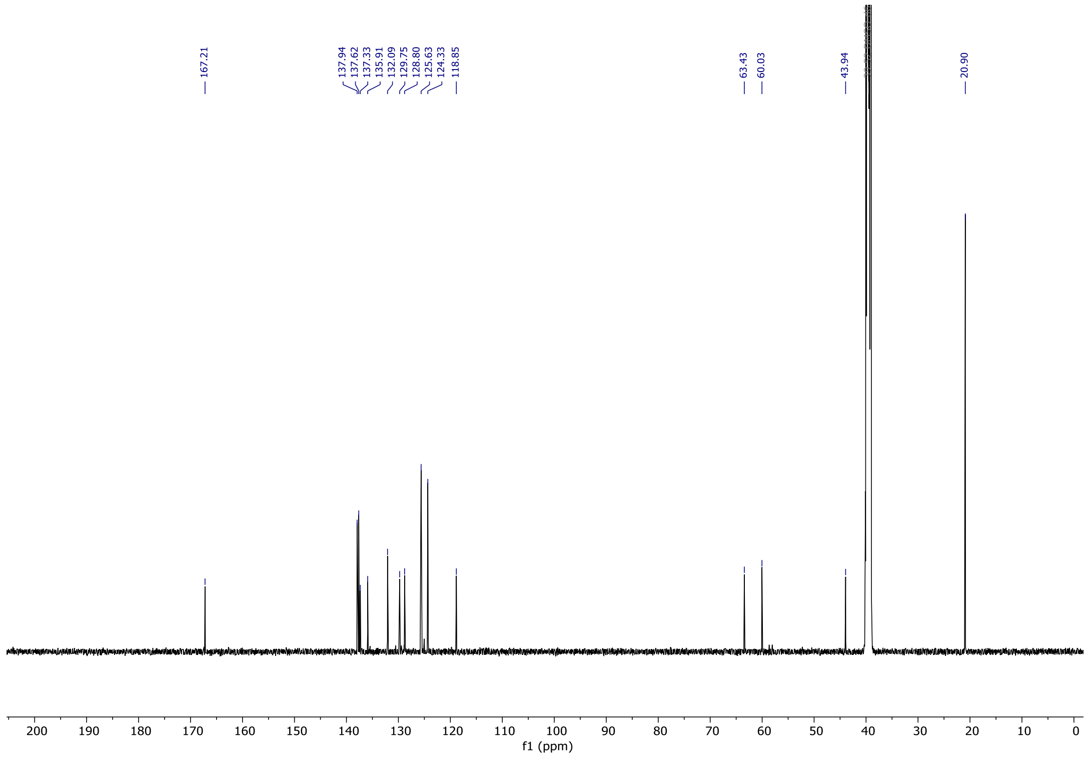

rac-1-(4-Methoxybenzyl)-4-(4-methoxyphenyl)-3-vinylazetidin-2-one (8e)

^{1}H-NMR (500 MHz, DMSO-d_6, 298 K):

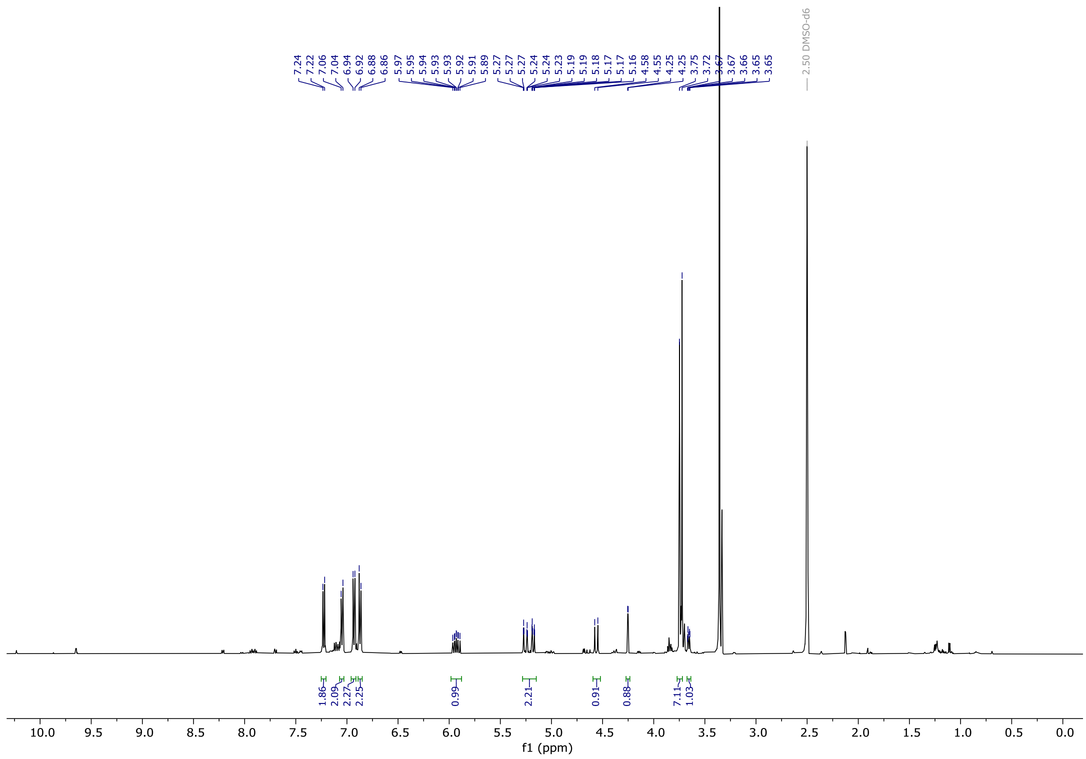

^{13}C-NMR (126 MHz, DMSO-d_6, 298 K):

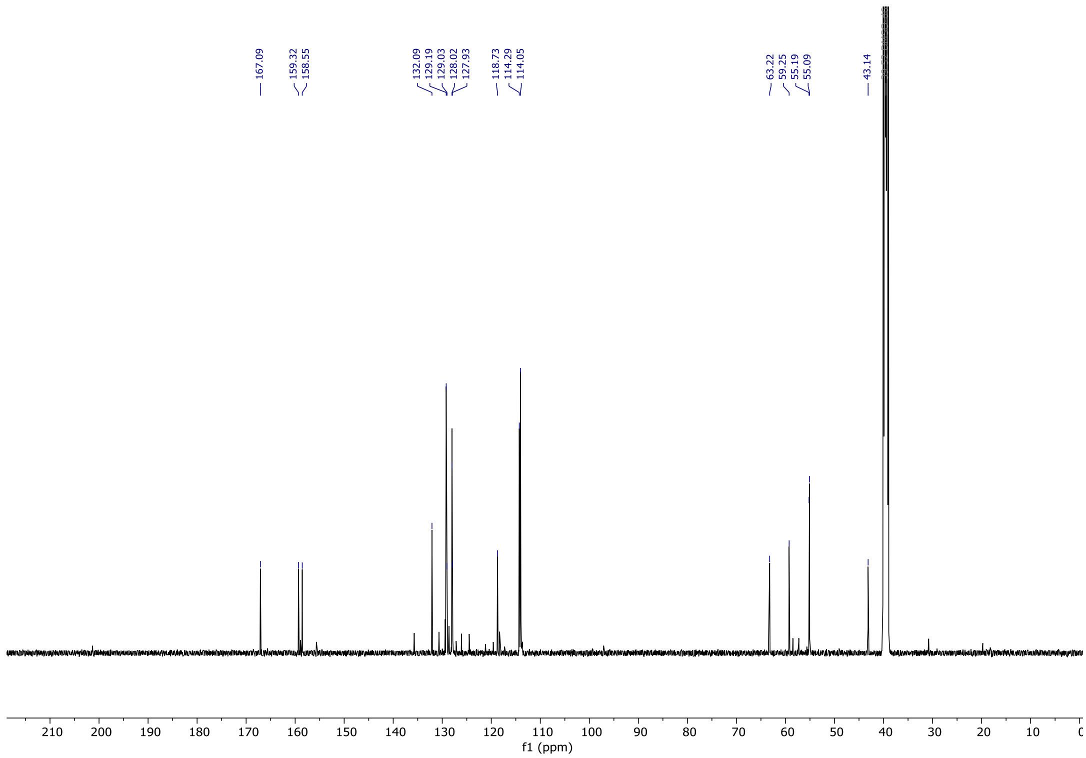

***rac*-1-Methyl-4-phenyl-3-vinylazetidin-2-one-*d*1 (8f-*d*1) and *rac*-1-methyl-4-phenyl-3-(vinyl-1-*d*)azetidin-2-one (8f'-*d*1)**

^{1}H-NMR (500 MHz, DMSO-d_6, 298 K):

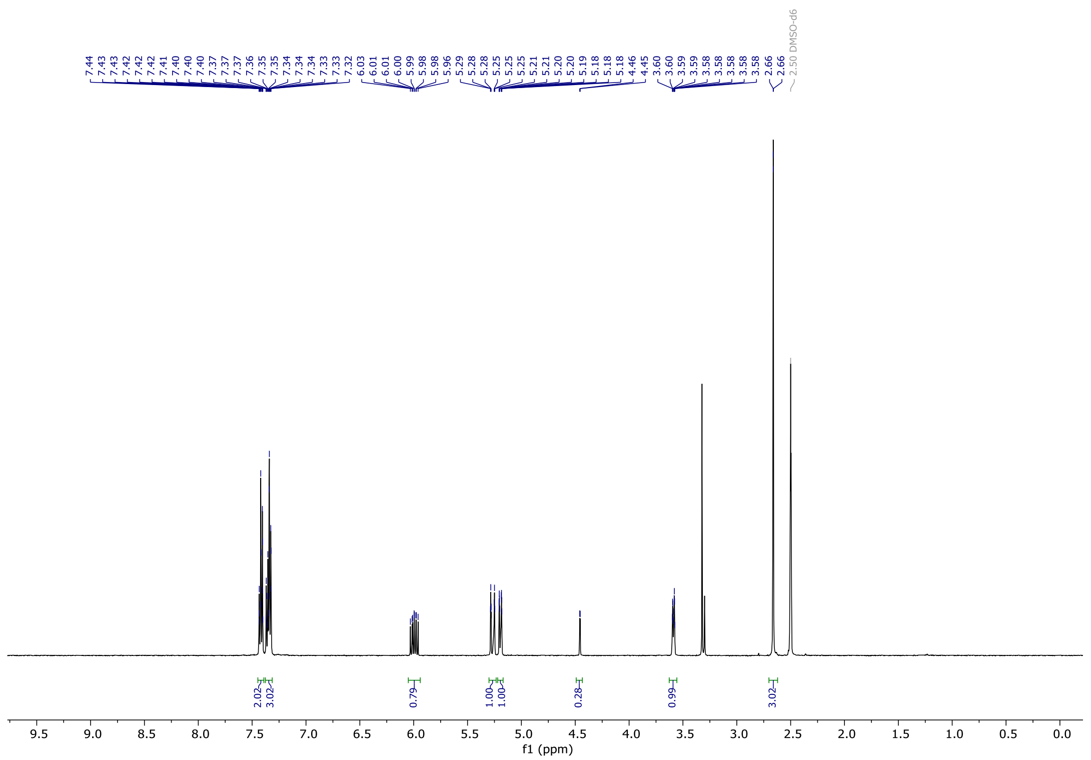

^{13}C-NMR (126 MHz, DMSO-d_6, 298 K):

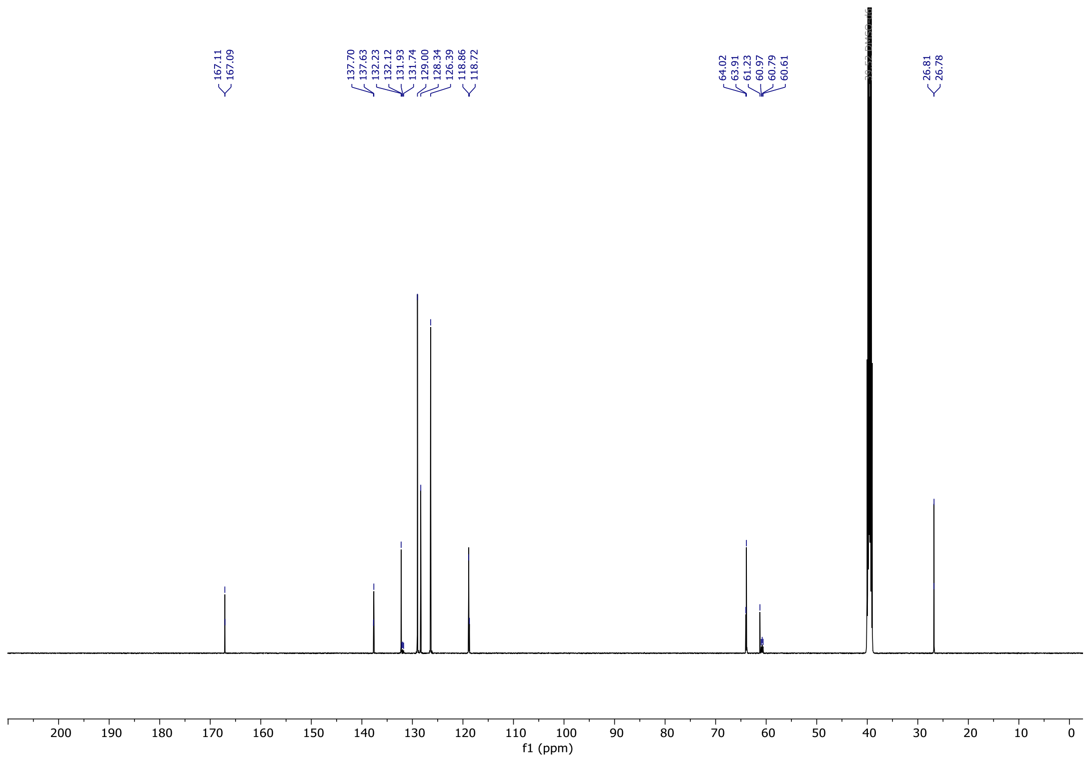

***rac*-1-Methyl-4-phenyl-3-(vinyl-1-*d*)azetidin-2-one-*d* (8f-*d*$_2$)**

^{1}H-NMR (500 MHz, DMSO-*d*$_6$, 298 K):

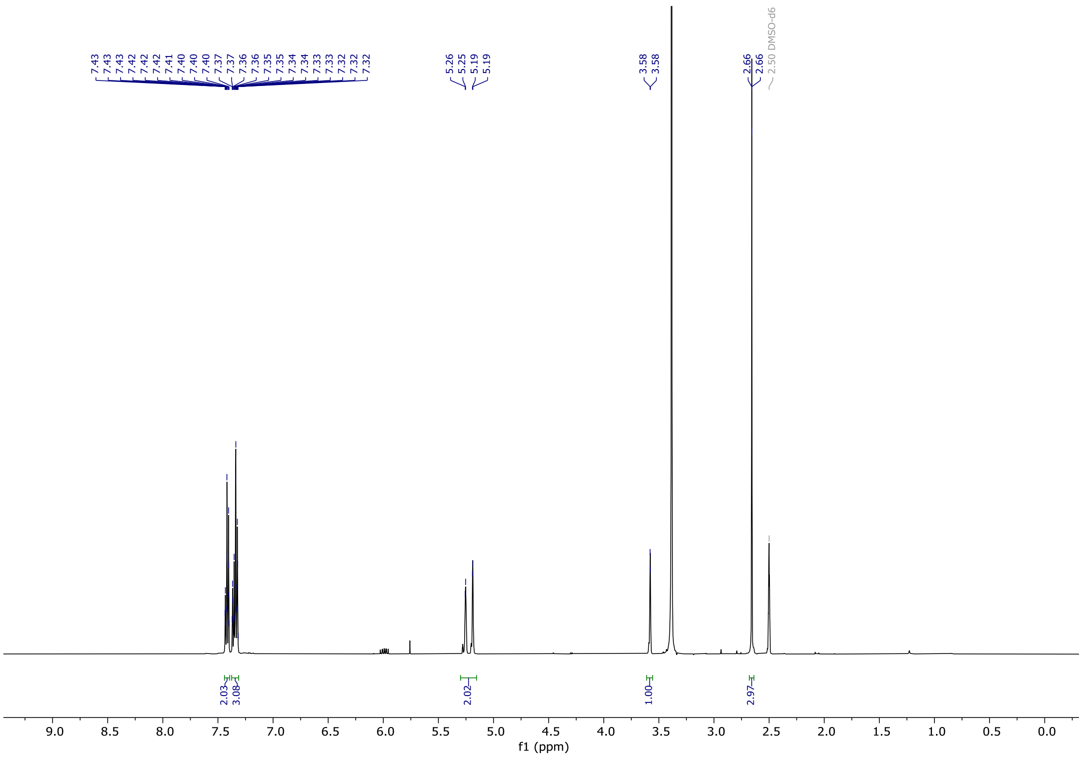

^{13}C-NMR (126 MHz, DMSO-*d*$_6$, 298 K):

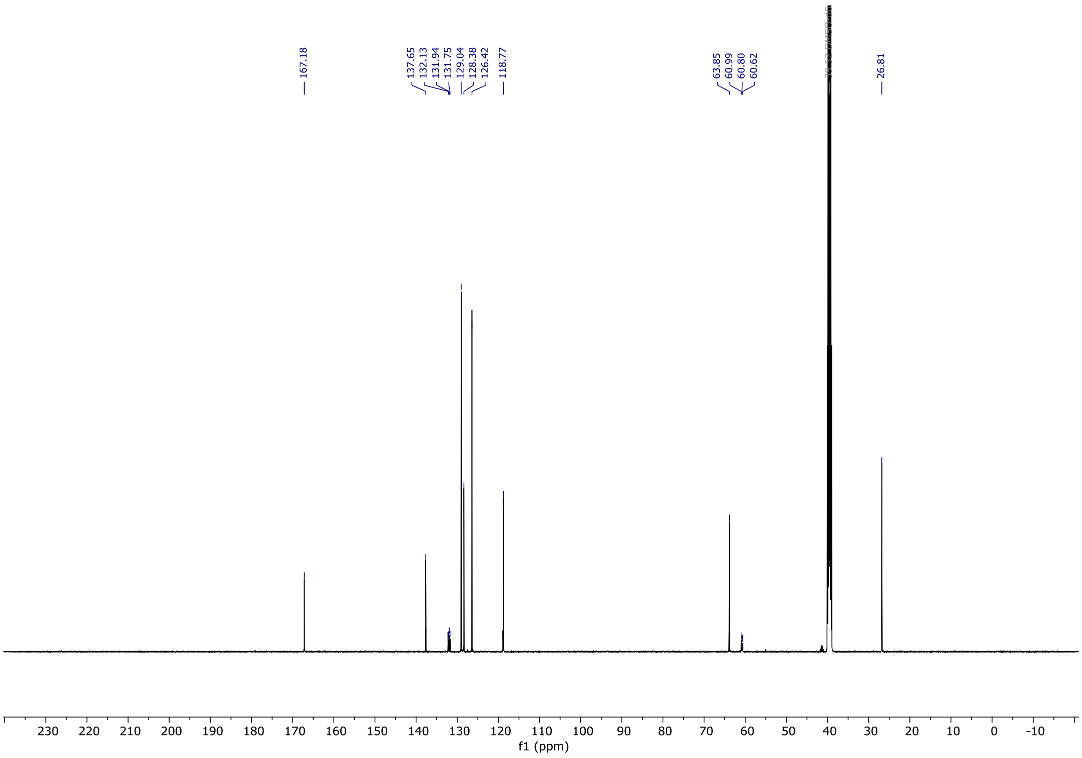

rac-4-(4-Fluorophenyl)-1-methyl-3-vinylazetidin-2-one (8g)

^{1}H-NMR (400 MHz, $CDCl_3$, 298 K):

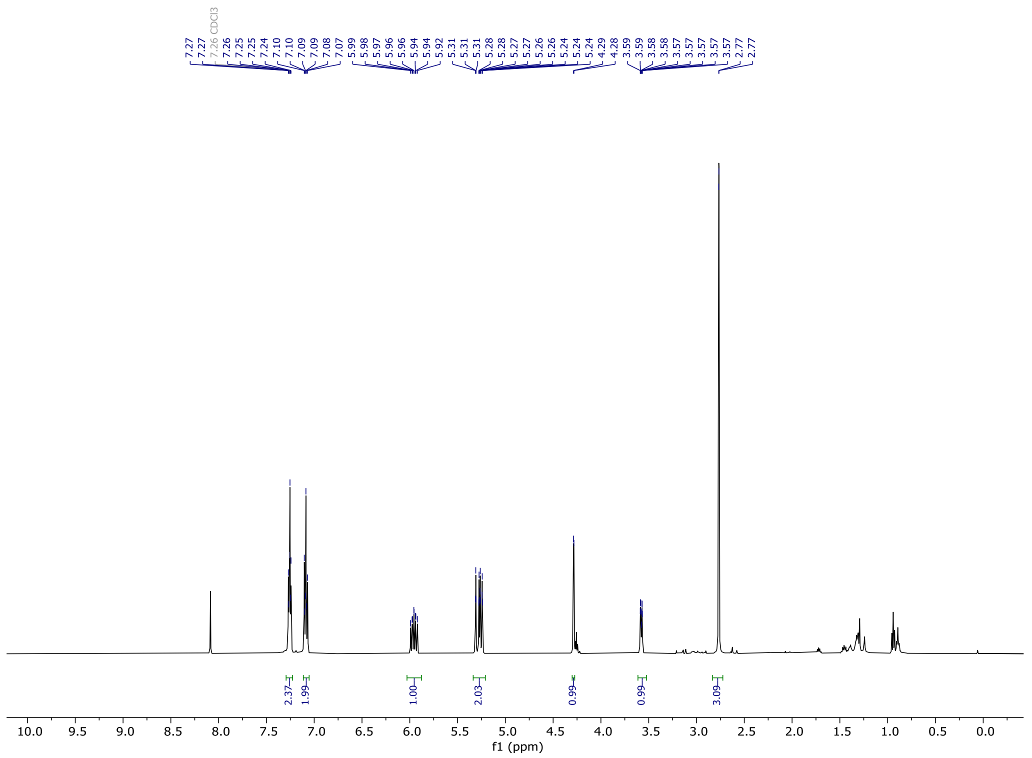

^{13}C-NMR (101 MHz, $CDCl_3$, 298 K):

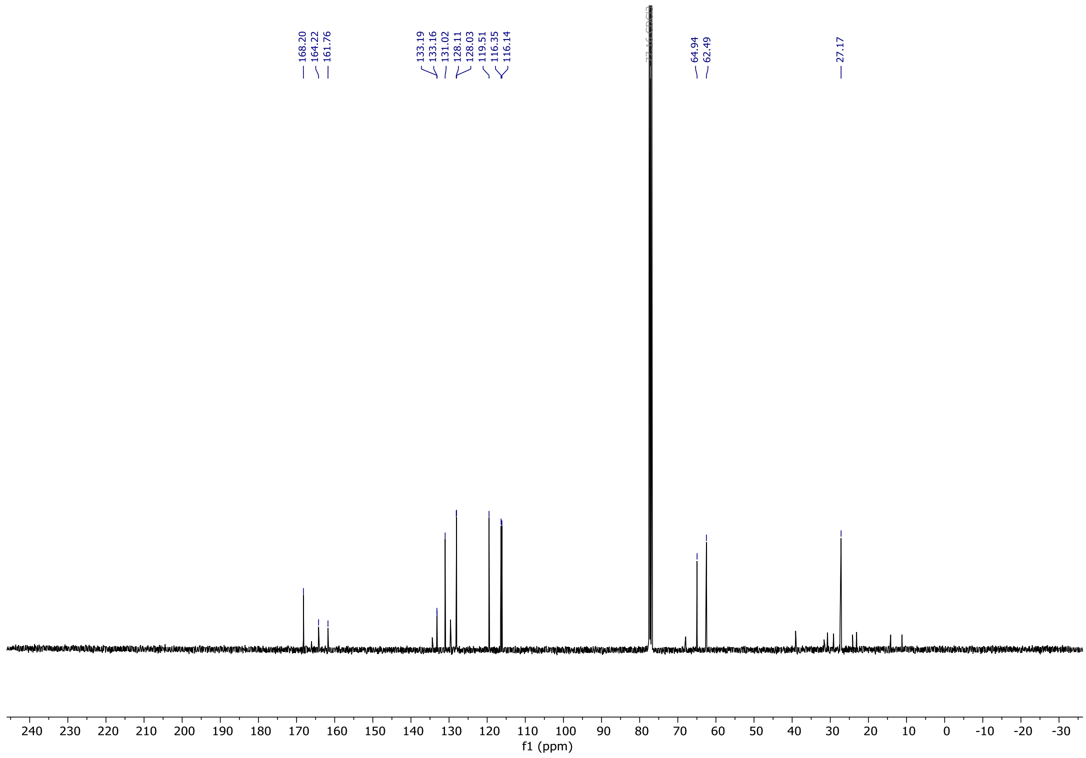

^{19}F-NMR (376 MHz, $CDCl_3$, 298 K):

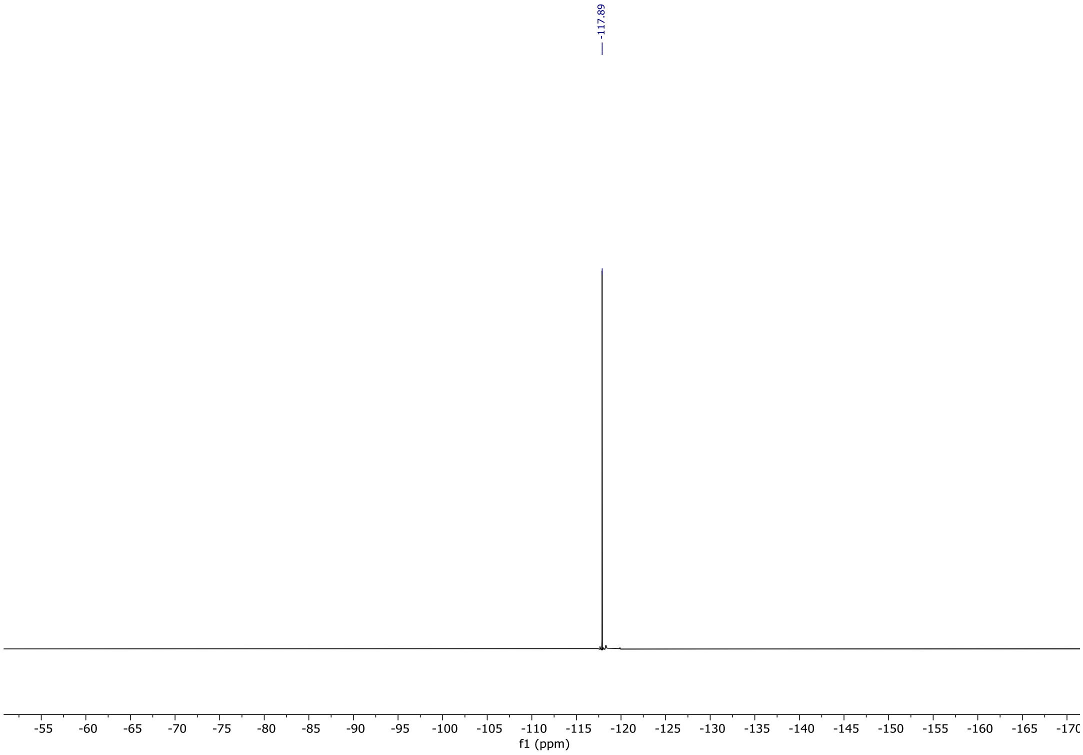

***rac*-1-Methyl-4-(naphthalen-2’-yl)-3-vinylazetidin-2-one (8h)**

^{1}H-NMR (500 MHz, DMSO-d_6, 298 K):

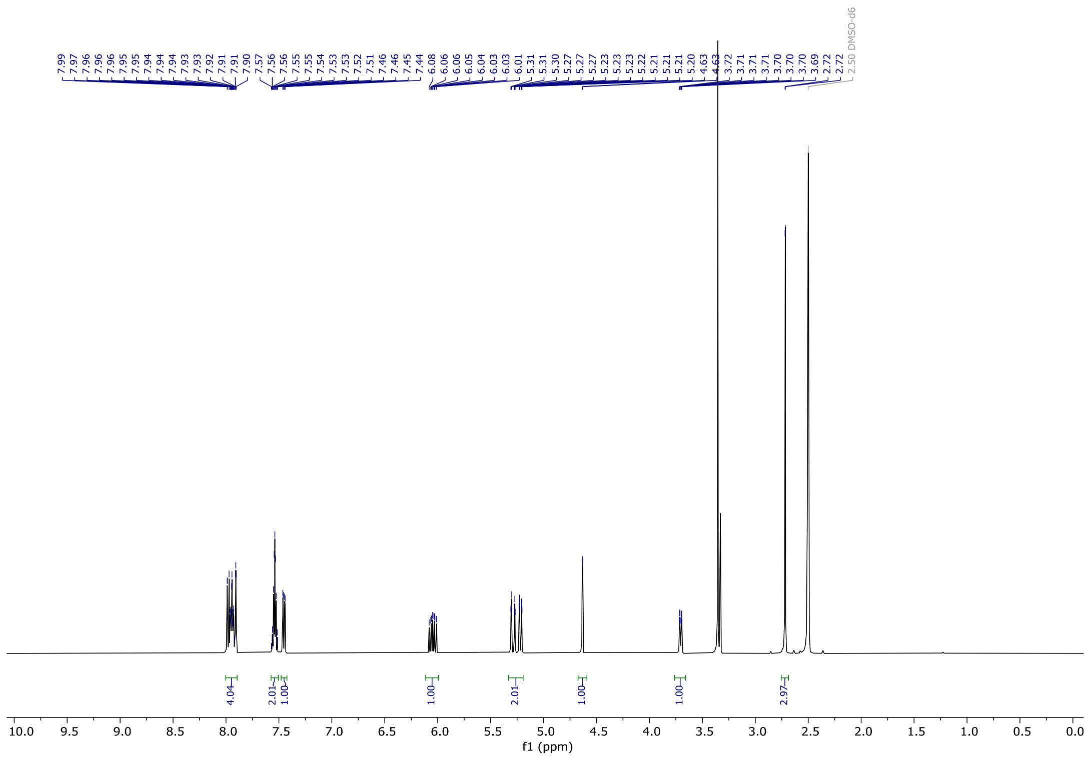

^{13}C-NMR (126 MHz, DMSO-d_6, 298 K):

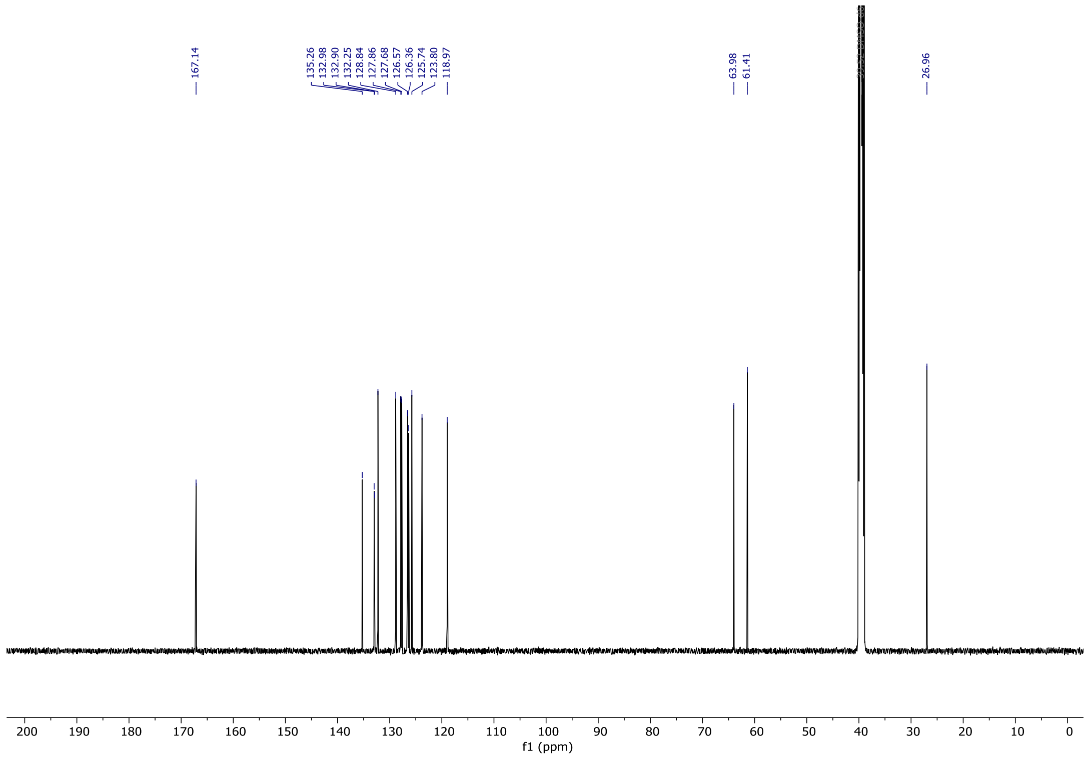

rac-4-(4-Bromophenyl)-1-methyl-3-vinylazetidin-2-one (8i)

^{1}H-NMR (500 MHz, DMSO-d_6, 298 K):

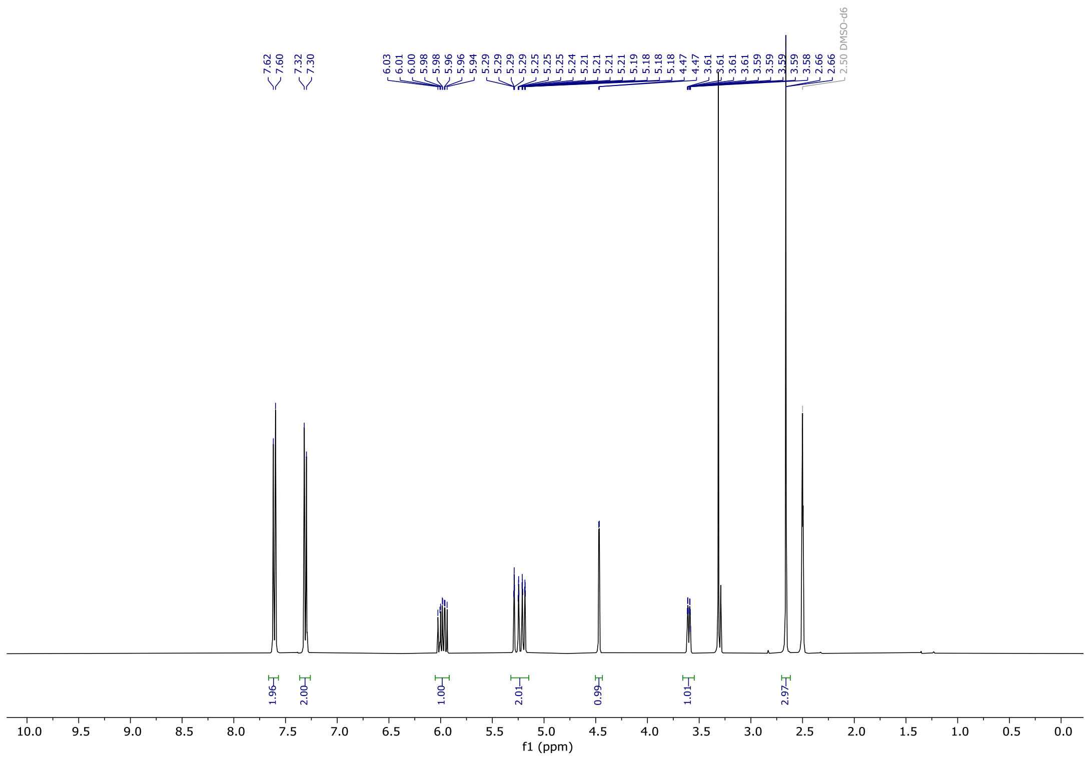

^{13}C-NMR (126 MHz, DMSO-d_6, 298 K):

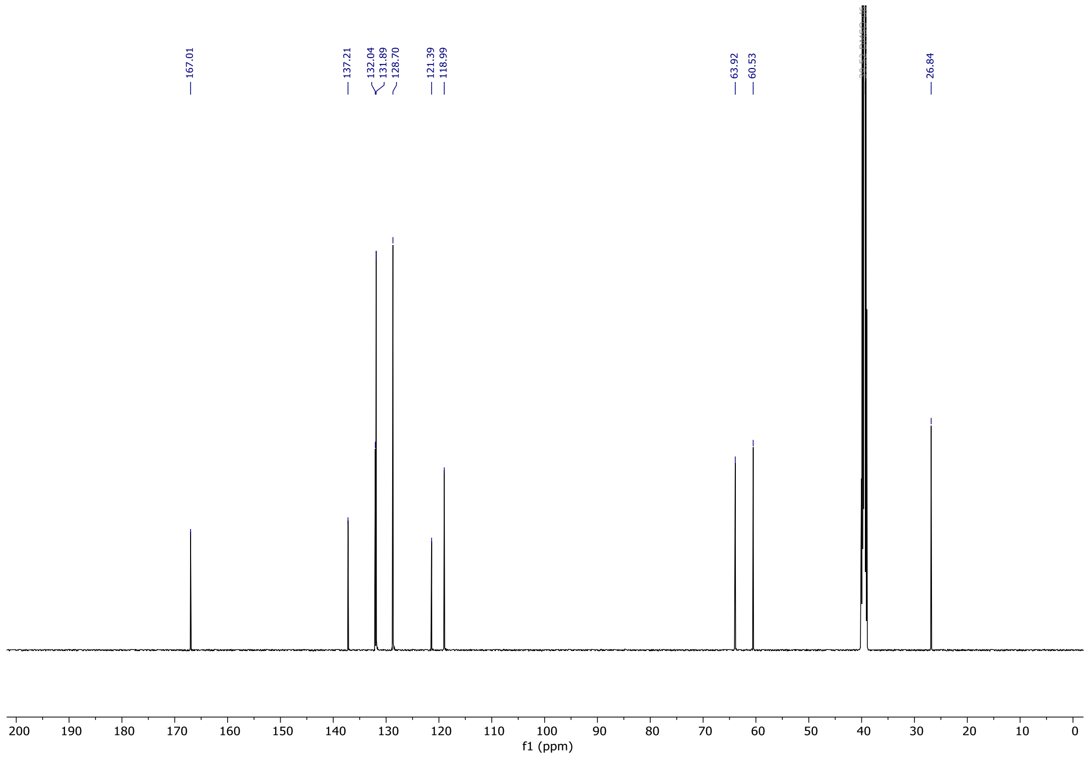

***rac*-1-Isopropyl-4-phenyl-3-vinylazetidin-2-one (8k)**

^{1}H-NMR (400 MHz, DMSO-d_6, 298 K):

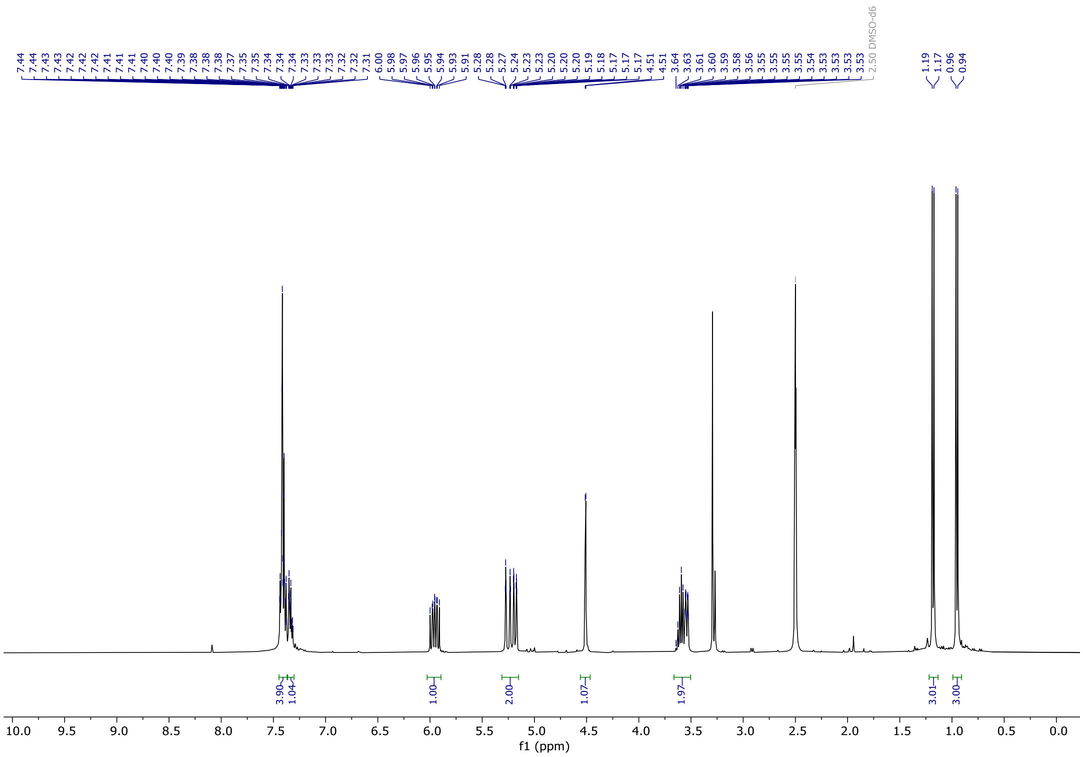

^{13}C-NMR (101 MHz, DMSO-d_6, 298 K):

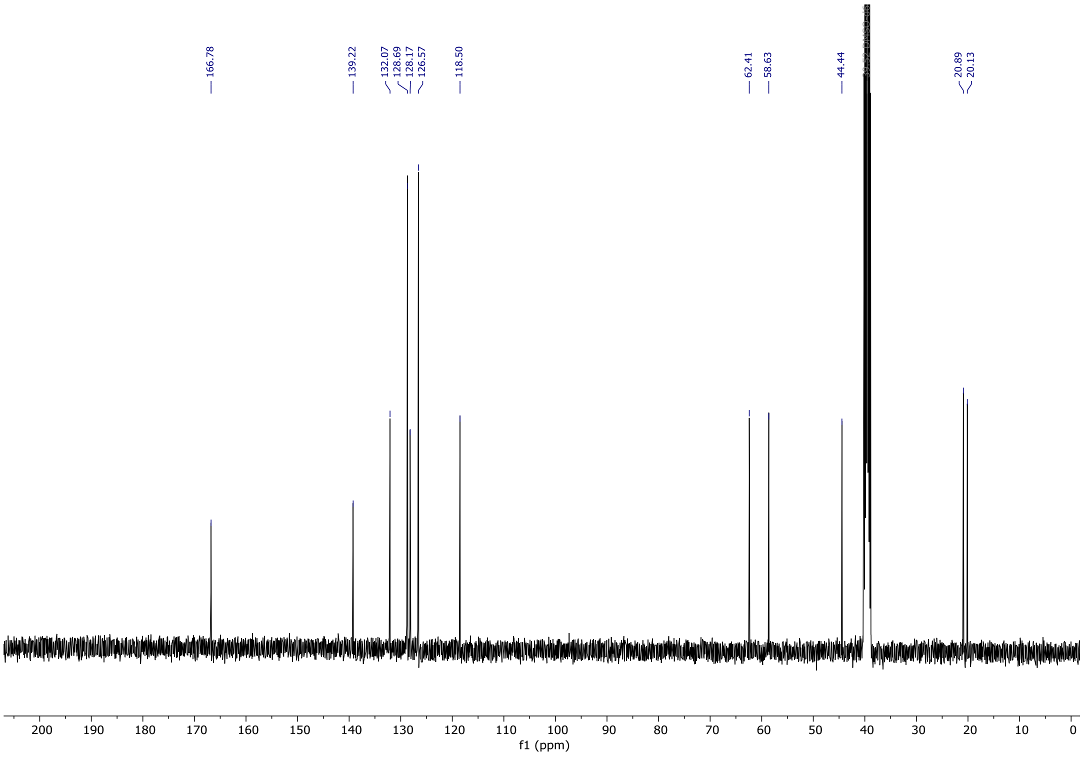

***rac*-4-phenyl-1-[(trimethylsilyl)methyl]-3-vinylazetidin-2-one (8l)**

^{1}H-NMR (400 MHz, DMSO-d_6, 298 K):

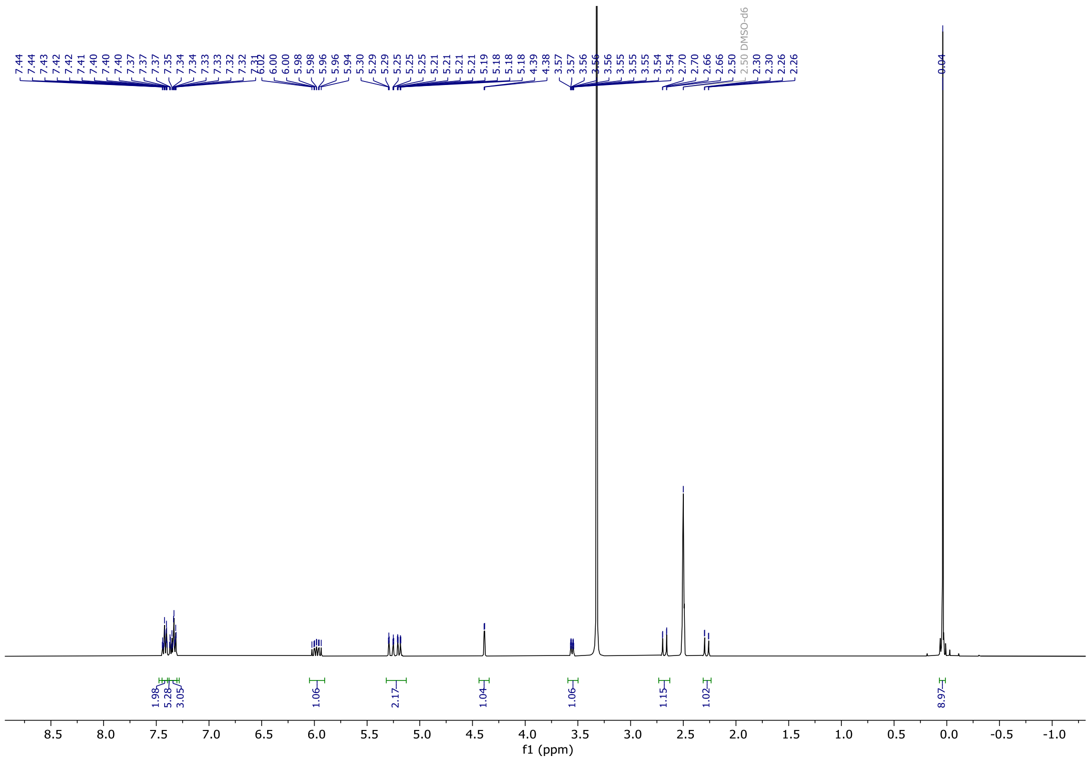

^{13}C-NMR (101 MHz, DMSO-d_6, 298 K):

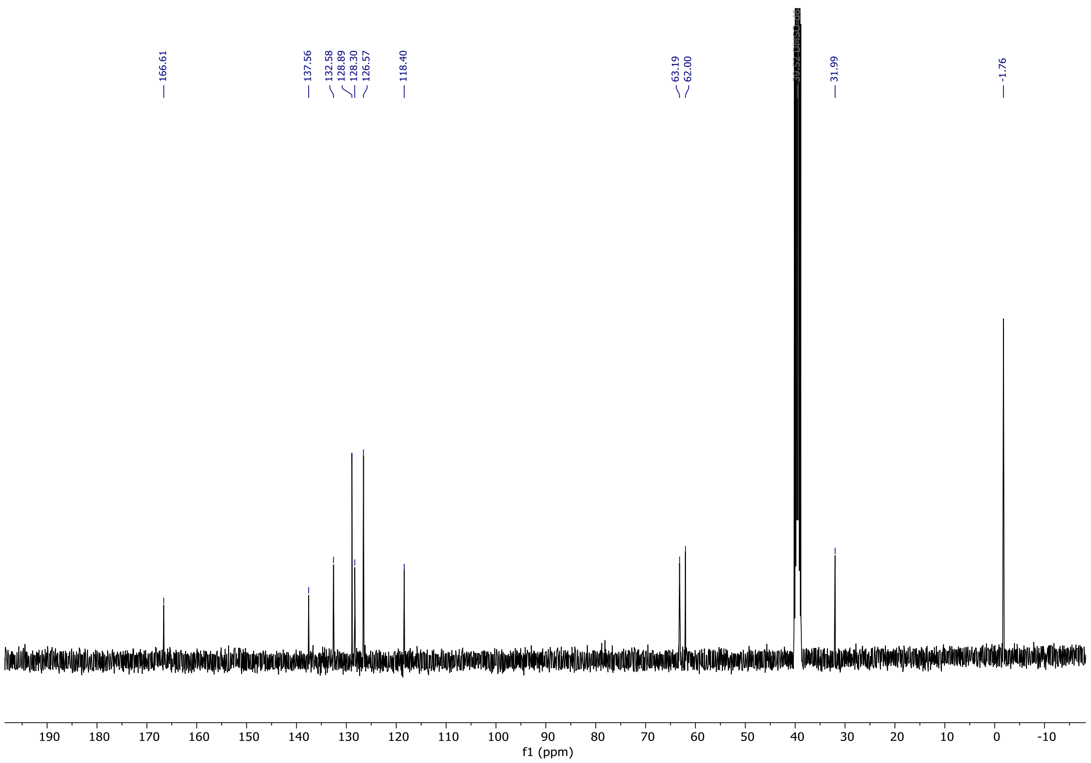

rac-1-[2-(benzyloxy)ethyl]-4-phenyl-3-vinylazetidin-2-one (8m)

^{1}H-NMR (500 MHz, DMSO-d_6, 298 K):

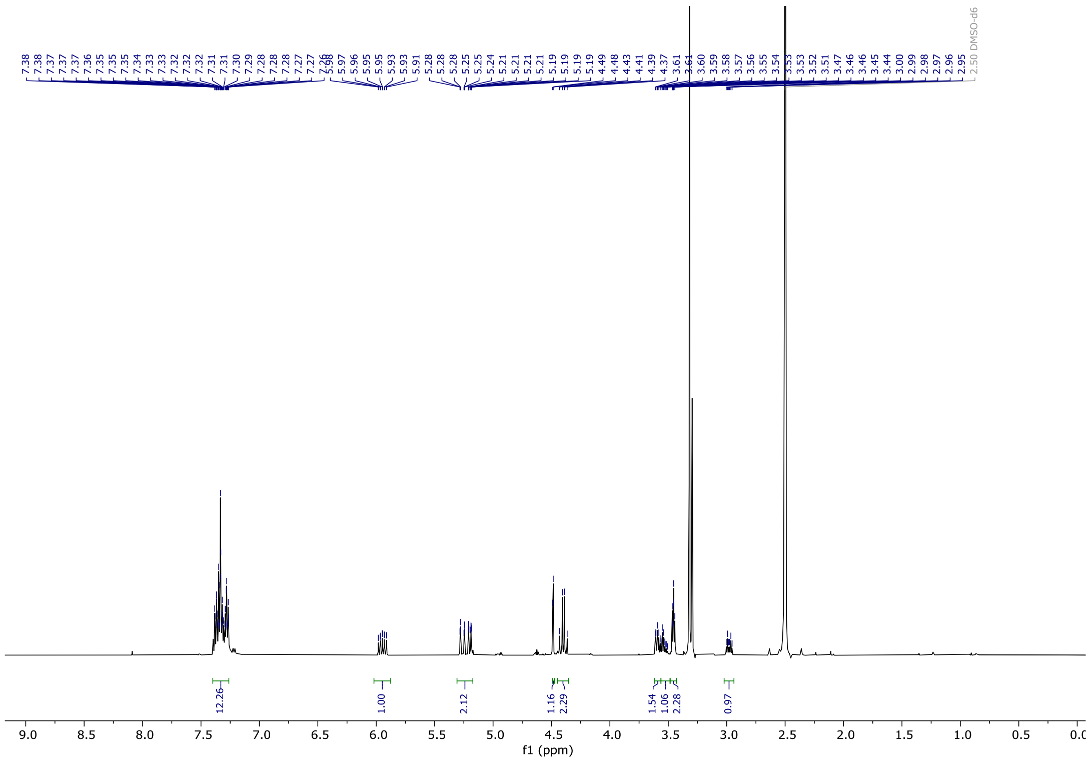

^{13}C-NMR (126 MHz, DMSO-d_6, 298 K):

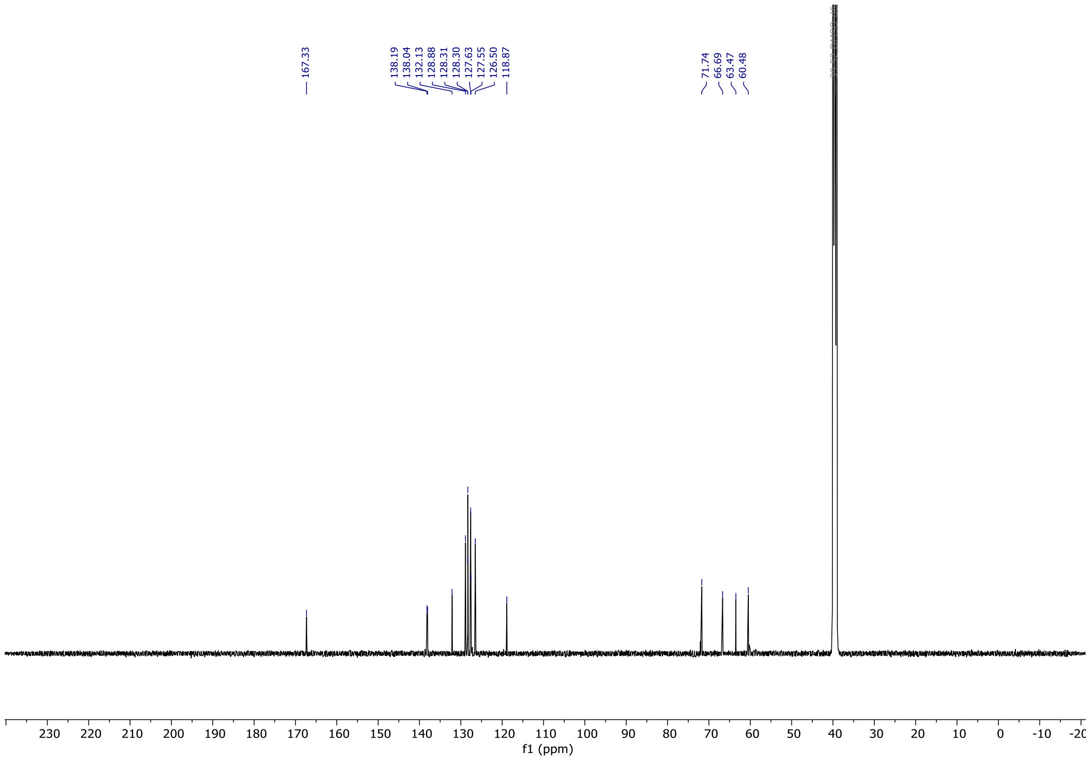

***rac*-1-Benzyl-4,4-dimethyl-3-vinylazetidin-2-one (9a)**

^{1}H-NMR (400 MHz, DMSO-d_6, 298 K):

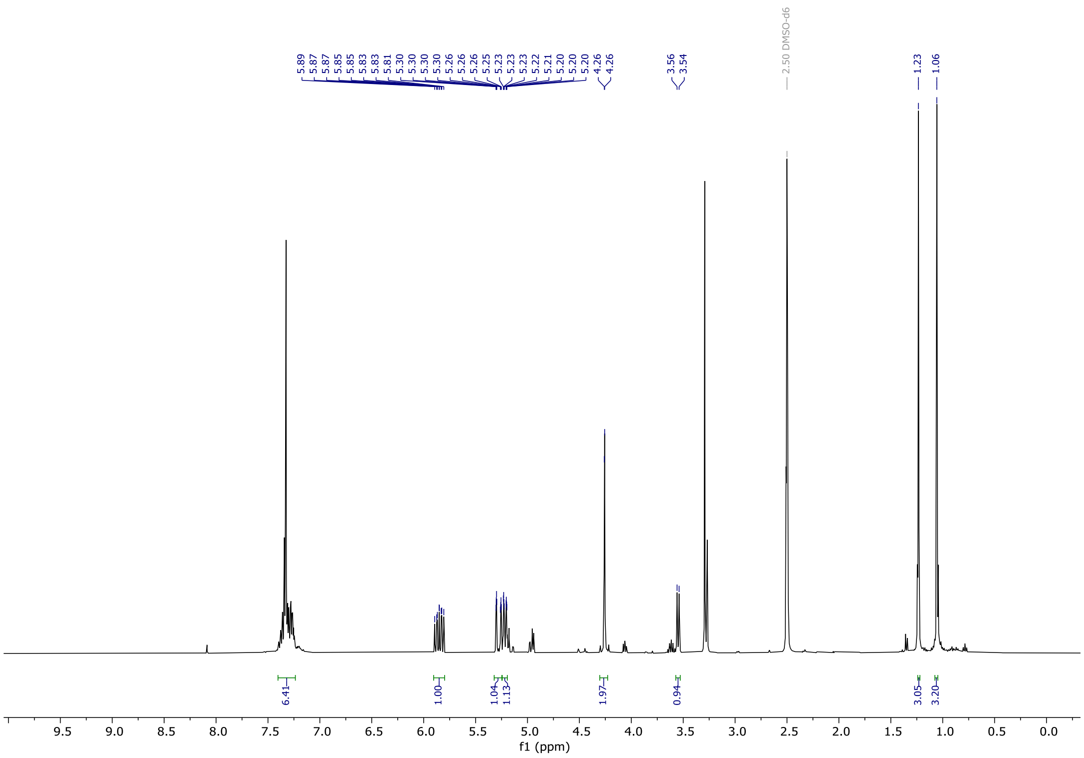

^{13}C-NMR (101 MHz, DMSO-d_6, 298 K):

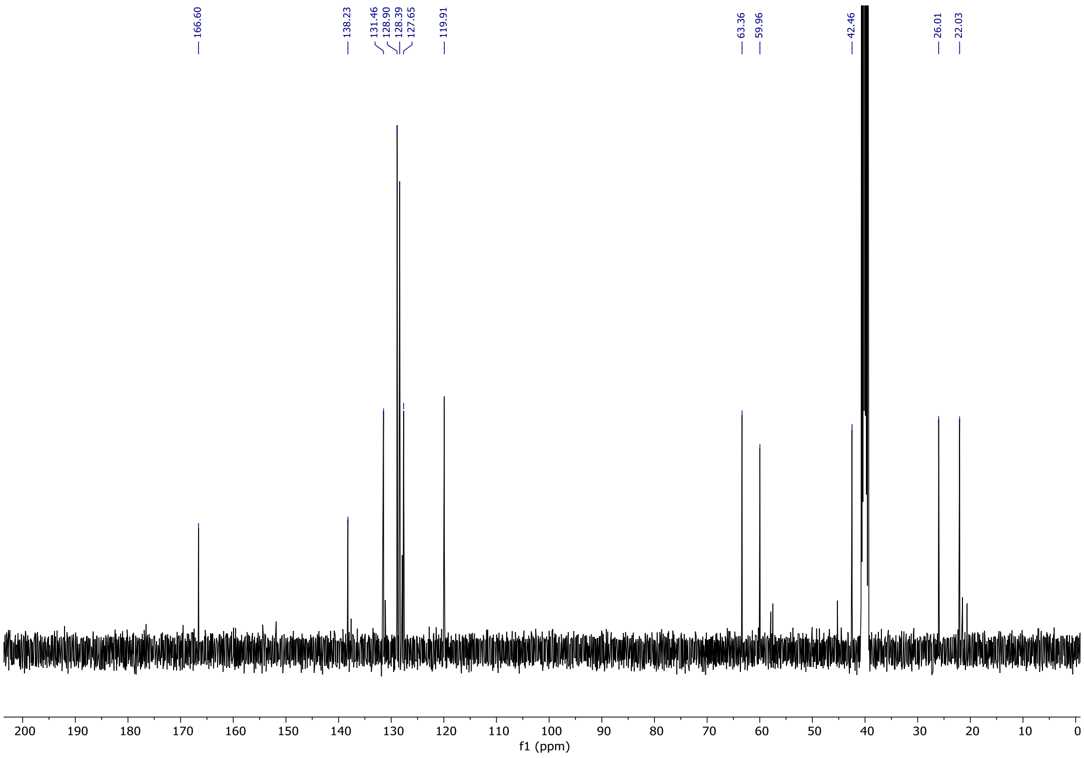

***rac*-1-Methyl-4,4-diphenyl-3-vinylazetidin-2-one (9b)**

^{1}H-NMR (400 MHz, DMSO-d_6, 298 K):

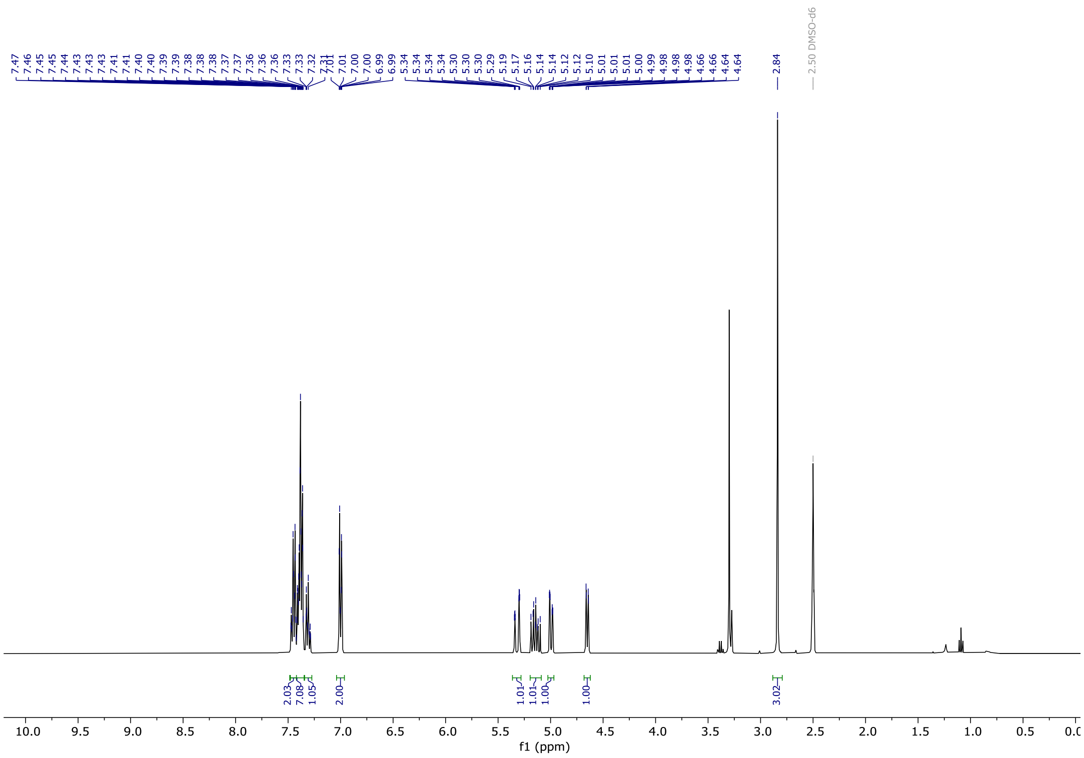

^{13}C-NMR (101 MHz, DMSO-d_6, 298 K):

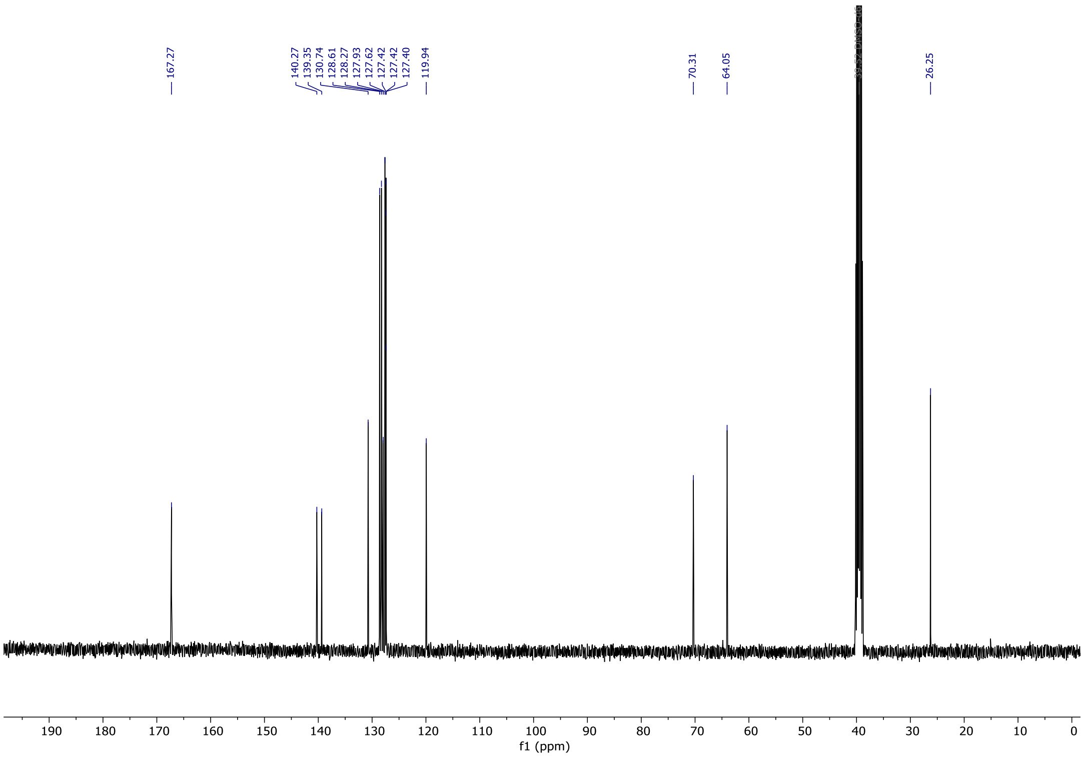

***rac*-1-Isopropyl-4,4-dimethyl-3-vinylazetidin-2-one (9c)**

^{1}H-NMR (500 MHz, DMSO-d_6, 298 K):

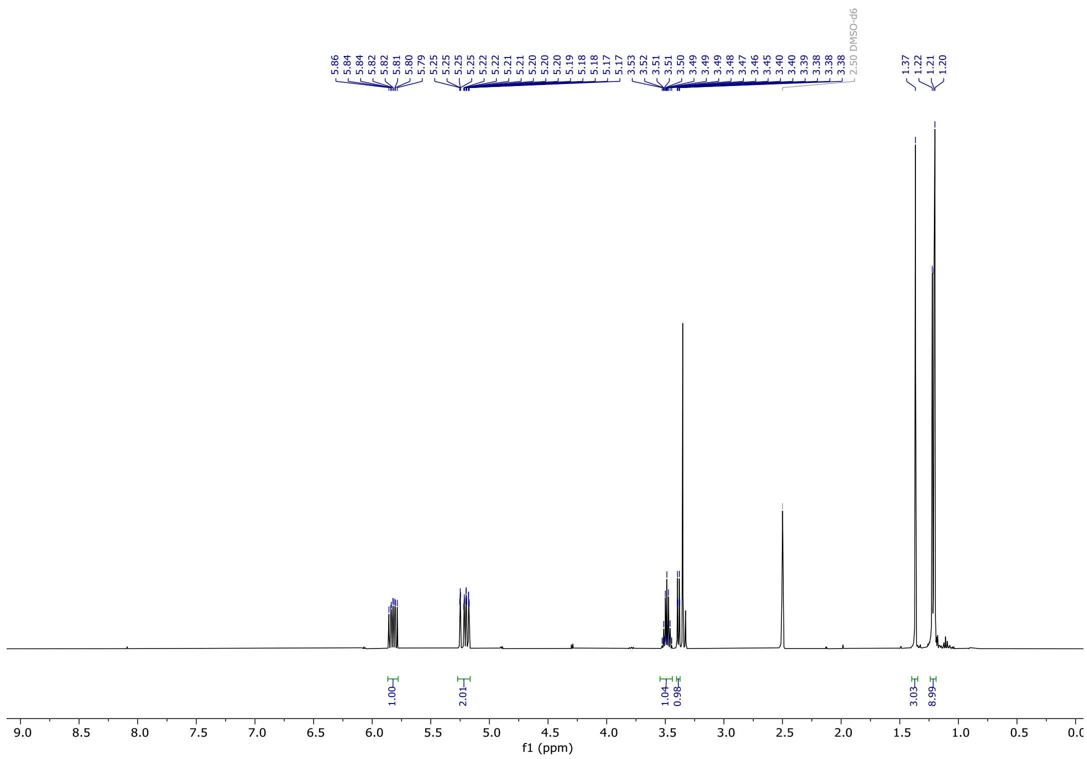

^{13}C-NMR (126 MHz, DMSO-d_6, 298 K):

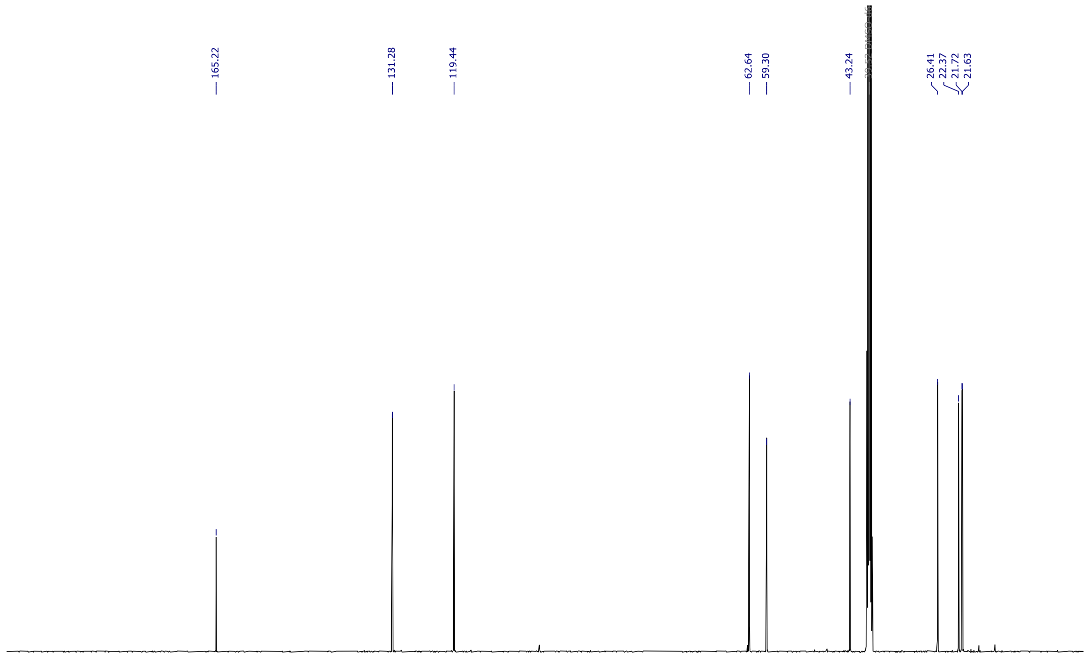

***rac*-1-Cyclohexyl-3-vinyl-1-azaspiro[3.5]nonan-2-one (9d)**

¹H-NMR (500 MHz, $CDCl_3$, 298 K):

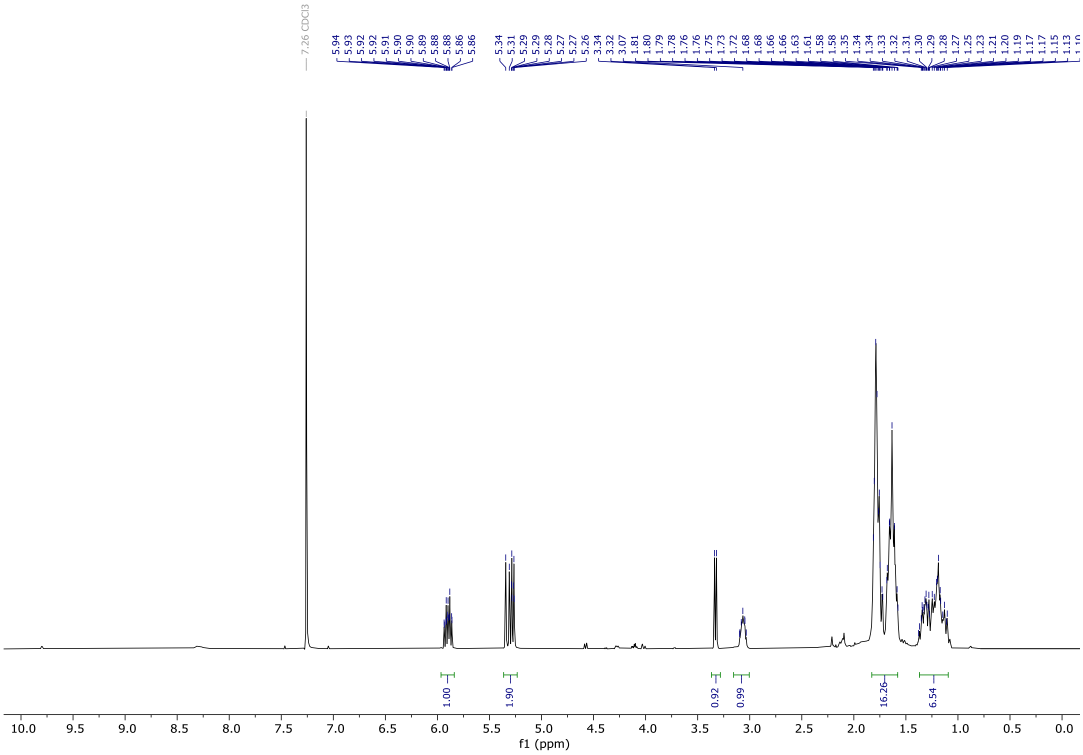

¹³C-NMR (126 MHz, $CDCl_3$, 298 K):

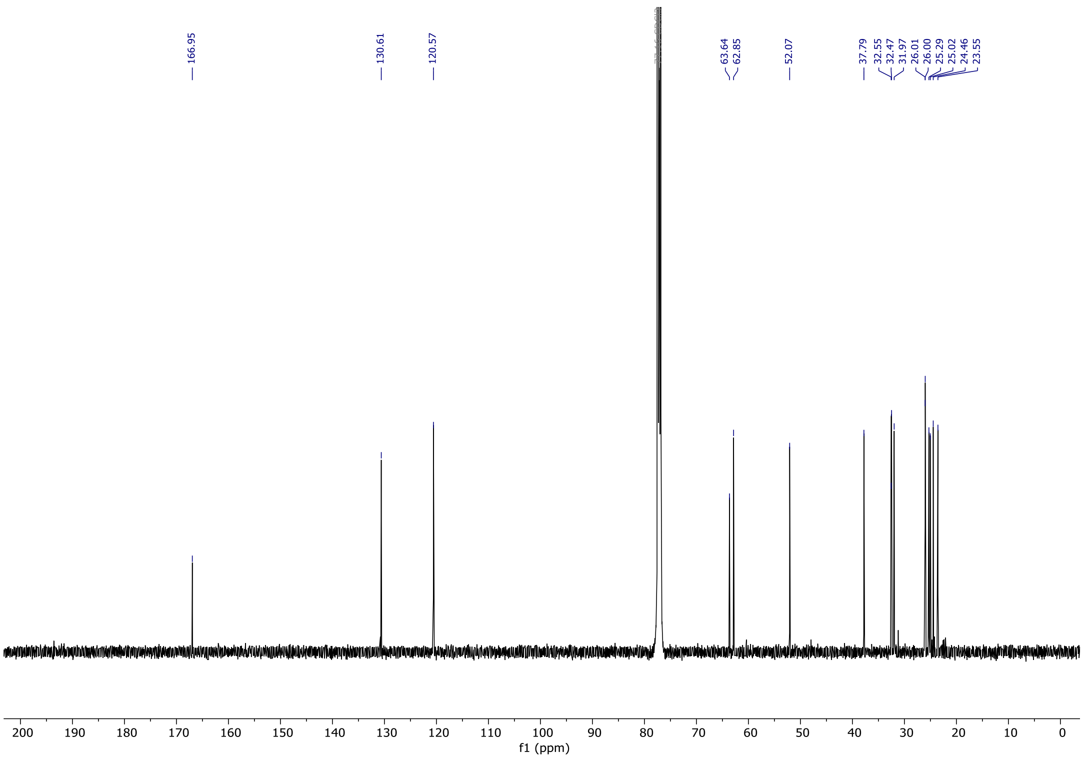

rac-1-Benzyl-3-[(E)-3,3-dimethylbut-1-en-1-yl]-4-phenylazetidin-2-one (12)

E-**14:**

^{1}H-NMR (500 MHz, DMSO-d_6, 298 K):

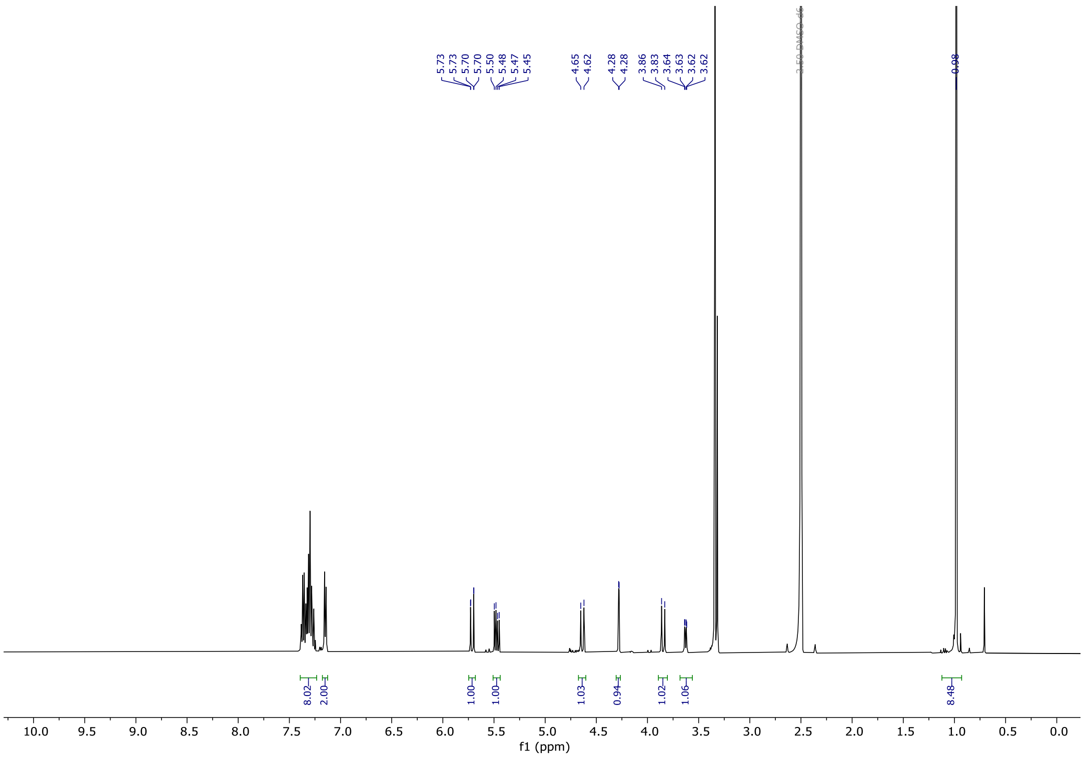

^{13}C-NMR (126 MHz, DMSO-d_6, 298 K):

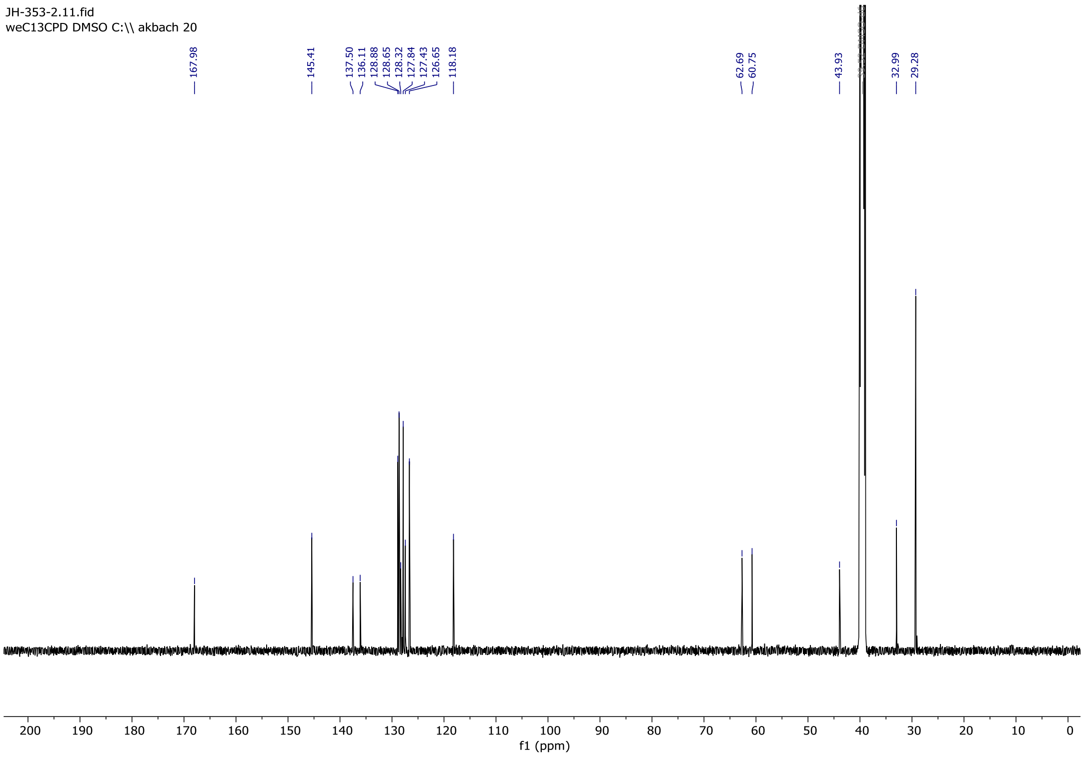

***rac*-1-Benzyl-4-phenyl-3-[2'-(4,4,5,5-tetramethyl-1,3,2-dioxaborolan-2-yl)ethyl]azetidin-2-one (18)**

^{1}H-NMR (500 MHz, DMSO-d_6, 298 K):

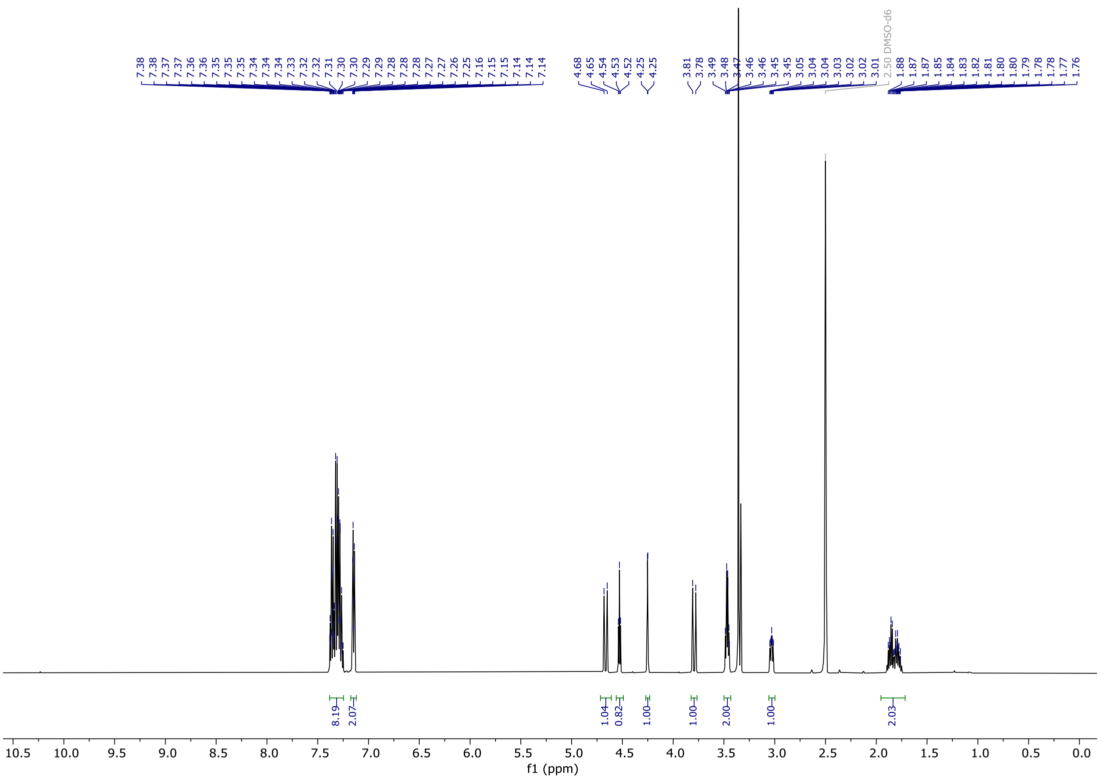

^{13}C-NMR (126 MHz, DMSO-d_6, 298 K):

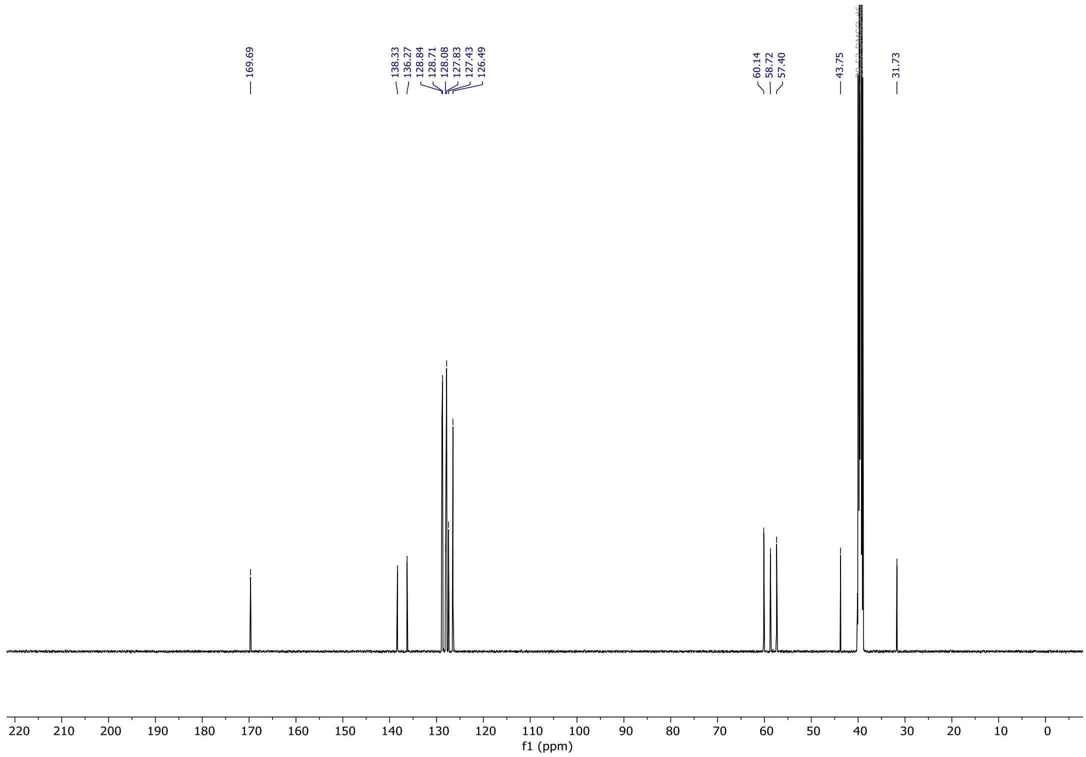

***rac*-1-Benzyl-3-(2'-hydroxyethyl)-4-phenylazetidin-2-one (19)**

^{1}**H-NMR** (500 MHz, DMSO-d_6, 298 K):

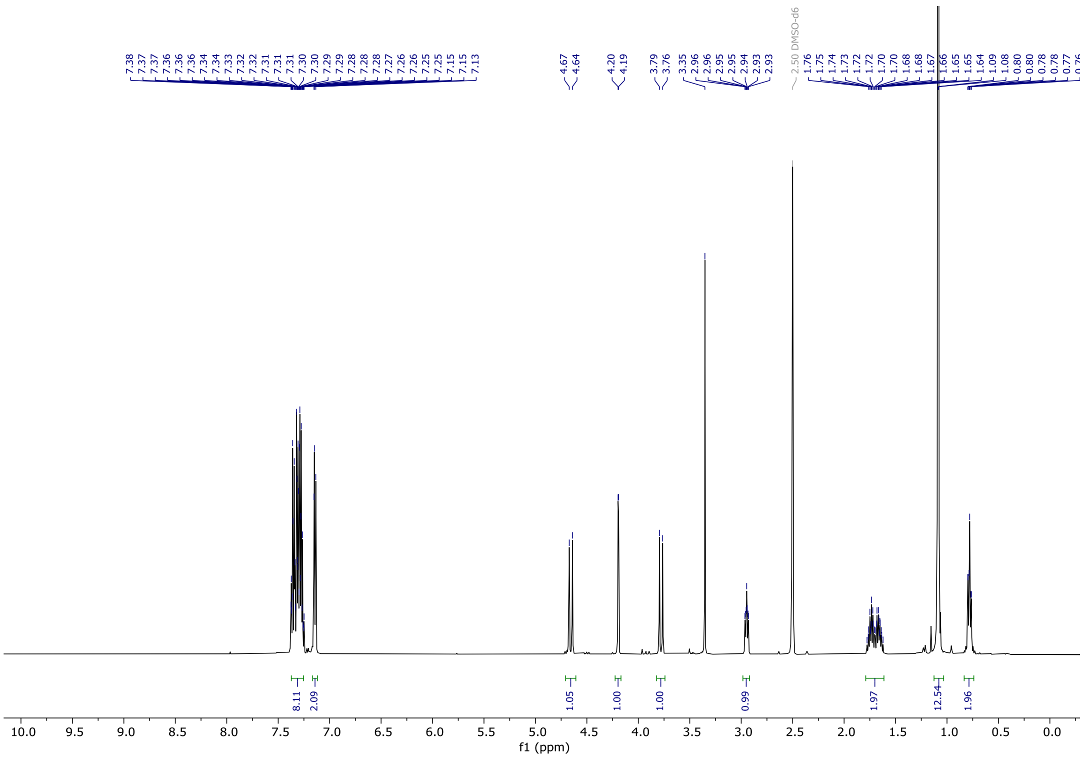

^{13}**C-NMR** (126 MHz, DMSO-d_6, 298 K):

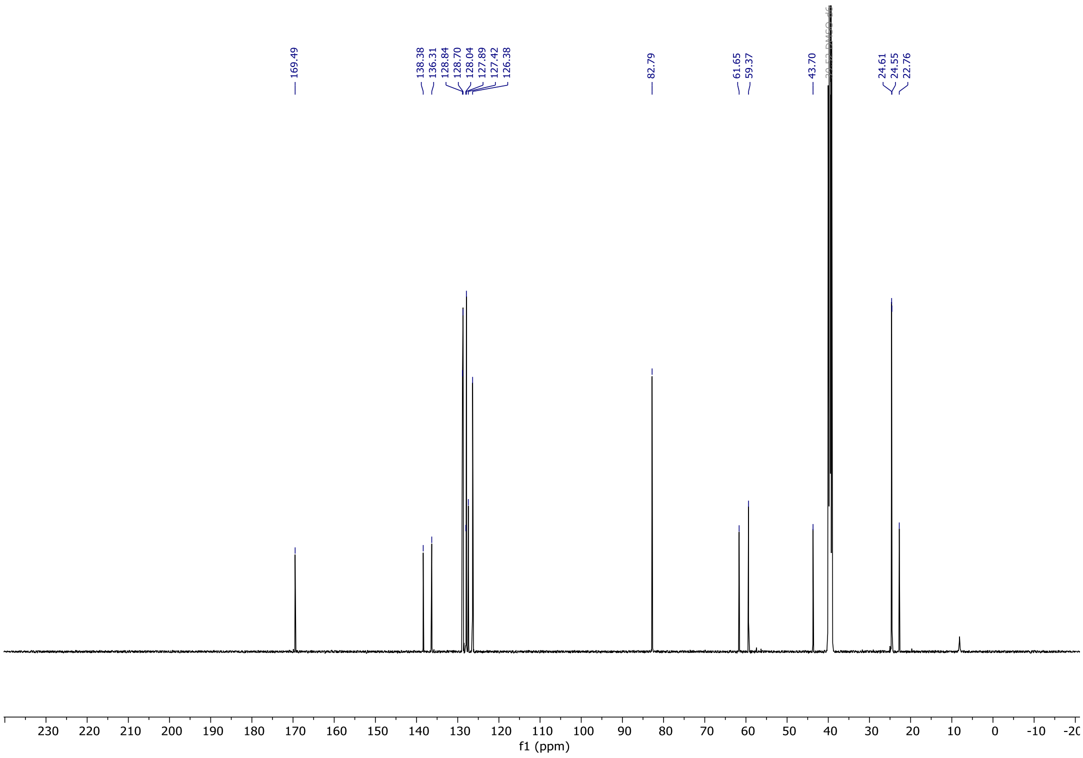

***rac*-2’-(-1-benzyl-2-oxo-4-phenylazetidin-3-yl)acetaldehyde (20)**

^{1}H-NMR (500 MHz, $CDCl_3$, 298 K):

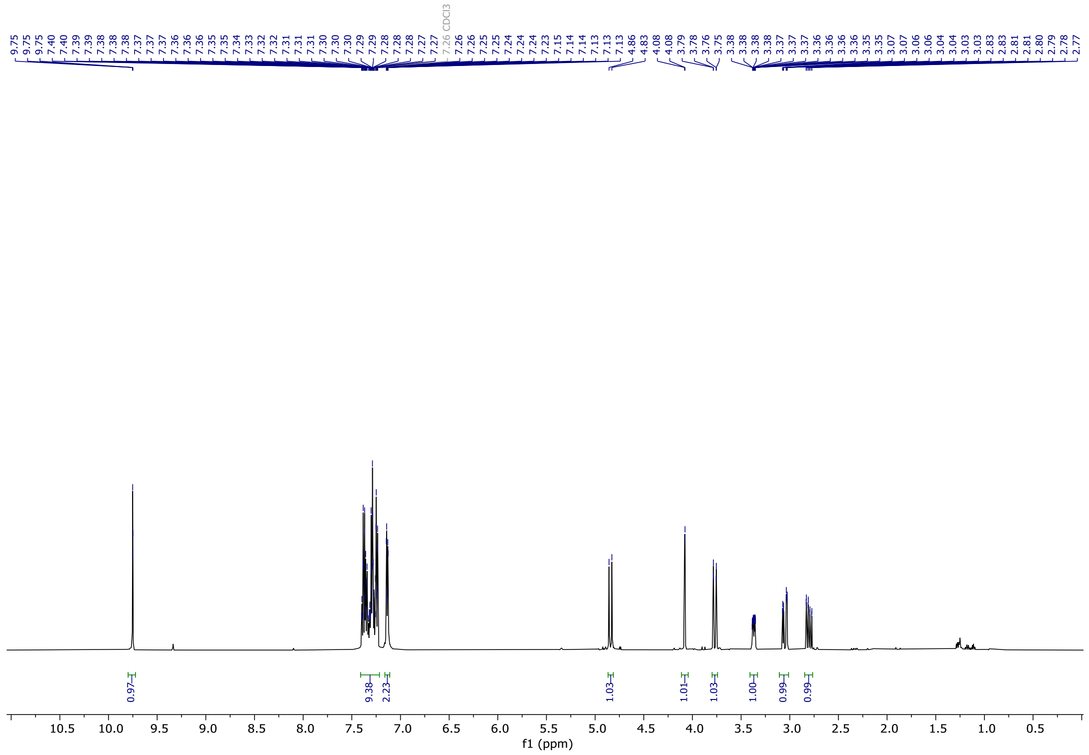

^{13}C-NMR (126 MHz, $CDCl_3$, 298 K):

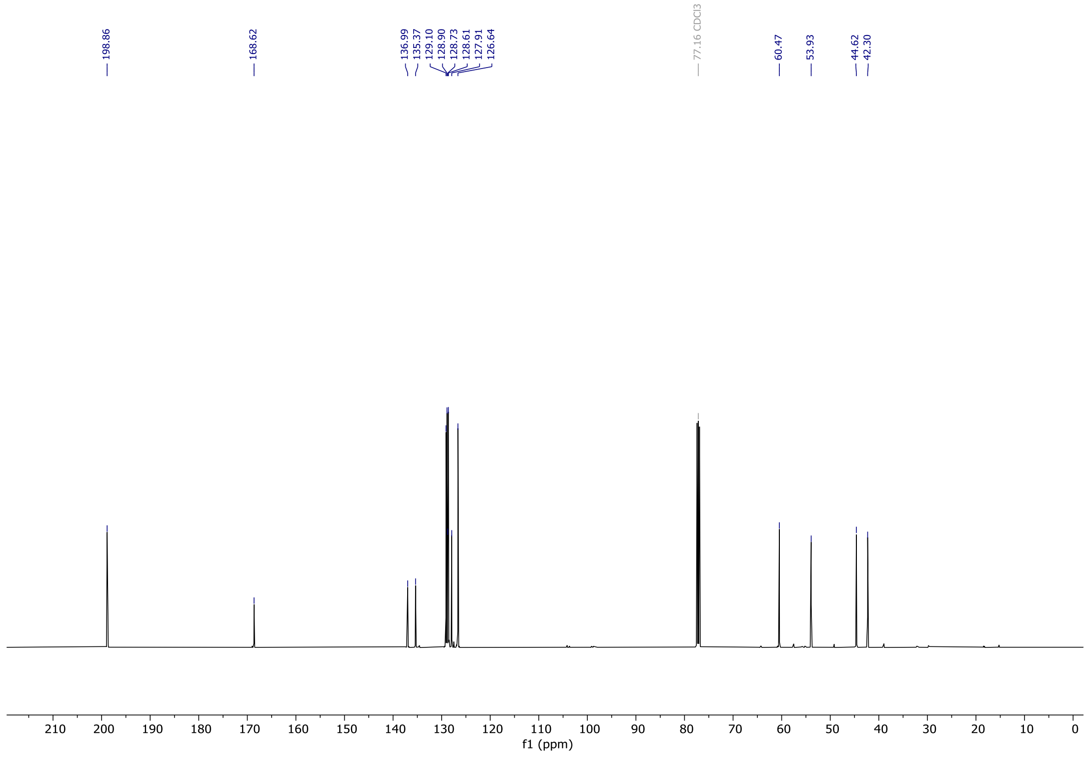

***rac*-1-benzyl-3-ethyl-4-phenylazetidin-2-one (21)**

^{1}H-NMR (400 MHz, $CDCl_3$, 298 K):

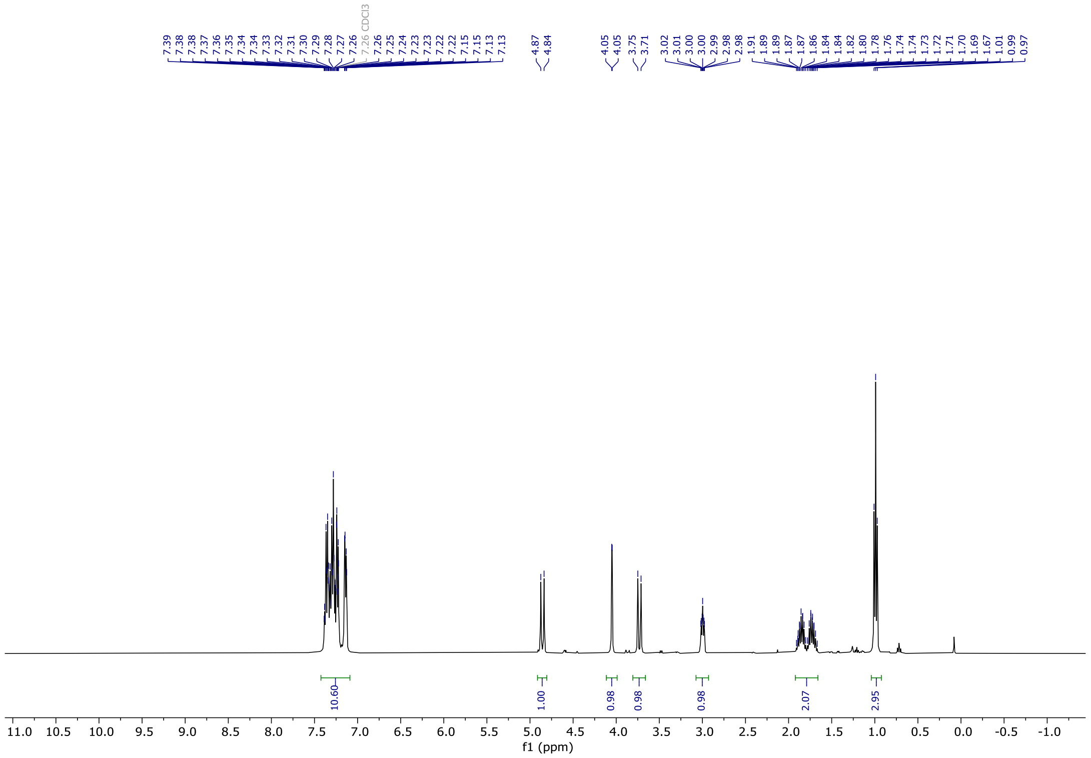

^{13}C-NMR (101 MHz, $CDCl_3$, 298 K):

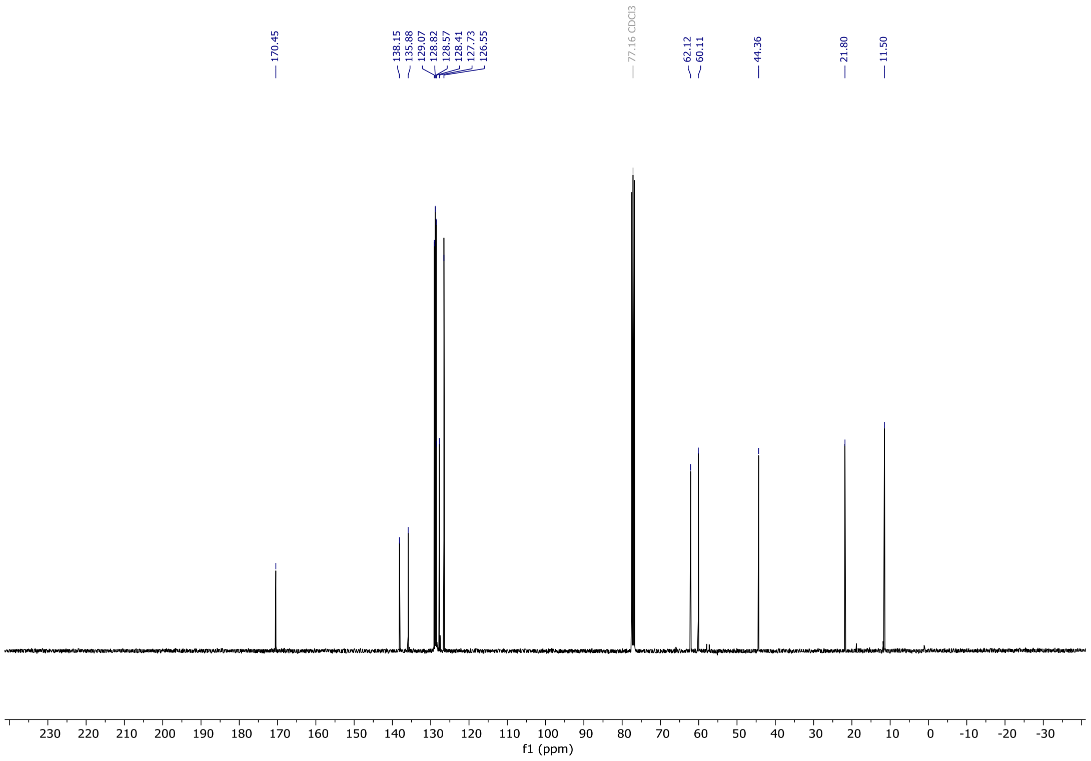

15. References

[1]: *for irradiation setup:* P. Freund, M. Pauls, D. Babushkina, T. Pickl, C. Bannwarth, T. Bach, *J. Am. Chem. Soc.* **2025**, *147*, 1434-1439.

[2]: C. Kerzig, O. S. Wenger, *Chem. Sci.* **2018**, *9*, 6670-6678.

[3]: M. Goez, B. H. M. Hussein, *Phys. Chem. Chem. Phys.* **2004**, *6*, 5490-5497.

[4]: M. Montalti, A. Credi, L. Prodi, M. T. Gandolfi, *Handbook of Photochemistry*, CRC/Taylor & Francis, Boca Raton, **2006**.

[5]: L. D. Elliott, S. Kayal, M. W. George, K. Brooker-Milburn, *J. Am. Chem. Soc.* **2020**, *142*, 14947-14956.

[6]: R. Kumar, E. H. Gleißner, E. G. V. Tiu, Y. Yamakoshi, *Org. Lett.* **2016**, *18*, 184-187.

[7]: M. Horn, J. Ihringer, P. T. Glink, J. Fraser Stoddart, *Chem. Eur. J.* **2003**, *9*, 4046-4054.

[8]: W. Pan, K. Hu, P. Bai, L. Yu, Q. Ma, T. Li, X. Zhang, C. Chen, K. Peng, W. Liu, Z. Sang, *Bioorg. Med. Chem. Lett.* **2016**, *26*, 2539-2543.

[9]: C. E. Hendrick, S. L. McDonald, Q. Wang, *Org. Lett.* **2013**, *15*, 3444-3447.

[10]: C.-H., Ou Yang, W.-H. Liu, S. Yang, Y.-Y. Chiang, J.-J. Shie, *Eur. J. Org. Chem.* **2022**, e202200209.

[11]: Y. Deng, X. Shi, G. Shi, X. Lu, J. Luo, L. Deng, *JACS Au* **2022**, *2*, 2678-2685.

[12]: a) H. V. Pham, A. S. Karns, C. D. Vanderwal, K. N. Houk, *J. Am. Chem. Soc.* **2015**, 137, 6956-6964. b) A. Abbas, B. Xing, T.-P. Loh, *Angew. Chem. Int. Ed.* **2014**, *53*, 7491-7494.

[13]: D.-L. Mo, D. J. Wink, L. L. Anderson, *Chem. Eur. J.* **2014**, *20*, 13217-13225.

[14]: D. J. Aitken, A. Frongia, X. Gaucher, J. Ollivier, H. Rafique, C. Sambiagio, F. Secci, *Tetrahedron Lett.* **2013**, *54*, 2825-2827.

[15]: S. D. Sharma, R. D. Anand, G. Kaur, *J. Chem. Res.* **2005**, *4*, 248-251.

[16]: L. Troisi, C. Granito, E. Pindinelli, *Tetrahedron* **2008**, *64*, 11632-11640.

[17]: J. K. Lam, Y. Schmidt, C. D. Vanderwal, *Org. Lett.* **2012**, *14*, 5556-5569.

[18]: J. Derosa, R. Kleinmans, V. T. Tran, M. K. Karunananda, S. R. Wisniewski, M. D. Eastgate, K. M. Engle, *J. Am. Chem. Soc.* **2018**, *140*, 17878-17883.

[19]: S. Mitra, R. Sarkar, A. Chakrabarty, S. Mukherjee, *Chem. Sci.*, **2022**, *13*, 12491-12497.

[20]: L. Zhang, Z. Zuo, X. Wan, Z. Huang, *J. Am. Chem. Soc.* **2014**, *136*, 15501-15504.

[21]: F. Zhang, N. S. Simpkins, A. J. Blake, *Org. Biomol. Chem.,* **2009**, *7*, 1963-1979.

[22]: M. Faltraccom V. Sukowski, M. van Druenen, T. A. Hamlin, F. M. Bickelhaupt, E. Ruijter, *J. Org. Chem.* **2020**, *85*, 9566-9584.

[23]: C. E. Evans, N. Prud‘homme, M. King, J.C. Scaiano, *J. Photochem. Photobiol. A: Chem.,* **1999**, 121, 105-110.

[24]: H.-J. Timpe, K.-P- Kronfeld, U. Lammel, *J. Photochem. Photobiol. A: Chemistry*,**1990**, *52*, 111-112.

[25]: M. Sakamoto, X. Cai, M. Hara, M. Fujitsuka, T. Majima, *J. Phys. Chem. A*, **2005**, *109*, 2452-2458.

[26]: R. Tang, P. Zhang, H. Li, Y. Liu, W. Wang, *J. Photochem. Photobiol. B: Biol.,* **2011**, *105*, 157-161.

[27]: C. Ley, A. Siedel, T. Bertaux, C. Croutxé-Barghorn, X. Allonas, *Angew. Chem. Int. Ed.,* **2023**, *62,* e202214784.

[28]: J. A. Moghtader, M.-S. Bertrams, D. Schollmeyer, C. Kerzig, *Angew. Chem. Int. Ed.* **2025**, *64*, e202509203.

[29]: M. J. Goodwin, A. M. Deetz, G. J. Meyer, *J. Am. Chem. Soc.* **2025**, *147,* 46794-46799.

[30]: H. N. Kagalwala, J. Gerberich, C. J. Smith, R. P. Mason, A. R. Lippert, *Angew. Chem. Int. Ed.* **2022**, *61*, e202115704.

[31]: T. E. Schirmer, J. C. G. Kürschner, Y. Uchida, Y. Taura, P. Gabriel, L. Næsborg, D. Yokogawa, Y. Aramaki, T. Ooi, *Angew. Chem. Int. Ed.* **2025**, *64*, e202502450.

[32]: G. Morselli, T. H. Eggenweiler, M. Villa, A. Prescimone, O. S. Wenger, *J. Am. Chem. Soc.* **2025**, *147*, 28226-28240.

[33]: P. Müller, K. Brettel, *Photochem. Photobiol. Sci.,* **2021**, *11*, 632-636.

[34]: M. Goez, D. v. Ramin-Marro, M. H. O. Musa, M. Schiewek, *J. Phys. Chem. Lett. A.*, **2004**, *108*, 1090-1100.

[35]: I. Carmichael, G. L. Hug, *J. Phys. Chem. Ref. Data.* **1986**, *15*, 1-250.

[36]: F. Neese, WIREs *Comput. Mol. Sci.*, **2012**, *2*, 73–78.

[37]: M. D. Hanwell, D. E. Curtis, D. C. Lonie, T. Vandermeersch, E. Zurek and G. R. Hutchison, *J. Cheminformatics*, **2012**, *4*, 17.

[38]: ORCA 6.0 Manual https://www.faccts.de/docs/orca/6.0/manual/index.html

[39]: T. J. B. Zähringer, M. Wienhold, R. Gilmour, C. Kerzig, *J. Am. Chem. Soc.* **2023**, *145*, 21576-21586.

[40]: M. V. Popescu, R. S. Paton, *Chem.* **2024**, *10*, 3428-3443.
